# An Atomically Dispersed Copper-Catalyst Lights the Way to Selective *N*-Demethylations

**DOI:** 10.1038/s41467-026-76282-0

**Published:** 2026-08-19

**Authors:** Wenhao Su, Suman Pradhan, Jun Hu, Peng Ren, Nils Rockstroh, Alina Skorynina, Piotr Kuśtrowski, Susanna Monti, Giovanni Barcaro, Chaoqin Zeng, Jabor Rabeah, Aleksander Jaworski, Laura Simonelli, Adam Slabon, Shoubhik Das

**Affiliations:** 1 Department of Chemistry, University of Bayreuth, Universitätsstraße 30, 95447 Bayreuth, Germany; 2 College of Chemical Engineering and Technology, Taiyuan University of Science and Technology, Taiyuan, 030024 China; 3 Leibniz-Institut für Katalyse e.V. (LIKAT Rostock), Albert-Einstein-Straße 29a, 18059 Rostock, Germany; 4 CLAESS Beamline, ALBA Synchrotron Light Facility, Carrer de la Llum 2-26, Cerdanyola del Vallès, Barcelona, 08290 Spain; 5 Faculty of Chemistry, Jagiellonian University, Gronostajowa 2, 30-387 Kraków, Poland; 6 CNR-ICCOM, Institute of Chemistry of Organometallic Compounds, Area della Ricerca, Pisa, I-56124 Italy; 7 CNR-IPCF, Institute for Chemical and Physical Processes, Area della Ricerca, Pisa, I-56124 Italy; 8 State Key Laboratory of Low Carbon Catalysis and Carbon Dioxide Utilization, Lanzhou Institute of Chemical Physics, Chinese Academy of Sciences, Lanzhou, 730000 Gansu China; 9 University of Chinese Academy of Sciences, Beijing, 100049 China; 10 Department of Chemistry, Stockholm University, Stockholm, 10691 Sweden; 11 Chair of Inorganic Chemistry, University of Wuppertal, Gaußstraße 20, 42119 Wuppertal, Germany

**Keywords:** Photocatalysis, Heterogeneous catalysis, Synthetic chemistry methodology

## Abstract

*N*-demethylation is a key transformation in drug metabolism, medicinal chemistry, and late-stage amine diversification. Herein, we report a noble-metal-free, recyclable heterogeneous photocatalyst comprising atomically dispersed Cu sites on functionalized graphitic carbon nitride (Cu@f-gC_3_N_4_) for selective *N*-demethylation and *N*-dealkylation. Under 390 nm irradiation and an oxygen atmosphere, a broad range of tertiary amines, including pharmaceutically relevant substrates, are converted to secondary amines in high yields. The reaction is readily scalable to 10 mmol, and the catalyst retains its activity over more than ten reuse cycles. Atomically isolated Cu sites were confirmed by XPS, ssNMR, STEM, and EXAFS. Mechanistic studies, including radical trapping, isotope labeling, reactive oxygen species detection, kinetic analysis, in situ FTIR, and DFT calculations, support a photoredox pathway involving amine oxidation, superoxide formation, hydrogen abstraction, iminium generation, and hydrolysis, providing a structurally defined and mechanistically understood photocatalytic platform.

## Abstract (web summary)

Herein, the authors present a noble-metal-free, recyclable, and structurally defined heterogeneous photocatalytic platform for selective *N*-demethylation based on atomically dispersed Cu sites supported on functionalized graphitic carbon nitride.

## Introduction

**Fig. 1:**
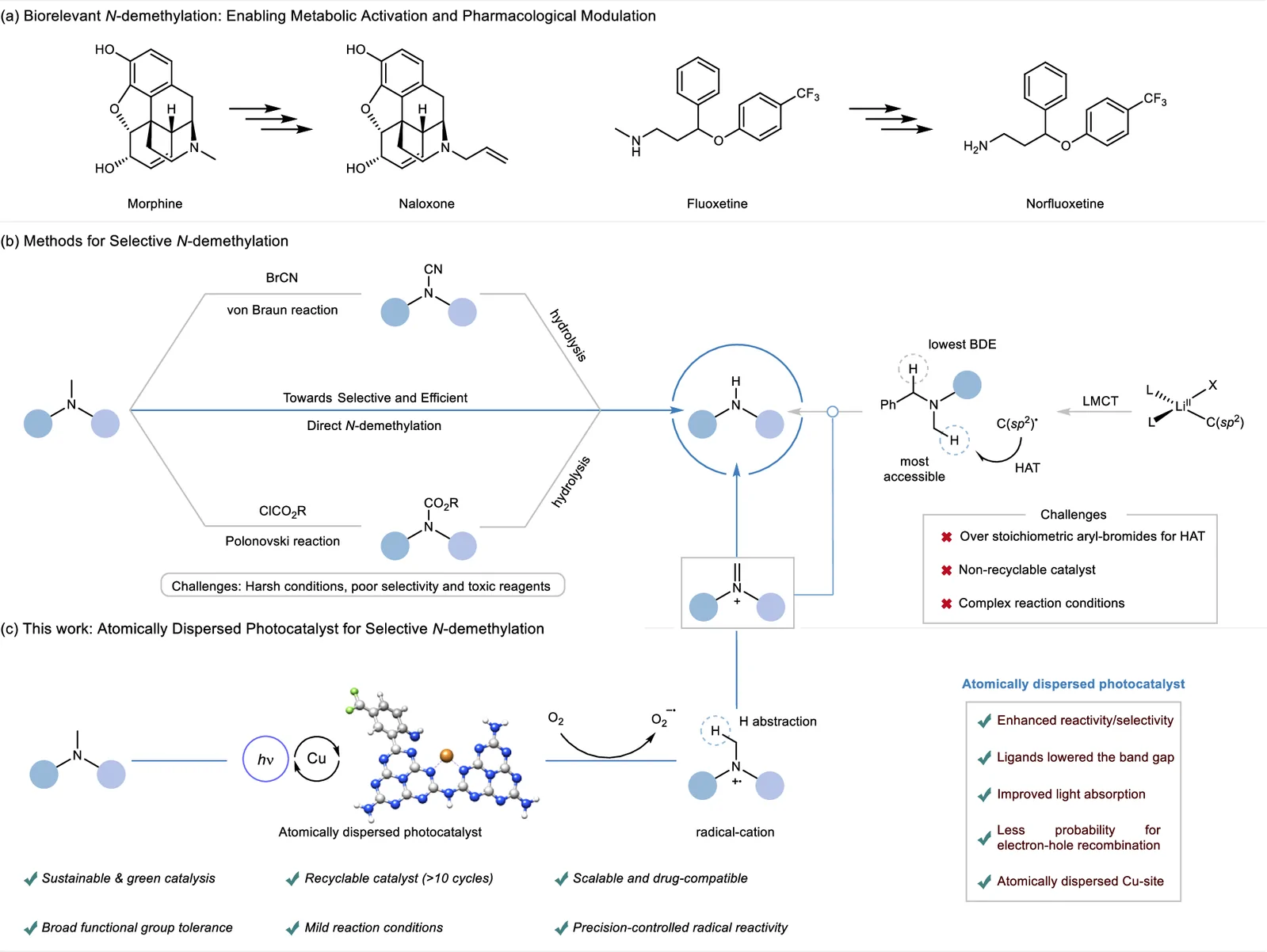
Conceptual framework and reaction design for *N*-demethylation. **a** Biorelevant *N*-demethylation in drug metabolism. **b** Challenges in conventional methods vs photoredox catalysis. **c** Advantages of selective *N*-demethylation by a Cu photocatalyst with high atomic dispersion.

*N*-demethylation reactions play a pivotal role in both biological metabolism and synthetic chemistry. In living systems, enzymatic *N*-demethylation, most notably catalyzed by cytochrome P450 enzymes, is essential for the biotransformation and detoxification of drugs, alkaloids, and other xenobiotics. These reactions convert tertiary or secondary amines into more polar demethylated metabolites, thereby increasing their water solubility and promoting excretion^1,2^. Furthermore, *N*-demethylation can act as a regulatory or activation step, generating intermediates with modified biological activity, such as in the formation of alkaloids and neurotransmitters^3–5^. Beyond biological contexts, controlled *N*-demethylation is a valuable tool in synthetic chemistry for functional group modification, drug metabolite preparation, and fine-tuning the pharmacological or physicochemical properties of nitrogen-containing compounds^6,7^. For example, *N*-demethylation of morphine comprises a crucial step in the synthesis of naloxone, an opioid antagonist essential for reversing opioid overdose (Fig. 1a). Similarly, the *N*-demethylation of fluoxetine produces norfluoxetine, a potent antidepressant that is significantly more selective for stimulating neurosteroid synthesis relative to serotonin reuptake inhibition compared with fluoxetine^8^. Despite its significance, selective *N*-demethylation remains synthetically challenging. Classical methods, including the von Braun and Polonovski reactions, often require toxic or stoichiometric reagents, such as cyanogen bromide or peroxides, and can suffer from limited functional-group tolerance, harsh reaction conditions, and problematic waste generation (Fig. 1b)^9^. In this aspect, transition-metal catalysis (e.g., Pd, Fe) and electrochemical strategies have improved the atom economy; however, challenges remain, including chemoselectivity, overoxidation, substrate compatibility, operational complexity, and catalyst recovery^6,10–12^. In this regard, peroxidase-catalyzed *N*-demethylation reactions have also been demonstrated through a radical mechanism; however, enzymatic approaches often suffer from restricted reaction conditions, including narrow pH ranges and time-consuming enzyme pretreatment, which complicate practical applications^3^. Therefore, the development of mild, selective, and sustainable *N*-demethylation strategies remains important for both synthetic and pharmaceutical applications.

Recently, photoredox catalysis has emerged as a powerful strategy for harnessing light energy to generate reactive species under mild conditions, enabling selective transformations^13,14^. Several elegant homogeneous photoredox and metallaphotoredox systems have demonstrated the synthetic feasibility of this approach. For instance, Rovis et al. recently reported an excellent photoinduced nickel-catalyzed strategy that employed C(*sp*^2^)–bromides as hydrogen atom transfer (HAT) reagents to achieve selective *N*-demethylation of trialkylamines (Fig. 1b)^9^. While this was the first effort to exhibit broad applications as well as selective transformations, several limitations remain, including reliance on stoichiometric amounts of bromides in this reaction and the use of a non-recyclable homogeneous catalyst. Yilmaz and Emmert described a mild aerobic photoredox *N*-dealkylation of tertiary amines using an Ir-based photocatalyst and equimolar nitromethane as a nucleophilic trapping reagent, demonstrating broad substrate tolerance across aliphatic amines, aniline derivatives, and pharmaceutically relevant molecules^15^. These studies have established that oxidative *N*-dealkylation can proceed through pathways involving aminium radical cations, iminium intermediates, reactive oxygen species, and hydrolysis. However, most homogeneous photoredox systems for *N*-demethylation still face limitations in terms of catalyst sustainability and process design. Precious-metal photocatalysts, particularly Ir- or Ru-based complexes, are highly effective but can be expensive, difficult to recover, and less attractive for applications where catalyst recycling, product purification, and metal contamination are important considerations^16^. In addition, homogeneous systems provide limited opportunities to correlate a structurally defined catalytic site with charge separation, oxygen activation, radical generation, and substrate oxidation^17^. Thus, while the elementary reaction manifold of aerobic photoredox *N*-dealkylation is known, translating this chemistry into a recyclable, noble-metal-free, and mechanistically well-defined heterogeneous photocatalytic platform remains an important challenge.

To address these issues, the development of a photocatalyst with a high degree of atomic dispersion offers a promising alternative. Their atomically dispersed metal centers can offer high metal utilization, well-defined coordination environments, and potentially tunable electronic structures, while retaining the practical advantages of heterogeneous catalysts, including separation and reuse^18^. Notably, this class of photocatalyst harnesses the advantages of homogeneous and heterogeneous catalysis by combining molecular-level reactivity with high stability as well as reusability^19,20^. Furthermore, due to their maximal atom utilization efficiency and the advent of advanced characterization techniques, it is now possible to establish detailed structure–activity relationships^21,22^. Graphitic carbon nitride is particularly appealing as a photocatalytic support because of its visible-light absorption, chemical robustness, tunable band structure, and ability to coordinate isolated metal sites through nitrogen-rich motifs. Incorporating earth-abundant transition-metal single sites into functionalized carbon nitride may therefore provide a sustainable strategy for developing recyclable photoredox catalysts that can mediate organic transformations while enabling detailed mechanistic interrogation^23^.

Considering these, we have designed a Cu-catalyst with high atomic dispersion supported on functionalized graphitic carbon nitride (Cu@f-gC_3_N_4_, *E*_VB_ = 1.39 V, *E*_CB_ = –0.93 V vs SCE in MeCN), for selective photocatalytic *N*-demethylation and *N*-dealkylation of tertiary amines under aerobic conditions (Fig. 1c). We argue that under the irradiation of 390 nm light irradiation, this photocatalyst should promote the simultaneous oxidation of amine to the corresponding *N*-radical cations (*E*_1/2_ = 1.04 V vs SCE in MeCN)^24^ and the reduction of molecular oxygen(O_2_) to superoxide radical anions (O_2_^•⁻^, *E*_1/2_ = –0.85 V vs SCE in MeCN)^25^. The superoxide radical anions selectively abstract a hydrogen atom from the methyl group, generating the corresponding iminium intermediate that should subsequently undergo hydrolysis to afford the corresponding secondary amines^26^. Furthermore, we rationalize that the use of a polar-protic solvent and a base is necessary to obtain the high selectivity as well as reactivity, since both of them stabilize the transient reactive species and enhance the proton transfer efficiency for further improvement in reaction selectivity^27,28^.

## Results and discussion

### Synthesis of the photocatalyst

At the outset of the project, Cu dispersed on aryl amino-substituted g-C_3_N_4_ (Cu@f-gC_3_N_4_) was prepared via a two-step process involving successive impregnation and calcination (Supplementary Information for the detailed synthesis procedure)^29^. In a typical synthesis, dicyandiamide (DCDA, 9 g) and 2-amino-5-(trifluoromethyl) benzonitrile (0.150 g, 1.6 wt%) were homogeneously mixed with Cu(NO_3_)_2_•3H_2_O (0.019 g, 0.2 wt%). The mixture was then stirred and heated in water (45 mL) at 95–100 °C until it was completely dried. The resulting mixture was removed, ground in an agate mortar, and loaded into a stainless-steel chamber. Subsequently, the GERO carbolite tube furnace chamber was heated for 244 min ( ~ 4 h) until it reached 550 °C under an aerobic atmosphere and was maintained for 4 h. After that, the furnace chamber was cooled to room temperature in another 6 h.

After synthesizing the photocatalyst, ultraviolet-visible diffuse reflectance spectroscopy (UV-Vis DRS) and Mott-Schottky measurements were collected to determine the photocatalyst’s electronic band structure (Supplementary Fig. 10). A band gap of 2.65 eV for the Cu@f-gC_3_N_4_ was derived from the Tauc plot. The positive slope of the Mott-Schottky curve suggested that the photocatheyst was n-type. The flat band potential (*E*_fb_) of Cu@f-gC_3_N_4_ was approximately –0.96 V (vs SCE), as determined from the intersection between the Mott-Schottky plot and the baseline. In an n-type semiconductor photocatalyst, the conduction band generally lies 0.1 to 0.2 eV above the flat-band potential, with this offset influenced by factors such as electron effective mass and carrier concentration. So, the conduction band potential (*E*_CB_) of Cu@f-gC_3_N_4_ was approximately –0.93 V (vs SCE). Based on *E*_VB_ = *E*_CB_ + *E*_g_, the valence band (VB) of Cu@f-gC_3_N_4_ was at ca. +1.39 V (vs SCE).

### Optimization of the reaction conditions

Initially, the reaction was optimized using *N,N*-dicyclohexylmethylamine (**1**) as the starting material and the synthesized Cu@f-gC_3_N_4_ as the photocatalyst. Systematic variation of key reaction parameters, including the base, oxidant, and solvent, revealed the optimal conditions. When 0.2 mmol of substrate was reacted under 390 nm irradiation using Cu@f-gC_3_N_4_ (5 mg) and CsOPiv (1.0 equiv) in ethanol (0.1 M) under an O_2_ atmosphere, the desired product, dicyclohexylamine (**2**), was obtained in 89% yield (Table 1, entry 1). Further examination of the bare f-gC_3_N_4_ (Cu-free) as a photocatalyst exhibited poor catalytic reactivity, giving only 30% yield of the product (Table 1, entry 8), which further demonstrated the importance of Cu-doping in this reaction. The effect of the solvent in this reaction was significant. In fact, replacing ethanol with MeCN or MeOH led to reduced yields of the desired product (50% and 62%, respectively; Table 1, entries 2 and 4). This observation clearly suggested that protic solvents were more favorable for this transformation, likely because they facilitated reduction of molecular oxygen, resulting in the formation of water^30^, which played a crucial role in the hydrolysis step to generate the final product. To verify this hypothesis, deuterium-labeling experiments were conducted, and the result confirmed that EtOH itself served as a hydrogen source under the reaction conditions (the deuterium labeling experiments in Supplementary Information for details). In contrast, in aprotic solvents such as MeCN, the methyl group served as the only hydrogen source, limiting oxygen reduction and leading to a lower product yield. Furthermore, different bases were evaluated, and it was observed that the weaker base CsOPiv was the most effective in this transformation. In fact, replacing this base with NaOPiv or KOPiv resulted in diminished product yields of 40% and 49%, respectively (Table 1, entries 5 and 6). Considering the apparent role of in situ water formed during photocatalysis in the reaction mechanism for this transformation, the deliberate addition of water was further evaluated; however, the introduction of 10 equivalents of water led to a reduced yield of 69% (Table 1, entry 7). This suggested that externally added bulk water did not improve the reaction efficiency and, in fact, could be detrimental which could be due to the fact that the solubility of the organic substrate was decreased in an aqueous medium or the catalytic reactivity was reduced in the presence of excess water, as we observed the catalyst adhering to the walls of the vessel after the reaction. Encouraged by all these observations, control experiments were conducted to assess the necessity of each reaction component. In fact, the reaction did not proceed in the absence of the photocatalyst, base, oxygen gas, or light (Table 1, entries 10–13), confirming the essential role of these components. The reaction was successfully scaled up to a 10 mmol (1.65 g) scale, affording the desired product in 71% yield, which clearly demonstrates the scalability of our protocol.

**Table 1:** Optimization of the reaction conditions^a^


Entry	Deviation from model reaction conditions	Yield (%)^b^
1	None	89
2	MeOH	62
3	TFE	20
4	MeCN	50
5	NaOPiv	40
6	KOPiv	49
7	H_2_O (10 equiv.)	69
8	gC_3_N_4_	23
9	f-gC_3_N_4_	30
10	no cat.	trace
11	no light	n.r.
12	no base	21
13	no O_2_	n.r.

^a^model reaction conditions: 1 (0.2 mmol), Cu@f-gC_3_N_4_ (5.0 mg), CsOPiv (1.0 equiv.), anhydrous ethanol (2.0 mL), O_2_ atmosphere (balloon).

^b^ ^1^H NMR yield using mesitylene as internal standard. n.r. = no reaction.

Under the applied photocatalytic conditions, only tertiary amines underwent *N*-dealkylation owing to favorable redox matching with the photocatalyst ( + 1.39 V vs SCE). In contrast, the higher oxidation potential of the resulting secondary amines rendered further oxidation thermodynamically less favorable, thereby preventing over-dealkylation. The effect of the base on this transformation is comparatively minor, as NaOPiv, KOPiv, and CsOPiv possess similar basicity. The variations in yield observed among these bases likely arise from cation-dependent factors such as solubility and ion pairing rather than intrinsic basic strength.

**Fig. 2:**
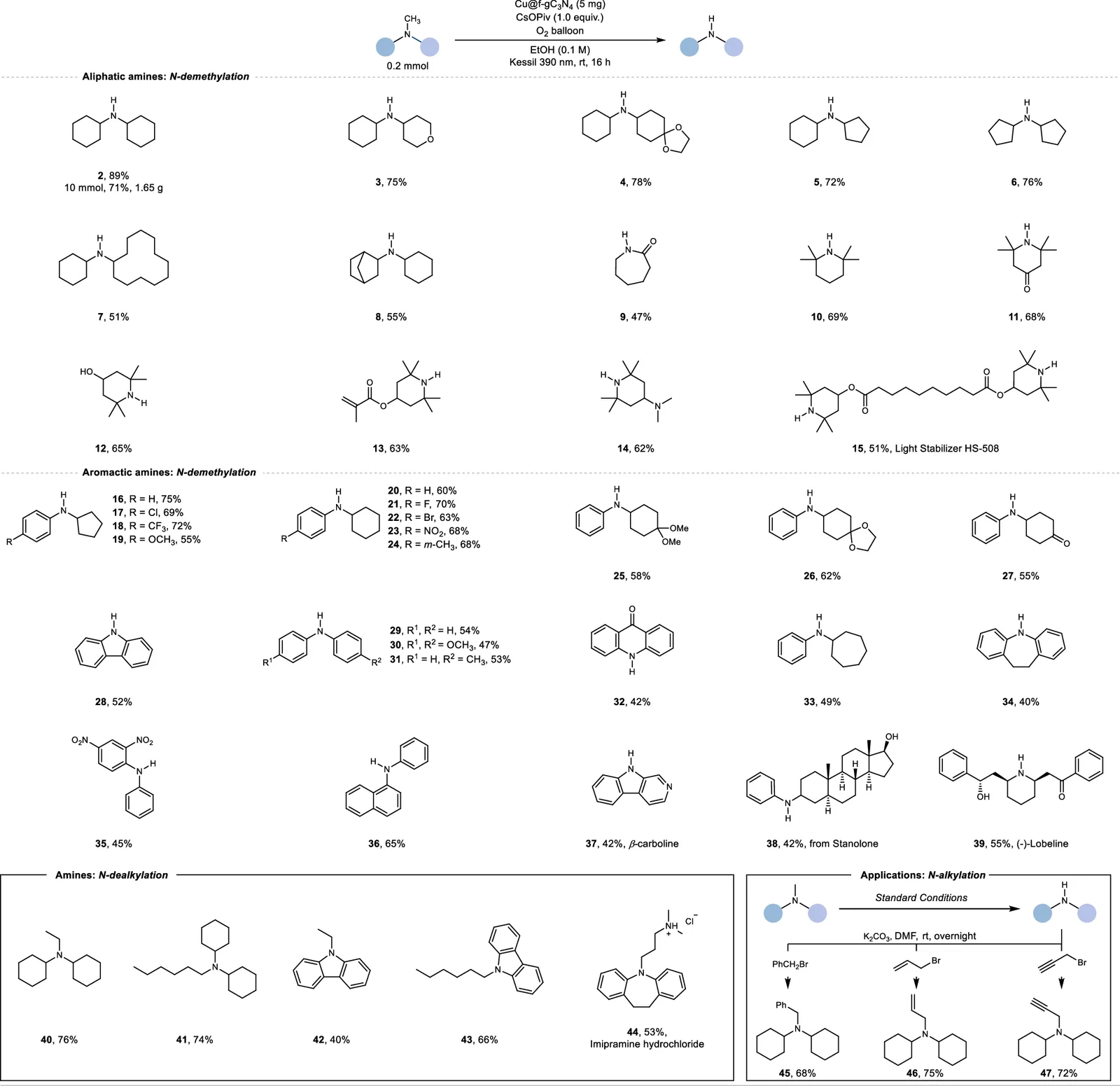
Substrate scope for *N*-demethylation. Reaction conditions: amines (0.2 mmol), CsOPiv (1.0 equiv.), Cu@f-gC_3_N_4_ (5 mg), ethanol (0.1 M), O_2_ atmosphere, light source: 390 nm light, cooling with a fan, 16 hours. ^1^H NMR yields were determined using mesitylene as an internal standard.

After optimizing the reaction conditions, the generality of this photocatalytic *N*-demethylation protocol was evaluated by applying a broad range of aliphatic and aromatic tertiary amines (Fig. 2). In fact, a variety of cyclic and acyclic aliphatic tertiary amines were well tolerated, affording the corresponding secondary amines in moderate to excellent yields (47–89%). Furthermore, different derivatives of dicyclohexylamine (**3**–**8**) were efficiently demethylated, with yields ranging from 51% to 76%, highlighting the applicability of our reaction protocol to sterically hindered amines. Other cyclic amines, including 1-methylazepan-2-one (**9**, 47%), 1,2,2,6,6-pentamethylpiperidine (**10**, 69%), and *N*,*N*−1,2,2,6,6-heptamethylpiperidin-4-amine (**14**, 62%), also underwent efficient demethylation. Additionally, functionalized aliphatic amines, including a hydroxy-substituted amine (**12**, 65%) and an ester-containing substrate (**13**, 63%), were well tolerated under the reaction conditions. Moreover, a complex light stabilizer (HS-508) was successfully demethylated (**15**, 51%), highlighting the method’s applicability to functionalized materials. Aromatic amines also proved to be excellent substrates, with electron-donating and electron-withdrawing substituents well tolerated. *N*-Methylanilines with various *para*-substituents (-Cl, -CF_3_, -OCH_3_) afforded moderate to good yields (55–75%). Furthermore, electron-deficient systems such as nitro- and halogen-substituted anilines reacted efficiently (60–70%). Additionally, heterocyclic amines, including 9-methyl-9*H*-carbazole (**28**, 52%), 10-methylacridin-9(10*H*)-one (**32**, 42%), and 5-methyl-10,11-dihydro-5*H*-dibenzo[*b*,*f*]azepine (**34**, 40%), were successfully demethylated. In addition to *N*-demethylation, this strategy is also applicable to various *N-*alkyl substrates, providing dealkylated secondary amines in 74–76% yield. Similarly, for *N*-alkylated 9*H*-carbazole (**42,** **43**) and imipramine hydrochloride (**44**), partial *N*-dealkylation was noted (40–66% yields), suggesting that steric and electronic effects influenced the site-selective cleavage. Notably, this photocatalytic *N*-demethylation strategy was also successfully applied to pharmaceutically relevant compounds, highlighting its potential for late-stage transformation in medicinal chemistry. (–)-Lobeline, a bioactive alkaloid with neuromodulatory properties, underwent selective demethylation to yield the corresponding secondary amine (**39**, 55%). Similarly, imipramine hydrochloride, a widely used tricyclic antidepressant (TCA), was converted to 10,11-dihydro-5*H*-dibenzo[*b*,*f*]azepine (**44**, 53%), a privileged scaffold in medicinal chemistry and a core structure of carbamazepine, an anticonvulsant drug widely used in the treatment of epilepsy and bipolar disorder. Furthermore, *β*-carboline derivatives, which are implicated in neuroactive and psychoactive effects, also reacted efficiently (**37**, 42%), highlighting the compatibility of our method with complex alkaloid scaffolds. Additionally, a steroid-derived substrate from stanolone (**38**, 42%) underwent successful demethylation, indicating that this protocol was applicable to hormonally active molecules, further expanding its relevance in drug metabolism studies. To further demonstrate the synthetic utility of our photocatalytic *N*-demethylation strategy, the late-stage transformation of the resulting secondary amine was explored via in situ *N*-alkylation (Fig. 2). In this approach, the demethylation product, a valuable secondary amine, served as a versatile synthetic intermediate, enabling facile derivatization through further alkylations with diverse electrophiles. For example, upon treatment with benzyl bromide, the secondary amine was efficiently converted into the *N*-benzylated product (**45**, 68%), providing a direct route to structurally relevant tertiary amines. Similarly, reaction with allyl bromide furnished the *N*-allylated amine in 75% yield (**46**), while propargylation with propargyl bromide afforded the corresponding *N*-propargylated derivative in 72% yield (**47**). These transformations highlighted the broad scope of downstream functionalization, offering access to pharmaceutically and chemically relevant amine scaffolds. This approach underscored the practicality and versatility of the photocatalytic *N*-demethylation protocol, facilitating efficient late-stage amine diversification. It should be noted that the ability to selectively modify the amine functionality without affecting the rest of the molecular framework makes our strategy a robust and valuable approach for the modification of complex molecules in medicinal chemistry, drug discovery, and materials science.

### Characterization of the photocatalyst

#### STEM

**Fig. 3:**
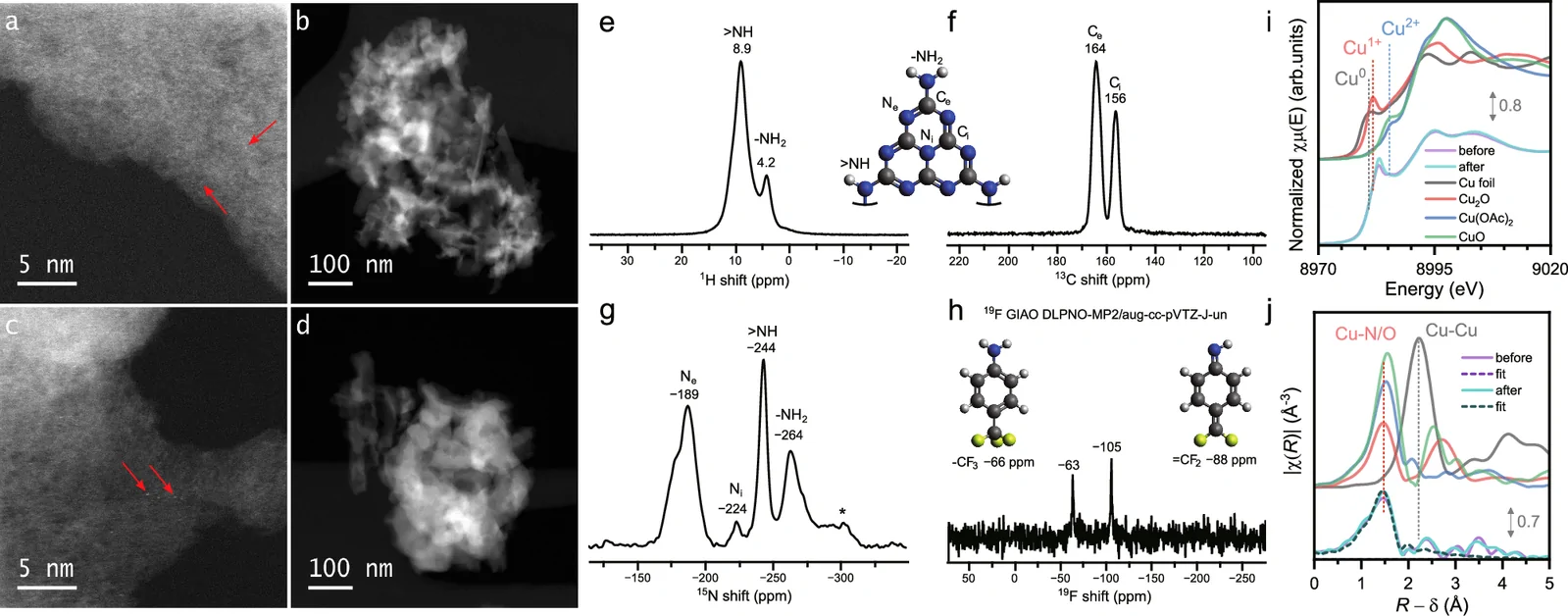
Characterization results of Cu@f-gC_3_N_4_ photocatalyst. **a**–**d** Selected HAADF–STEM images of the photocatalyst at different magnifications before (a-b), and after reaction (**c**, **d**), respectively. Red arrows show examples of Cu single sites. **e** ^1^H MAS, (**f**) ^1^H–^13^C CPMAS, **g** ^1^H–^15^N CPMAS, and **h** ^19^F MAS NMR spectra of Cu@f-gC_3_N_4_ Photocatalyst at 14.1 T. Spinning sideband is marked with an asterisk. **i** Normalized Cu K-edge XANES spectra. **j** FT-EXAFS spectra of Cu@f-gC_3_N_4_ photocatalyst, Cu foil, Cu_2_O, Cu(II) acetate, and CuO. FT spectrum of the Cu foil was scaled down by a factor of 2.2 to facilitate visual comparison with the catalyst spectra.

To confirm the presence of copper (Cu) atoms on the gC_3_N_4_ support, scanning transmission electron microscopy (STEM) coupled with energy dispersive X-ray spectroscopy (EDX) was performed. As visible from the contrast in the high-angle annular dark field (HAADF) image shown in Fig. 3a, Cu is present as single atoms. The presence of Cu was verified by EDX spectroscopy (Supplementary Fig. 12). Due to the low content of Cu in the sample, the absence of nanoparticles and small clusters (Fig. 3b) cannot be ruled out unambiguously by this method as their number would be very small. However, the observation of single atoms as well as the weak Cu signal obtained by EDX at various regions of the material provides a strong indication of the presence of Cu in dispersed form. Analysis of the photocatalyst after the reaction (Fig. 3c, d) showed that the principal structure of the material is retained, with single atoms still being present.

#### SS-NMR study

To probe the local structure of the photocatalyst and assess structural changes upon Cu^2+^ doping, solid-state MAS NMR was conducted (Fig. 3e–h). The ^1^H MAS, ^13^C CPMAS, and ^15^N CPMAS spectra (Fig. 3e–g) exhibited very similar appearance and chemical shifts for related polymeric carbon nitride catalysts^31–34^. ^1^H NMR signals from the terminal (surface) -NH_2_ groups and the bridging >NH linkers were observed at 4.2 and 8.9 ppm, respectively. Functionalization of the graphitic carbon nitride was evidenced by the ^19^F MAS NMR spectrum shown in Fig. 3h. Two observed ^19^F NMR signals at −66 and −105 ppm indicated the presence of -CF_3_ and =CF_2_ groups in the material^34–36^. To corroborate assignments of these ^19^F NMR signals, chemical shifts were calculated for both -CF_3_ and =CF_2_ scenarios by considering respective models shown in Fig. 3h. The results suggested that the precursor underwent partial defluorination and was present in the material as a mixture of two structures shown in Fig. 3h.

#### XPS

The surface composition of Cu@f-gC_3_N_4_ was further confirmed by X-ray photoelectron spectroscopy (XPS) (Supplementary Fig. 13). The spectra exhibited characteristic peaks attributed to the g-C_3_N_4_ phase: (i) at 287.8 eV in the C 1 s region, assigned to *sp*^2^-bonded C atoms in N-C = N, as well as (ii) at 398.5 eV (*sp*^2^-hybridized N atoms in C-N = C), 400.2 eV (bridging N atoms in N-(C)_3_) and 401.1 eV (N atoms in amino groups) in the N 1 s region^37,38^. The XPS Cu 2p spectrum showed no distinct peaks, indicating a Cu content below the detection limit on the surface.

#### XANES and EXAFS experiments

The presence of isolated Cu single-atom active sites was further confirmed by analyzing Cu@f-gC_3_N_4_ using XANES and EXAFS, which revealed the valence state and local coordination of the Cu atoms in the support matrix, respectively. As shown in Fig. 3i, the Cu K-edge XANES spectra of the Cu@f-gC_3_N_4_ photocatalyst lie between those of Cu_2_O and CuO, indicating a partial positive oxidation state (Cu^δ+^, 1<δ < 2)^39^. To probe the local coordination environment, Fourier-transformed k-weighted EXAFS spectra in R-space were recorded (Fig. 3j). For Cu@f-gC_3_N_4_, a prominent scattering peak was observed at ~1.5 Å (uncorrected for phase shift), corresponding to the first coordination shell of Cu–N/O bonds. A weaker peak appeared at ~2.4 Å, consistent with Cu–C scattering, similar to that found in the molecular complex C_32_H_16_CuN_8_, which likely represented the second coordination shell. The absence of a peak near ~2.2 Å, characteristic of Cu–Cu scattering, suggested that Cu was not present in metallic form, in agreement with the XANES part of the spectra. Moreover, the very low spectral contribution above 2 Å most likely corresponds to a Cu isolated atom/ion form, dispersed on a modified g-C_3_N_4_ matrix via Cu–N–C bonding^39–41^, well in agreement with the HAADF-STEM results described above.

#### EPR

Electron paramagnetic resonance (EPR) spectroscopy was also employed to confirm the presence of isolated copper single-atom sites within the Cu@f-gC_3_N_4_ catalyst. The resulting spectrum (Supplementary Fig. 15 A) provides definitive structural insights and clearly exhibits the characteristic signal of isolated paramagnetic Cu^2+^ ions. Notably, the spectrum displayed a distinct 9-line superhyperfine splitting pattern of the XY spin Hamiltonian component. This pattern arises from the magnetic coupling between an unpaired Cu^2^⁺ electron (S = 1/2) and four equivalents, directly coordinated ^14^N nuclear spin (I = 1) within the graphitic carbon nitride support matrix. This provides unambiguous evidence that copper ion is immobilized as an isolated single-atom site, coordinated to four nitrogen donors within the f-gC_3_N_4_ framework.

#### Mechanistic investigations

**Fig. 4:**
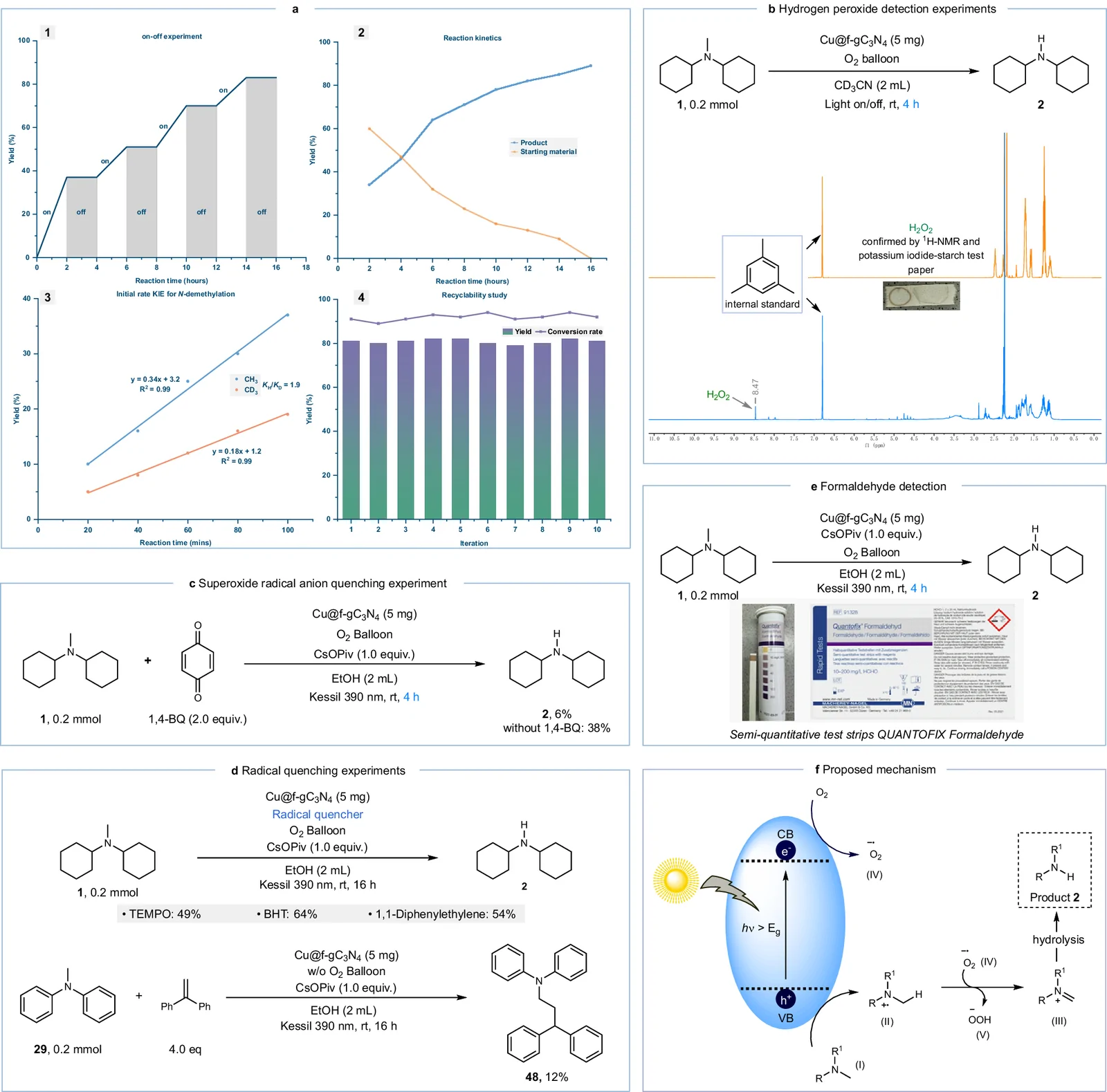
Compilation of experimental mechanistic studies. **a** Light on–off control, kinetic profile, catalyst recyclability, and initial rate KIE for photocatalytic *N*-demethylation; **b** Detection of H_2_O_2_ formation; **c** Quenching of superoxide radical anion (O_2_•⁻); **d** Radical trapping experiments with different scavengers; **e** Detection of formaldehyde as the oxidation product; **f** Proposed mechanism consistent with the experimental findings.

To elucidate the mechanism of this photocatalytic *N*-demethylation, a series of control experiments, radical-trapping studies, isotope-labeling experiments, in-situ EPR, in situ FTIR, and kinetic analyses were conducted, all of which suggested a radical-mediated oxidative pathway under photocatalytic conditions (Fig. 4). Furthermore, an on-off experiment demonstrated that product formation occurred only under continuous light irradiation, confirming the necessity of photoactivation for the reaction to proceed, and this photocatalytic pathway followed a redox process rather than an energy-transfer process (Fig. 4a–1). Reaction kinetics revealed a steady consumption of the tertiary amine, accompanied by a concurrent increase in the secondary amine product (Fig. 4a-2). To evaluate the robustness of the catalyst, recyclability experiments were performed for ten consecutive cycles without any significant loss of yield (Fig. 4a-3). Furthermore, a kinetic isotope effect (KIE) study revealed a *K*_H_/*K*_D_ value of 1.9, indicating that the cleavage of C–H bond was involved in the rate-determining step (Fig. 4a-4).

The presence of reactive oxygen species (ROS) was confirmed through multiple detection methods. At first, hydrogen peroxide (H_2_O_2_) was detected via ¹H-NMR and KI-starch test strips, suggesting that oxygen reduction played a role in the reaction (Fig. 4b). This observation was further confirmed by spin-trapping EPR spectroscopy, which revealed the formation of the DMPO-superoxide anion radical spin adduct (DMPO-^•^O_2_^−^) upon irradiation of Cu@f-gC_3_N_4_ in ethanol under aerobic conditions (Supplementary Fig. 15B). Additionally, quenching experiments using 1,4-benzoquinone (BQ), a known superoxide radical scavenger, resulted in a significant drop in yield, indicating the involvement of O_2_-derived radical species (Fig. 4c). Furthermore, radical-trapping experiments using 2,2,6,6-tetramethylpiperidinyloxyl, butylhydroxytoluol, and 1,1-diphenylethylene also yielded diminished yields, supporting the presence of radical intermediates (Fig. 4d). It is important to note that the experiment with 1,1-diphenylethylene was conducted without the use of an O_2_ balloon, resulting in a very low yield of the product (**48**, 12%). This suggested that, in the absence of oxygen or the superoxide radical anion, the reaction likely proceeded through the deprotonation of the aminyl radical cation, forming a methylene radical intermediate which then reacted with the 1,1-diphenylethylene species to produce the product, **48** (Fig. 4d). The detection of formaldehyde by using formaldehyde test strips confirmed that the demethylation process proceeded through the cleavage of C–N bond, releasing formaldehyde as a byproduct (Fig. 4e). Furthermore, the demethylation of the tertiary amine substrate (*N,N*-dicyclohexylmethylamine) was further confirmed by EPR spectroscopy which showed a distinct three-line EPR signal at g = 2.003 with a hyperfine coupling constant of 15 G under aerobic irradiation of *N,N*-dicyclohexylmethylamine indicating the formation of nitrogen-centered radical (Supplementary Fig. 15C, intermediate (II)). These radical-mediated mechanistic insights were independently corroborated by transmission-mode Fourier-transform infrared (FTIR) spectroscopy (Supplementary Fig. 7A–C). The FTIR spectrum exhibited a marked reduction in the intensity of the vibrations bands at 1020–1080 and 2750–3050 cm^−1^ of the parent *N,N*-dicyclohexylmethylamine substrate accompanied by the emergence of a new shoulder at 1610-1710 cm^−1^. This spectral change is consistent with the formation of the secondary amine product, dicyclohexylamine, confirming the successful demethylation of the starting tertiary amine. Moreover, the FTIR spectra provide direct evidence for the proposed C = N^+^ iminium intermediate pathway. The transient, time-dependent band centered around 1630-1660 cm^−1^ (Supplementary Fig. 7) corresponds to the C = N^+^ stretch.

**Fig. 5:**
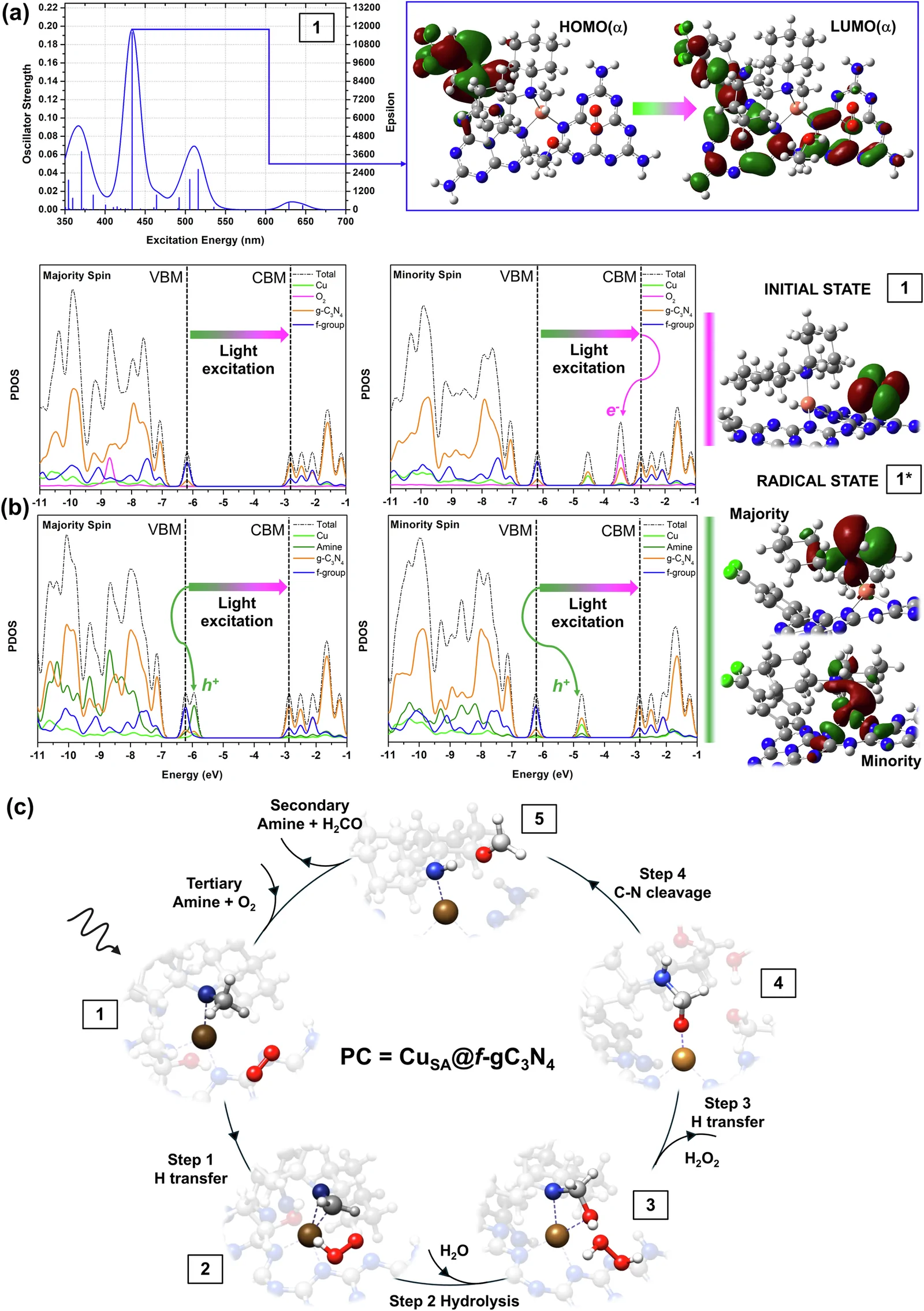
Excited-state dynamics and catalytic cycle evolution. **a** UV simulation of the local minimum shown in Fig. 5a, reporting the main orbitals involved in the transition at 434 nm. **b** Majority and minority PDOS of the same structure, showing the orbitals within the minority spin states where *h*^*+*^ (in green) and *e*^*-*^ (in magenta) can migrate as a result of the HOMO-LUMO transition indicated in the picture. **c** Catalytic cycle reporting the local minima with a focus on the evolution of the chemical environment around the *N* atom of the amine substrate. Color coding: C gray, O red, N blue, H white, F green, and Cu orange.

Encouraged by all this information, molecular modeling calculations, including classical molecular dynamics simulations based on a reactive force field (ReaxFF-RMD), and quantum-chemical calculations at the density functional theory (DFT) level, were used to support/validate the experimental characterizations and to elucidate the mechanism of the *N*-demethylation reaction. The RMDs were employed solely to define/characterize the salient features of a supramolecular framework in ethanol solutions (explicit molecules) to confirm the validity of a representative small system for the quantum-chemistry calculations (all the details of the simulations are reported in the SI). The model of the g-C_3_N_4_ PC developed earlier^29,31,42^ could provide insight into possible coordination environments, relative energetic trends, and reaction steps, rather than reproducing the experimental photocatalytic processes. The model consisted of two melem units coordinated to a Cu atom via two lateral nitrogens and functionalized with an aryl amino moiety (f-gC_3_N_4_, Fig. 5). The reaction pathway was reproduced through the GGA-Plane-Wave (PW) method and the Nudged Elastic Band (NEB) algorithm. In contrast, excitation profiles, projected density of states (PDOS), and natural bond orbital (NBO) charges were obtained using a localized-based method with a hybrid XC-functional. The calculations were focused on the aliphatic tertiary amine reported as compound **2** in Fig. 2.

**Fig. 6:**
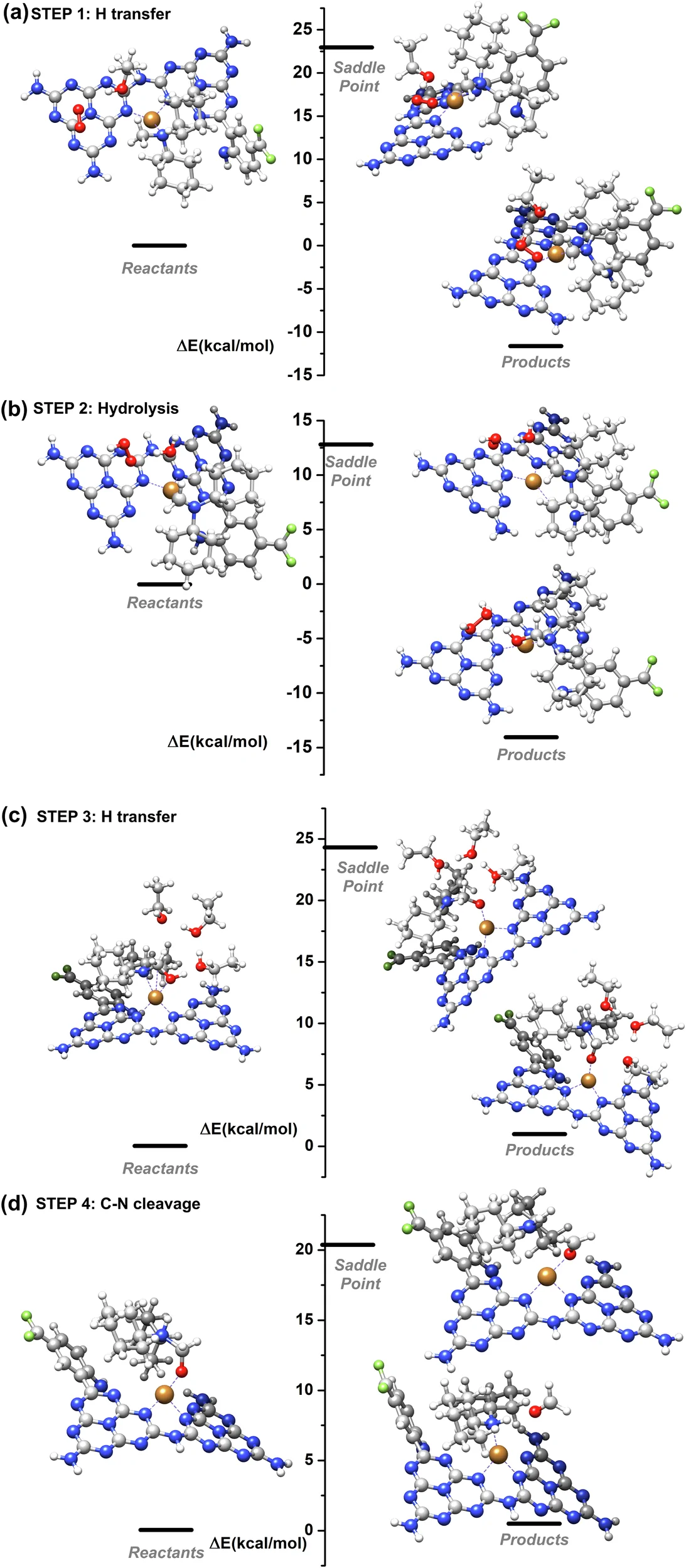
Stepwise mechanism of the *N*-demethylation process. **a**–**d** The four steps of the *N*-demethylation process modeled by using substrate 2 from Fig. 2. In each panel, the structures of the reactants, products, and transition states are shown, along with an estimate of the energy barrier. Color coding: C gray, O red, N blue, H white, F green, and Cu orange.

Figure 6a (and Supplementary Fig. 20a) showed the local minimum corresponding to the starting configuration of the *N*-demethylation process, where substrate **2** was adsorbed via its *N* atom on Cu in the presence of an O_2_ and an EtOH molecule from the solvent. Examination of the simulated UV absorption profile shown in Fig. 5a revealed that a resonance enhancement, favored by the presence of the aryl moiety, was responsible for the strong absorption peak located at 434 nm. Such a peak was dominated by a HOMO (VB Maximum)-LUMO (CB Minimum) transition, in which the HOMO was localized on the aryl moiety and the LUMO was mainly delocalized over the melem units of the g-C_3_N_4_ support (as shown in the orbital plot in Fig. 5a). By inspecting the corresponding PDOS (Fig. 5b), the presence of virtual states within the band gap of the minority-spin energy level scheme was apparent. The shape of such orbitals (Fig. 5b) confirmed that, upon irradiation, the excited electrons populated the empty states of the O_2_ molecule, leading to the stabilization of a superoxide radical O_2_•⁻. This latter species induced hydrogen abstraction from the CH_3_ moiety of the tertiary amine, leading to the formation of an O_2_H⁻ species and a radical intermediate on the tertiary amine. The PDOS of this radical revealed occupied states within the band gap above the VBM, localized on the Cu and N atoms. These states can induce hole migration (as shown in the lower panels of Fig. 5b) stabilizing an iminium cation intermediate bound to the Cu SAC.

Hydrogen migration was studied at the DFT level in its ground state (without charge separation), and reactants, products, and saddle species are shown in Fig. 6a. Assuming that a solvent molecule drives the hydrogen-transfer process, the energy barrier was approximately 23 kcal/mol. In the structure of the products, the O_2_H species was not bound to the g-C_3_N_4_ support, and another minimum, where O_2_H was adsorbed on a C atom of the support (Supplementary Fig. 20b), next to the Cu SAC, was found. In the absence of charge separation, this bound configuration was about 16 kcal/mol higher in energy. However, a stabilization of the corresponding charged species (O_2_H⁻), as discussed by Shirashi et al. in the investigation of O_2_ reactivity in the presence of photoexcited g-C_3_N_4_ was expected^43^. Regarding the evolution of the substrate, the NBO charge analysis suggested that the hydrogen transfer was accompanied by an electronic depletion (of about 0.5 e^-^) of both the *N* and the alpha-C atoms (the carbon of the former CH_3_ moiety) of the imine. The presence of holes, resulting from charge separation induced by photoexcitation, sustained the electronic depletion, favoring H transfer.

The following steps of the reaction involved the hydrolysis of the iminium ion intermediate, leading to the formation of the final products. The single steps of the overall process were displayed in Fig. 6, whereas the catalytic cycle was summarized in Fig. 5c. The electrophilic carbon of the iminium ion was attacked by a water molecule formed in situ (Step 2), which was dissociated in a OH⁻ (which was inserted between the electrophilic carbon of the substrate and the Cu atom of the PC) and H^+^, migrating on the O_2_H⁻ species, restoring overall neutrality and leading to the formation of one of the expected products, i.e. H_2_O_2_. This step was investigated on the ground-state potential energy surface. Reactants, products, and the transition state are shown in Fig. 6b. The identified energy barrier was about 13 kcal/mol. Successively, an H atom could be transferred from the terminal -OH group to the N atom of the substrate. Assuming that this transfer, too, could be mediated by the action of the EtOH molecules, we inserted three EtOH molecules. We found an energy barrier of about 24 kcal/mol, which was similar to that of the first H transfer (Step 3, Fig. 6c). Then, modeling the C–N cleavage (Step 4, Fig. 6d) led to the formation of another experimentally observed product, namely formaldehyde, and to the stabilization of the secondary amine bound to the Cu ion of the PC. The barrier of this last process was approximately 20 kcal/mol.

A recent paper by Colonnello et al.^44^ provided a spectroscopically confirmed reason to question the active photocatalytic role of single-atom copper on carbon nitride supports, revealing that the smooth, continuous catalytic cycle often depicted for Cu may be an idealized model that fails in real systems due to irreversible structural collapse. However, in the present system, structural collapse appears to be prevented by the vicinal aryl ligand, which rigidifies the local nitrogen coordination environment. This rigidity imposes a significant steric and energetic penalty, thereby hindering the transition toward a linear geometry that would otherwise lead to an irreversible reduction of the metal site, as reported in the cited paper. Furthermore, virtual states within the band gap of oxygen molecules facilitate the transfer of photoexcited electrons, thereby forming superoxide radical species. This mechanism prevents reduction of the copper center, allowing it to retain a + 2 oxidation state throughout the catalytic cycle. Such behavior is consistent with the experimentally observed high recyclability of the photocatalyst.

Based on the experimental mechanistic investigations and DFT calculations, the catalytic mechanism is summarized in Fig. 4f where the photoexcitation of Cu@f-gC_3_N_4_ generated electron-hole pairs; the electrons (e^-^) at the conduction band edge migrate to O_2_ to form a superoxide O_2_•⁻ (**IV**) radical ion: the simultaneous hydrogen abstraction from the tertiary amine (**I**) and the hole migration from the photocatalyst to the substrate leads to the formation of an iminium ion intermediate (**III**) and O_2_H^-^ species (**V**). The hydrolysis of the iminium ion intermediate (**III**) ultimately yielded the secondary amine as the final product. All intermediates and byproducts, such as O_2_•⁻, H_2_O_2_, and HCHO, were experimentally detected, further strengthening the mechanistic conclusions in this manuscript.

In conclusion, this work establishes a mild and selective photocatalytic protocol for the *N*-demethylation of tertiary amines using a highly atomically dispersed Cu@f-gC_3_N_4_ photocatalyst under 390 nm light irradiation and aerobic conditions. Under 390 nm irradiation, Cu@f-gC_3_N_4_ enables the conversion of diverse aliphatic, aromatic, and pharmaceutically relevant tertiary amines into secondary amines under mild conditions. And the reaction can be performed on a 10 mmol scale, demonstrating the operational robustness of the system. Thorough characterization of the photocatalyst was carried out, and the heterogeneous photocatalyst demonstrated broad applicability in terms of sustainability due to its recyclability ( > 10 cycles). The significance of this work extends beyond replacing a homogeneous photocatalyst with a heterogeneous material. In contrast to noble-metal photoredox systems, the present platform uses earth-abundant Cu, allows catalyst recovery and reuse, and operates with molecular oxygen as the terminal oxidant. Moreover, the heterogeneous Cu@f-gC_3_N_4_ environment enables productive *N*-demethylation without sacrificial nucleophilic trapping additives such as nitromethane. This feature is synthetically important because the resulting secondary amine products can be directly engaged in downstream functionalization, as demonstrated by one-pot benzylation, allylation, and propargylation reactions. Thus, the process delivers not only demethylated products but also a practical entry to late-stage amine diversification. Mechanistic studies further show that the atomically dispersed Cu sites and the functionalized carbon nitride matrix jointly contribute to the reaction by facilitating charge separation, O_2_ activation, radical formation, iminium generation, and hydrolysis. HAADF-STEM, ssNMR, XANES/EXAFS, XPS, and EPR confirm the isolated Cu environment, while radical trapping, ROS detection, isotope labeling, kinetic analysis, in situ FTIR, and DFT calculations support the proposed radical/iminium pathway. Therefore, this study establishes Cu@f-gC_3_N_4_ as a structurally defined and mechanistically interrogable heterogeneous single-atom photocatalyst for sustainable *N*-dealkylation chemistry, providing a complementary platform to existing homogeneous photoredox methods.

## Methods

### General procedure for photocatalytic *N*-demethylation

A dried microwave tube equipped with a magnetic stirring bar was charged with Cu@f-gC_3_N_4_ (5 mg) and Cesium pivalate (0.2 mmol, 1.0 equiv.). The tube was then sealed with a cap and evacuated using Schlenk techniques and flushed with O_2_ gas three times. Under Oxygen gas flow, amine starting material (if liquid, otherwise added before flushing cycle) (0.2 mmol, 1.0 equiv.) and EtOH (2.0 mL) were added using a syringe flushed with inert gas. The resulting mixture was stirred for 16 hours under irradiation with 40 W KESSIL® lamps emitting light at 390 nm. After the reaction time, the reaction mixture was filtered. To determine the NMR yield of the *N*-demethylation product through ^1^H NMR, mesitylene (13.9 µL) was added as the internal standard. The purification was performed via flash column chromatography (20%–40% ethyl acetate in hexane) or TLC plate chromatography (20%–40% ethyl acetate in hexane).

## Supplementary information

Supplementary Information

Transparent Peer Review file

## Data Availability

The authors declare that all data supporting the findings of this study are available within the article and its Supplementary Information files. Extra data is available from the corresponding author upon request. Supplementary tables and figures, experimental procedures, characterization, and spectra of all materials are included in the Supplementary Information.
