# Supplementary material for: An Atomically Dispersed Copper-Catalyst Lights the Way to Selective N-Demethylations
Source: Nat Commun. 2026 Aug 19;17:8489. doi: 10.1038/s41467-026-76282-0 (PMC13490395; doi:10.1038/s41467-026-76282-0)
Supplement: Supplementary file 1 [file 41467_2026_76282_MOESM1_ESM.pdf]

Supplementary Information for

An Atomically Dispersed Copper-Catalyst Lights the Way to Selective *N*-Demethylations

Wenhao Su,[1†] Suman Pradhan,[1†] Jun Hu,[1] Peng Ren,[2] Nils Rockstroh,[3] Alina Skorynina,[4] Piotr Kuśtrowski,[5] Susanna Monti,[6] Giovanni Barcaro,[7] Chaoqin Zeng,[8,9] Jabor Rabeah,[3,8] Aleksander Jaworski,[10] Laura Simonelli,[4] Adam Slabon,[11]* Shoubhik Das[1]*

[1]Department of Chemistry, University of Bayreuth; Universitätsstraße 30, 95447 Bayreuth, Germany.

[2]College of Chemical Engineering and Technology, Taiyuan University of Science and Technology, Taiyuan 030024, China.

[3]Leibniz-Institut für Katalyse e.V. (LIKAT Rostock), Albert-Einstein-Straße 29a, 18059 Rostock, Germany.

[4]CLAESS Beamline, ALBA Synchrotron Light Facility, Carrer de la Llum 2-26, Cerdanyola del Vallès, Barcelona 08290, Spain.

[5]Faculty of Chemistry, Jagiellonian University, Gronostajowa 2, 30-387 Kraków, Poland.

[6]CNR-ICCOM, Institute of Chemistry of Organometallic Compounds, Area della Ricerca, Pisa I-56124, Italy.

[7]CNR-IPCF, Institute for Chemical and Physical Processes, Area della Ricerca, Pisa I-56124, Italy.

[8]State Key Laboratory of Low Carbon Catalysis and Carbon Dioxide Utilization, Lanzhou Institute of Chemical Physics, Chinese Academy of Sciences, Lanzhou 730000, Gansu, China.

[9]University of Chinese Academy of Sciences, Beijing 100049, China.

[10]Department of Chemistry, Stockholm University, Stockholm 10691, Sweden.

[11]Chair of Inorganic Chemistry, University of Wuppertal, Gaußstraße 20, 42119 Wuppertal, Germany.

†These authors contributed equally to this work.

*Corresponding authors: slabon@uni-wuppertal.de & Shoubhik.Das@uni-bayreuth.de

Table of contents

1 General information 3

2 Experimental section 5

2.1 Synthesis of the photocatalyst 5
2.2 General procedure for synthesis of substrates 5
2.2.1 General procedure (GP1) for *N*-methylation of diarylamines[1] 5
2.2.2 General procedure (GP2) for *N*-methylation and *N*-alkylation of aliphatic amines[2] 6
2.2.3 General procedure (GP3) for reductive amination of ketones with *N*-methyl amines[3] 6
2.2.4 General procedure (GP4) for reductive amination sequence[4] 7
2.3 General procedure (GP5) for photocatalytic *N*-demethylation 7
2.4 Optimization of the reaction conditions 8
2.5 Recyclability experiments 8
2.6 Gram-scale reaction 9
2.7 Synthetic applications 10
2.8 Comparison of representative *N*-demethylation methods 11
2.9 Mechanistic investigations 11
2.9.1 Kinetics of the reaction 11
2.9.2 On-Off experiment 12
2.9.3 Initial rate KIE for *N*-demethylation 12
2.9.4 Hydrogen peroxide detection experiments[5] 13
2.9.5 Superoxide radical anion quenching experiment 14
2.9.6 Apparent quantum yield 14
2.9.7 Radical quenching experiments 15
2.9.8 FTIR experiments 15
2.9.9 Formaldehyde detection 16
2.9.10 Deuterium labelling experiments 16

3 Characterization of the Cu@f-gC_3N_4 catalyst 18

3.1 UV–Visible DRS spectroscopy 18
3.2 The Mott-Schottky (MS) measurements 18
3.3 Inductively coupled plasma optical emission spectroscopy (ICP-OES) 19
3.4 Solid-state nuclear magnetic resonance (ssNMR) 20
3.5 Scanning transmission electron microscopy (STEM) 20
3.6 X-ray photoelectron spectroscopy (XPS) 21
3.7 XANES and EXAFS 22
3.8 EPR experiments 23

4 Computational details 24

4.1 Preliminary model building and reactive molecular dynamics (RMD) simulations 24
4.2 Quantum chemistry calculations 28

5 Characterization of products 29

6 NMR spectra 45

7 Supplementary references 88

1 General information

Materials: Chemicals and solvents were obtained from commercial suppliers (Sigma Aldrich, TCI, Thermo Fisher Scientific, J&K Scientific, Fluorochem and ABCR) and used without further purification. Non-commercial starting materials were prepared as described below according to literature procedures. All reactions were carried out under Nitrogen atmosphere using Schlenk techniques. Thin Layer Chromatography (TLC) was performed using TLC plates from Merck (SiO_2, Kieselgel 60 F254 neutral, on aluminum with fluorescence indicator) and compounds were visualized by UV detection (254 nm). Flash column chromatographic purification of products was accomplished using an automated chromatography system with on-line UV and ELSD detection using Grace® Silica flash Cartridges.

NMR spectroscopy: Nuclear Magnetic Resonance (NMR) spectra were recorded on a Bruker Avance III 400 (101 MHz for ^{13}C) Fourier Transform NMR spectrometer at 300 K, using the non or partly deuterated solvent as internal standard (^{1}H: δ = 7.26 ppm, ^{13}C: δ = 77.16 ppm for $CDCl_3$; ^{1}H: δ = 2.50 ppm, ^{13}C: δ = 39.52 ppm for DMSO-d_6; ^{1}H: δ = 3.31 ppm, ^{13}C: δ = 49.00 ppm for MeOD). Chemical shifts (δ) are given in ppm and coupling constants (*J*) are reported in Hertz (Hz). Multiplicities are described as s (singlet), d (doublet), t (triplet), q (quartet), p (quintet), br s (broad singlet) and m (multiplet) or combinations thereof. ^{13}C NMR spectra were recorded with complete proton decoupling. ^{1}H NMR yields were determined by addition of a known amount of an internal standard and dissolving everything in a suitable deuterated solvent, followed by ^{1}H NMR analysis.

GC-MS: Gas chromatography-mass spectrometry samples were prepared by dissolving 0.1-5 mg of the compound in acetone (GC-MS quality) and further diluted to a concentration of 10^{-5}–10^{-6} M. The samples were subsequently filtered using a CHROMAFIL® PET-20/25 syringe filter and 3 µL were injected. The apparatus was an Agilent Technologies 7890 A GC System coupled to an Agilent Technologies 5975 C inert MSD with triple-axis detector. As column an Optima 725820.30 30 m × 250 µm × 0.25 µm was selected. Carrier gas was helium. Inlet temperature heater: 225 °C. Oven program: 70 °C for 3 min, then heating 5 °C min^{-1} to 160 °C and heating at 160 °C for 2 min. A one-minute post-run at 280 °C ends the oven program.

HRMS: High resolution mass spectrometry samples were prepared by dissolving 0.1–5 mg of the compound in DMSO or CH_3CN/H_2O and further diluted to a concentration of 10^{-5}–10^{-6} M. Formic acid (0.1%) was added prior to injection. 10 µL of each sample was injected using the CapLC system (Waters) and electrosprayed using a standard electrospray source. Samples were

injected with an interval of 3 minutes. Positive ion mode accurate mass spectra were acquired using a Q-TOF II instrument (Waters). The MS was calibrated prior to use with a 0.1% H_3PO_4 solution. The spectra were locking mass corrected using the known mass of the nearest H_3PO_4 cluster or a known background ion. Analytes were detected as protonated or as a sodium adduct. All measured masses are within a difference of 5 ppm compared to the calculated mass unless specified otherwise.

Photocatalysis setup: Four 40 W KESSIL® lamps equipped with a switch to regulate the intensity of illumination were secured with clamps and positioned to face a rack where the reaction tubes were placed. An electric fan was positioned in front of the rack to prevent overheating from LED light irradiation. The reaction tubes were capped with septa. The reaction mixture was agitated with a magnetic stirring plate set at a speed between 800 to 1000 rpm. To prevent contamination from other light sources, the fume hood was covered with aluminium foil.

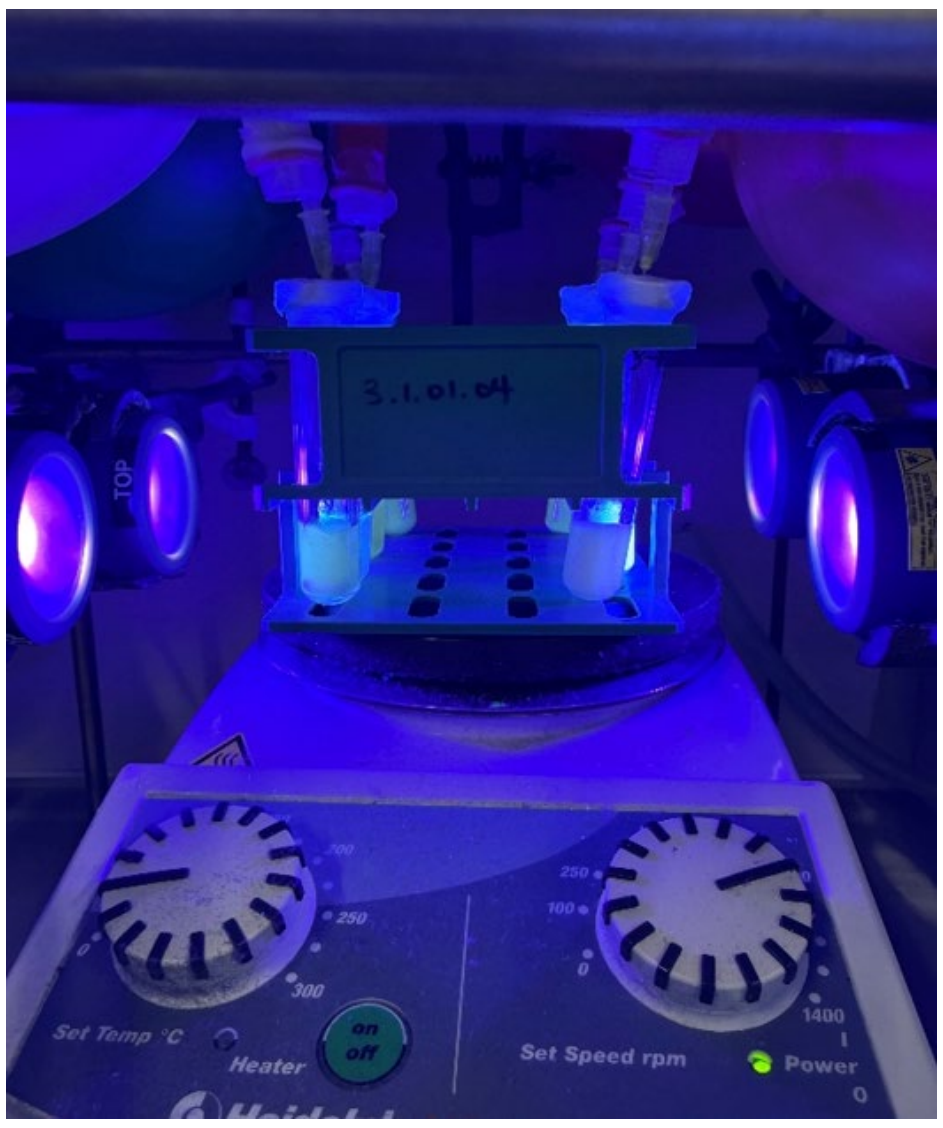

Supplementary Fig. 1. Photocatalysis setup used in this work.

2 Experimental section

2.1 Synthesis of the photocatalyst

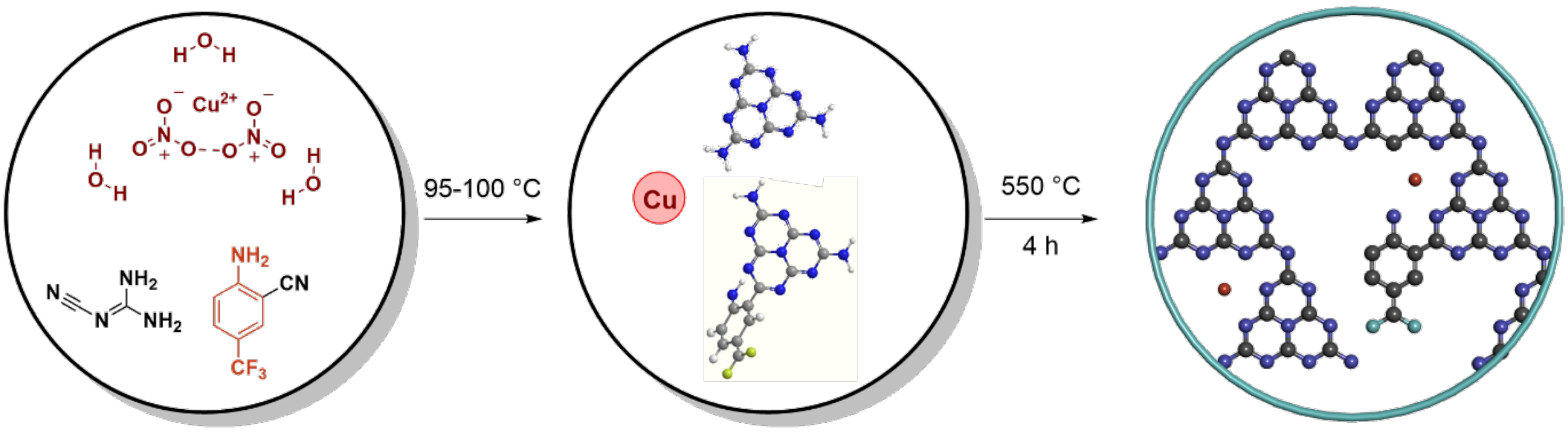

The atomically dispersed heterogeneous photocatalyst, Cu@f-gC_3N_4 was prepared through a two-step process by involving successive impregnation and calcination. In a typical synthesis, dicyandiamide (DCDA, 9 g, 107 mmol) and 2-amino-5-(trifluoromethyl) benzonitrile (0.150 g, 1.6 wt%, 0.8 mmol) were homogeneously mixed with $Cu(NO_3)_2$ •$3H_2O$ (0.019 g, 0.2 wt%, 0.08 mmol). The mixture was then stirred and heated in water (45 mL) at 95–100 °C until it was completely dried. The resulting mixture was removed, grounded in an agate mortar, and loaded onto a stainless-steel chamber. Subsequently, the chamber was heated to 550 °C in a GERO carbolite tube furnace for 244 min under aerobic atmosphere. The temperature was maintained for 4 h followed by cooling the chamber to room temperature in 6 h.

2.2 General procedure for synthesis of substrates

2.2.1 General procedure (GP1) for *N*-methylation of diarylamines[1]

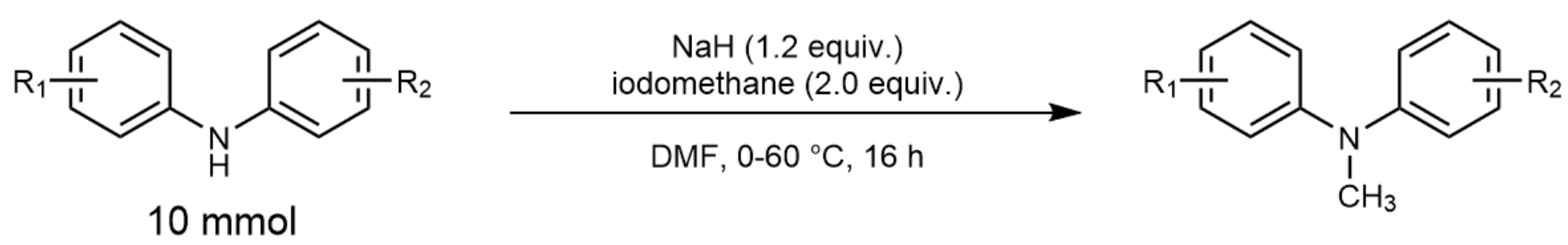

A sealed tube equipped with a magnetic stirrer bar was charged with diarylamine (10 mmol, 1.0 equiv.) and DMF (3 mL/mmol), then sealed with a rubber septum under a nitrogen atmosphere. The mixture was cooled in an ice bath, and NaH (60% in mineral oil, 1.2 equiv.) was added. Afterward, the ice bath was removed, and the resulting mixture was stirred at room temperature for 30 minutes. The mixture was then cooled again in the ice bath, and iodomethane (2.0 equiv.) was added. The ice bath was removed, and the reaction mixture was stirred at room temperature for 1 hour under a nitrogen atmosphere. Subsequently, the sealed tube was heated and stirred at 60 °C for 16 hours. After cooling the reaction mixture to 0 °C, 100 mL water and 30 mL EtOAc were added. The organic layer was separated, washed

thoroughly with 30 mL brine (5–6 times), dried over Na_2SO_4, filtered, and concentrated under reduced pressure. The crude product was then purified by column chromatography on silica gel using hexane/ethyl acetate (10:1 v/v) as eluent to provide the desired products.

2.2.2 General procedure (GP2) for *N*-methylation and *N*-alkylation of aliphatic amines[2]

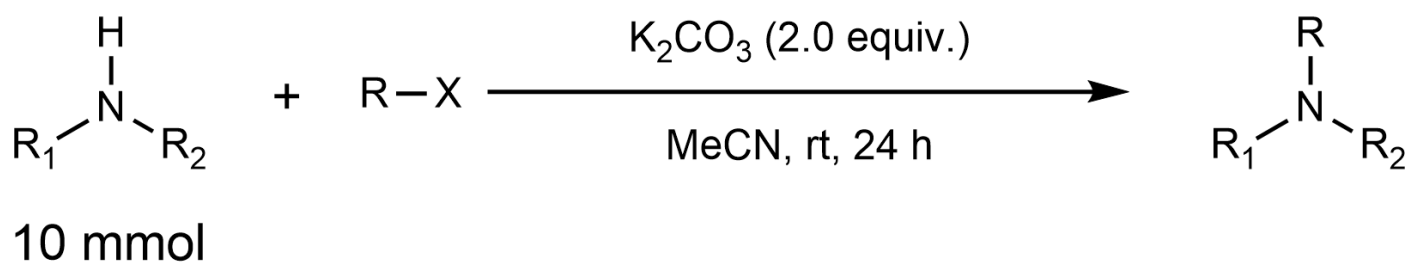

A sealed tube equipped with a magnetic stirrer bar was charged with the amine (10 mmol, 1.0 equiv.), K_2CO_3 (1.8 equiv.), and anhydrous MeCN (10.0 mL/mmol of amine) were combined. The alkylating agent (e.g., iodomethane or alkyl halide, 0.9 equiv.) was then added. The reaction mixture was stirred at room temperature for 24 hours. After completion, the reaction was concentrated under reduced pressure, and the residue was dissolved in 30 mL CH_2Cl_2. The organic solution was washed with 30 mL 1 M aqueous NaOH. The aqueous layer was extracted with CH_2Cl_2, and the combined organic layers were washed successively with brine and water. The organic layers were then dried over anhydrous Na_2SO_4, filtered, and concentrated under reduced pressure. The crude product was then purified by column chromatography on silica gel using hexane/ethyl acetate (10:1 v/v) as eluent to provide the desired products.

2.2.3 General procedure (GP3) for reductive amination of ketones with *N*-methyl amines[3]

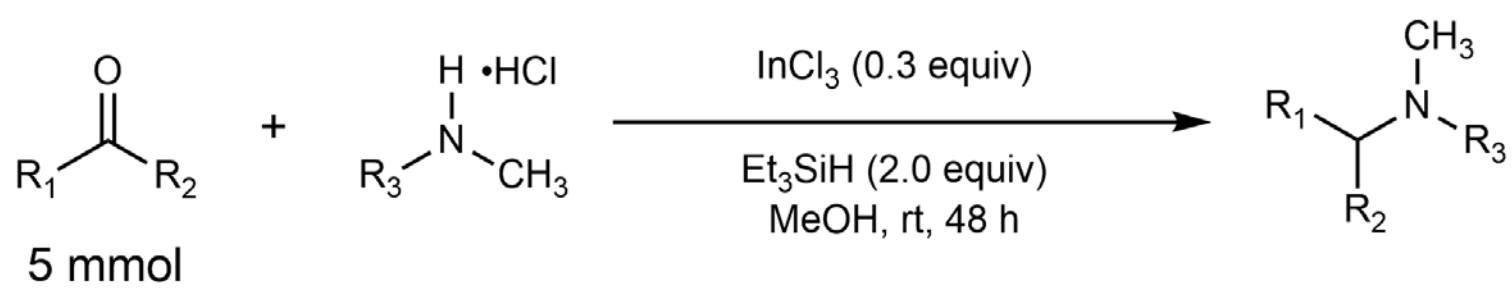

To a stirred solution of the carbonyl substrate (5.0 mmol, 1.0 equiv.) in methanol (20.0 mL) at room temperature, the corresponding amine-HCl salt (5.0 mmol) was added. The mixture was stirred for 1 hour. Et_3SiH (1.5 mL, 10.0 mmol) was then added dropwise via syringe, followed by $InCl_3$ (330 mg, 1.5 mmol). The reaction was allowed to stir at room temperature and was monitored by TLC (hexane/ethyl acetate (5:1 v/v)). Upon completion, the reaction was quenched with 10 mL saturated K_2CO_3 solution. The mixture was extracted with ethyl acetate. The combined organic layers were washed with brine, dried over anhydrous Na_2SO_4, filtered, and concentrated under reduced pressure. The crude product was then purified by column

chromatography on silica gel using hexane/ethyl acetate (10:1 v/v) as eluent to provide the desired products.

2.2.4 General procedure (GP4) for reductive amination sequence[4]

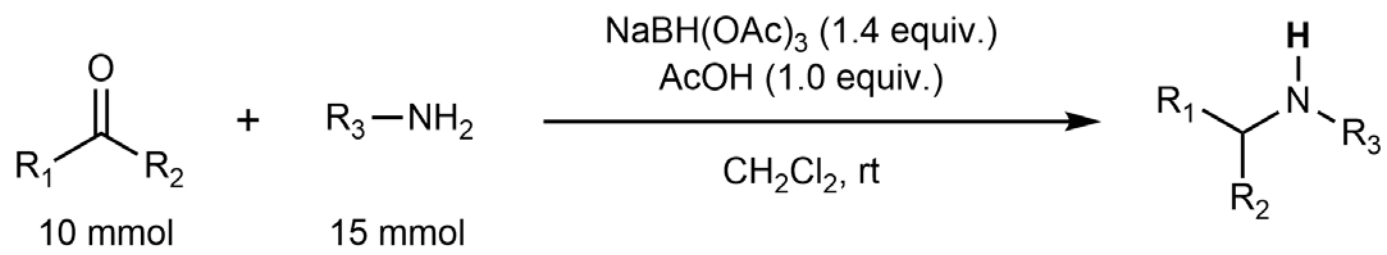

To a stirred solution of the ketone (10 mmol, 1.0 equiv.) and the amine (15 mmol, 1.5 equiv.) in DCM (0.33 M) were added sodium triacetoxyborohydride (1.4 equiv.) and AcOH (1.0 equiv.). Upon reaction completion (24–48 h), 30 mL 1 N aqueous NaOH was added, and the product was extracted with 30 mL Et_2O. The combined organic layers were dried upon Na_2SO_4 and then concentrated in vacuo. The crude residue was directly used in the next step.

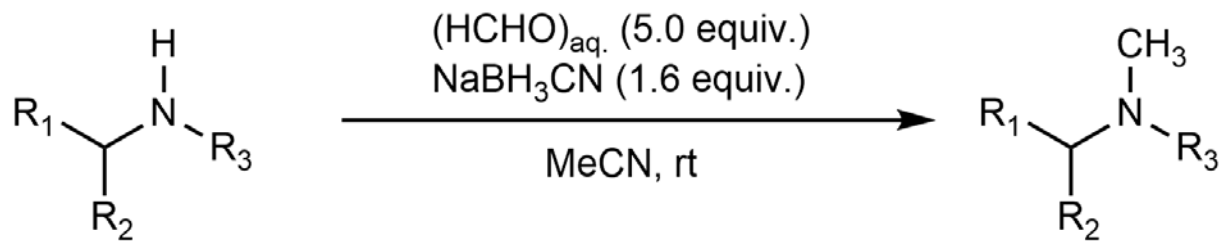

To a stirred solution of the amine (10 mmol, 1.5 equiv.) and formaldehyde (37% in water, 5.0 equiv.) in MeCN (1 M) were added sodium cyanoborohydride (1.6 equiv.). Upon reaction completion, 20 mL 0.1 M aqueous NaOH was added, and the reaction mixture was extracted with Et_2O. The combined organic layers were washed with brine, dried over anhydrous Na_2SO_4, filtered, and concentrated under reduced pressure. The crude product was then purified by column chromatography on silica gel using hexane/ethyl acetate (10:1 v/v) as eluent to provide the desired products.

2.3 General procedure (GP5) for photocatalytic *N*-demethylation

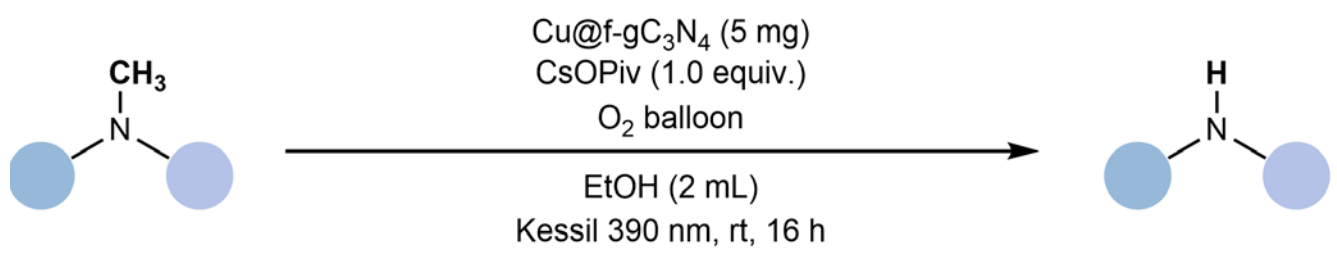

A dried microwave tube equipped with a magnetic stirring bar was charged with Cu@f-gC_3N_4 (5 mg) and Cesium pivalate (0.2 mmol, 1.0 equiv.). The tube was then sealed with a cap and evacuated using Schlenk techniques and flushed with O_2 gas three times. Under Oxygen gas flow, amine starting material (if liquid, otherwise added before flushing cycle) (0.2 mmol, 1.0 equiv.) and EtOH (2.0 mL) were added using a syringe flushed with inert gas. The resulting mixture was stirred for 16 hours under irradiation with 40 W KESSIL® lamps emitting light at 390 nm. After the reaction time, the reaction mixture was filtered. To determine the NMR yield of the *N*-demethylation product through ^{1}H NMR, mesitylene (13.9 µL) was added as the

internal standard. The purification was performed via flash column chromatography (20%–40% ethyl acetate in hexane) or TLC plate chromatography (20%–40% ethyl acetate in hexane).

2.4 Optimization of the reaction conditions

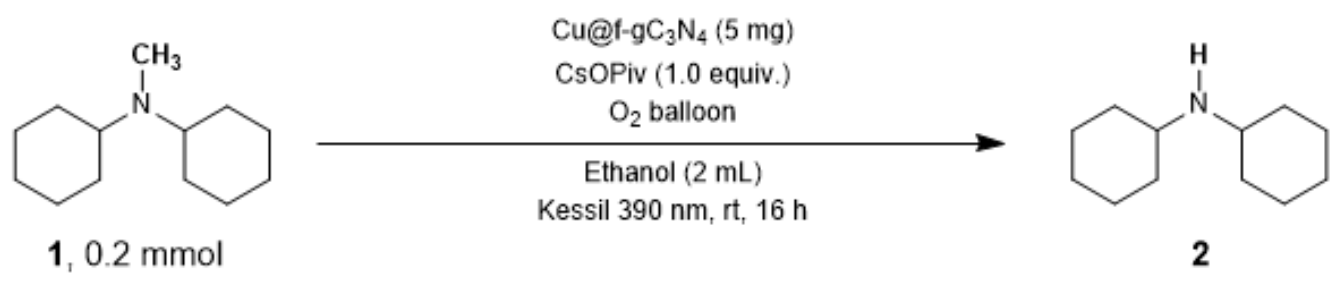

Entry	Variation	Yield (%) [a]	Entry	Variation	Yield (%) [a]
1	none	89	19	MeCN, $K_2S_2O_8$	trace
2	MeCN	50	20	Co@f-gC_3N_4	75
3	TFE	20	21	Ni@f-gC_3N_4	50
4	THF	44	22	Cu@f3-gC_3N_4	58
5	MeOH	62	23	Cu@f5-gC_3N_4	63
6	HFIP	trace	24	Cu@f24-gC_3N_4	69
7	DCE	trace	25	f-gC_3N_4	30
8	NaOPiv	40	26	gC_3N_4	23
9	KOPiv	49	27	$Cu_{0.1wt\%}$@f-gC_3N_4	58
10	CsOPiv (2.0 eq)	66	28	$Cu_{0.4wt\%}$@f-gC_3N_4	87
11	CsOPiv (0.5 eq)	86	29	no catalyst	trace
12	MeCN, K_2HPO_4	55	30	no base	21
13	MeCN, Cs_2CO_3	42	31	no O_2	NR
14	MeCN, Na_2CO_3	30	32	no light	NR
15	MeCN, DBU	39	33	Kessil 427 nm	46
16	MeCN, TFA	53	34	Kessil 456 nm	15
17	MeCN, 30% H_2O_2	trace	35	CdS	20
18	MeCN, *t*-BuOOH	NR	36	TiO_2	9

[a] ^{1}H NMR yield using mesitylene as internal standard

Supplementary Table 1. Optimization of the reaction conditions for *N*-demethylation.

2.5 Recyclability experiments

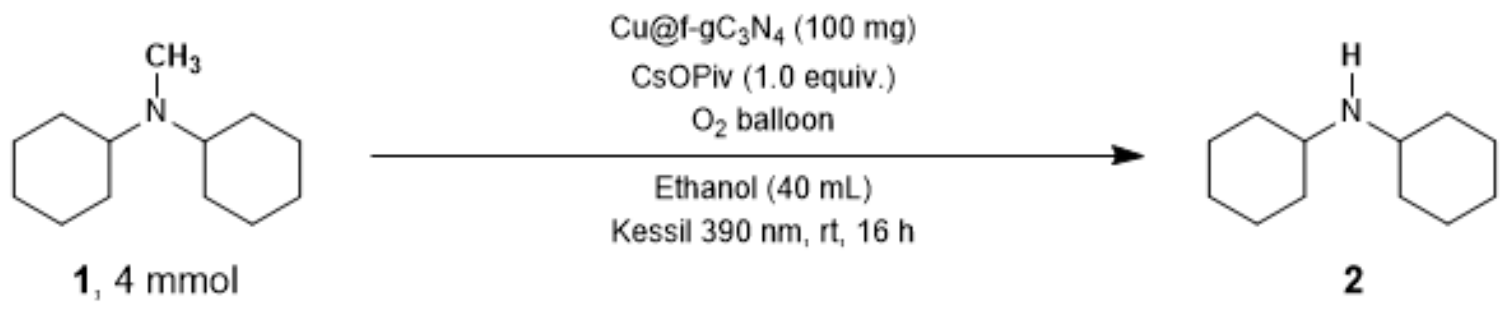

To assess the stability of the catalyst, a photocatalytic *N*-demethylation of *N,N*-dicyclohexylmethylamine was up-scaled and used for a recyclability test. To ensure the stability, the recycled catalyst was used for at least 9 consecutive experiments under identical reaction conditions. After completing the photocatalytic *N*-demethylation using fresh Cu@f-gC_3N_4 catalyst according to **GP5**, the reaction mixture was transferred into a centrifuge tube and a

small portion of it was taken to determine the NMR yield. Meanwhile, an equivalent weight of water was added to another centrifuge tube to balance the weight. Both centrifuge tubes were centrifuged at 4000 r/s for 12 minutes, resulting in the formation of a deposit of the catalyst. Afterwards, the catalyst in the centrifuge tube was washed with acetone and water three times, respectively. At last, the catalyst was dried in the oven for 48 hours at 60 °C before reuse in subsequent reactions.

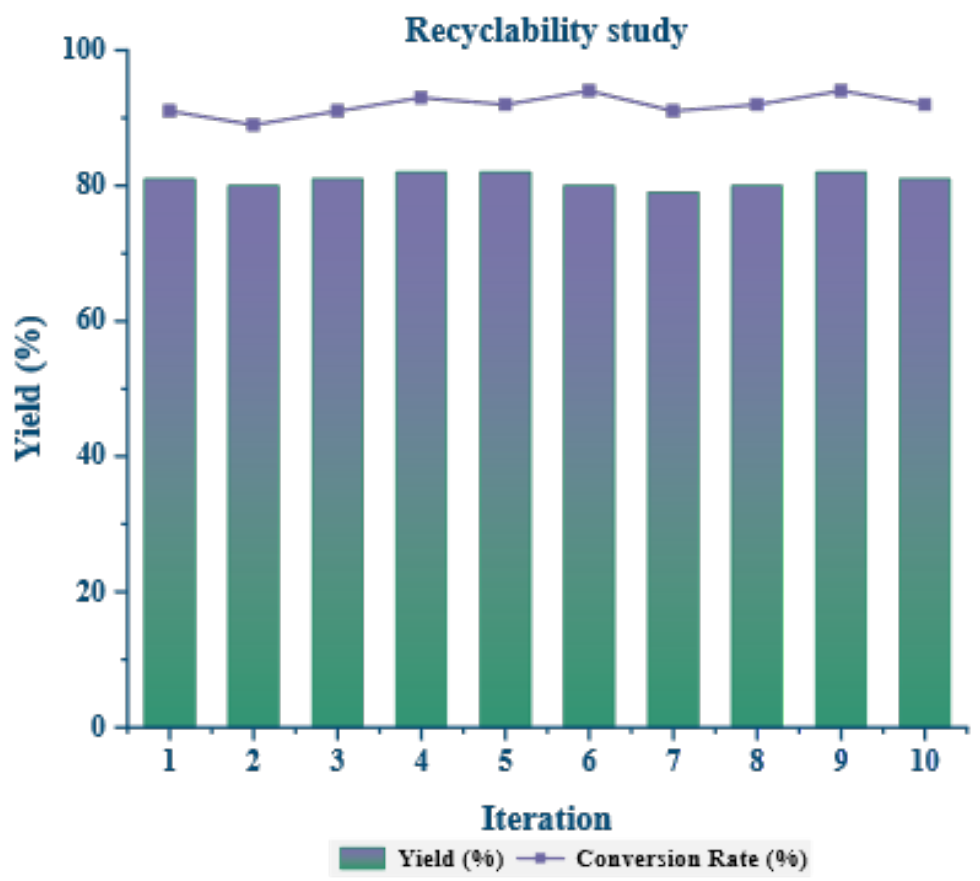

Iteration	Yield (%)	Conversion rate (%)
1	81	91
2	80	89
3	81	91
4	82	93
5	82	92
6	80	94
7	79	91
8	80	92
9	82	94
10	81	92

Supplementary Fig. 2. Recycle experiments of the *N*-demethylation reaction using *N,N*-dicyclohexylmethylamine.

2.6 Gram-scale reaction

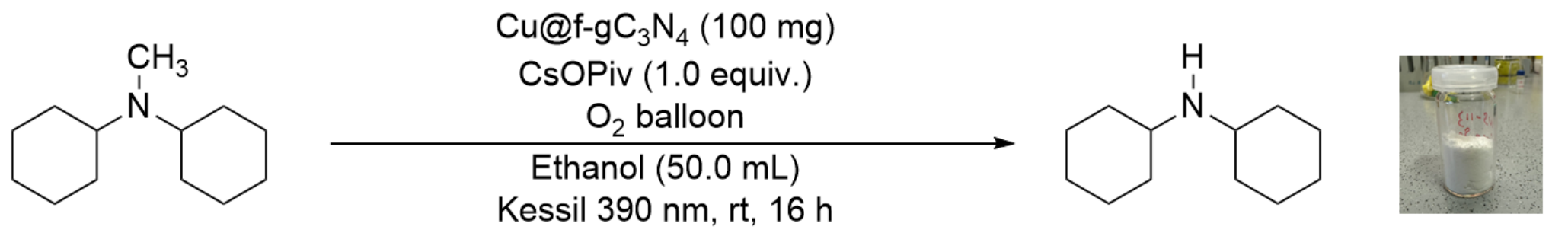

1, 1.95 g, 10 mmol **2,** 81% NMR yield 1.65 g, 71%

The gram-scale reaction was conducted in a 100 mL Schlenk tube. The tube along with a magnetic stirring bar was charged with Cu@f-gC_3N_4 (100 mg) and Cesium pivalate (10 mmol, 1.0 equiv.). The reaction mixture was purged with oxygen gas three times. Under Oxygen gas flow, *N,N*-Dicyclohexylmethylamine (10 mmol, 1.0 equiv.) and dry EtOH (50.0 mL) were added using a syringe flushed with inert gas. The tube was then sealed and subjected to photocatalysis at room temperature for 16 h with a fan cooling. The catalyst was collected from the reaction mixture, and the reaction mixture was analysed by ^{1}H NMR to confirm the product yield. The reaction mixture was concentrated to remove the solvent and diluted with dichloromethane. It was then washed with water and dried over anhydrous Na_2SO_4. After removing the solvent under vacuum, the residue was treated with 50 mL 1 M HCl in methanol. The solvent was

evaporated, and the resulting solid was washed twice with 20 mL of ethyl acetate. Finally, the product was dried to obtain the HCl salt.

2.7 Synthetic applications

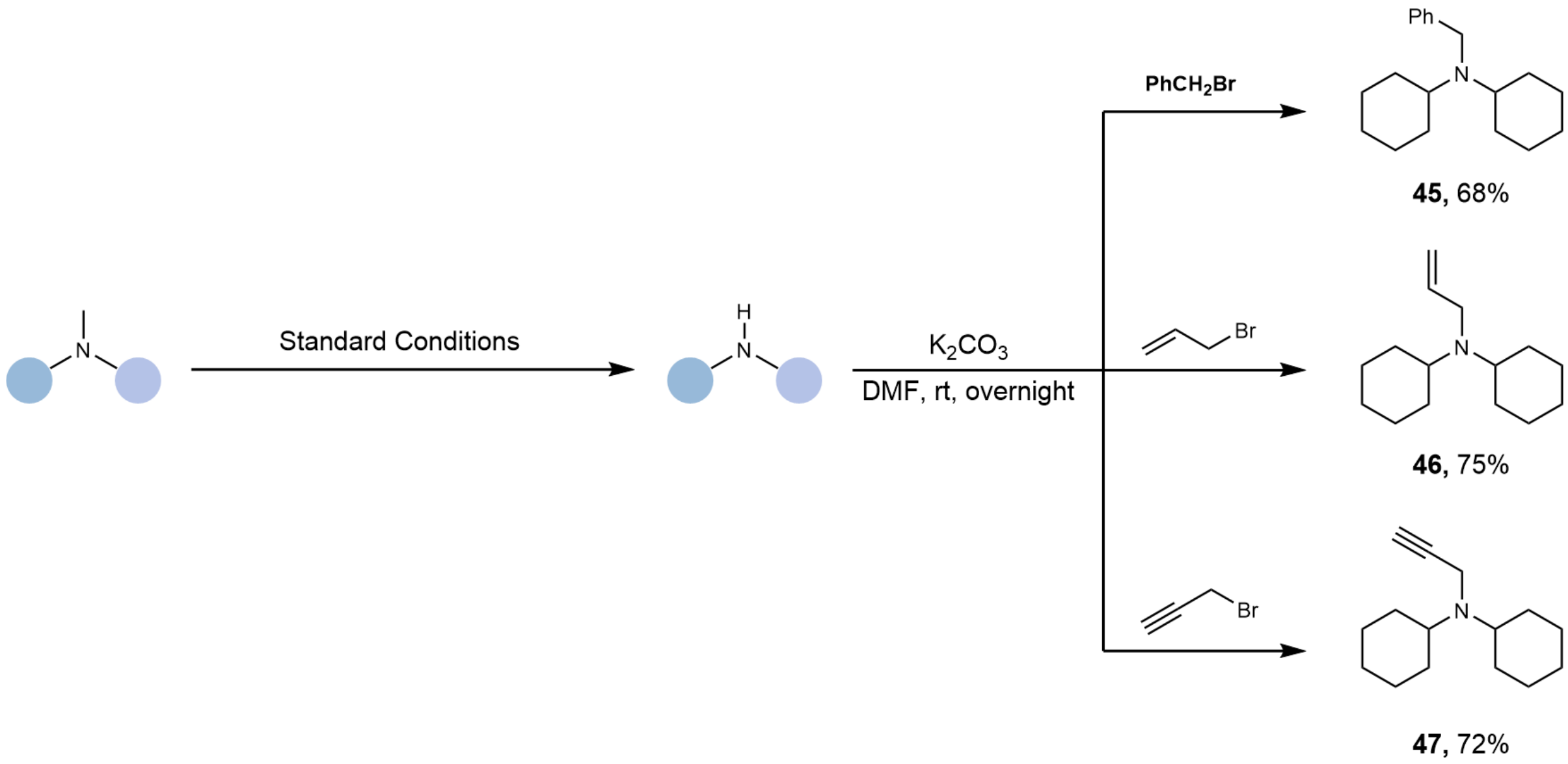

A dried microwave tube equipped with a magnetic stirring bar was charged with Cu@f-gC_3N_4 (5 mg) and Cesium pivalate (0.2 mmol, 1.0 equiv.). The tube was sealed with a cap, evacuated using Schlenk techniques, and flushed with O_2 gas three times. Under a continuous flow of oxygen, the amine starting material (0.2 mmol, 1.0 equiv.; if liquid, added after O_2 flushing) and ethanol (2.0 mL) were introduced via a syringe pre-flushed with inert gas. The mixture was irradiated with a 40 W KESSIL® lamp (λ = 390 nm) and stirred for 16 h at room temperature.

Upon completion, the reaction mixture was filtered through a 0.2 µm PTFE syringe filter to remove the catalyst and insoluble solids. The filtrate was concentrated under reduced pressure. The resulting residue was redissolved in dry DMF (2.0 mL), followed by the addition of K_2CO_3 (0.4 mmol, 2.0 equiv.) and the desired alkyl bromide (0.3 mmol, 1.5 equiv.). The mixture was stirred at room temperature overnight.

After completion, the reaction was quenched with water, extracted with ethyl acetate, and the combined organic layers were dried over anhydrous Na_2SO_4, filtered, and concentrated. The crude product was purified by flash column chromatography using hexane/ethyl acetate (10:1 v/v) as eluent to afford the corresponding *N*-alkylated product **45-47**.

2.8 Comparison of representative *N*-demethylation methods

Entry	Method / Reference	Catalyst Type	Oxidant / Redox Partner	Stoichiometric Additives	Light Source	Recyclability	Substrate Scope	Key Features / Limitations
1	**This work** ($Cu@f\text{-}gC_3N_4$)	Heterogeneous Cu photocatalyst with high atomic dispersion	O_2 (air)	None	390 nm LED (visible light)	Yes (>10 cycles demonstrated)	Aliphatic and aromatic tertiary amines; late-stage functionalization of complex molecules	Mild, aerobic conditions; no stoichiometric HAT reagent; recyclable catalyst; scalable (10 mmol); formaldehyde as benign byproduct
2	Rovis *et al.* JACS 2023	Homogeneous Ni/photoredox system	Redox-neutral (photoredox)	Stoichiometric aryl bromides (HAT reagent)	Visible light	No	Broad scope of trialkylamines	Elegant and selective; requires stoichiometric halide reagent; homogeneous, non-recyclable catalyst
3	Yilmaz & Emmert, J. Org. Chem. 2023	Homogeneous photoredox catalyst	O_2	Sacrificial additives	Visible light	No	Mainly tertiary amines	Mild conditions; limited recyclability; additive-dependent
4	Peroxidase-mediated N-demethylation	Enzymatic	H_2O_2 / O_2	Enzyme preparation	None	Limited	Selected alkaloids	High selectivity; narrow pH range; limited substrate scope
5	Polonovski / von Braun reactions	Stoichiometric chemical reagents	Peracids / BrCN	Stoichiometric oxidants	None	No	Limited functional-group tolerance	Harsh conditions; toxic reagents; poor selectivity

Supplementary Table 2. Comparison of representative *N*-demethylation methods.

2.9 Mechanistic investigations

2.9.1 Kinetics of the reaction

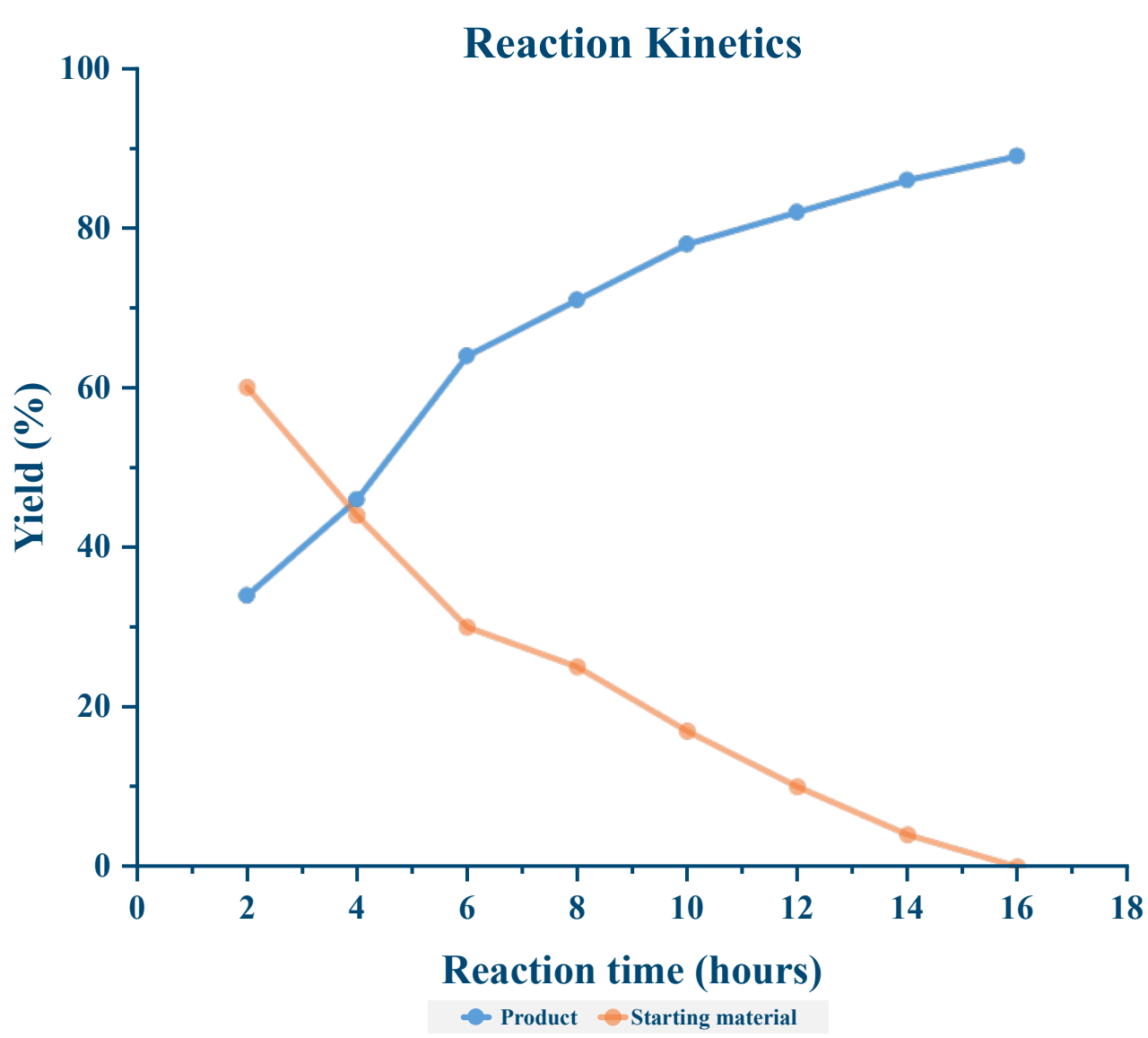

Supplementary Fig. 3. Kinetics of the *N*-demethylation reaction.

Reaction time (hours)	2	4	6	8	10	12	14	16
Yield (%)	34	46	64	71	78	82	85	89
Starting material (%)	60	47	32	23	16	13	9	0

Supplementary Table 3. Kinetics of the *N*-demethylation reaction.

2.9.2 On-Off experiment

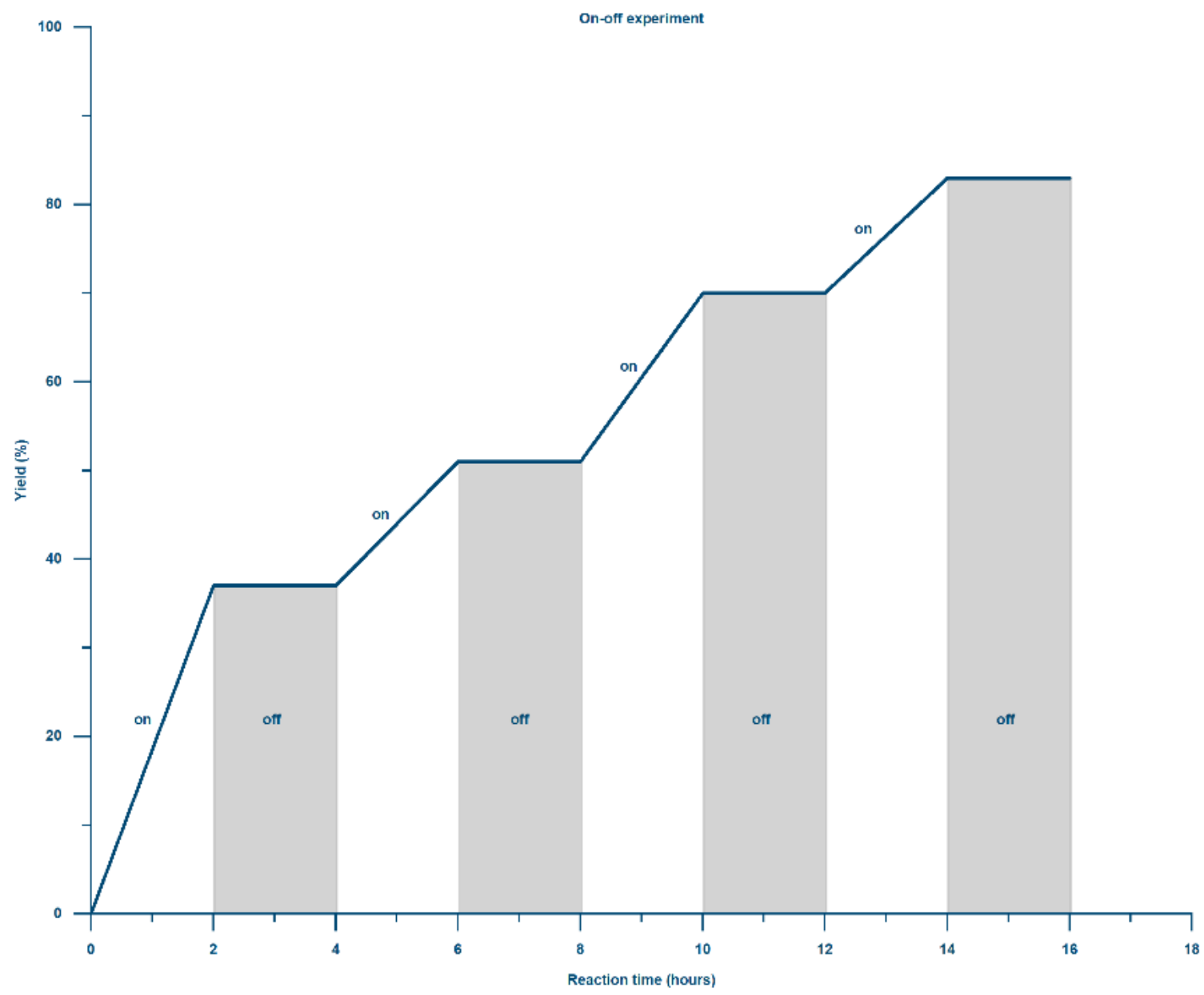

Supplementary Fig. 4. On-Off experiment.

Light on/off		on	off	on	off	on	off	on	off
Reaction time (hours)	0	2	4	6	8	10	12	14	16
Yield (%)	0	37	37	51	51	70	70	83	83

Supplementary Table 4. On-Off experiment.

2.9.3 Initial rate KIE for *N*-demethylation

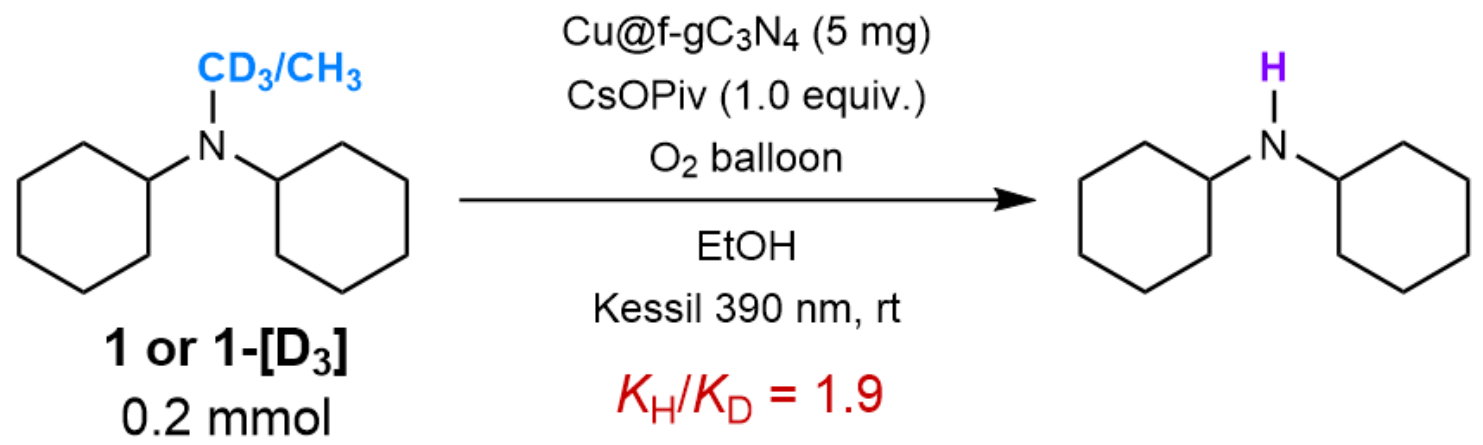

General procedure: A dried microwave tube equipped with a magnetic stirring bar was charged with Cu@f-gC_3N_4 (5 mg) and cesium pivalate (0.2 mmol, 1.0 equiv.). The tube was then sealed with a cap and evacuated using Schlenk techniques and flushed with O_2 gas three times. Under Oxygen gas flow, **1 or 1-[D₃]** (0.2 mmol, 1.0 equiv.) and dry EtOH (2.0 mL) were added using a syringe flushed with inert gas. The resulting mixture was stirred for 16 hours under irradiation with 40 W KESSIL® lamps emitting light at 390 nm. After the reaction time, the reaction mixture was filtered. To determine the NMR yield of the *N*-demethylation product through ^{1}H NMR, mesitylene (13.9 µL) was added as the internal standard.

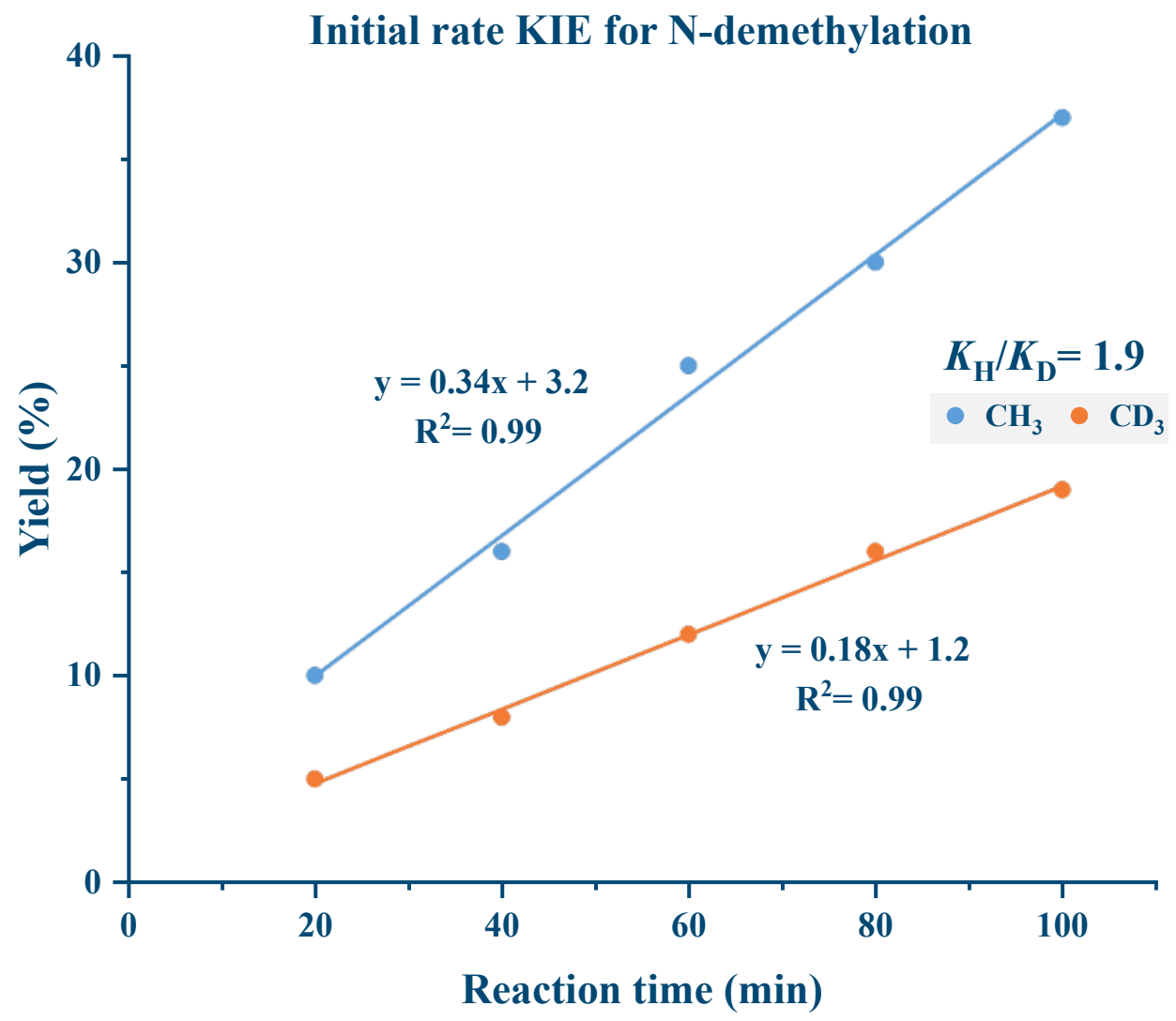

Supplementary Fig. 5. Initial rate KIE for *N*-demethylation.

Reaction time (mins)	20	40	60	80	100
Yield of 2a (%)	10	16	25	30	37
Yield of 2a-[D_3] (%)	5	8	12	16	19

Supplementary Table 5. Initial rate KIE for *N*-demethylation.

2.9.4 Hydrogen peroxide detection experiments[5]

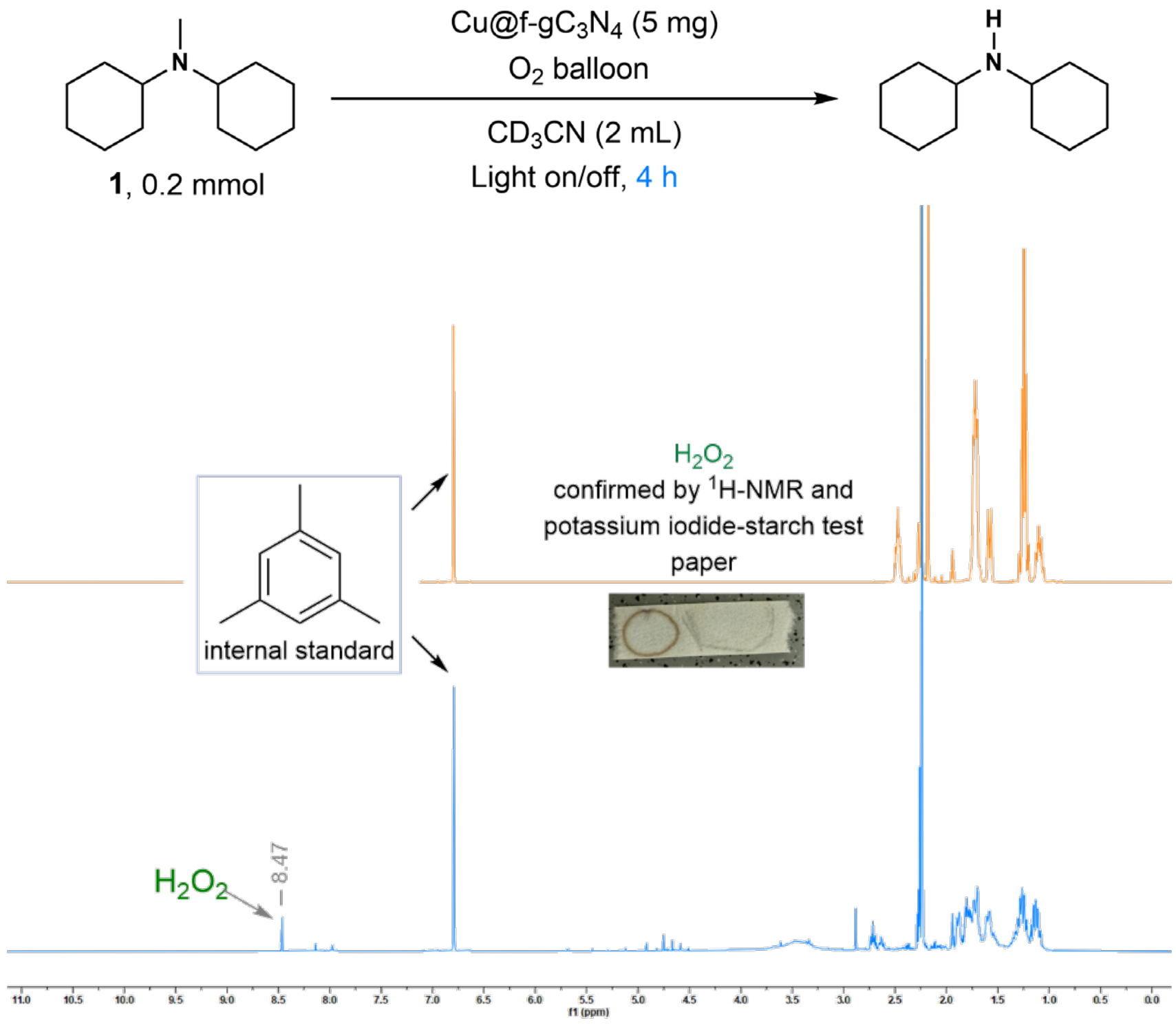

Supplementary Fig. 6. Evidence for hydrogen peroxide formation.

A dried microwave tube equipped with a magnetic stirring bar was charged with Cu@f-gC_3N_4 (5 mg). The tube was then sealed with a cap and evacuated using Schlenk techniques and flushed with O_2 gas three times. Under Oxygen gas flow, **1** (0.2 mmol, 1.0 equiv.) and dry CD_3CN (2.0 mL) were added using a syringe flushed with inert gas. The resulting mixture was stirred for 4 hours under irradiation with 40 W KESSIL® lamps emitting light at 390 nm. A parallel reaction was performed under identical conditions except in the dark, achieved by shielding the reaction vessel from light using aluminum foil. Upon completion of the reaction, the mixture was filtered directly into an NMR tube to remove the solid catalyst. Mesitylene (13.9 µL) was added as the internal standard, and the yield of the *N*-demethylation product was determined by ^1H NMR spectroscopy.

2.9.5 Superoxide radical anion quenching experiment

A dried microwave tube equipped with a magnetic stirring bar was charged with Cu@f-gC_3N_4 (5 mg), Cesium pivalate (0.2 mmol, 1.0 equiv.) and 1,4-Benzoquinone (2.0 equiv.). The tube was then sealed with a cap and evacuated using Schlenk techniques and flushed with O_2 gas three times. Under Oxygen gas flow, **1** (0.2 mmol, 1.0 equiv.) and dry EtOH (2.0 mL) were added using a syringe flushed with inert gas. The resulting mixture was stirred for 4 hours under irradiation with 40 W KESSIL® lamps emitting light at 390 nm. A parallel experiment was conducted under the same conditions, except without the addition of 1,4-benzoquinone, to assess its influence on the reaction outcome.

2.9.6 Apparent quantum yield

The amount of formed product (mol): M = 0.2 mmol x 89% = 0.178 mmol = 1.78 x 10^{-4} mol

The light intensity of Kessil LED lamp (λ = 390 nm, 40W) was 0.06 W/cm^2.

$$\eta = \frac{N_e}{N_p} \times 100\% = \frac{\frac{2 \times M \times N_A}{S \times P \times t}}{h \times \frac{c}{\lambda}} \times 100\% = \frac{2 \times M \times N_A \times h \times c}{S \times P \times t \times \lambda} \times 100\%$$

$$= \frac{2 * 1.78 \times 10^{-4} * 6.022 \times 10^{23} \times 6.63 \times 10^{-34} \times 3 \times 10^{8}}{1.5 \times \pi \times 0.06 \times 16 \times 3600 \times 390 \times 10^{-9}} \times 100\%$$

$$= 0{,}7\%$$

Where, M represents the amount of formed product (mol), N_A is the Avogadro constant (6.022 x 10^{23} mol^{-1}), h is the Planck constant (6.63x10^{-34} J·s), c is the light speed (3x10^8 $m \cdot s^{-1}$), S is the irradiation area (1,5π cm^2), P is the light intensity (0.06 $W \cdot cm^{-2}$), t is irradiation time (16 x 3600 s), λ is the wavelength of light (m).

2.9.7 Radical quenching experiments

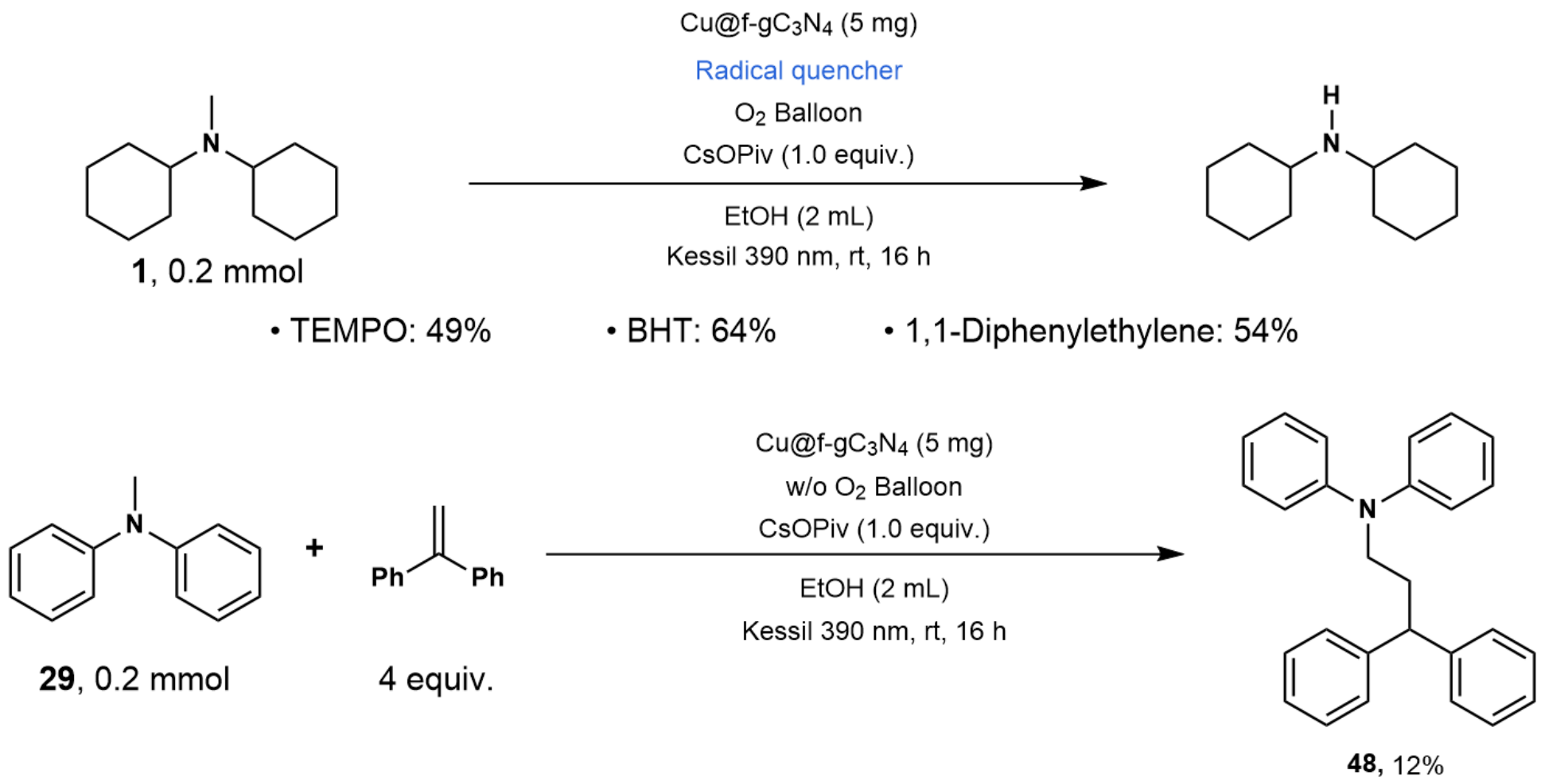

A dried microwave tube equipped with a magnetic stirring bar was charged with Cu@f-gC_3N_4 (5 mg), Cesium pivalate (0.2 mmol, 1.0 equiv.) and radical quencher (4.0 equiv., unless the quencher was a liquid, in which case it was added after the O_2 flushing step). The tube was then sealed with a cap and evacuated using Schlenk techniques and flushed with O_2 gas three times. Under Oxygen gas flow, **1** (0.2 mmol, 1.0 equiv.) and dry EtOH (2.0 mL) were added using a syringe flushed with inert gas. The resulting mixture was stirred for 16 hours under irradiation with 40 W KESSIL® lamps emitting light at 390 nm.

As the quenching experiments showed a moderate decrease in product yield but did not completely suppress the reaction, further studies were conducted to gain mechanistic insight. Specifically, a reaction using *N*-methyldiphenylamine as the substrate and BHT (4.0 equiv.) as the radical scavenger was performed under identical conditions but in the absence of O_2. Under these modified conditions, compound **48** was successfully detected.

***N*-(3,3-diphenylpropyl)-*N*-phenylaniline (48):**

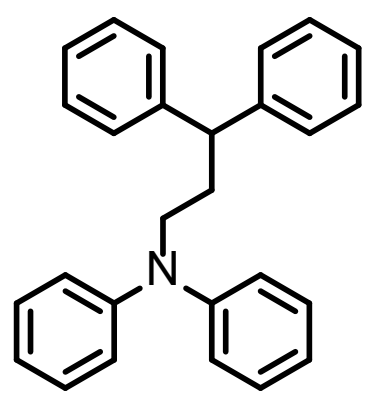

Formula: $C_{27}H_{25}N$

48, 12%

^{1}H NMR (500 MHz, CDCl₃) δ 7.27 (m, 4H), 7.22 (m, 8H), 7.20 – 7.16 (m, 2H), 6.95 – 6.87 (m, 6H), 3.95 (t, *J* = 7.8 Hz, 1H), 3.69 – 3.62 (m, 2H), 2.47 – 2.40 (m, 2H).

^{13}C NMR (126 MHz, $CDCl_3$) δ 147.95, 144.48, 129.38, 128.68, 127.88, 126.49, 121.28, 120.98, 50.87, 49.12, 33.21.

HRMS (ESI): $[M+H]^+$ calcd. 364.2065, found 364.2072.

2.9.8 FTIR experiments

Cu@f-gC_3N_4 catalyst with *N,N*-dicyclohexylmethylamine was prepared using the standard KBr pellet method. Approximately 1 mg of the dried sample was thoroughly mixed with about

200 mg of dried spectroscopic-grade potassium bromide (KBr) and finely ground in an agate mortar. The mixture was then pressed into a transparent pellet under a certain pressure. Then measured in the instrument (Bruker Invenio). Signals were recorded with light irradiation.

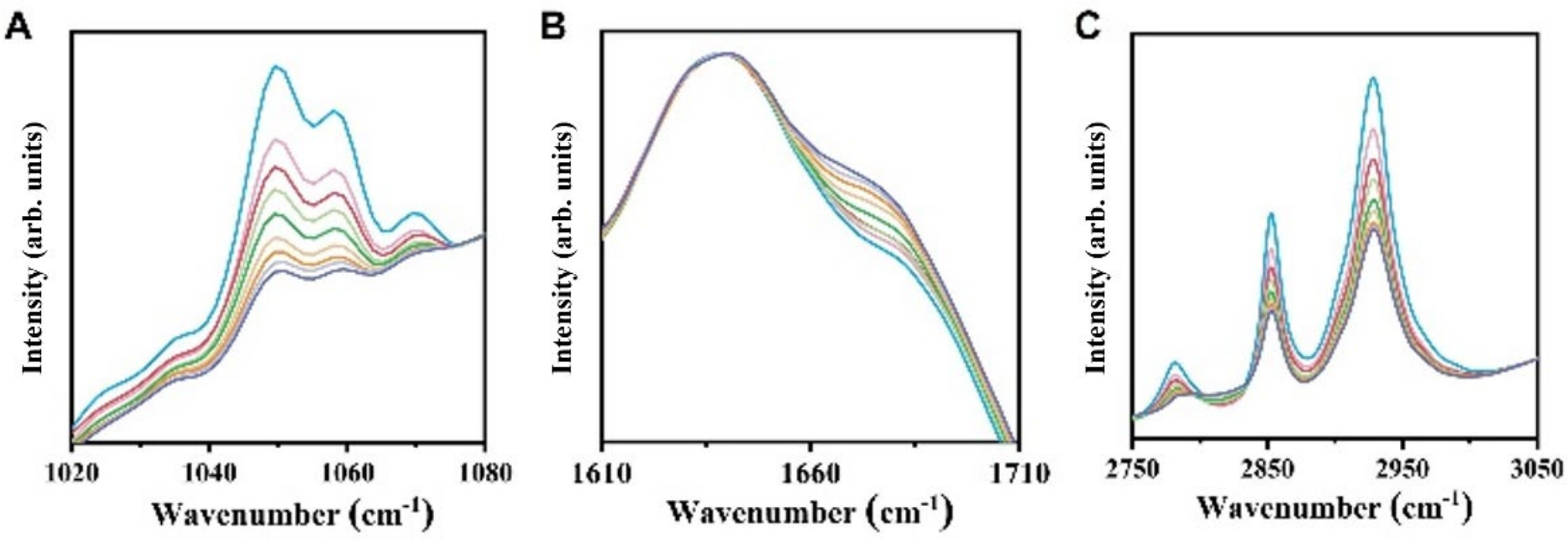

Supplementary Fig. 7: FTIR spectra of Cu@f-gC_3N_4 catalyst mixed with *N*-cyclohexyl-*N*-methylcyclohexanamine at different irradiation times, showing characteristic regions; (**A**) 1020–1080 cm^{-1}; (**B**) 1610–1710 cm^{-1}; (**C**) 2750–3050 cm^{-1}.

2.9.9 Formaldehyde detection

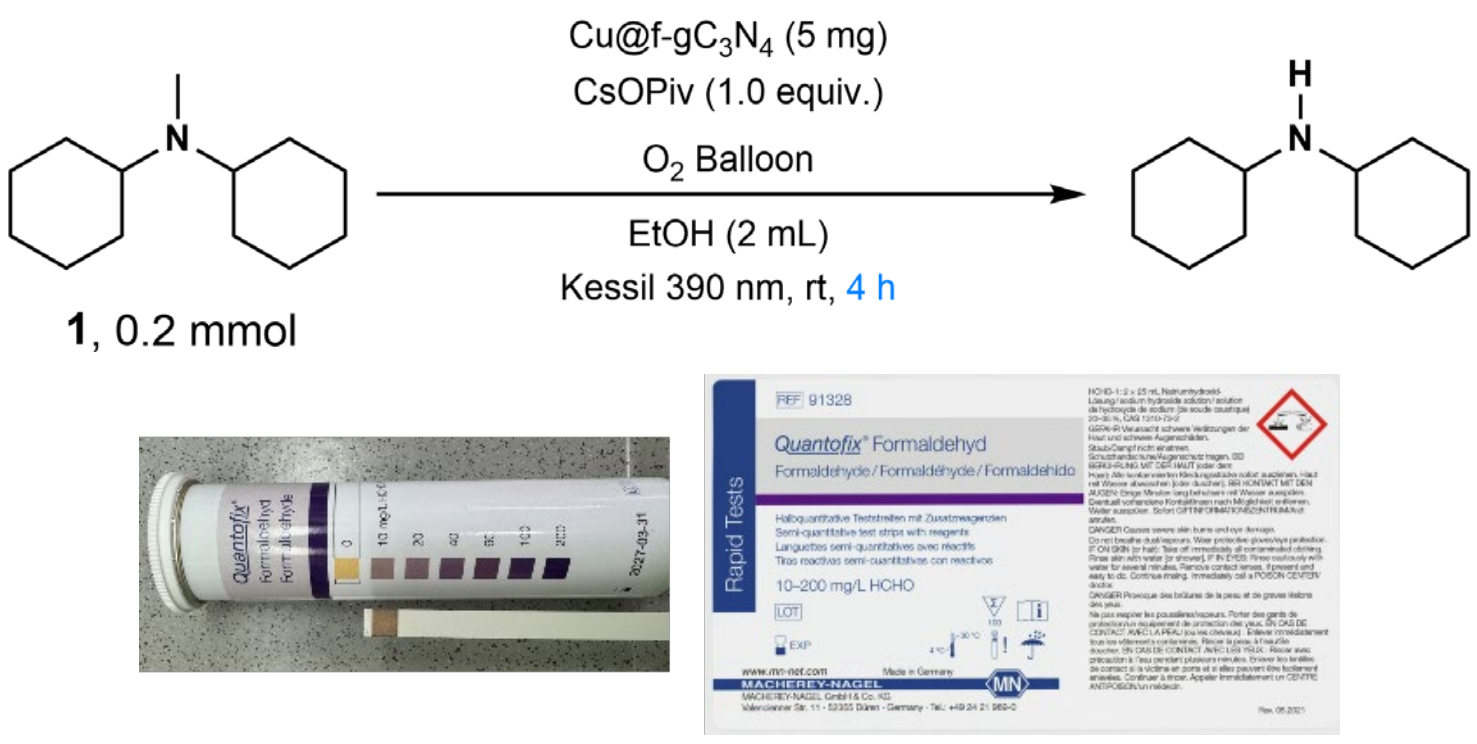

Supplementary Fig. 8. Evidence for formaldehyde formation.

A dried microwave tube equipped with a magnetic stirring bar was charged with Cu@f-gC_3N_4 (5 mg) and Cesium pivalate (0.2 mmol, 1.0 equiv.). The tube was then sealed with a cap and evacuated using Schlenk techniques and flushed with O_2 gas three times. Under Oxygen gas flow, **1** (0.2 mmol, 1.0 equiv.) and dry EtOH (2.0 mL) were added using a syringe flushed with inert gas. The resulting mixture was stirred for 4 hours under irradiation with 40 W KESSIL® lamps emitting light at 390 nm. After the reaction, the presence of formaldehyde in the mixture was assessed using semi-quantitative QUANTOFIX® Formaldehyde test strips.

2.9.10 Deuterium labelling experiments

A dried microwave reaction tube equipped with a magnetic stirring bar was charged with Cu@f-gC_3N_4 (5 mg) and CsOPiv (0.2 mmol, 1.0 equiv.). The tube was sealed with a cap, evacuated under vacuum using Schlenk techniques, and flushed with O_2 gas three times. Under

an O_2 atmosphere, substrate 1 (0.2 mmol, 1.0 equiv.; either non-deuterated or *N*-deuterated analogs) and 2.0 mL of solvent (EtOH or CD_3OD, anhydrous) were added *via* a syringe pre-flushed with inert gas. The reaction mixture was stirred at room temperature for 16 hours under irradiation with a 40 W KESSIL® lamp (λ = 390 nm). After completion, the reaction mixture was filtered through a 0.25 µm PTFE syringe filter to remove the catalyst and insoluble materials. The filtrate was directly analyzed by HRMS to determine deuterium incorporation in the products.

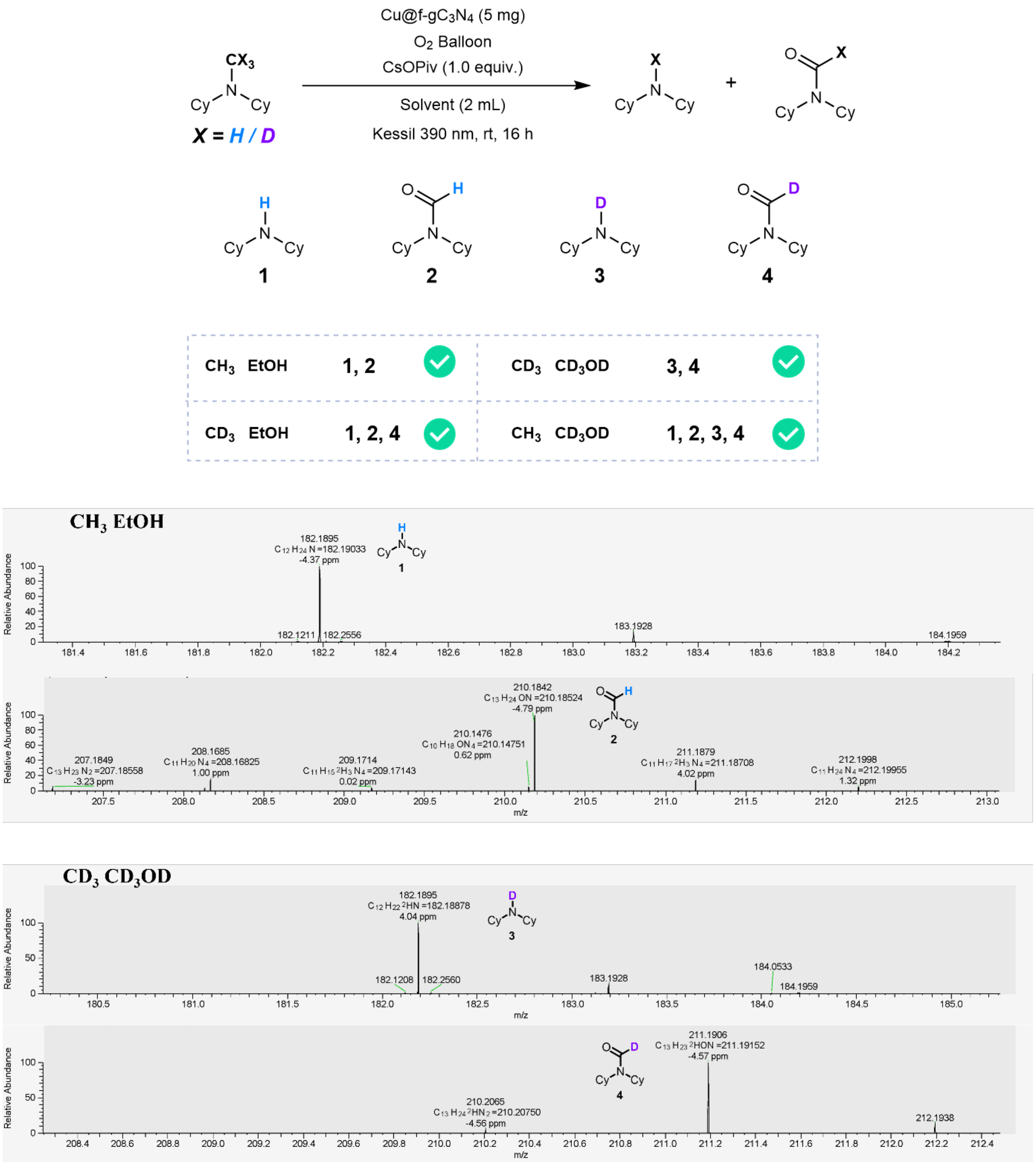

Supplementary Fig. 9. HRMS spectra.

3 Characterization of the Cu@f-gC_3N_4 catalyst

3.1 UV–Visible DRS spectroscopy

The solid UV–Vis absorption curve (**Supplementary Fig. 10a**) was obtained from diffuse reflectance spectra (DRS) measured on a Shimadzu UV-3101PC spectrophotometer. $BaSO_4$ was used as the reflectance standard. Photoluminescence experiments were conducted in solid-state on an Edinburgh FLSP920 spectrofluorometer, with a photomultiplier detector operating in the 200–900 nm wavelength range at room temperature.

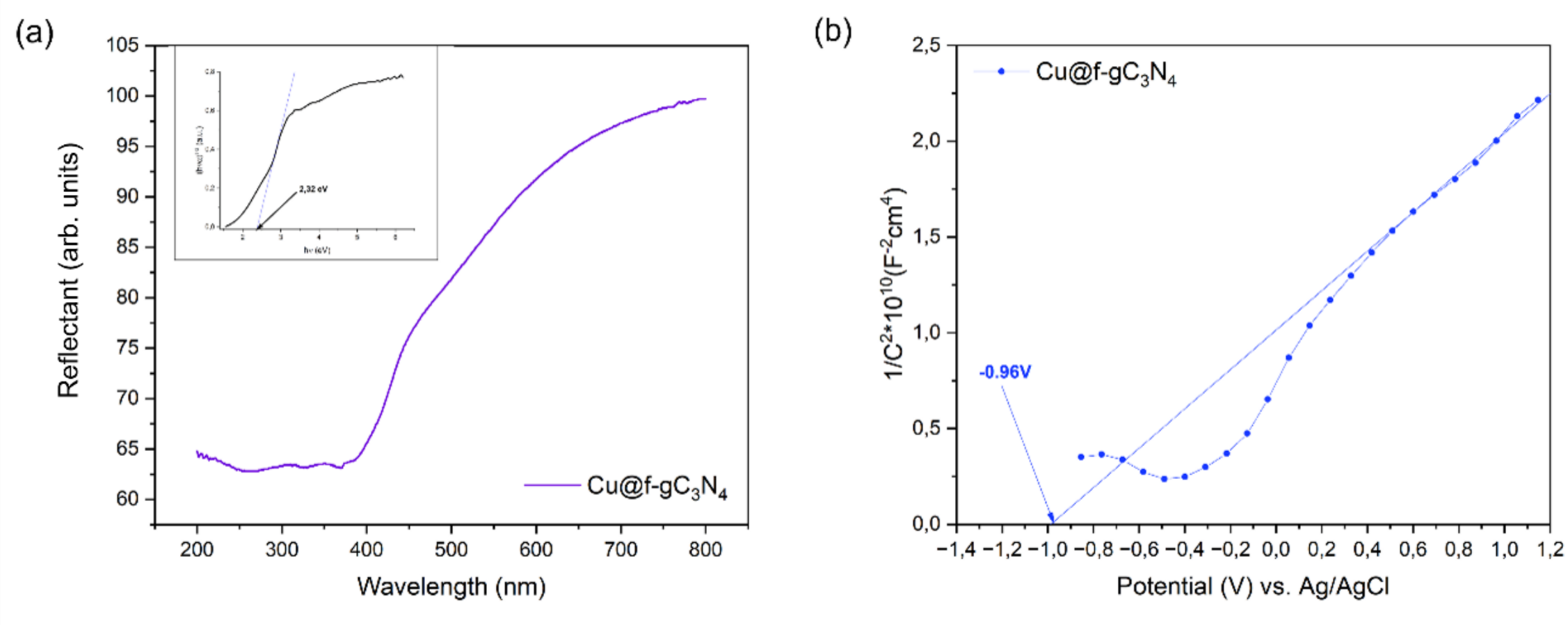

Supplementary Fig. 10. UV–Visible DRS spectroscopy. (a) UV–vis diffuse reflectance spectra, Tauc plot and (b) Mott-Schottky plot of Cu@f-gC_3N_4.

3.2 The Mott-Schottky (MS) measurements

The Mott-Schottky experiments (**Supplementary Fig. 10b**) were conducted employing a three-electrode configuration cell. The working electrode consisted of a thin film obtained through the drop-coating method, while the counter electrode comprised a Pt-foil electrode, and the reference electrode utilized an Ag/AgCl/3 M KCl electrode. The thin film electrodes were prepared by combining 5 mg of the sample with a solution composed of 50 µL ethanol and 10 µL of 5 wt% Nafion solution, followed by sonication for 1 hour. Subsequently, 15 µL of the resulting mixture was pipetted onto a FTO glass surface to create 1 × 1 cm^2 coating films, which were then dried at room temperature and further subjected to overnight drying at 40 °C in a vacuum oven. The electrolyte solution employed was 0.5 M Na_2SO_4 (with a measured pH of 7.47), degassed with N_2. Measurements were carried out using a Metrohm Autolab potentiostat-galvanostat PGSTAT204 workstation in the absence of light, with an AC amplitude of 5 mV and frequencies of 1000 Hz.

To further clarify the electronic structure, electrochemical flat-band potential measurements were conducted. Typically, flat-band potential values are derived from the Mott-Schottky equation.

$$\frac{1}{C^2} = \frac{2}{\varepsilon\varepsilon_0 N_D}\left(E - E_{fb} - \frac{K_B T}{q}\right) \quad (1)$$

Here, C represents the space charge capacitance, N_D denotes the donor density, and ε, ε_0 are the dielectric constants of the film electrode and free space, respectively. E refers to the applied potential, E_{fb} is the flat-band potential, K_B is Boltzmann's constant, T is the temperature, and q is the electronic charge. The flat-band potential E_{fb} can be obtained by extrapolating the plot to where $1/C^2 = 0$ (**Supplementary Fig. 10b**).

The positive slope of the Mott-Schottky curve suggested that the photocatalyst was n-type. Consequently, the flat band potential (E_{fb}) of Cu@f-gC_3N_4 was approximately -0.96 V (vs Ag/AgCl (3M KCl)), as determined from the intersection between the Mott-Schottky plot and the baseline. In n-type semiconductor photocatalyst, the conduction band generally lies closely to the flat-band potential, with this offset influenced by factors such as electron effective mass and carrier concentration. So, the conduction band of the Cu@f-gC_3N_4 can be calculated as -0.96V (vs. Ag/AgCl (3M KCl)). Additionally, using the band gap energy E_g, the valence band (VB) can be determined by $E_{VB}=E_{CB}+E_g$. Therefore, the valence band (VB) of Cu@f-gC_3N_4 was at ca. +1.36 V (vs. Ag/AgCl (3M KCl)). Based on: $E_{SCE}=E_{Ag/AgCl\ (3M\ KCl)} + 0.031$ V, so E_{CB} = -0.93 V (vs SCE), E_{VB}= +1.39 V (vs SCE).

3.3 Inductively coupled plasma optical emission spectroscopy (ICP-OES)

ICP-OES
ANALYSENERGEBNIS

Catalysis LIKAT Leibniz-Institut für Katalyse e.V.

FILTER: *Rockstroh, Nils* *15215* *15216*

ICP-Nr	Probe		Ergebnis Element	erw.	gef(a)	gef(b)
15215	WS Cu-FWW-23-Air be 18.12.2025	w%	Cu	-	0,206	0,193
15216	WS Cu-FWW-23-Air aft 18.12.2025	w%	Cu	-	0,187	0,187

erw.= erwarteter Wert, gef(1) / (2) = gefundener Wert (Einfach-, Doppelbestimmung) - = keine Angabe

Datu 18.12.2025

Supplementary Fig. 11. ICP-OES results.

For the detection of the Cu content inductively coupled plasma optical emission spectroscopy (ICP-OES) was applied. Therefore, the samples were dissolved in a mixture of HF and aqua regia and digested in a microwave oven (Anton Paar Multiwave 5000, Graz, Austria) at 220 °C and 50 bar. The resulting solution was then analyzed with a 715-ES ICP emission spectrometer (Varian/Agilent, Palo Alto, CA).

3.4 Solid-state nuclear magnetic resonance (ssNMR)

Magic-angle-spinning (MAS) NMR experiments were performed at a magnetic field of 14.1 T (Larmor frequencies of 600.12, 150.92, 60.83, and 564.69 MHz for ^{1}H, ^{13}C, ^{15}N, and ^{19}F respectively) on a Bruker Avance-III spectrometer. The ^{1}H and ^{19}F MAS NMR spectra were acquired using a 1.3 mm probe head and a 60 kHz MAS rate. This acquisition involved a use of a rotor-synchronized, double-adiabatic spin-echo sequence with a 90° excitation pulse of 1.25 μs (2.30 μs for ^{19}F) followed by a pair of 50.0 μs tanh/tan short high-power adiabatic pulses (SHAPs) with 5 MHz frequency sweep. All pulses operated at the nutation frequency of 200 kHz (110 kHz for ^{19}F). 64 signal transients (10240 for ^{19}F) were acquired using a relaxation delay of 5 s. Cross-polarization (CP) ^{1}H-^{13}C and ^{1}H-^{15}N CPMAS NMR spectra were recorded using a 7 mm probe head with a 7 kHz MAS rate, 65 kHz spinal and 64 proton decoupling. For ^{1}H-^{13}C CPMAS acquisition, Hartmann-Hahn matched radiofrequency fields were applied for a contact interval of 1.5 ms and 2048 signal transients were collected using a relaxation delay of 5 s. The ^{1}H-^{15}N CPMAS acquisitions involved contact interval of 5ms, and 16384 scans collected with relaxation time of 5 s. Chemical shifts are reported with respect to TMS (^{1}H, ^{13}C), nitromethane (^{15}N), and trichlorofluoromethane (^{19}F).

3.5 Scanning transmission electron microscopy (STEM)

Scanning Transmission Electron Microscopy (STEM) measurements were carried out at an operation voltage of 200 kV on a probe aberration-corrected JEM-ARM200F (JEOL, Corrector: CEOS). The microscope is equipped with a JED-2300 (JEOL) energy-dispersive X-ray (EDX) spectrometer having a silicon drift detector (dry SD60GV). High-Angle Annular Dark Field (HAADF) and Annular Bright Field (ABF) detectors were used for STEM imaging. The solid samples were deposited onto a holey carbon supported Ni grid (mesh 300) without any pre-treatment and subsequently transferred to the microscope.

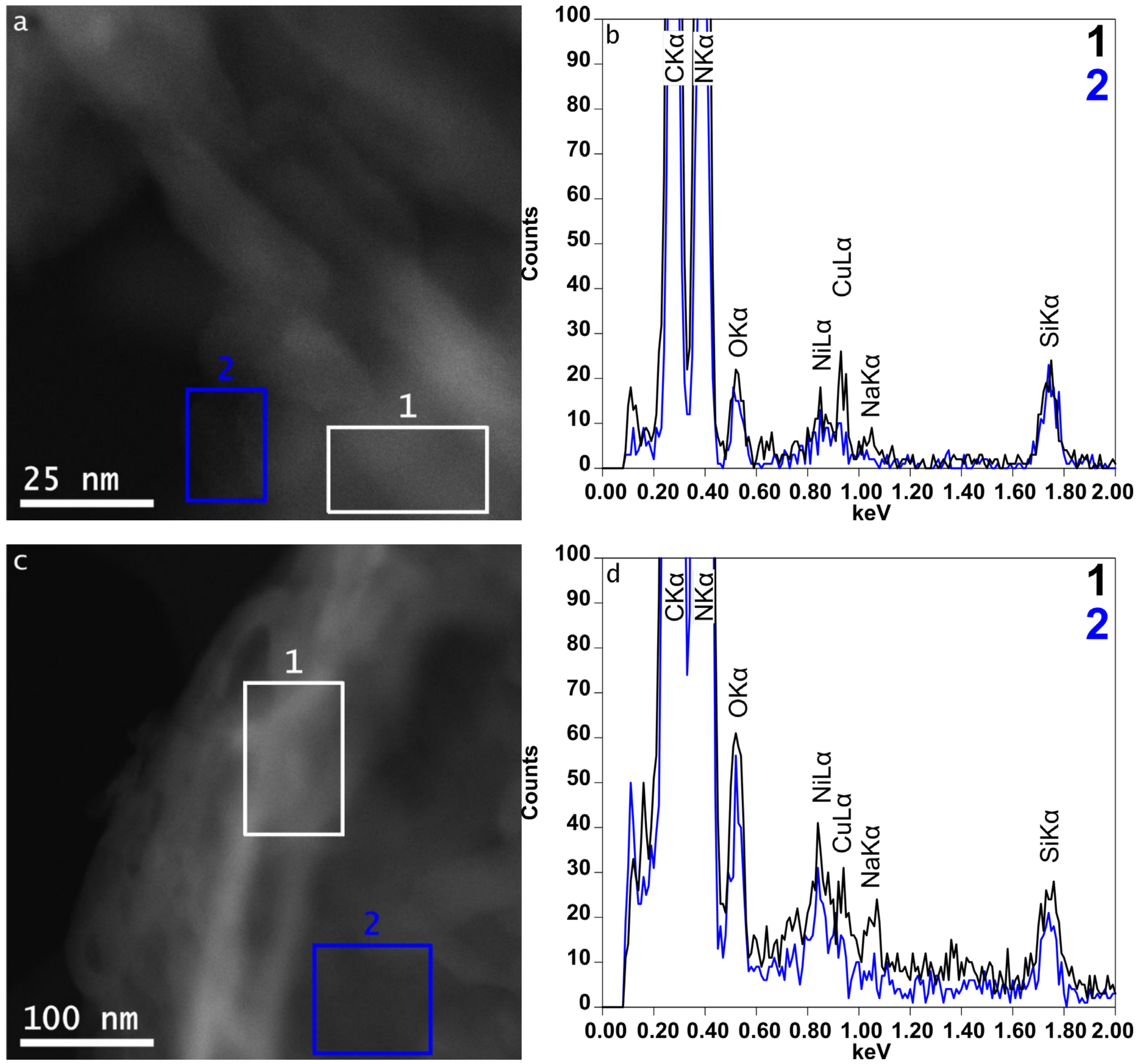

Supplementary Fig. 12. Selected EDX spectra showing the presence of Cu on the f-gC_3N_4 support (a, b) before and (c, d) after the reaction. The spectra correspond to the regions highlighted in the respective HAADF images shown in a and c. The Cu Lα signal is quite weak but not negligible, as expected for the low amounts of Cu.

3.6 X-ray photoelectron spectroscopy (XPS)

XPS spectra were collected on a Prevac spectrometer equipped with a hemispherical VG SCIENTA R3000 analyzer (pass energy of 100 eV) and a monochromatized aluminum source AlKα (1486.6 eV). Binding energies were calibrated using the C 1s line at 284.8 eV. After a Shirley background subtraction, raw spectra were fitted using Gaussian-Lorentzian peak shapes in the Casa XPS software. The surface composition of Cu@f-gC_3N_4 was studied by XPS spectra which were measured in C 1*s*, N 1*s* and Cu 2*p* regions **(Supplementary Fig. 13)**. The photoelectron emission in the C 1s region, described by the intense peak at 287.8 eV (sp^2-bonded C atoms in N-C=N), perfectly matches the emission expected for the g-C_3N_4 structure.

Additionally, the presence of both sp^2- and sp^3-hybridized carbon is observed (peak at 284.8 eV), which is attributed to contamination by adventitious carbon. The presence of the g-C_3N_4 phase is also confirmed by three XPS N 1s peaks at 398.5 eV (sp^2-hybridized N atoms in C-N=C), 400.2 eV (bridging N atoms in N-$(C)_3$) and 401.1 eV (N atoms in amino groups). A broad feature centered around 404 eV indicates π-excitation of nitrogen atoms within graphitic structures. In the Cu 2*p* region, there are no clear signals indicating the presence of significant amounts of Cu atoms on the surface.

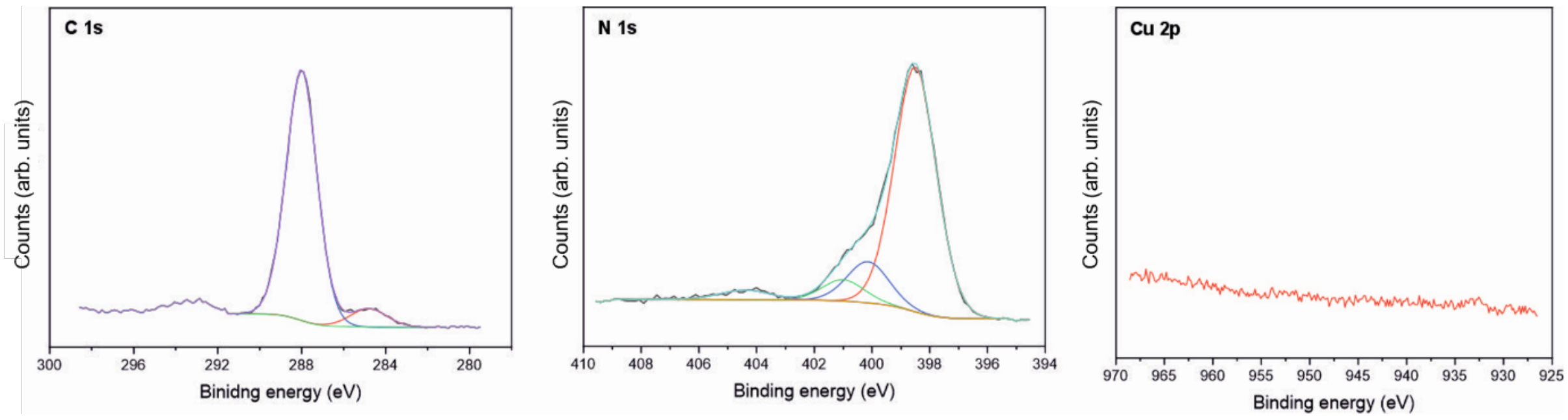

Supplementary Fig. 13: High-resolution XPS spectra of Cu@f-gC_3N_4 measured in C 1*s*, N 1*s*, and Cu 2*p* regions.

3.7 XANES and EXAFS

X-ray absorption spectra (XAS) at Cu *K*-edge were recorded at the CLÆSS[6] beamline of the ALBA synchrotron (proposal #2024108916), using a double-crystal Si(311) monochromator. The monochromator was calibrated using a Cu foil before the measurements. XAS spectra were collected at room temperature under vacuum conditions in transmission mode for the standard reference compounds (Cu foil, CuO, Cu(II) acetate and Cu_2O) and in fluorescence mode for the catalyst using 4-channel silicon drift detector (SDD) (**Supplementary Fig. 14**). The samples were mixed uniformly with cellulose and pressed in pellets.

The data were analyzed using the Athena and Artemis software of the DEMETER package. Extended X-ray absorption fine structure (EXAFS) fitting was conducted in *R*-space, after Fourier transforming the data in the *k*-range of 3–12 $Å^{-1}$ with k^1, k^2, and k^3 weightings applied simultaneously. The fitting was carried out over an *R*-range of 1.2–2.2 Å, considering Cu–N scattering path. The amplitude reduction factor (S_0^2) was fixed at 0.94, as determined from the fit of the Cu foil reference. The fitting results are shown in **Supplementary Table 6.**

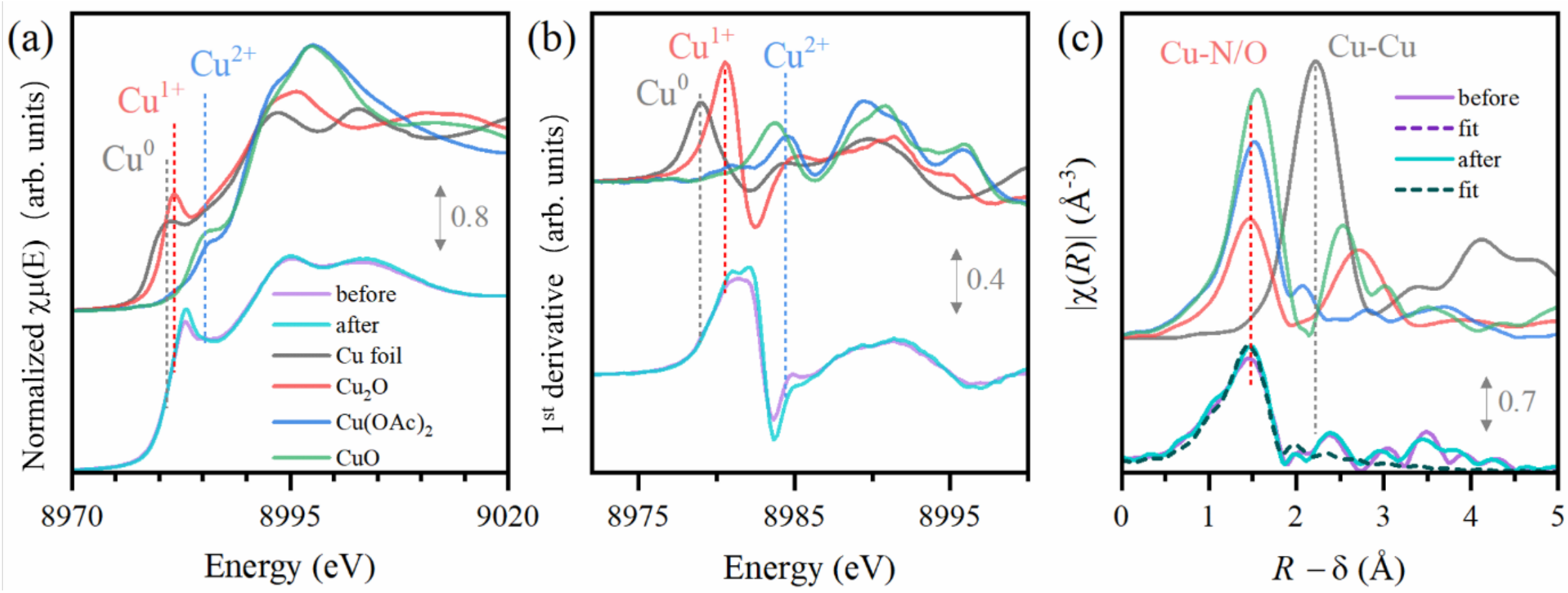

Supplementary Fig. 14. XAS spectra. (a) Normalized Cu K-edge XANES spectra, (b) first derivative of the normalized XANES spectra, and (c) FT-EXAFS spectra of Cu@f-gC_3N_4 photocatalyst, Cu foil, Cu_2O, Cu(II) acetate and CuO. FT spectrum of the Cu foil was scaled down by a factor of 2.2 to facilitate visual comparison with the catalyst spectra.

**Sample**	**Scattering path**	**CN**	**Δ*E* (eV)**	**R (Å)**	**σ^2 (Å^2)**	**R_f**
Cu@f-gC_3N_4 fresh	Cu–N	2.24 ± 0.59	0.65 ± 3.71	1.91 ± 0.06	0.007 ± 0.004	0.026
Cu@f-gC_3N_4 spent	Cu–N	2.08 ± 0.48	0.48 ± 3.35	1.91 ± 0.05	0.005 ± 0.003	0.020

Supplementary Table 6. Relative energy shift and best-fit results from the structural analysis of Cu@f-gC_3N_4 at the Cu *K*-edge. CN is the coordination number; Δ*E* is the energy shift correction; *R* is the interatomic distance and σ^2 is the Debye-Waller factor. R_f is the R-factor, which represents the relative error of the fit and data. 3.0 < k < 12; 1.2 < R < 2.2.

3.8 EPR experiments

EPR spectra of the solid sample of Cu@f-gC_3N_4 catalyst and the reaction mixture were measured with Bruker E500 spectrometer equipped with a high sensitivity resonator). For detection of $O_2^{\bullet-}$, Cu@f-gC_3N_4 catalyst in ethanol was mixed with DMPO(5,5-dimethyl-1-pyrroline N-oxide, Adamas) under stream of O_2. A capillary tube was used to draw 80 µL of the mixture, which was then placed into the EPR instrument. The samples were first tested in the dark for 1 run, followed by in situ irradiation with a Xenon lamp with cutoff filter <390 nm.

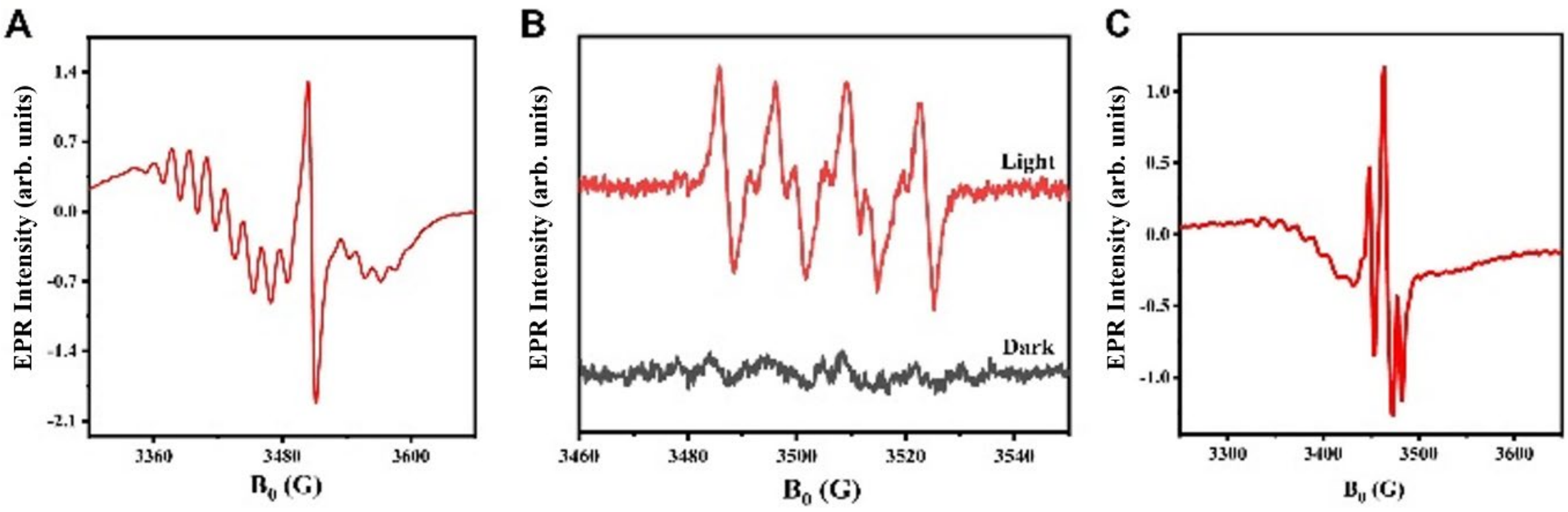

Supplementary Fig. 15. EPR spectra of Cu@f-gC$_3$N$_4$ catalyst. (**A**) solid sample; (**B**) during irradiation in ethanol under aerobic conditions showing DMPO-$O_2^{\bullet-}$ spin adduct signal (g = 2.006, A_N = 13.3; A_H^β = 10.0 G); (**C**) mixed with *N*-cyclohexyl-*N*-methylcyclohexanamine in ethanol showing the nitrogen radical signal (g = 2.003, A_N = 15).

4 Computational details

4.1 Preliminary model building and reactive molecular dynamics (RMD) simulations

We started the theoretical investigation by considering an extended atomistic model of the 2D catalyst material, including reactants, in an ethanol solution (**Supplementary Fig. 16**).

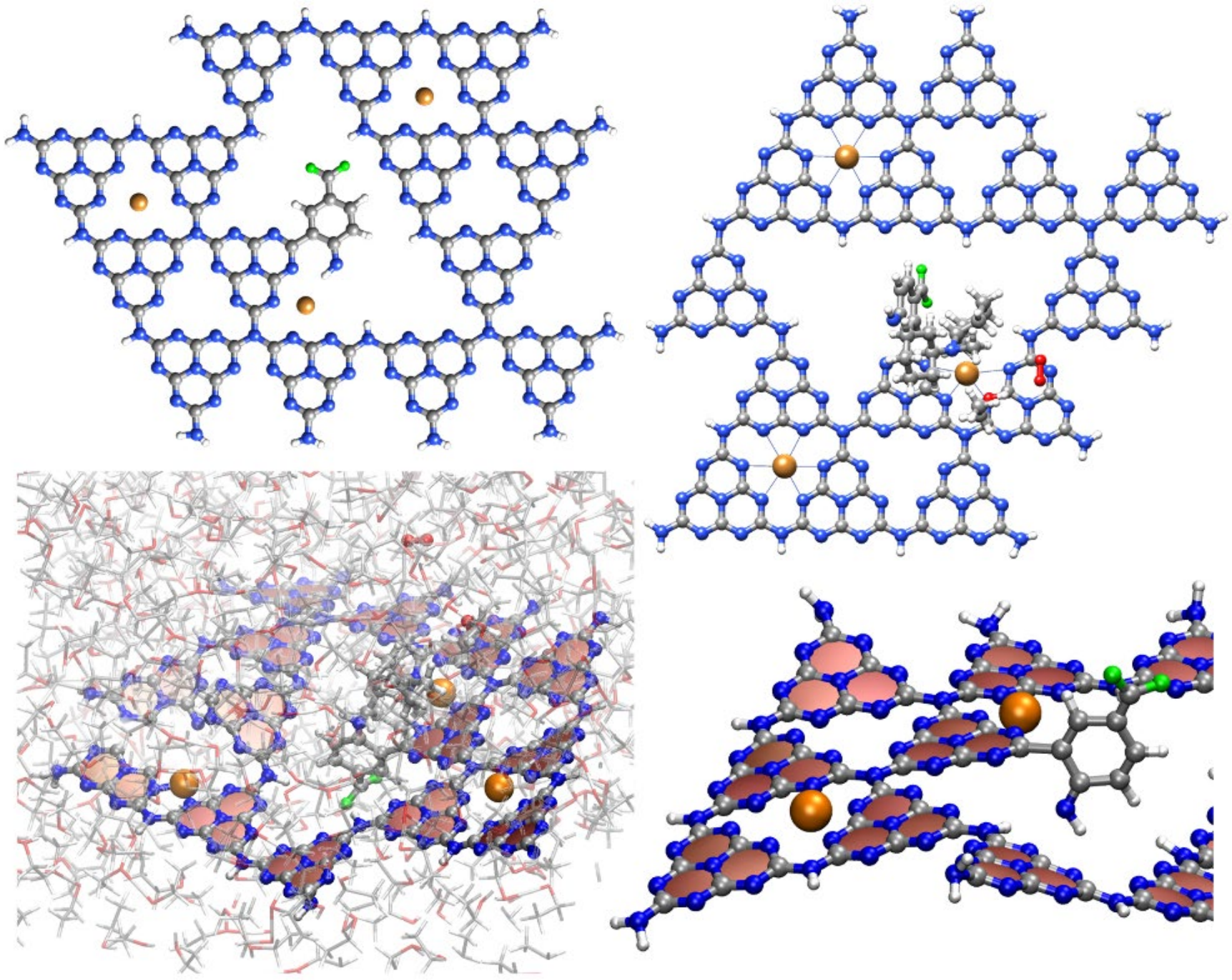

Supplementary Fig. 16. **Top left:** starting structure of the supramolecular model representing a portion of the catalyst with three Cu atoms and the functionalizing ligand. **Top-right:** optimized configuration where a tertiary amine is present (substrate 2 – **Fig. 2** of the main article – ethanol molecules have been undisplayed for clarity). **Bottom left:** a snapshot from the RMD simulations at T=300K showing the evolution of catalyst and reactants in solution.

Bottom right: evolved configuration of the catalyst during its dynamics (extracted from an RMD snapshot). The color of the ring filling indicates their deformation relative to the flat arrangement (in practice, they preserve planarity). Color coding: C gray, O red, N blue, H white, F green, and Cu orange.

We checked the stability of the whole structure, and the dynamics of the Cu atoms at room temperature and pressure by performing RMD simulations based on *ad hoc* (tested and validated) force fields (ReaxFF flavor[7-9]) in a complex environment where all the species were considered at the atomic level, and reactivity was always on.

After the system optimization and equilibration for approximately 100 ps, the production RMD was performed in the NVT ensemble for about 800 ps. The time step was set to 0.2 fs, and the Anderson thermostat controlled the temperature with a relaxation constant of 0.1 ps. The system configurations were collected every 0.05 ps. The analysis of the sampled data was focused on the last 500 ps of the trajectories. We examined atom-atom radial distribution functions (RDFs), distance distributions, and distance evolutions.

All these calculations were carried out by the ReaxFF MD code available in the LAMMPS program[10]. Visual inspection of the collected snapshots was fundamental to understanding the reactive scenarios and the reorganization of the catalyst structure.

The snapshots revealed that the 2D polymeric framework underwent significant bending and twisting in the liquid environment, preserving the planarity of the individual heptazine/triazine rings, which were not deformed internally. Cu atoms shifted from their highly ordered, symmetrical locations, visible in the starting structure, to dynamic coordination environments during the system fluctuations. The rigid holes in the initial framework deformed flexibly to encapsulate the bulky functionalizing ligand and the tertiary amine substrate, stabilizing the intermediate states via supramolecular host-guest interactions. The ethanol solvent environment heavily crowds the catalyst, driving the conformational changes through continuous intermolecular collisions and solvation forces.

Having two distinct characteristic positions of the Cu atoms (depicted in **Supplementary Fig. 17**), namely a pristine region of the supramolecular framework (top panel), and a region featuring the attached functionalizing ligand (bottom panel), we examined the behavior of the coordinated N atoms separately. **Supplementary Fig. 17** shows the distribution of the Cu-N distances, tracking the thermal structural fluctuations of the coordinated nitrogen atoms surrounding the copper centers. In both states, a highly pronounced, narrow pink peak is centered at approximately 1.85 Å. This corresponds to a strongly bound, highly stable

chemisorption distance of the N directly coordinated to the copper center. The sharp distribution confirms that these core coordination bonds remain rigid and firmly bound during the dynamics. The broad distribution centered at approximately 3.3 Å in the top plot reflects the standard non-coordinating Ns farther in the unperturbed, open macrocyclic pocket. When the functionalizing ligand is present, the far-range black peak disappears and is replaced by the red distribution in the range 2.8-3.0 Å, suggesting a confinement effect due to the presence of the bulky ligand. Indeed, the distant Ns were forced closer to the active Cu center, modifying the local electronic environment and favoring the reaction kinetics. This picture was complemented by an examination of the Cu-N radial distribution function and the evolution of various Cu-N distances (**Supplementary Fig. 18**).

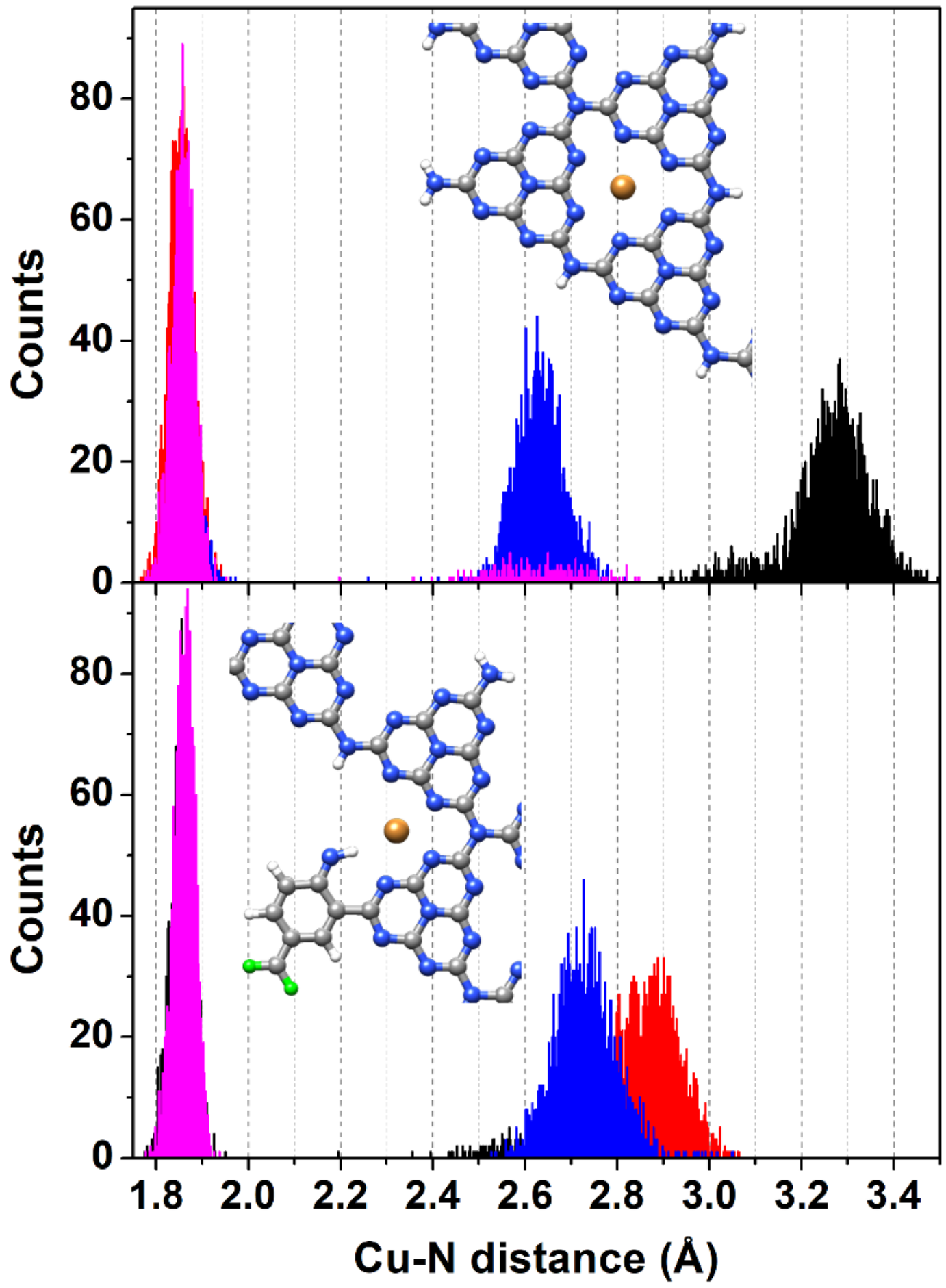

Supplementary Fig. 17. Distribution of Cu–N distances (Å) from RMD simulations, comparing unfunctionalized (**top**) and functionalized (**bottom**) supramolecular catalyst holes.

The very sharp, high-intensity RDF peak at ~1.85 Å (**Supplementary Fig. 18**) confirms a highly ordered and rigid first coordination shell between Cu and the closest framework N. In contrast, the other low peaks stretching out to 9 Å depict the maintained well-defined periodic organization of the successive nitrogen shells. The examination of the time-resolved Cu-N distances shows that structural rearrangement and re-equilibration can occur in the regions far

from the ligand. Indeed, the lines corresponding to the ligand-functionalized pocket remain constrained between 2.6 Å and 2.9 Å, due to the pocket-locking effect of the bulky ligand.
These four structural views show the continuous, undulating, wave-like rippling of the polymeric sheet across the simulation, and the uniform color filling of the ring shows that individual triazine/heptazine rings remain perfectly flat. The entire sheet accommodates stress by bending, rather than breaking or distorting its chemical bonds. Besides, the ligand remained stably anchored on the top ridge of the corrugated sheet, keeping its active site accessible to incoming substrates.

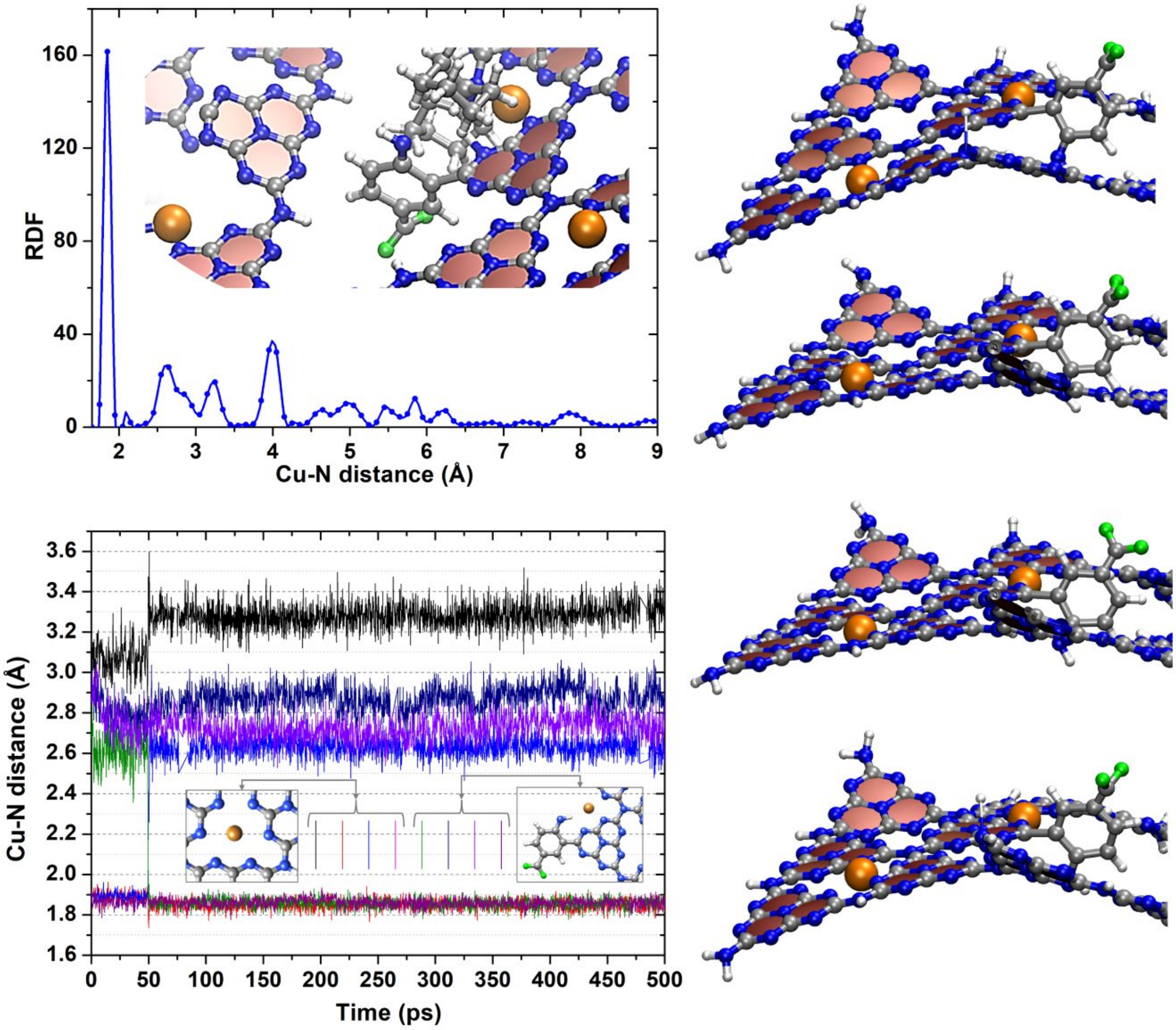

Supplementary Fig. 18. Structural evolution of the supramolecular catalyst showing long-range order through Cu–N distance radial distribution function (**top left**) and marked distance fluctuations in the pristine regions of the framework (**bottom left plots**). The simulation snapshots on the right depict macroscopic wave-like wrinkling of the 2D polymeric sheet in ethanol, while individual triazine/heptazine rings retain the local planarity.

4.2 Quantum chemistry calculations

To investigate the *N*-demethylation process using quantum-chemistry optimizations, we used a reduced model of the f-C_3N_4 PC, which has proven reliable and effective in earlier work[11-13]. This was made of two melem units functionalized by an aryl amino moiety carrying a CF_2 group, and by a Cu single atom bridging the two melem units via two N atoms (**Supplementary Fig. 19**).

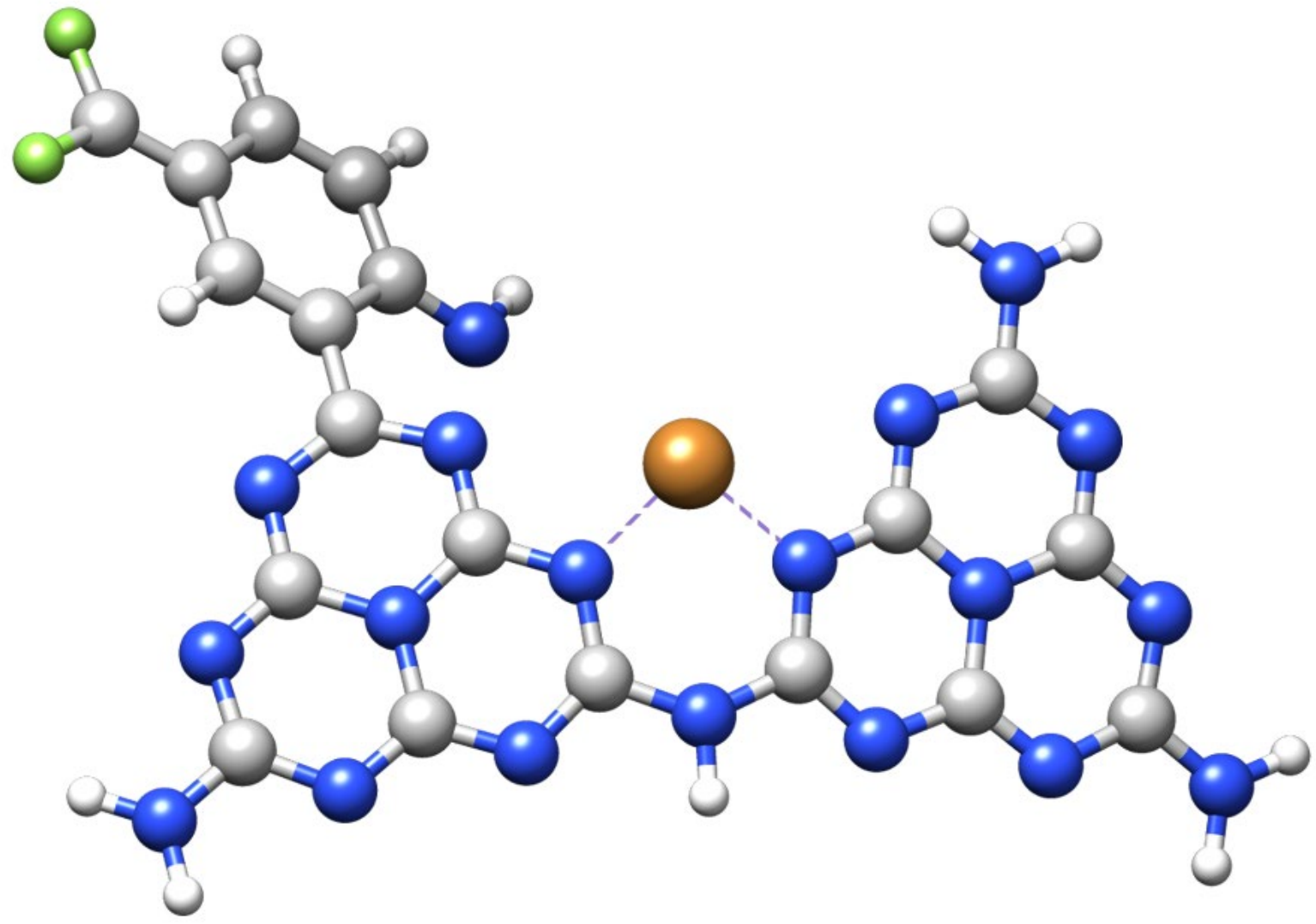

Supplementary Fig. 19: reduced model of the Cu@f-C_3N_4 PC employed in the computational investigation. Color coding: C gray, O red, N blue, H white, F green, and Cu orange.

The computational protocol for describing the reaction pathways and estimating the energy barriers was based on a combination of the GGA-Plane-Wave (PW) and Nudged Elastic Band (NEB) approaches. A localized-based method employing a hybrid XC functional was used to describe the excitation profiles, the projected density of states (PDOS), and the natural bond orbital (NBO) charges, as detailed below.

We started by performing local optimizations by using the Quantum Espresso (QE) software and reproducing possible reaction pathways via the nudged elastic band (NEB) approach implemented in QE[14]. All the calculations employed Ultrasoft (US) pseudopotentials, the generalized gradient approximation (GGA) with the Perdew–Burke–Ernzerhof (PBE) XC functional, and dispersion corrections via the Grimme D3 parametrization implemented in the QE package. Wave function and charge density cutoffs for the plane-wave basis sets of 50 and 500 Ry were employed, respectively. Single-particle wave functions were calculated spin-unrestricted by applying a smearing of the one-particle levels of 0.002 Ry, which automatically optimizes the total spin-state of the system. We employed a quite large orthorhombic unit cell

with dimensions of 22.0, 22.0, and 18.0 Å in the *x*, *y,* and *z* directions, respectively, in the presence of a +2 charge, according to the experimental indications on the local charge of the metal center of the PC.

The minima obtained with the QE software, providing the structure of the reactants and products of the different steps of the *N*-demethylation reaction, were locally optimized with the Gaussian16 software[15] using the B3LYP hybrid functional, the Grimme DFT-D3(BJ) empirical dispersion, the 6-31G(d,p) basis set for the C, N, O, F, and H atoms, and the LANL08 basis set for the Cu atom, in agreement with the approach employed in previous investigation[11]. Solvent (ethanol) was implicitly described by using the SMD method[16]. The PDOS and the MO iso-surfaces shown in Fig. 5 of the main article have been derived with such an approach. Time-dependent DFT (TDDFT) calculations were performed on the optimized geometry by requesting the first 50 roots.

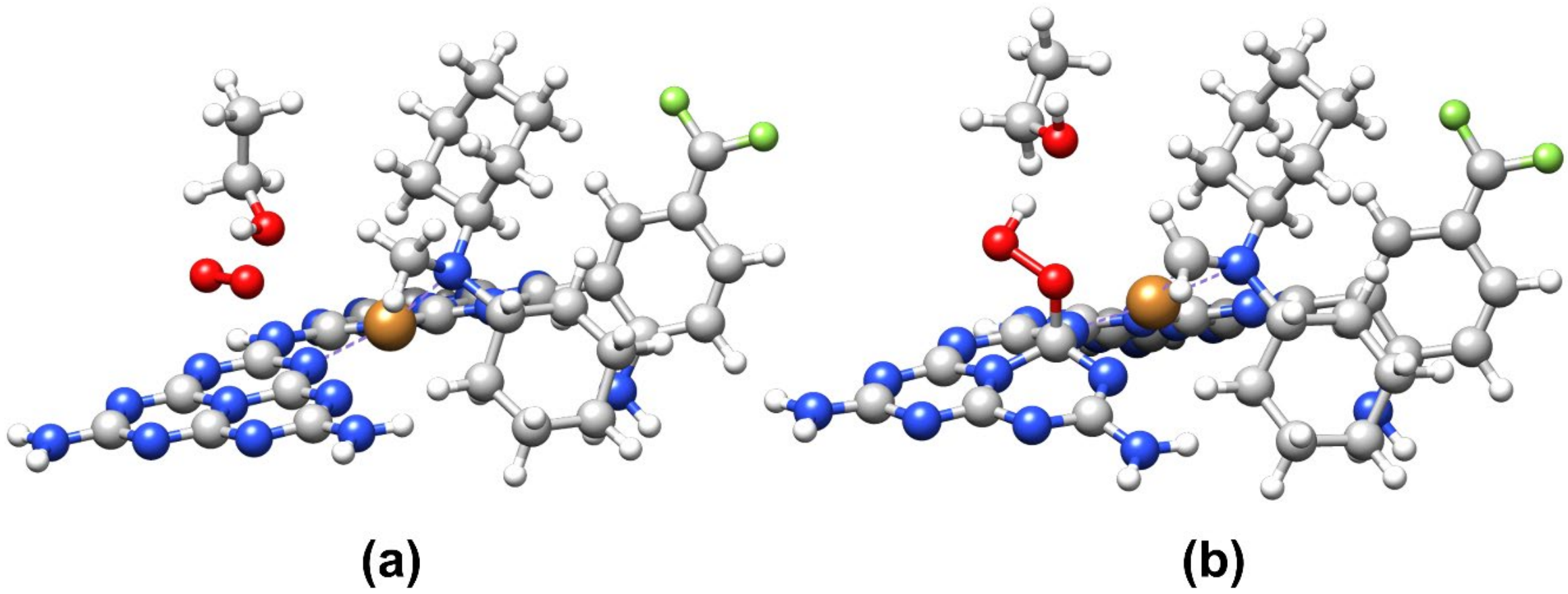

Supplementary Fig. 20: Optimized geometries before and after H transfer from the tertiary amine on the Cu SAC/O_2/EtOH system. (a) starting geometry of the tertiary amine (substrate 2 from **Fig. 2** of the main article) bound to the Cu SAC, in the presence of a molecule of O_2 and a molecule of EtOH; (b) H-transferred geometry where the resulting O_2H species is bound to a C atom of the g-C_3N_4 substrate. Color coding: C gray, O red, N blue, H white, F green, and Cu orange.

5 Characterization of products

dicyclohexylamine (2): Prepared according to the **GP5**, using the corresponding amine (0.2 mmol, 1.0 equiv.), CsOPiv (0.2 mmol, 1.0 equiv.), Cu@f-gC_3N_4 and EtOH (2 mL, 0.1 M). The reaction mixture was treated with 1 M HCl in methanol to afford **2** in 89% yield as white solid.

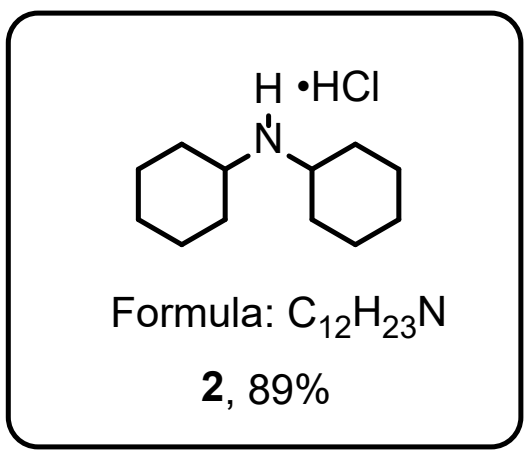

^{1}H NMR (500 MHz, MeOD) δ 3.20 (m, *J* = 11.4, 7.4, 3.8 Hz, 2H), 2.13 – 2.04 (m, 4H), 1.92 – 1.83 (m, 4H), 1.75 – 1.69 (m, 2H), 1.44 – 1.32 (m, 8H), 1.23 (m, 2H).

^{13}C NMR (126 MHz, MeOD) δ 54.44, 30.47, 26.15, 25.50.

HRMS (ESI): $[M+H]^+$ calcd. 182.1909, found 182.1895.

***N*-cyclohexyltetrahydro-2*H*-pyran-4-amine (3):** Prepared according to the **GP5**, using the corresponding amine (0.2 mmol, 1.0 equiv.), CsOPiv (0.2 mmol, 1.0 equiv.), Cu@f-gC_3N_4 and EtOH (2 mL, 0.1 M). Purification by column chromatography on silica gel using hexane/ethyl acetate (9:1 v/v) as eluent afforded **3** in 75% yield as colorless oil.

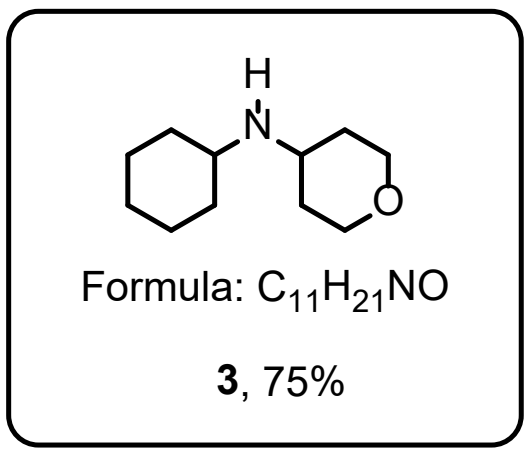

^{1}H NMR (500 MHz, MeOD) δ 4.01 (dd, *J* = 11.8, 4.7 Hz, 2H), 3.55 – 3.41 (m, 3H), 3.22 (m, 1H), 2.11 (m, 2H), 2.00 (m, 2H), 1.88 (m, 2H), 1.76 – 1.63 (m, 3H), 1.45 – 1.33 (m, 4H), 1.30 – 1.18 (m, 1H).

^{13}C NMR (126 MHz, MeOD) δ 66.91, 54.23, 51.57, 30.76, 30.56, 26.14, 25.48.

HRMS (ESI): $[M+H]^+$ calcd. 184.1701, found 184.1692.

***N*-cyclohexyl-1,4-dioxaspiro[4.5]decan-8-amine (4):** Prepared according to the **GP5**, using the corresponding amine (0.2 mmol, 1.0 equiv.), CsOPiv (0.2 mmol, 1.0 equiv.), Cu@f-gC_3N_4 and EtOH (2 mL, 0.1 M). Purification by column chromatography on silica gel using hexane/ethyl acetate (9:1 v/v) as eluent afforded **4** in 78% yield as white solid.

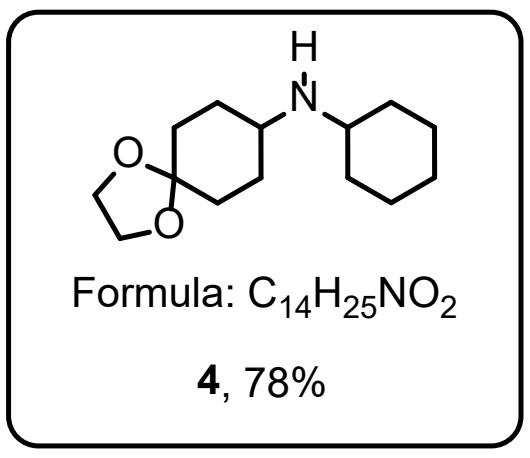

^{1}H NMR (500 MHz, $CDCl_3$) δ 3.88 (s, 4H), 3.13 – 2.96 (m, 2H), 2.51 (m, 1H), 2.38 – 2.25 (m, 1H), 2.24 – 2.15 (m, 4H), 2.02 (m, 2H), 1.81 (m, 4H), 1.76 – 1.57 (m, 4H), 1.51 (td, *J* = 13.6, 4.0 Hz, 2H).

^{13}C NMR (126 MHz, $CDCl_3$) δ 107.04, 64.59, 64.49, 54.67, 52.76, 38.48, 33.22, 29.32, 29.22, 27.70, 26.56.

HRMS (ESI): $[M+H]^+$ calcd. 240.1964, found 240.1950.

***N*-cyclopentylcyclohexanamine (5):** Prepared according to the **GP5**, using the corresponding amine (0.2 mmol, 1.0 equiv.), CsOPiv (0.2 mmol, 1.0 equiv.), Cu@f-gC_3N_4 and EtOH (2 mL, 0.1 M). Purification by column chromatography on silica gel using hexane/ethyl acetate (9:1 v/v) as eluent afforded **5** in 72% yield as pale yellow oil.

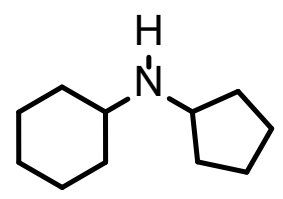

Formula: $C_{11}H_{21}N$

5, 72%

^{1}H NMR (500 MHz, $CDCl_3$) δ 3.12 (p, *J* = 7.1 Hz, 1H), 2.38 (tt, *J* = 10.6, 3.8 Hz, 1H), 1.84 – 1.74 (m, 4H), 1.68 – 1.51 (m, 5H), 1.45 (m, 2H), 1.23 – 1.13 (m, 4H), 1.07 (m, 1H), 1.01 – 0.92 (m, 2H).

^{13}C NMR (126 MHz, $CDCl_3$) δ 56.53, 55.22, 34.11, 33.88, 33.75, 29.70, 26.21, 25.27, 23.92.

Experimental data are in accordance with the literature[17].

dicyclopentylamine (6): Prepared according to the **GP5**, using the corresponding amine (0.2 mmol, 1.0 equiv.), CsOPiv (0.2 mmol, 1.0 equiv.), Cu@f-gC_3N_4 and EtOH (2 mL, 0.1 M). Purification by column chromatography on silica gel using hexane/ethyl acetate (9:1 v/v) as eluent afforded **6** in 76% yield as pale yellow liquid.

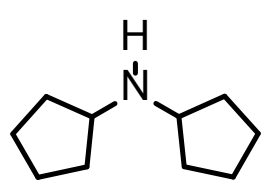

Formula: $C_{10}H_{19}N$

6, 76%

^{1}H NMR (500 MHz, $CDCl_3$) δ 3.06 (p, *J* = 7.2 Hz, 2H), 1.89 – 1.78 (m, 4H), 1.63 (m, 4H), 1.54 – 1.43 (m, 4H), 1.24 (m, 4H).

^{13}C NMR (126 MHz, $CDCl_3$) δ 58.64, 33.75, 33.63, 24.04.

Experimental data are in accordance with the literature[18].

***N*-cyclohexylcyclododecanamine (7):** Prepared according to the **GP5**, using the corresponding amine (0.2 mmol, 1.0 equiv.), CsOPiv (0.2 mmol, 1.0 equiv.), Cu@f-gC_3N_4 and EtOH (2 mL, 0.1 M). Purification by column chromatography on silica gel using hexane/ethyl acetate (9:1 v/v) as eluent afforded **7** in 51% white solid.

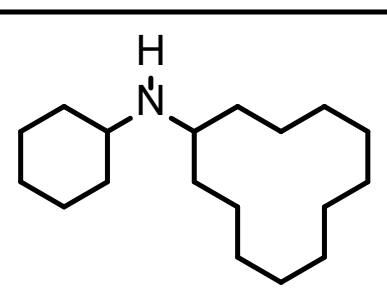

Formula: $C_{18}H_{35}N$

7, 51%

^{1}H NMR (500 MHz, $CDCl_3$) δ 3.21 (m, 1H), 2.94 (m, 1H), 2.22 – 2.12 (m, 2H), 1.78 (m, 8H), 1.56 – 1.41 (m, 4H), 1.38 – 1.08 (m, 18H).

^{13}C NMR (126 MHz, $CDCl_3$) δ 54.94, 52.84, 30.84, 29.24, 26.37, 24.86, 24.73, 24.27, 23.11, 22.97, 21.02.

HRMS (ESI): $[M+H]^+$ calcd. 266.2848, found 266.2857.

N-cyclohexylbicyclo[2.2.1]heptan-2-amine (8): Prepared according to the **GP5**, using the corresponding amine (0.2 mmol, 1.0 equiv.), CsOPiv (0.2 mmol, 1.0 equiv.), Cu@f-gC_3N_4 and EtOH (2 mL, 0.1 M). Purification by column chromatography on silica gel using hexane/ethyl acetate (9:1 v/v) as eluent afforded **8** in 55% yield as white solid.

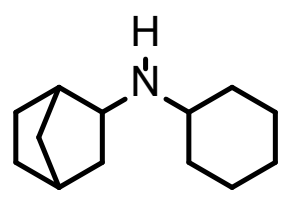

Formula: $C_{13}H_{23}N$

8, 55%

^{1}H NMR (500 MHz, $CDCl_3$) δ 3.50 (m, 1H), 2.91 (m, 1H), 2.53 – 2.44 (m, 2H), 2.31 – 2.26 (m, 1H), 2.16 (m, 1H), 2.05 – 1.92 (m, 2H), 1.90 – 1.76 (m, 3H), 1.75 – 1.60 (m, 4H), 1.59 – 1.49 (m, 2H), 1.38 (m, 2H), 1.31 – 1.15 (m, 3H).

^{13}C NMR (126 MHz, $CDCl_3$) δ 77.36, 58.55, 57.19, 56.59, 56.24, 39.48, 38.48, 38.20, 37.00, 36.57, 36.47, 35.68, 33.36, 30.36, 29.54, 29.37, 29.30, 28.29, 27.91, 27.74, 25.02, 24.99, 24.93, 24.88, 22.02.

HRMS (ESI): $[M+H]^+$ calcd. 194.1909, found 194.1908.

azepan-2-one (9): Prepared according to the **GP5**, using the corresponding amine (0.2 mmol, 1.0 equiv.), CsOPiv (0.2 mmol, 1.0 equiv.), Cu@f-gC_3N_4 and EtOH (2 mL, 0.1 M). The reaction mixture was treated with 1 M HCl in methanol to afford **9** in 47% yield as white solid.

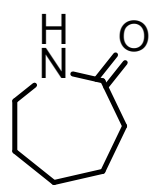

Formula: $C_6H_{11}NO$

9, 47%

^{1}H NMR (500 MHz, $CDCl_3$) δ 6.38 (s, 1H), 3.24 – 3.15 (m, 2H), 2.50 – 2.39 (m, 2H), 1.80 – 1.71 (m, 2H), 1.71 – 1.60 (m, 4H).

^{13}C NMR (126 MHz, $CDCl_3$) δ 183.00, 44.14, 33.54, 30.03, 29.90, 27.17, 22.10.

Experimental data are in accordance with the literature[19].

2,2,6,6-tetramethylpiperidine (10): Prepared according to the **GP5**, using the corresponding amine (0.2 mmol, 1.0 equiv.), CsOPiv (0.2 mmol, 1.0 equiv.), Cu@f-gC_3N_4 and EtOH (2 mL, 0.1 M). The reaction mixture was treated with 1 M HCl in methanol to afford **10** in 69% yield as white solid.

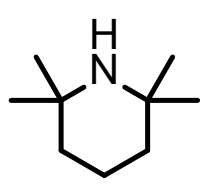

Formula: $C_9H_{19}N$

10, 69%

^{1}H NMR (500 MHz, MeOD) δ 1.84 – 1.78 (m, 2H), 1.70 – 1.64 (m, 4H), 1.44 (s, 12H).

^{13}C NMR (126 MHz, MeOD) δ 57.86, 35.97, 27.62, 17.14.

HRMS (ESI): $[M+H]^+$ calcd. 142.1590, found 142.1589.

Experimental data are in accordance with the literature[20].

2,2,6,6-tetramethylpiperidin-4-one (11): Prepared according to the **GP5**, using the corresponding amine (0.2 mmol, 1.0 equiv.), CsOPiv (0.2 mmol, 1.0 equiv.), Cu@f-gC_3N_4 and EtOH (2 mL, 0.1 M). Purification by column chromatography on silica gel using hexane/ethyl acetate (9:1 v/v) as eluent afforded **11** in 68% yield as white solid.

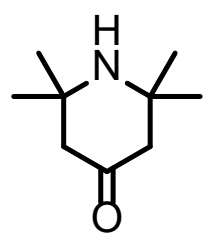

Formula: $C_9H_{17}NO$

11, 68%

^{1}H NMR (500 MHz, $CDCl_3$) δ 2.25 (s, 4H), 1.21 (s, 12H).

^{13}C NMR (126 MHz, $CDCl_3$) δ 211.21, 55.52, 54.24, 32.09.

Experimental data are in accordance with the literature[21].

2,2,6,6-tetramethylpiperidin-4-ol (12): Prepared according to the **GP5**, using the corresponding amine (0.2 mmol, 1.0 equiv.), CsOPiv (0.2 mmol, 1.0 equiv.), Cu@f-gC_3N_4 and EtOH (2 mL, 0.1 M). The reaction mixture was treated with 1 M HCl in methanol to afford **12** in 65% yield as white solid.

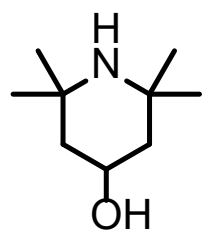

Formula: $C_9H_{19}NO$

12, 65%

^{1}H NMR (500 MHz, $CDCl_3$) δ 3.92 (tt, *J* = 11.5, 4.1 Hz, 1H), 2.23 (s, 3H), 1.83 (ddt, *J* = 12.2, 4.3, 1.4 Hz, 2H), 1.39 – 1.33 (m, 2H), 1.15 (s, 6H), 1.02 (s, 6H).

^{13}C NMR (126 MHz, MeOD) δ 64.99, 52.88, 48.30, 34.40, 28.35.

Experimental data are in accordance with the literature[22].

2,2,6,6-tetramethylpiperidin-4-yl methacrylate (13): Prepared according to the **GP5**, using the corresponding amine (0.2 mmol, 1.0 equiv.), CsOPiv (0.2 mmol, 1.0 equiv.), Cu@f-gC_3N_4 and EtOH (2 mL, 0.1 M). Purification by column chromatography on silica gel using hexane/ethyl acetate (9:1 v/v) as eluent afforded **13** in 63% yield as white solid.

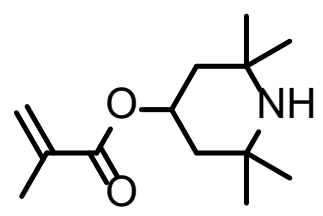

Formula: $C_{13}H_{23}NO_2$

13, 63%

^{1}H NMR (500 MHz, $CDCl_3$) δ 6.04 (m, 1H), 5.51 (p, *J* = 1.6 Hz, 1H), 5.22 (m, 1H), 1.95 – 1.90 (m, 2H), 1.90 (t, *J* = 1.3 Hz, 3H), 1.22 (s, 6H), 1.17 (t, *J* = 11.8 Hz, 2H), 1.13 (s, 6H).

^{13}C NMR (126 MHz, $CDCl_3$) δ 167.06, 136.82, 125.19, 69.17, 51.53, 43.90, 34.84, 29.78, 29.12, 18.38.

Experimental data are in accordance with the literature[23].

***N*,*N*,2,2,6,6-hexamethylpiperidin-4-amine (14):** Prepared according to the **GP5**, using the corresponding amine (0.2 mmol, 1.0 equiv.), CsOPiv (0.2 mmol, 1.0 equiv.), Cu@f-gC_3N_4 and EtOH (2 mL, 0.1 M). Purification by column chromatography on silica gel using hexane/ethyl acetate (4:1 v/v) as eluent afforded **14** in 62% yield as white solid

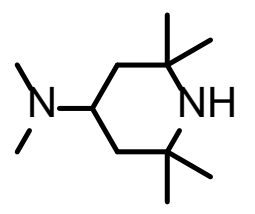

Formula: $C_{11}H_{24}N_2$

14, 62%

^{1}H NMR (500 MHz, CDCl$_3$) δ 2.54 (tt, *J* = 12.1, 3.3 Hz, 1H), 2.16 (s, 6H), 1.66 – 1.58 (m, 2H), 1.07 (s, 6H), 1.01 (s, 6H), 0.86 (t, *J* = 12.2 Hz, 2H).

^{13}C NMR (126 MHz, CDCl$_3$) δ 56.19, 50.95, 48.32, 41.21, 41.01, 41.00, 35.31, 28.60.

Experimental data are in accordance with the literature[24].

bis(2,2,6,6-tetramethylpiperidin-4-yl) decanedioate (15): Prepared according to the **GP5**, using the corresponding amine (0.2 mmol, 1.0 equiv.), CsOPiv (0.2 mmol, 1.0 equiv.), Cu@f-gC_3N_4 and EtOH (2 mL, 0.1 M). Purification by column chromatography on silica gel using DCM/MeOH (9:2 v/v) as eluent afforded **15** in 51% yield as white solid.

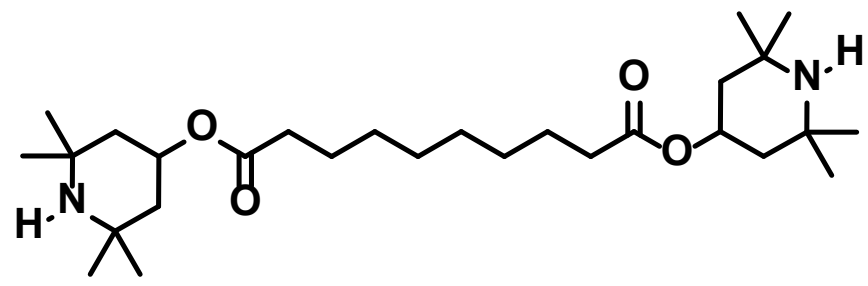

Formula: $C_{28}H_{52}N_2O_4$

15, 51%

^{1}H NMR (500 MHz, CDCl$_3$) δ 5.18 (m, 2H), 2.26 (t, *J* = 7.5 Hz, 4H), 1.92 – 1.87 (m, 4H), 1.59 (m, 4H), 1.29 (m, 8H), 1.23 (s, 12H), 1.19 – 1.01 (m, 18H).

^{13}C NMR (126 MHz, CDCl$_3$) δ 173.53, 68.57, 51.69, 44.02, 34.86, 34.78, 29.22, 29.18, 28.99, 25.10.

HRMS (ESI): $[M+H]^+$ calcd. 481.4000, found 481.3991.

***N*-cyclopentylaniline (16):** Prepared according to the **GP5**, using the corresponding amine (0.2 mmol, 1.0 equiv.), CsOPiv (0.2 mmol, 1.0 equiv.), Cu@f-gC_3N_4 and EtOH (2 mL, 0.1 M). Purification by column chromatography on silica gel using hexane/ethyl acetate (20:1 v/v) as eluent afforded **16** in 75% yield as pale yellow oil.

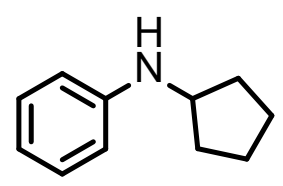

Formula: $C_{11}H_{15}N$

16, 75%

^{1}H NMR (500 MHz, CDCl$_3$) δ 7.19 – 7.14 (m, 2H), 6.68 (tt, *J* = 7.3, 1.1 Hz, 1H), 6.63 – 6.59 (m, 2H), 3.83 – 3.76 (m, 1H), 3.74 – 3.55 (m, 1H), 2.06 – 1.99 (m, 2H), 1.72 (m, 2H), 1.63 (m, 2H), 1.51 – 1.44 (m, 2H).

^{13}C NMR (126 MHz, CDCl$_3$) δ 148.17, 129.32, 117.01, 113.27, 54.76, 33.73, 24.21.

Experimental data are in accordance with the literature[25].

4-chloro-*N*-cyclopentylaniline (17): Prepared according to the **GP5**, using the corresponding amine (0.2 mmol, 1.0 equiv.), CsOPiv (0.2 mmol, 1.0 equiv.), Cu@f-gC_3N_4 and EtOH (2 mL, 0.1 M). Purification by column chromatography on silica gel using hexane/ethyl acetate (20:1 v/v) as eluent afforded **17** in 69% yield as pale yellow oil.

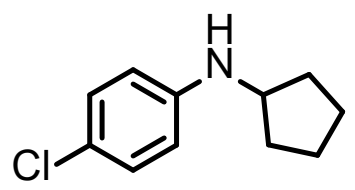

Formula: $C_{11}H_{14}ClN$

17, 69%

^{1}H NMR (500 MHz, $CDCl_3$) δ 7.13 – 7.08 (m, 2H), 6.53 – 6.49 (m, 2H), 3.74 (m, 1H), 3.70 – 3.57 (m, 1H), 2.01 (m, 2H), 1.77 – 1.68 (m, 2H), 1.67 – 1.58 (m, 2H), 1.48 – 1.41 (m, 2H).
^{13}C NMR (126 MHz, $CDCl_3$) δ 146.69, 129.09, 121.49, 114.28, 54.86, 33.59, 24.17.
Experimental data are in accordance with the literature[26].

***N*-cyclopentyl-4-(trifluoromethyl)aniline (18):** Prepared according to the **GP5**, using the corresponding amine (0.2 mmol, 1.0 equiv.), CsOPiv (0.2 mmol, 1.0 equiv.), Cu@f-gC_3N_4 and EtOH (2 mL, 0.1 M). Purification by column chromatography on silica gel using hexane/ethyl acetate (20:1 v/v) as eluent afforded **18** in 72% yield as pale yellow oil.

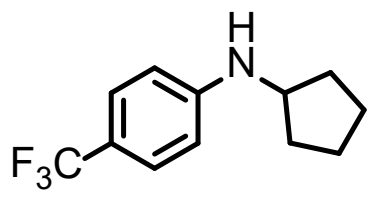

Formula: $C_{12}H_{14}F_3N$

18, 72%

^{1}H NMR (500 MHz, $CDCl_3$) δ 7.41 – 7.35 (m, 2H), 6.60 – 6.56 (m, 2H), 3.99 (s, 1H), 3.85 – 3.77 (m, 1H), 2.08 – 2.00 (m, 2H), 1.78 – 1.69 (m, 2H), 1.69 – 1.60 (m, 2H), 1.51 – 1.43 (m, 2H).
^{13}C NMR (126 MHz, $CDCl_3$) δ 150.49, 126.66 (q, J = 3.7 Hz), 125.21 (q, J = 270.2 Hz), 118.32 (q, J = 32.6 Hz), 112.21, 54.43, 33.57, 24.14.
^{19}F NMR (471 MHz, $CDCl_3$) δ -60.88.
Experimental data are in accordance with the literature[27].

***N*-cyclopentyl-4-methoxyaniline (19):** Prepared according to the **GP5**, using the corresponding amine (0.2 mmol, 1.0 equiv.), CsOPiv (0.2 mmol, 1.0 equiv.), Cu@f-gC_3N_4 and EtOH (2 mL, 0.1 M). Purification by column chromatography on silica gel using hexane/ethyl acetate (9:1 v/v) as eluent afforded **19** in 55% yield as pale yellow oil.

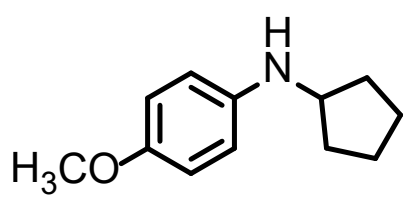

Formula: $C_{12}H_{17}NO$

19, 55%

^{1}H NMR (500 MHz, $CDCl_3$) δ 6.83 – 6.75 (m, 2H), 6.62 – 6.56 (m, 2H), 3.76 (s, 3H), 3.75 – 3.71 (m, 1H), 3.38 (s, 1H), 2.01 (m, 2H), 1.72 (s, 2H), 1.68 – 1.57 (m, 2H), 1.46 (m, 2H).
^{13}C NMR (126 MHz, $CDCl_3$) δ 151.90, 142.46, 114.93, 114.62, 77.42, 76.91, 55.89, 55.58, 33.68, 24.20.
Experimental data are in accordance with the literature[28].

***N*-cyclohexylaniline (20):** Prepared according to the **GP5**, using the corresponding amine (0.2 mmol, 1.0 equiv.), CsOPiv (0.2 mmol, 1.0 equiv.), Cu@f-gC_3N_4 and EtOH (2 mL, 0.1 M). Purification by column chromatography on silica gel using hexane/ethyl acetate (20:1 v/v) as eluent afforded **20** in 60% yield as pale yellow oil.

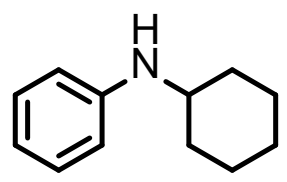

Formula: $C_{12}H_{17}N$

20, 60%

^{1}H NMR (500 MHz, CDCl$_3$) δ 7.23 – 7.16 (m, 2H), 6.70 (tt, *J* = 7.3, 1.2 Hz, 1H), 6.66 – 6.59 (m, 2H), 3.55 (s, 1H), 3.29 (m, 1H), 2.16 – 2.05 (m, 2H), 1.80 (m, 2H), 1.75 – 1.65 (m, 1H), 1.48 – 1.35 (m, 2H), 1.33 – 1.13 (m, 3H).
^{13}C NMR (126 MHz, CDCl$_3$) δ 147.49, 129.35, 116.89, 113.20, 51.74, 33.57, 26.04, 25.14.
HRMS (ESI): $[M+H]^+$ calcd. 176.1434, found 176.1429.

Experimental data are in accordance with the literature[29].

***N*-cyclohexyl-4-fluoroaniline (21):** Prepared according to the **GP5**, using the corresponding amine (0.2 mmol, 1.0 equiv.), CsOPiv (0.2 mmol, 1.0 equiv.), Cu@f-gC_3N_4 and EtOH (2 mL, 0.1 M). Purification by column chromatography on silica gel using hexane/ethyl acetate (20:1 v/v) as eluent afforded **21** in 70% yield as yellow oil.

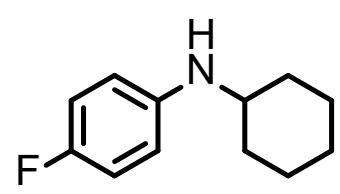

Formula: $C_{12}H_{16}FN$

21, 70%

^{1}H NMR (500 MHz, CDCl$_3$) δ 6.89 – 6.83 (m, 2H), 6.54 – 6.50 (m, 2H), 3.47 – 3.26 (m, 1H), 3.17 (m, 1H), 2.07 – 2.01 (m, 2H), 1.76 (m, 2H), 1.68 – 1.62 (m, 1H), 1.40 – 1.31 (m, 2H), 1.27 – 1.20 (m, 1H), 1.12 m, 2H).
^{13}C NMR (126 MHz, CDCl$_3$) δ 156.56, 154.70, 143.84 (d, *J* = 1.8 Hz), 115.85, 115.67, 114.21, 114.16, 52.58, 33.60, 26.06, 25.16.
^{19}F NMR (471 MHz, CDCl$_3$) δ -128.91 (m, 1F).
Experimental data are in accordance with the literature[30].

4-bromo-*N*-cyclohexylaniline (22): Prepared according to the **GP5**, using the corresponding amine (0.2 mmol, 1.0 equiv.), CsOPiv (0.2 mmol, 1.0 equiv.), Cu@f-gC_3N_4 and EtOH (2 mL, 0.1 M). Purification by column chromatography on silica gel using hexane/ethyl acetate (20:1 v/v) as eluent afforded **22** in 63% yield as pale yellow oil.

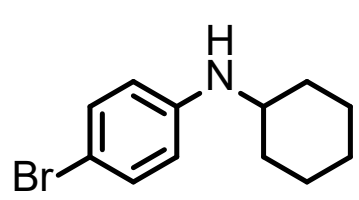

Formula: $C_{12}H_{16}BrN$

22, 63%

^{1}H NMR (500 MHz, CDCl$_3$) δ 7.24 – 7.19 (m, 2H), 6.47 – 6.44 (m, 2H), 3.54 (s, 1H), 3.20 (m, 1H), 2.03 (m, 2H), 1.76 (m, 2H), 1.65 (m, 1H), 1.41 – 1.30 (m, 2H), 1.25 – 1.19 (m, 1H), 1.13 m, 2H).

^{13}C NMR (126 MHz, $CDCl_3$) δ 146.47, 132.04, 114.77, 108.28, 51.87, 33.40, 25.99, 25.08. Experimental data are in accordance with the literature[30].

***N*-cyclohexyl-4-nitroaniline (23):** Prepared according to the **GP5**, using the corresponding amine (0.2 mmol, 1.0 equiv.), CsOPiv (0.2 mmol, 1.0 equiv.), Cu@f-gC_3N_4 and EtOH (2 mL, 0.1 M). Purification by column chromatography on silica gel using hexane/ethyl acetate (9:1 v/v) as eluent afforded **23** in 68% yield as yellow solid.

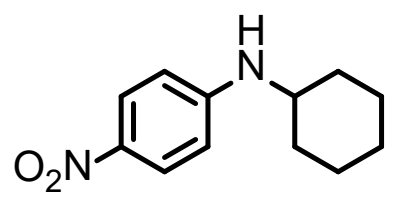

Formula: $C_{12}H_{16}N_2O_2$

23, 68%

^{1}H NMR (500 MHz, $CDCl_3$) δ 8.14 – 7.97 (m, 2H), 6.56 – 6.37 (m, 2H), 4.42 (s, 1H), 3.44 – 3.27 (m, 1H), 2.06 (m, 2H), 1.83 – 1.76 (m, 2H), 1.72 – 1.64 (m, 1H), 1.45 – 1.35 (m, 2H), 1.28 – 1.20 (m, 3H).

^{13}C NMR (126 MHz, $CDCl_3$) δ 152.56, 137.60, 126.71, 111.27, 51.67, 33.06, 25.69, 24.88. Experimental data are in accordance with the literature[31].

***N*-cyclohexyl-3-methylaniline (24):** Prepared according to the **GP5**, using the corresponding amine (0.2 mmol, 1.0 equiv.), CsOPiv (0.2 mmol, 1.0 equiv.), Cu@f-gC_3N_4 and EtOH (2 mL, 0.1 M). Purification by column chromatography on silica gel using hexane/ethyl acetate (20:1 v/v) as eluent afforded **24** in 68% yield as pale yellow oil.

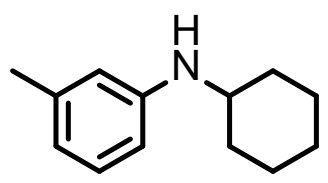

Formula: $C_{13}H_{19}N$

24, 68%

^{1}H NMR (500 MHz, $CDCl_3$) δ 7.05 (m, 1H), 6.51 – 6.48 (m, 1H), 6.43 – 6.39 (m, 2H), 3.47 (s, 1H), 3.25 (m, 1H), 2.27 (s, 3H), 2.10 – 2.01 (m, 2H), 1.79 – 1.73 (m, 2H), 1.66 (m, 1H), 1.42 – 1.33 (m, 2H), 1.27 – 1.20 (m, 1H), 1.14 (m, 2H).

^{13}C NMR (126 MHz, $CDCl_3$) δ 147.55, 139.14, 129.26, 117.88, 114.00, 110.36, 51.75, 33.67, 26.08, 25.17, 21.79.

Experimental data are in accordance with the literature[30].

***N*-(4,4-dimethoxycyclohexyl)aniline (25):** Prepared according to the **GP5**, using the corresponding amine (0.2 mmol, 1.0 equiv.), CsOPiv (0.2 mmol, 1.0 equiv.), Cu@f-gC_3N_4 and EtOH (2 mL, 0.1 M). Purification by column chromatography on silica gel using hexane/ethyl acetate (20:1 v/v) as eluent afforded **25** in 58% yield as pale yellow oil.

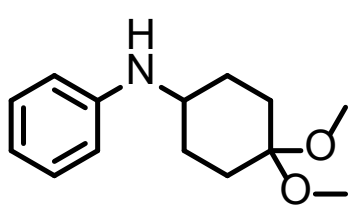

Formula: $C_{14}H_{21}NO_2$

25, 58%

^{1}H NMR (500 MHz, $CDCl_3$) δ 7.19 – 7.14 (m, 2H), 6.68 (tt, *J* = 7.3, 1.1 Hz, 1H), 6.62 – 6.57 (m, 2H), 3.53 (s, 1H), 3.35 (m, 1H), 3.20 (d, *J* = 16.8 Hz, 6H), 2.03 – 1.94 (m, 4H), 1.57 – 1.49 (m, 2H), 1.41 (m, 2H).

^{13}C NMR (126 MHz, CDCl$_3$) δ 147.39, 129.44, 117.22, 113.30, 99.57, 50.73, 48.01, 47.59, 30.70, 29.08.
HRMS (ESI): $[M+H]^+$ calcd. 236.1645, found 204,13777 (-OCH$_3$).

***N*-phenyl-1,4-dioxaspiro[4.5]decan-8-amine (26):** Prepared according to the **GP5**, using the corresponding amine (0.2 mmol, 1.0 equiv.), CsOPiv (0.2 mmol, 1.0 equiv.), Cu@f-gC_3N_4 and EtOH (2 mL, 0.1 M). Purification by column chromatography on silica gel using hexane/ethyl acetate (20:1 v/v) as eluent afforded **26** in 62% yield as white solid.

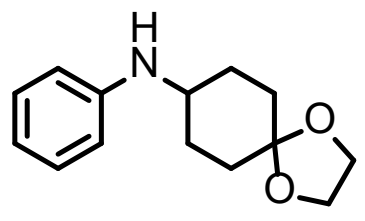

Formula: $C_{14}H_{19}NO_2$
26, 62%

^{1}H NMR (500 MHz, CDCl$_3$) δ 7.19 – 7.14 (m, 2H), 6.68 (tt, *J* = 7.3, 1.1 Hz, 1H), 6.62 – 6.58 (m, 2H), 3.96 (s, 4H), 3.53 (s, 1H), 3.38 (tt, *J* = 9.8, 3.8 Hz, 1H), 2.09 – 2.02 (m, 2H), 1.84 – 1.77 (m, 2H), 1.67 (td, *J* = 12.8, 4.1 Hz, 2H), 1.52 (m, 2H).
^{13}C NMR (126 MHz, CDCl$_3$) δ 147.39, 129.45, 117.24, 113.32, 108.45, 64.52, 64.44, 50.32, 33.22, 30.17.
HRMS (ESI): $[M+H]^+$ calcd. 234.1489, found 234.1483.

Experimental data are in accordance with the literature[32].

4-(phenylamino)cyclohexan-1-one (27): Prepared according to the **GP5**, using the corresponding amine (0.2 mmol, 1.0 equiv.), CsOPiv (0.2 mmol, 1.0 equiv.), Cu@f-gC_3N_4 and EtOH (2 mL, 0.1 M). Purification by column chromatography on silica gel using hexane/ethyl acetate (20:1 v/v) as eluent afforded **27** in 55% yield as colorless solid.

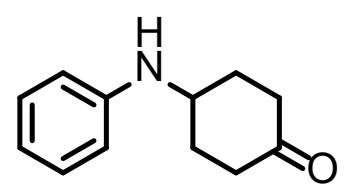

Formula: $C_{12}H_{15}NO$
27, 55%

^{1}H NMR (500 MHz, CDCl$_3$) δ 7.23 – 7.18 (m, 2H), 6.73 (tt, *J* = 7.3, 1.2 Hz, 1H), 6.67 – 6.63 (m, 2H), 3.77 (tt, *J* = 9.4, 3.7 Hz, 1H), 3.60 (s, 1H), 2.52 – 2.43 (m, 4H), 2.38 – 2.30 (m, 2H), 1.78 – 1.72 (m, 2H).
^{13}C NMR (126 MHz, CDCl$_3$) δ 210.49, 146.92, 129.59, 117.91, 113.44, 49.69, 39.06, 32.22.
HRMS (ESI): $[M+H]^+$ calcd. 190.1226, found 190.1232.

9*H*-carbazole (28): Prepared according to the **GP5**, using the corresponding amine (0.2 mmol, 1.0 equiv.), CsOPiv (0.2 mmol, 1.0 equiv.), Cu@f-gC_3N_4 and EtOH (2 mL, 0.1 M). Purification by column chromatography on silica gel using hexane/ethyl acetate (15:1 v/v) as eluent afforded **28** in 52% yield as white solid.

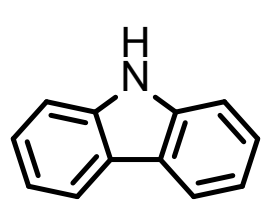

Formula: $C_{12}H_9N$
28, 52%

^{1}H NMR (500 MHz, $CDCl_3$) δ 8.09 (d, *J* = 7.9 Hz, 2H), 8.04 (s, 1H), 7.51 – 7.38 (m, 3H), 7.31 – 7.19 (m, 2H).

^{13}C NMR (126 MHz, $CDCl_3$) δ 139.58, 125.97, 123.46, 120.47, 119.57, 110.70.

Experimental data are in accordance with the literature[33].

diphenylamine (29): Prepared according to the **GP5**, using the corresponding amine (0.2 mmol, 1.0 equiv.), CsOPiv (0.2 mmol, 1.0 equiv.), Cu@f-gC_3N_4 and EtOH (2 mL, 0.1 M). Purification by column chromatography on silica gel using hexane/ethyl acetate (15:1 v/v) as eluent afforded **29** in 54% yield as brown solid.

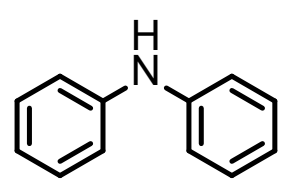

Formula: $C_{12}H_{11}N$

29, 54%

^{1}H NMR (500 MHz, $CDCl_3$) δ 7.31 – 7.26 (m, 4H), 7.11 – 7.07 (m, 4H), 6.94 (tt, *J* = 7.3, 1.2 Hz, 2H), 5.71 (s, 1H).

^{13}C NMR (126 MHz, $CDCl_3$) δ 143.22, 129.48, 121.12, 117.92.

Experimental data are in accordance with the literature[34].

bis(4-methoxyphenyl)amine (30): Prepared according to the **GP5**, using the corresponding amine (0.2 mmol, 1.0 equiv.), CsOPiv (0.2 mmol, 1.0 equiv.), Cu@f-gC_3N_4 and EtOH (2 mL, 0.1 M). Purification by column chromatography on silica gel using hexane/ethyl acetate (9:1 v/v) as eluent afforded **30** in 47% yield as brown solid.

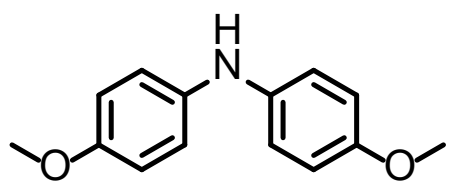

Formula: $C_{14}H_{15}NO_2$

30, 47%

^{1}H NMR (500 MHz, $CDCl_3$) δ 6.98 – 6.93 (m, 4H), 6.87 – 6.82 (m, 4H), 5.32 (s, 1H), 3.79 (s, 6H).

^{13}C NMR (126 MHz, $CDCl_3$) δ 154.28, 138.02, 119.60, 114.79, 55.72.

Experimental data are in accordance with the literature[35].

4-methyl-*N*-phenylaniline (31): Prepared according to the **GP5**, using the corresponding amine (0.2 mmol, 1.0 equiv.), CsOPiv (0.2 mmol, 1.0 equiv.), Cu@f-gC_3N_4 and EtOH (2 mL, 0.1 M). Purification by column chromatography on silica gel using hexane/ethyl acetate (20:1 v/v) as eluent afforded **31** in 53% yield as brown solid.

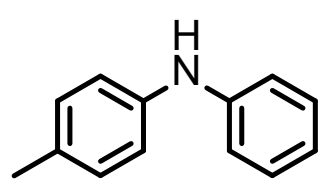

Formula: $C_{13}H_{13}N$

31, 53%

^{1}H NMR (500 MHz, $CDCl_3$) δ 7.27 – 7.23 (m, 2H), 7.10 (d, *J* = 8.3 Hz, 2H), 7.04 – 7.00 (m, 4H), 6.89 (tt, *J* = 7.3, 1.2 Hz, 1H), 5.60 (s, 1H), 2.32 (s, 3H).

^{13}C NMR (126 MHz, $CDCl_3$) δ 144.05, 140.38, 131.06, 129.99, 129.44, 120.41, 119.01, 116.96, 20.83.

Experimental data are in accordance with the literature[34].

acridin-9(10*H*)-one (32): Prepared according to the **GP5**, using the corresponding amine (0.2 mmol, 1.0 equiv.), CsOPiv (0.2 mmol, 1.0 equiv.), Cu@f-gC_3N_4 and EtOH (2 mL, 0.1 M). Purification by column chromatography on silica gel using hexane/ethyl acetate (9:1 v/v) as eluent afforded **32** in 42% yield as brown solid.

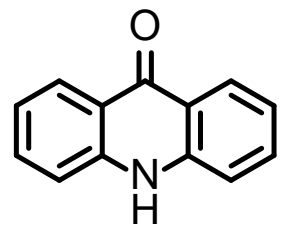

Formula: $C_{13}H_9NO$

32, 42%

^{1}H NMR (500 MHz, DMSO) δ 11.74 (s, 1H), 8.24 (dd, *J* = 8.1, 1.5 Hz, 2H), 7.72 (ddd, *J* = 8.4, 6.9, 1.6 Hz, 2H), 7.54 (dd, *J* = 8.5, 1.1 Hz, 2H), 7.25 (ddd, *J* = 8.0, 6.8, 1.1 Hz, 2H).

^{13}C NMR (126 MHz, DMSO) δ 176.79, 140.90, 133.48, 126.04, 121.02, 120.51, 117.36.

Experimental data are in accordance with the literature[36].

***N*-phenylcycloheptanamine (33):** Prepared according to the **GP5**, using the corresponding amine (0.2 mmol, 1.0 equiv.), CsOPiv (0.2 mmol, 1.0 equiv.), Cu@f-gC_3N_4 and EtOH (2 mL, 0.1 M). Purification by column chromatography on silica gel using hexane/ethyl acetate (20:1 v/v) as eluent afforded **33** in 49% yield as pale yellow oil.

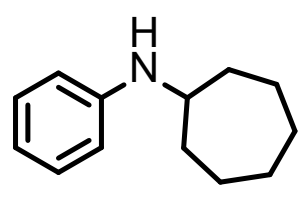

Formula: $C_{13}H_9NO$

33, 49%

^{1}H NMR (500 MHz, $CDCl_3$) δ 7.20 – 7.15 (m, 2H), 6.68 (tt, *J* = 7.3, 1.1 Hz, 1H), 6.58 – 6.55 (m, 2H), 3.60 (s, 1H), 3.47 (m, 1H), 2.05 – 1.98 (m, 2H), 1.73 – 1.62 (m, 4H), 1.61 – 1.55 (m, 2H), 1.55 – 1.50 (m, 2H), 1.50 – 1.46 (m, 2H).

^{13}C NMR (126 MHz, $CDCl_3$) δ 147.45, 129.38, 116.85, 113.31, 53.74, 34.95, 28.49, 24.53.

Experimental data are in accordance with the literature[37].

10,11-dihydro-5*H*-dibenzo[*b*,*f*]azepine (34): Prepared according to the **GP5**, using the corresponding amine (0.2 mmol, 1.0 equiv.), CsOPiv (0.2 mmol, 1.0 equiv.), Cu@f-gC_3N_4 and EtOH (2 mL, 0.1 M). Purification by column chromatography on silica gel using hexane/ethyl acetate (20:1 v/v) as eluent afforded **34** in 40% yield as brown solid.

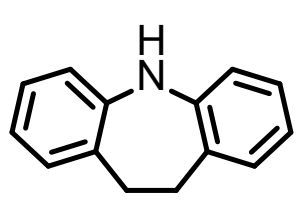

Formula: $C_{14}H_{13}N$

34, 40%

^{1}H NMR (500 MHz, $CDCl_3$) δ 7.14 – 7.06 (m, 4H), 6.81 (td, *J* = 7.4, 1.2 Hz, 2H), 6.76 (dd, *J* = 7.9, 1.2 Hz, 2H), 6.01 (s, 1H), 3.11 (s, 4H).

^{13}C NMR (126 MHz, $CDCl_3$) δ 142.57, 130.81, 128.77, 126.95, 119.57, 118.05, 35.05.

Experimental data are in accordance with the literature[38].

2,4-dinitro-*N*-phenylaniline (35): Prepared according to the **GP5**, using the corresponding amine (0.2 mmol, 1.0 equiv.), CsOPiv (0.2 mmol, 1.0 equiv.), Cu@f-gC_3N_4 and EtOH (2 mL, 0.1 M). Purification by column chromatography on silica gel using hexane/ethyl acetate (9:1 v/v) as eluent afforded **35** in 45% yield as brown solid.

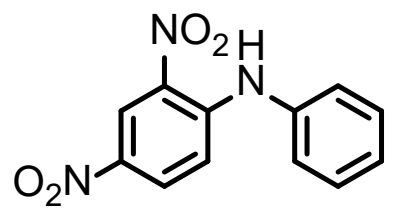

Formula: $C_{12}H_9N_3O_4$

35, 45%

^{1}H NMR (500 MHz, $CDCl_3$) δ 9.99 (s, 1H), 9.19 (d, *J* = 2.7 Hz, 1H), 8.17 (ddd, *J* = 9.5, 2.7, 0.7 Hz, 1H), 7.54 – 7.49 (m, 2H), 7.41 – 7.37 (m, 1H), 7.33 – 7.30 (m, 2H), 7.17 (d, *J* = 9.5 Hz, 1H).

^{13}C NMR (126 MHz, $CDCl_3$) δ 147.25, 137.54, 136.80, 131.23, 130.40, 130.07, 127.89, 125.66, 124.25, 116.17.

Experimental data are in accordance with the literature[39].

***N*-phenylnaphthalen-1-amine (36):** Prepared according to the **GP5**, using the corresponding amine (0.2 mmol, 1.0 equiv.), CsOPiv (0.2 mmol, 1.0 equiv.), Cu@f-gC_3N_4 and EtOH (2 mL, 0.1 M). Purification by column chromatography on silica gel using hexane/ethyl acetate (20:1 v/v) as eluent afforded **36** in 65% yield as grey solid.

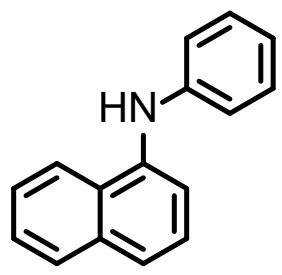

Formula: $C_{16}H_{13}N$

36, 65%

^{1}H NMR (500 MHz, $CDCl_3$) δ 8.06 – 8.02 (m, 1H), 7.92 – 7.86 (m, 1H), 7.59 (dt, *J* = 7.6, 1.2 Hz, 1H), 7.50 (dddd, *J* = 14.9, 8.2, 6.8, 1.5 Hz, 2H), 7.44 – 7.37 (m, 2H), 7.30 – 7.26 (m, 2H), 7.04 – 6.98 (m, 2H), 6.93 (tt, *J* = 7.3, 1.1 Hz, 1H), 5.95 (s, 1H).

^{13}C NMR (126 MHz, $CDCl_3$) δ 144.88, 138.87, 134.82, 129.48, 128.68, 127.85, 126.25, 126.16, 125.82, 123.10, 121.93, 120.61, 117.50, 115.97.

Experimental data are in accordance with the literature[40].

9*H*-pyrido[3,4-*b*]indole (37): Prepared according to the **GP5**, using the corresponding amine (0.2 mmol, 1.0 equiv.), CsOPiv (0.2 mmol, 1.0 equiv.), Cu@f-gC_3N_4 and EtOH (2 mL, 0.1 M). Purification by column chromatography on silica gel using hexane/ethyl acetate (4:1 v/v) as eluent afforded **37** in 42% yield as grey solid.

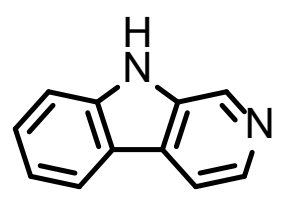

Formula: $C_{11}H_8N_2$

37, 42%

^{1}H NMR (500 MHz, $CDCl_3$) δ 9.18 (s, 1H), 8.98 – 8.90 (m, 1H), 8.49 (d, *J* = 5.3 Hz, 1H), 8.15 (dt, *J* = 7.9, 1.0 Hz, 1H), 7.99 (dd, *J* = 5.3, 1.0 Hz, 1H), 7.60 – 7.51 (m, 2H), 7.30 (ddd, *J* = 8.0, 6.5, 1.5 Hz, 1H).

^{13}C NMR (126 MHz, $CDCl_3$) δ 140.70, 139.09, 136.15, 133.74, 129.14, 128.69, 121.95, 121.57, 120.24, 114.96, 111.78.

Experimental data are in accordance with the literature[41].

(5*S*,8*R*,9*S*,10*S*,13*S*,14*S*,17*S*)-10,13-dimethyl-3-(phenylamino)hexadecahydro-1*H*-cyclopenta[a]phenanthren-17-ol (38): Prepared according to the **GP5**, using the corresponding amine (0.2 mmol, 1.0 equiv.), CsOPiv (0.2 mmol, 1.0 equiv.), Cu@f-gC_3N_4 and EtOH (2 mL, 0.1 M). Purification by column chromatography on silica gel using hexane/ethyl acetate (9:1 v/v) as eluent afforded **38** in 42% yield as white solid.

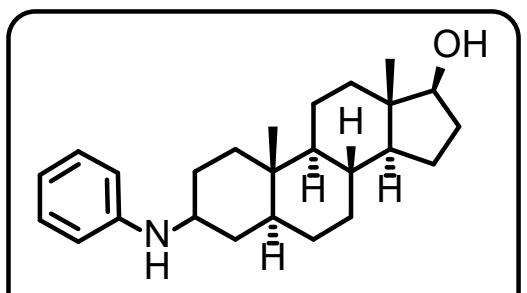

Formula: $C_{11}H_8N_2$

38, 42%

^{1}H NMR (500 MHz, CDCl$_3$) δ 7.20 – 7.13 (m, 2H), 6.66 (tt, *J* = 7.3, 1.1 Hz, 1H), 6.62 – 6.57 (m, 2H), 3.89 (s, 1H), 3.72 – 3.59 (m, 2H), 2.11 – 2.01 (m, 1H), 1.80 (m, 1H), 1.77 – 1.71 (m, 2H), 1.68 (m, 1H), 1.63 – 1.52 (m, 4H), 1.50 (m, 1H), 1.46 – 1.32 (m, 4H), 1.32 – 1.14 (m, 6H), 1.11 – 1.01 (m, 1H), 1.00 – 0.87 (m, 2H), 0.85 (s, 3H), 0.74 (s, 3H).

^{13}C NMR (126 MHz, CDCl$_3$) δ 147.47, 129.39, 116.68, 112.95, 82.05, 54.71, 51.18, 47.40, 43.09, 40.50, 36.85, 36.28, 35.61, 33.09, 32.71, 31.66, 30.60, 28.54, 25.96, 23.45, 20.49, 11.65, 11.27.

HRMS (ESI): $[M+H]^+$ calcd. 368.2948, found 368.2938.

2-((2*R*,6*S*)-6-((*S*)-2-hydroxy-2-phenylethyl)piperidin-2-yl)-1-phenylethan-1-one (39): Prepared according to the **GP5**, using the corresponding amine (0.2 mmol, 1.0 equiv.), CsOPiv (0.2 mmol, 1.0 equiv.), Cu@f-gC_3N_4 and EtOH (2 mL, 0.1 M). Purification by column chromatography on silica gel using hexane/ethyl acetate (9:1 v/v) as eluent afforded **39** in 55% yield as yellow solid.

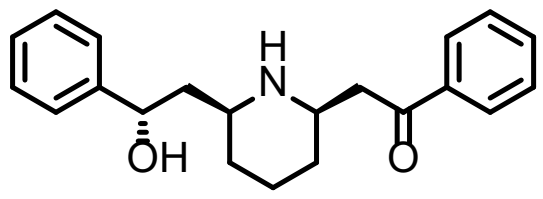

Formula: $C_{21}H_{25}NO_2$

39, 55%

^{1}H NMR (500 MHz, CDCl$_3$) δ 8.00 – 7.94 (m, 2H), 7.59 – 7.55 (m, 1H), 7.49 – 7.44 (m, 2H), 7.37 – 7.29 (m, 4H), 7.26 – 7.22 (m, 1H), 4.61 (d, *J* = 8.2 Hz, 1H), 4.47 (dd, *J* = 11.5, 2.6 Hz, 1H), 3.81 (d, *J* = 8.2 Hz, 1H), 3.52 – 3.43 (m, 1H), 2.99 – 2.87 (m, 2H), 2.40 (tt, *J* = 10.8, 2.8 Hz, 1H), 1.82 (dt, *J* = 13.4, 2.8 Hz, 1H), 1.77 (m, 2H), 1.74 – 1.67 (m, 1H), 1.64 (m, 1H), 1.58 – 1.52 (m, 1H), 1.52 – 1.45 (m, 1H), 1.44 – 1.37 (m, 1H).

^{13}C NMR (126 MHz, CDCl$_3$) δ 198.72, 141.85, 136.89, 133.51, 128.86, 128.45, 128.27, 127.67, 125.89, 84.82, 79.99, 61.36, 53.77, 43.73, 41.13, 33.50, 32.72, 24.06.

HRMS (ESI): $[M+H]^+$ calcd. 324.1958, found 324.1950.

***N*-benzyl-*N*-cyclohexylcyclohexanamine (45):** Prepared according to the **GP5**. Upon completion, the reaction mixture was filtered through a 0.2 μm PTFE syringe filter. The filtrate was concentrated under reduced pressure. The resulting residue was redissolved in dry DMF (2.0 mL), followed by the addition of K_2CO_3 (0.4 mmol, 2.0 equiv.) and benzyl bromide (0.3 mmol, 1.5 equiv.). The mixture was stirred at room temperature overnight. Purification by column chromatography on silica gel using hexane/ethyl acetate (20:1 v/v) as eluent afforded **45** in 68% yield as colorless oil.

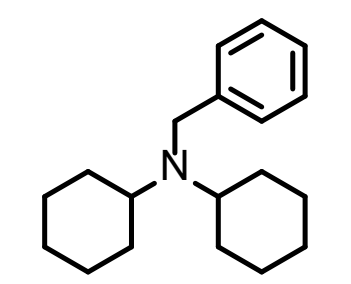
Formula: $C_{19}H_{29}N$
45, 68%

^{1}H NMR (500 MHz, CDCl$_3$) δ 7.40 – 7.37 (m, 2H), 7.29 (dd, *J* = 8.4, 6.8 Hz, 2H), 7.22 – 7.18 (m, 1H), 3.76 (s, 2H), 2.54 (tt, *J* = 11.4, 3.4 Hz, 2H), 1.82 – 1.70 (m, 8H), 1.58 (m, 2H), 1.32 – 1.24 (m, 4H), 1.24 – 1.14 (m, 4H), 1.04 (qt, *J* = 12.6, 3.5 Hz, 2H).
^{13}C NMR (126 MHz, CDCl$_3$) δ 142.37, 126.51, 126.32, 124.61, 56.29, 48.61, 30.58, 25.10, 24.93.
HRMS (ESI): $[M+H]^+$ calcd. 272.2373, found 272.2365.

***N*-allyl-*N*-cyclohexylcyclohexanamine (46):** Prepared according to the **GP5**. Upon completion, the reaction mixture was filtered through a 0.2 μm PTFE syringe filter. The filtrate was concentrated under reduced pressure. The resulting residue was redissolved in dry DMF (2.0 mL), followed by the addition of K_2CO_3 (0.4 mmol, 2.0 equiv.) and allyl bromide (0.3 mmol, 1.5 equiv.). The mixture was stirred at room temperature overnight. Purification by column chromatography on silica gel using hexane/ethyl acetate (20:1 v/v) as eluent afforded **46** in 75% yield as colorless oil.

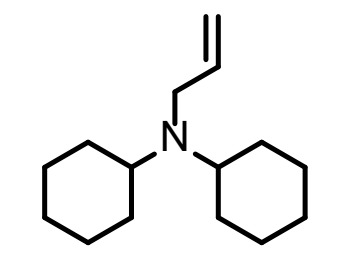
Formula: $C_{15}H_{27}N$
46, 75%

^{1}H NMR (500 MHz, CDCl$_3$) δ 5.81 (ddt, *J* = 17.2, 10.1, 5.9 Hz, 1H), 5.13 (dq, *J* = 17.2, 1.8 Hz, 1H), 4.97 (dq, *J* = 10.1, 1.7 Hz, 1H), 3.19 (dt, *J* = 5.9, 1.6 Hz, 2H), 2.54 (m, 2H), 1.80 – 1.64 (m, 8H), 1.58 (m, 2H), 1.28 – 1.16 (m, 8H), 1.05 (m, 2H).
^{13}C NMR (126 MHz, CDCl$_3$) δ 140.67, 114.47, 57.90, 49.44, 31.92, 26.59, 26.47.
HRMS (ESI): $[M+H]^+$ calcd. 222.2216, found 222.2210.

***N*-cyclohexyl-*N*-(prop-2-yn-1-yl)cyclohexanamine (47):** Prepared according to the **GP5**. Upon completion, the reaction mixture was filtered through a 0.2 μm PTFE syringe filter. The filtrate was concentrated under reduced pressure. The resulting residue was redissolved in dry DMF (2.0 mL), followed by the addition of K_2CO_3 (0.4 mmol, 2.0 equiv.) and propargyl

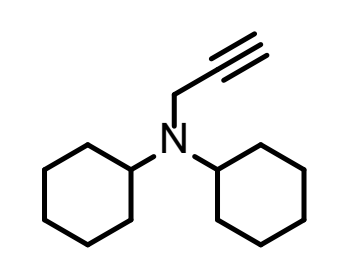
Formula: $C_{15}H_{25}N$
47, 72%

bromide (0.3 mmol, 1.5 equiv.). The mixture was stirred at room temperature overnight. Purification by column chromatography on silica gel using hexane/ethyl acetate (20:1 v/v) as eluent afforded **47** in 75% yield as grey solid.

^{1}H NMR (500 MHz, $CDCl_3$) δ 3.46 (d, *J* = 2.4 Hz, 2H), 2.75 (tt, *J* = 10.9, 3.3 Hz, 2H), 2.12 (t, *J* = 2.4 Hz, 1H), 1.86 – 1.74 (m, 8H), 1.60 (dqd, *J* = 12.3, 3.2, 1.5 Hz, 2H), 1.26 (dddd, *J* = 25.3, 15.6, 12.5, 9.3 Hz, 8H), 1.10 (qt, *J* = 12.2, 3.5 Hz, 2H).
^{13}C NMR (126 MHz, $CDCl_3$) δ 84.06, 71.04, 57.40, 34.95, 31.33, 26.40, 26.28.
HRMS (ESI): $[M+H]^+$ calcd. 220.2060, found 220.2054.

6 NMR spectra

dicyclohexylamine (2)

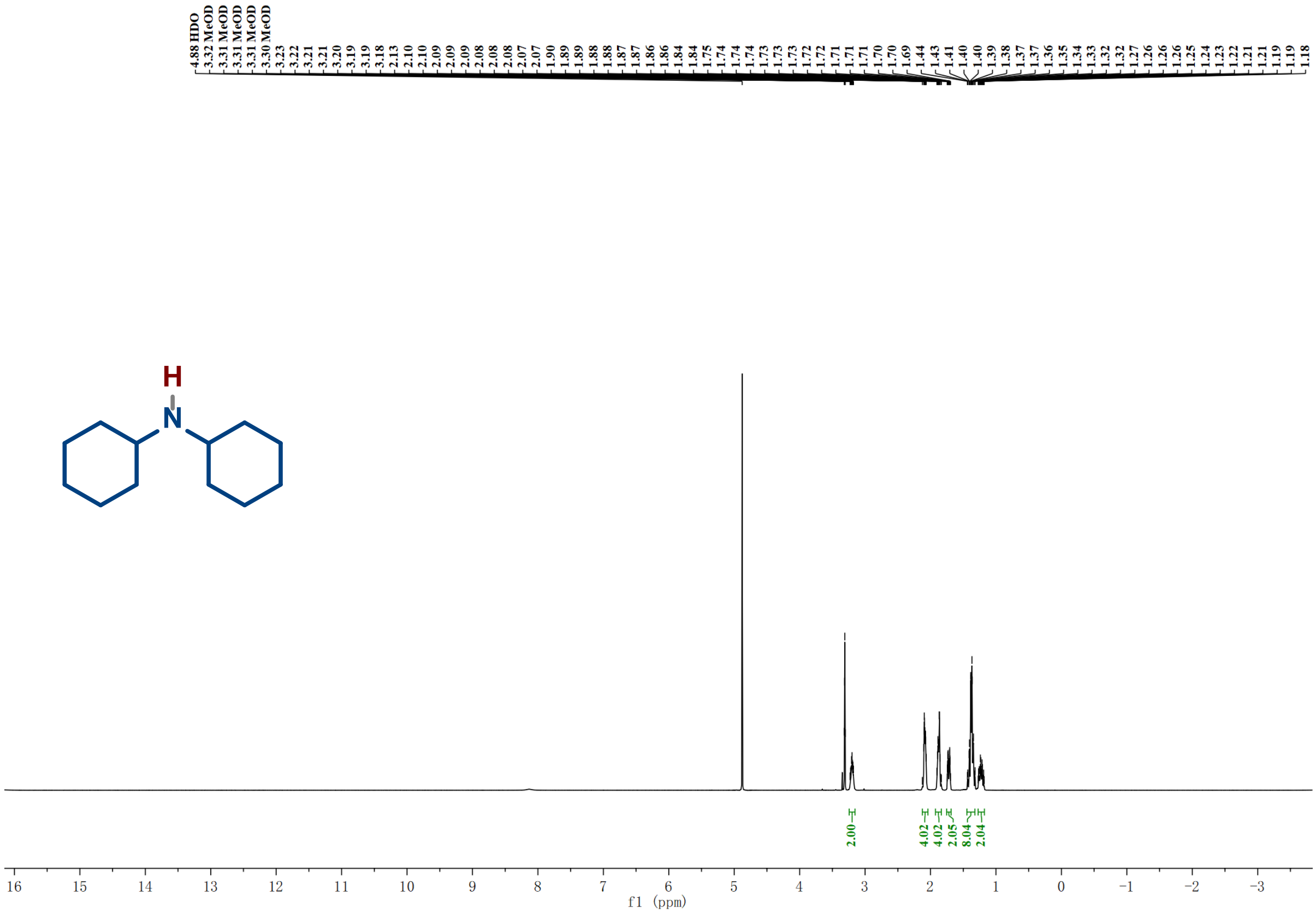

^{1}H NMR (500 MHz, MeOD)

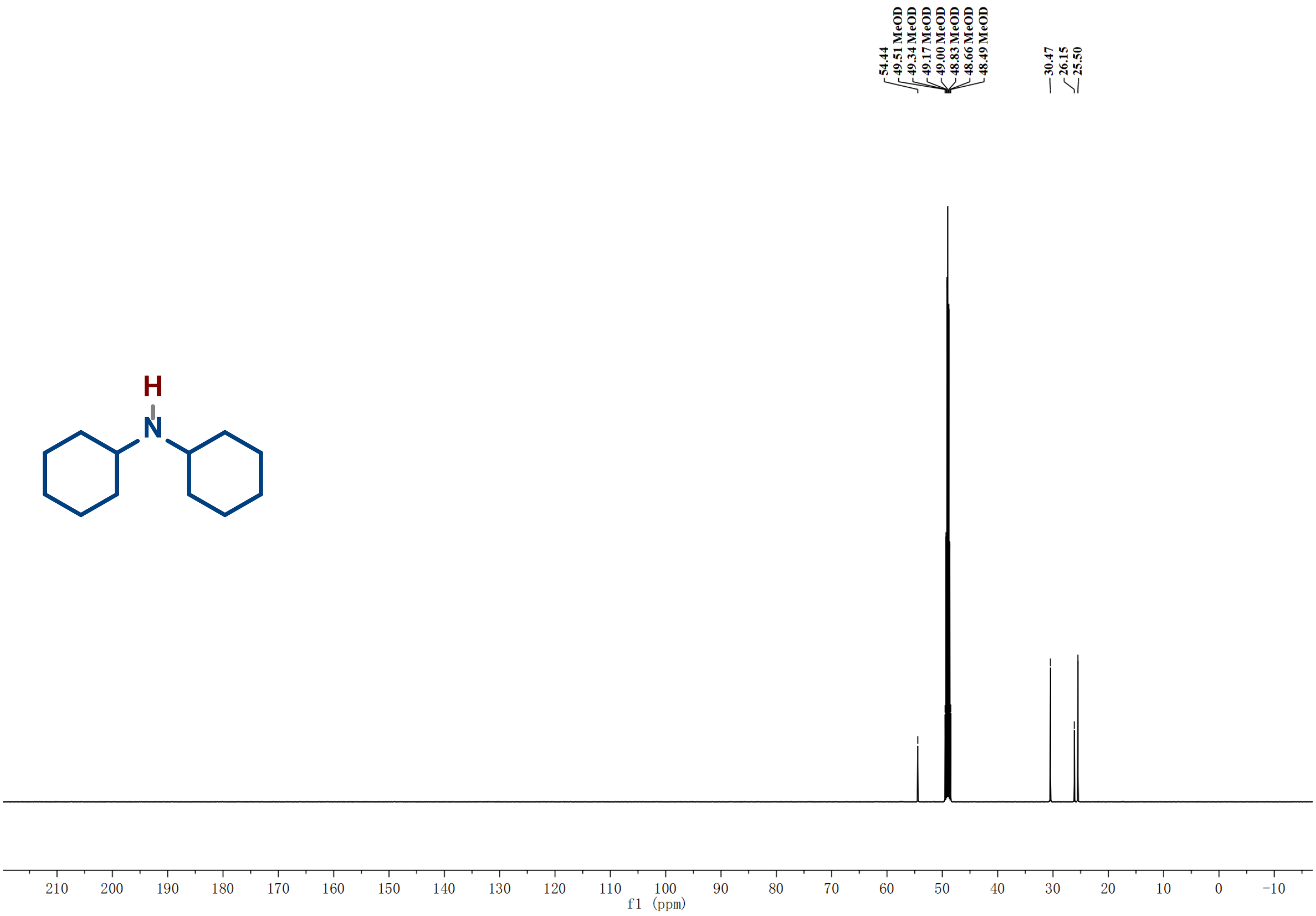

^{13}C NMR (126 MHz, MeOD)

***N*-cyclohexyltetrahydro-2*H*-pyran-4-amine (3)**

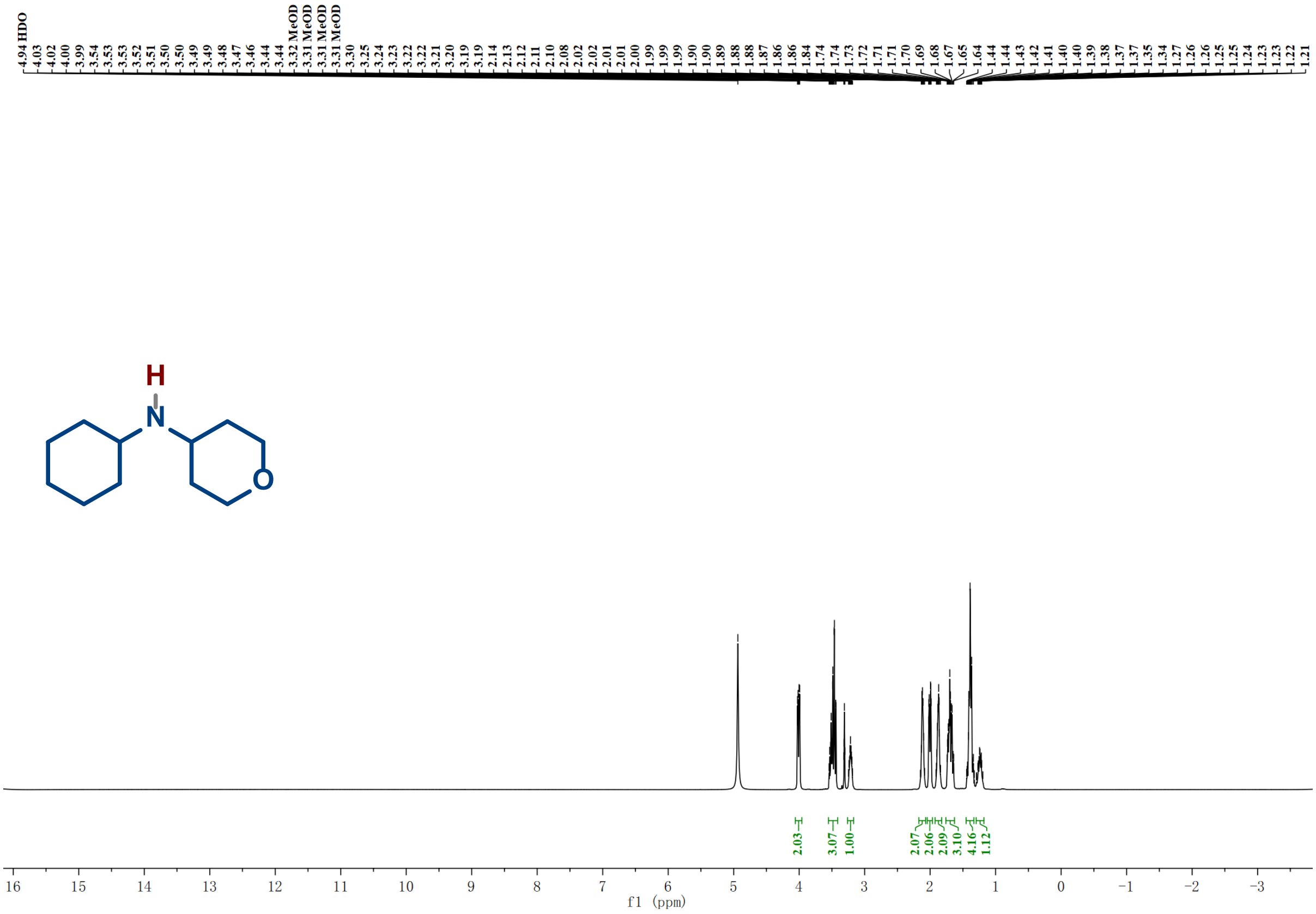

^{1}H NMR (500 MHz, MeOD)

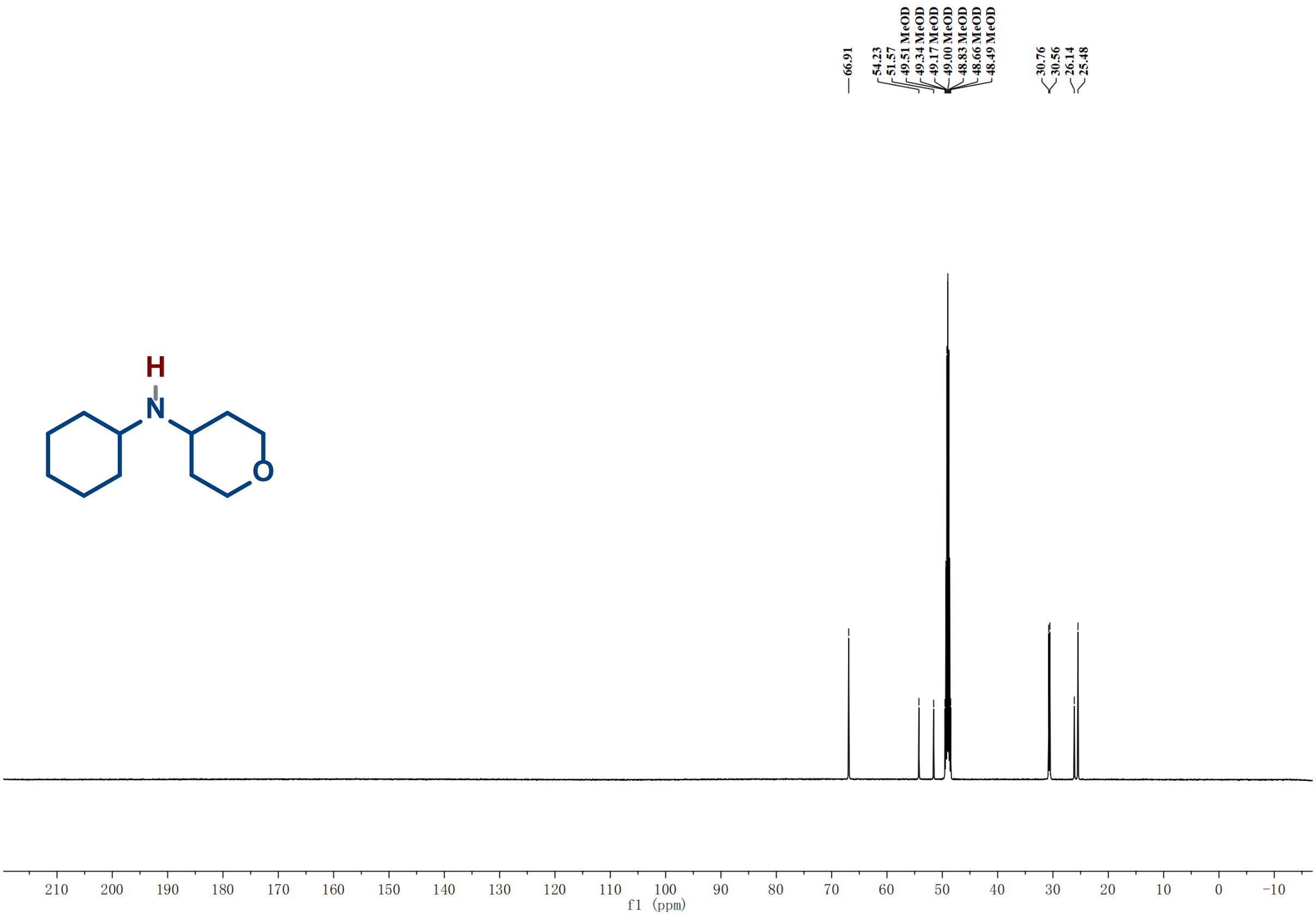

^{13}C NMR (126 MHz, MeOD)

***N*-cyclohexyl-1,4-dioxaspiro[4.5]decan-8-amine (4)**

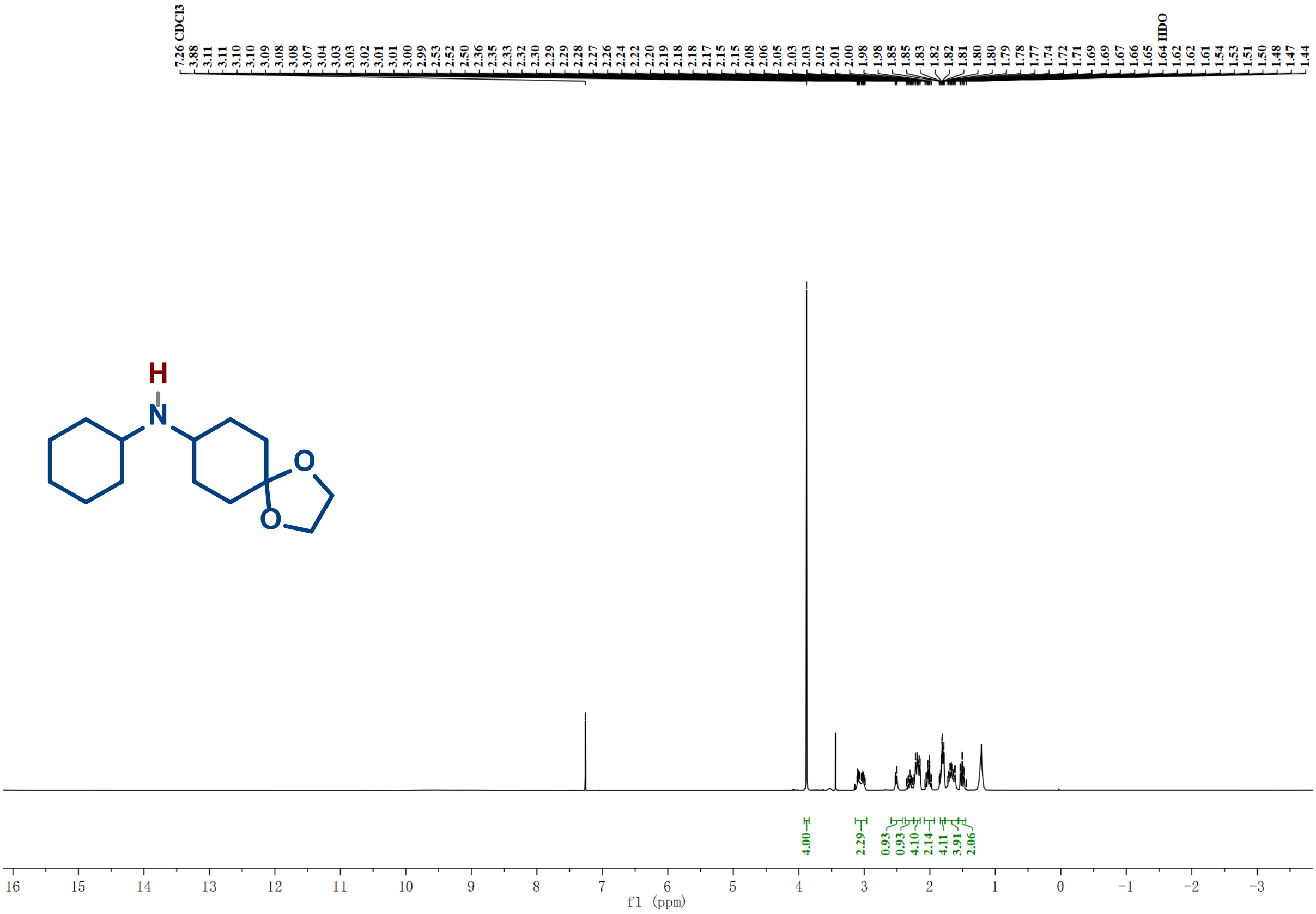

^{1}H NMR (500 MHz, $CDCl_3$)

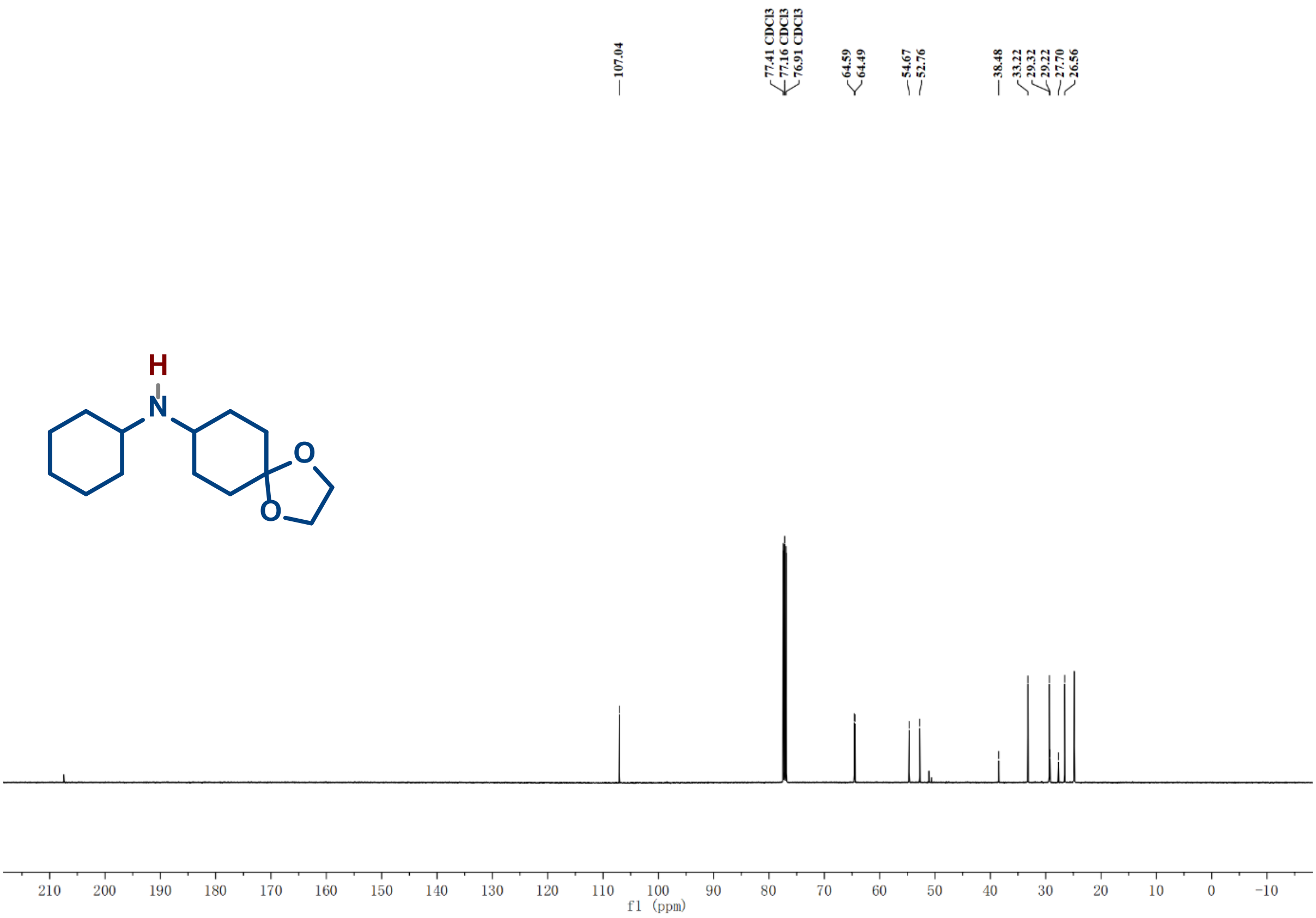

^{13}C NMR (126 MHz, $CDCl_3$)

N-cyclopentylcyclohexanamine (5)

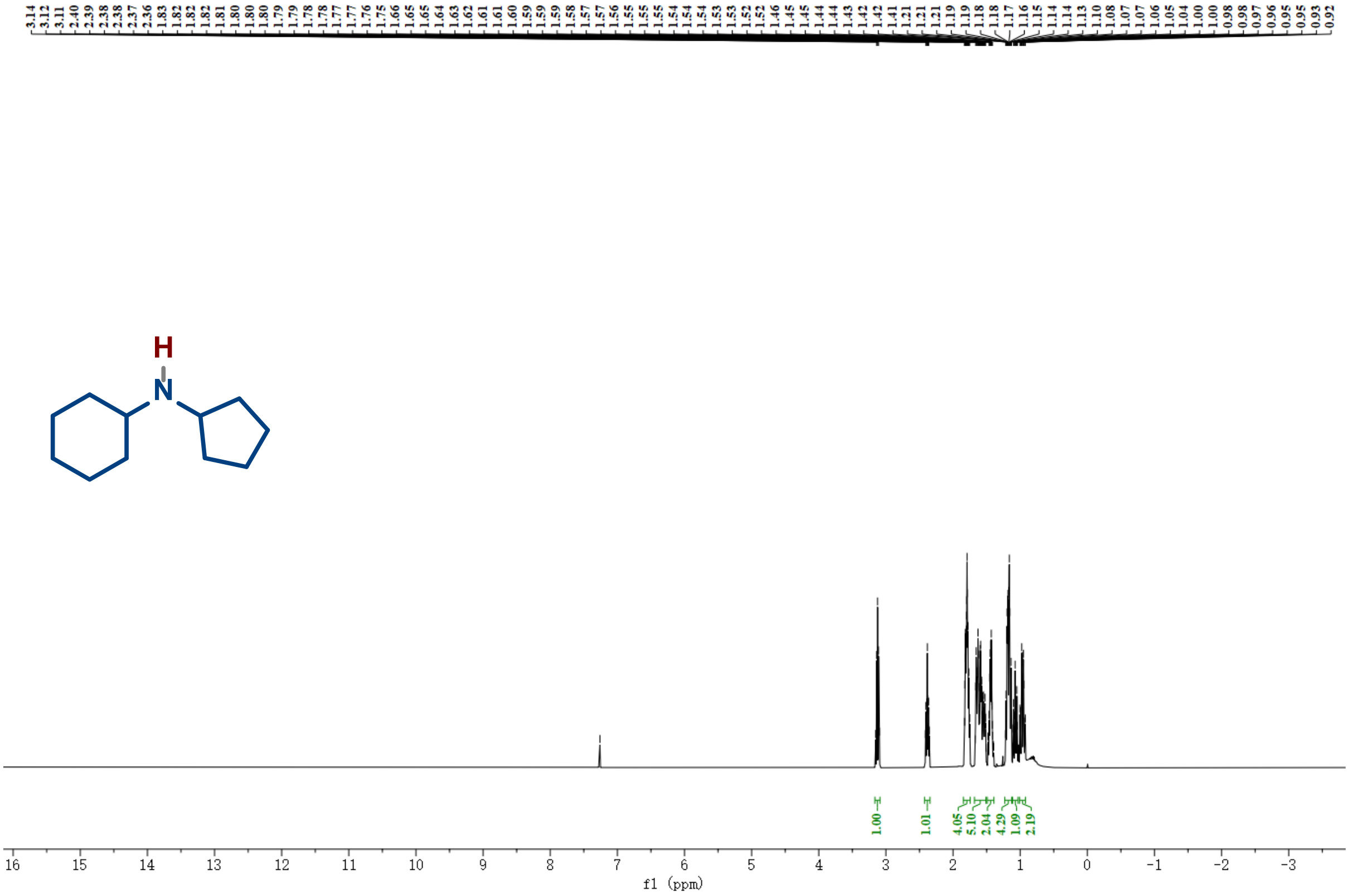

^{1}H NMR (500 MHz, CDCl$_3$)

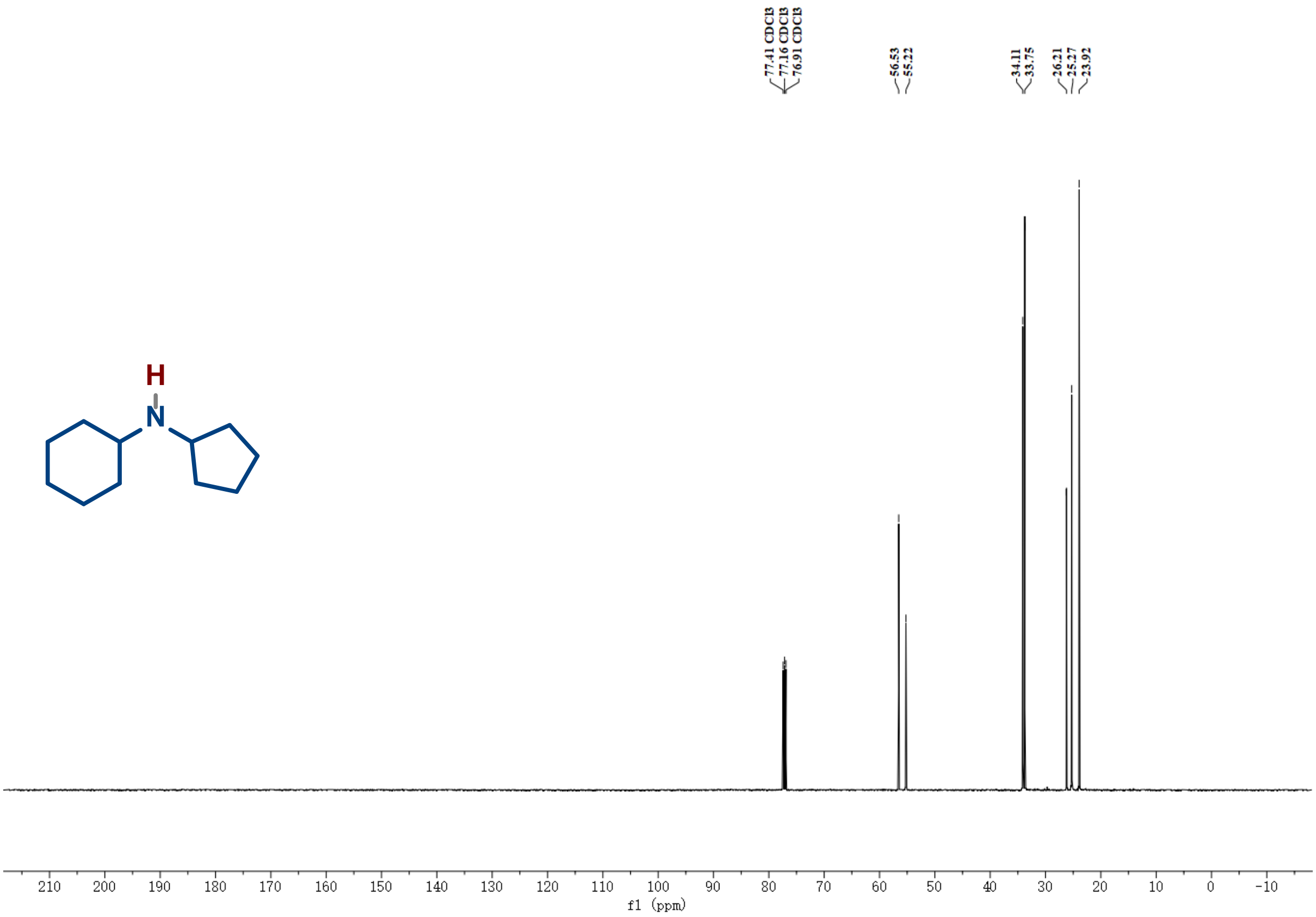

^{13}C NMR (126 MHz, CDCl$_3$)

dicyclopentylamine (6)

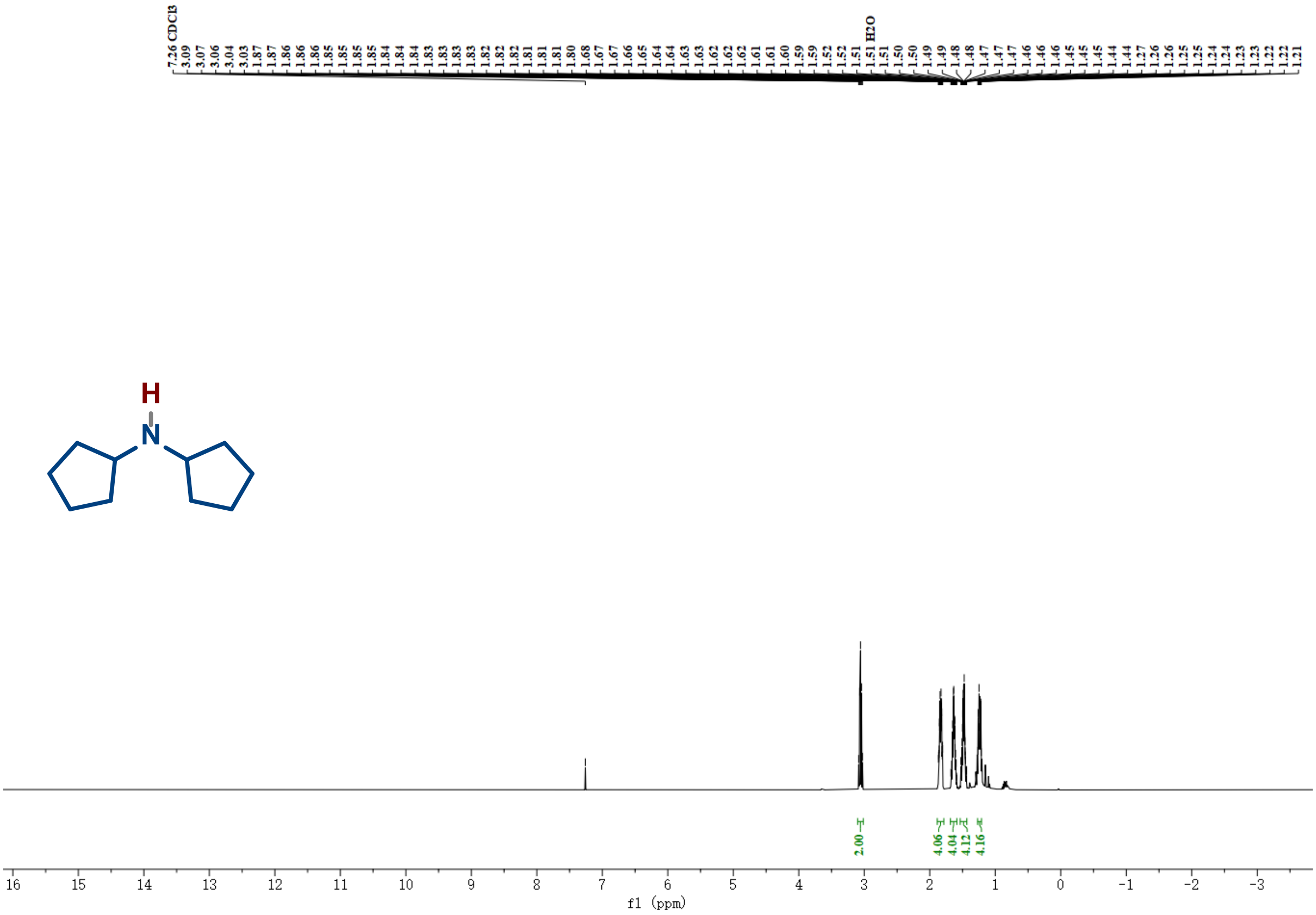

^{1}H NMR (500 MHz, $CDCl_3$)

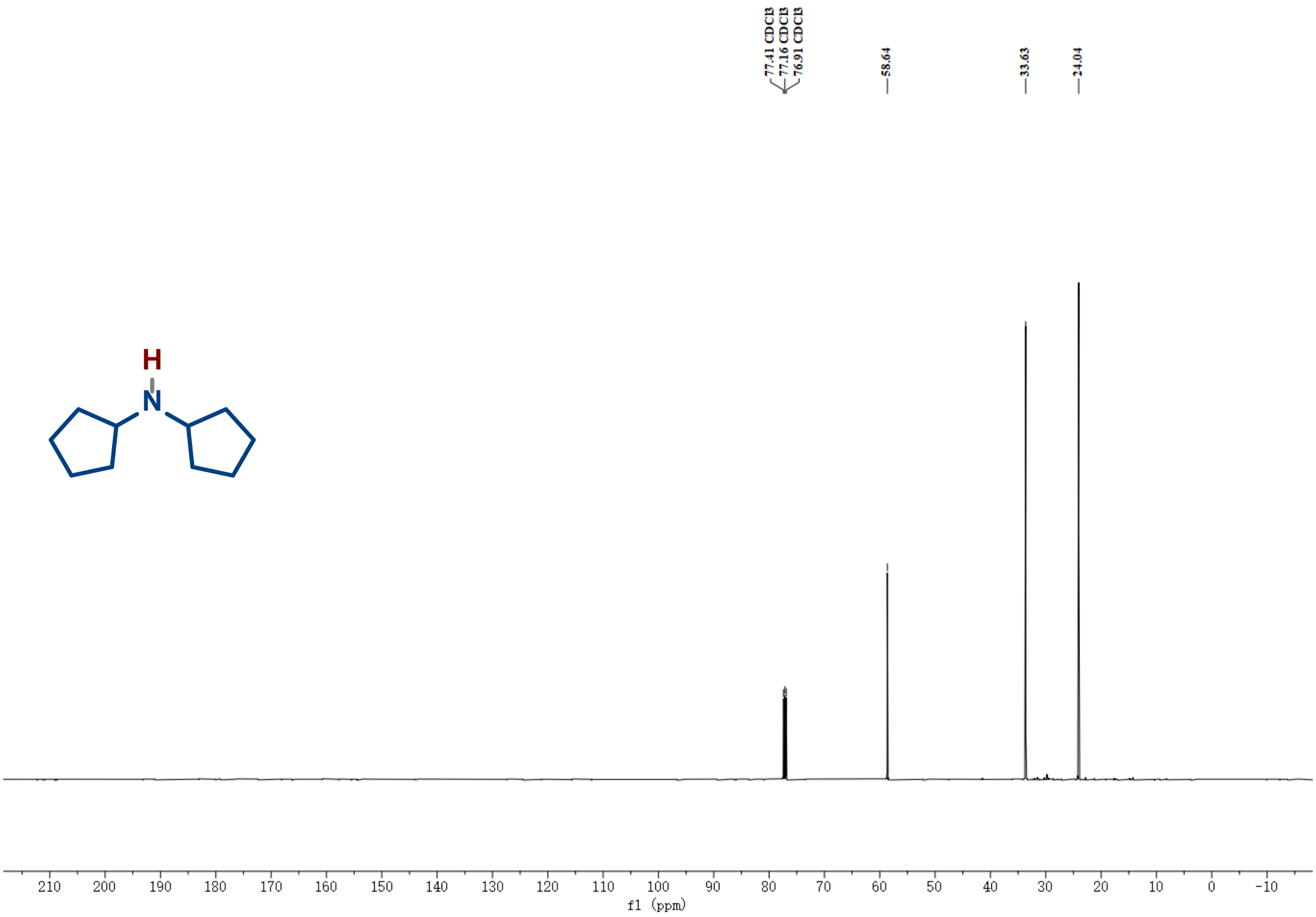

^{13}C NMR (126 MHz, $CDCl_3$)

N-cyclohexylcyclododecanamine (7)

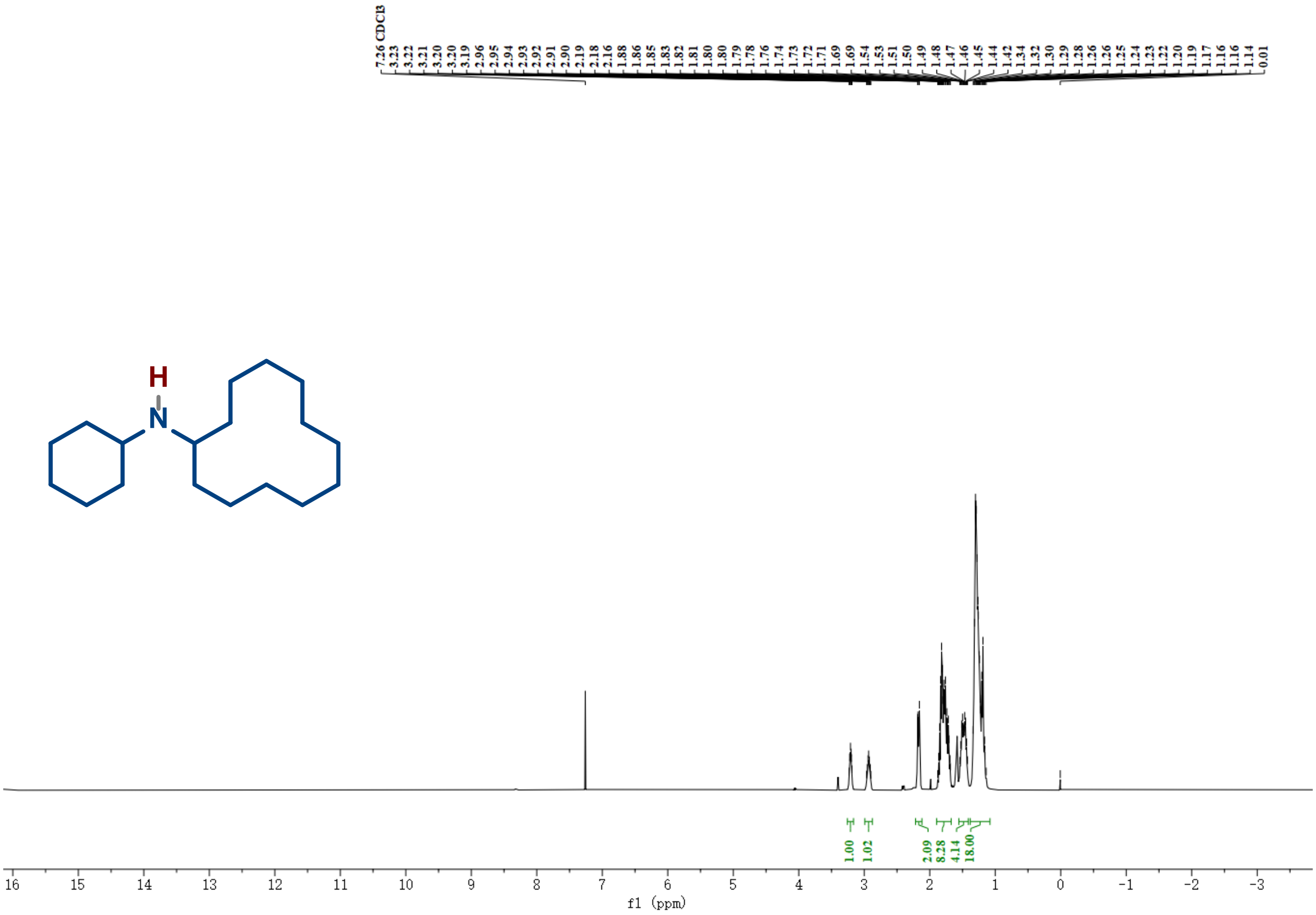

^{1}H NMR (500 MHz, CDCl$_3$)

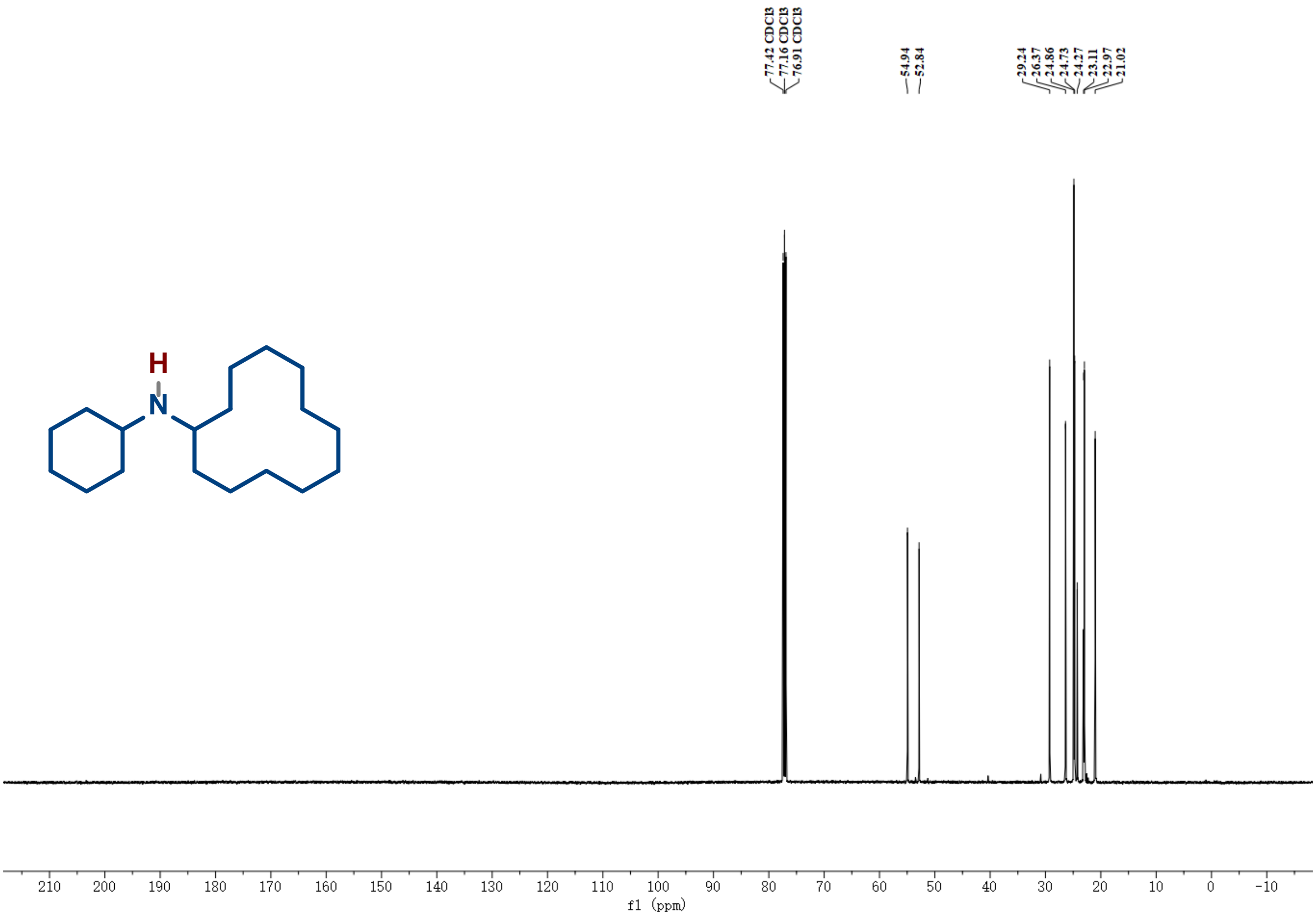

^{13}C NMR (126 MHz, CDCl$_3$)

***N*-cyclohexylbicyclo[2.2.1]heptan-2-amine (8)**

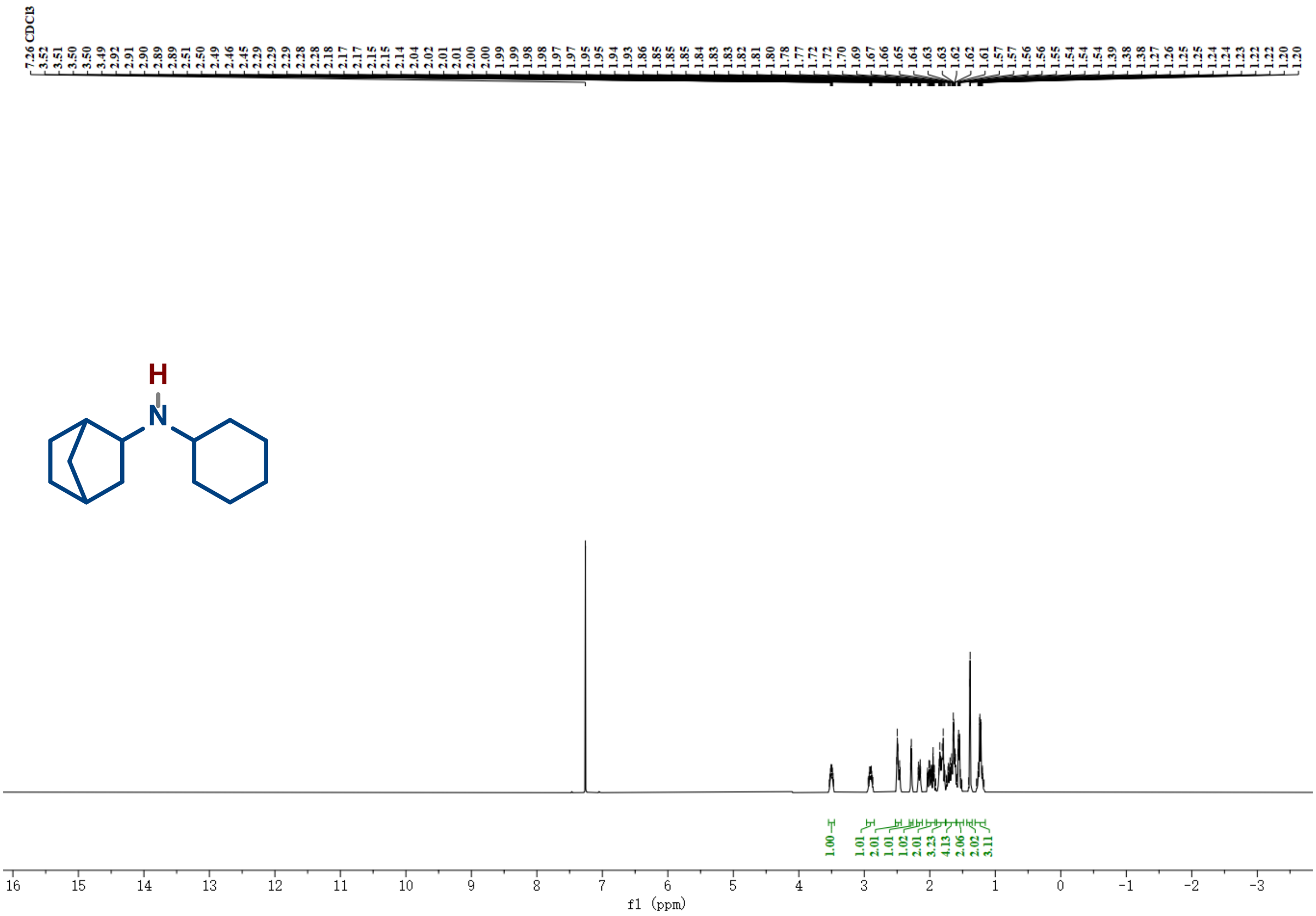

^{1}H NMR (500 MHz, CDCl$_3$)

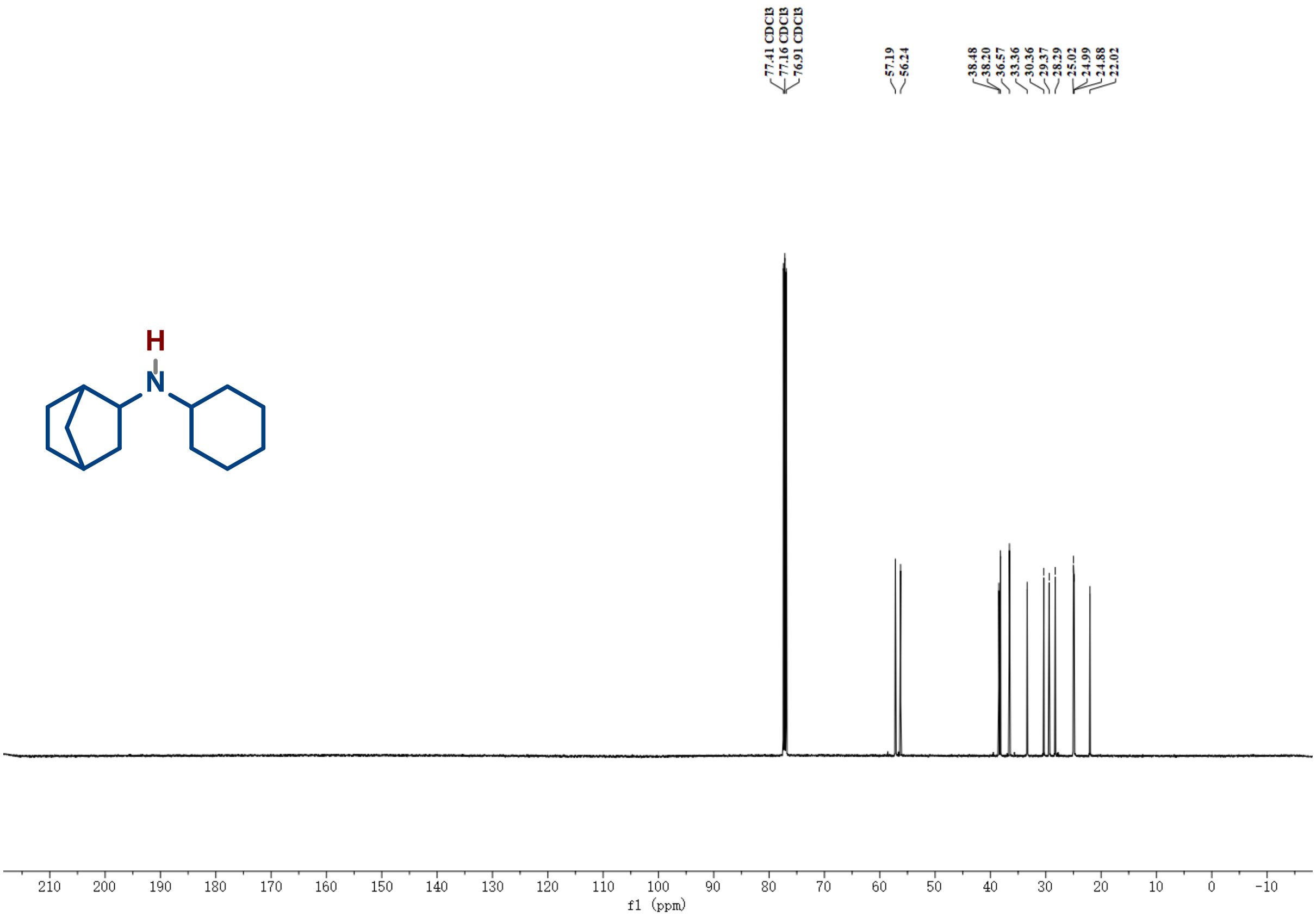

^{13}C NMR (126 MHz, CDCl$_3$)

azepan-2-one (9)

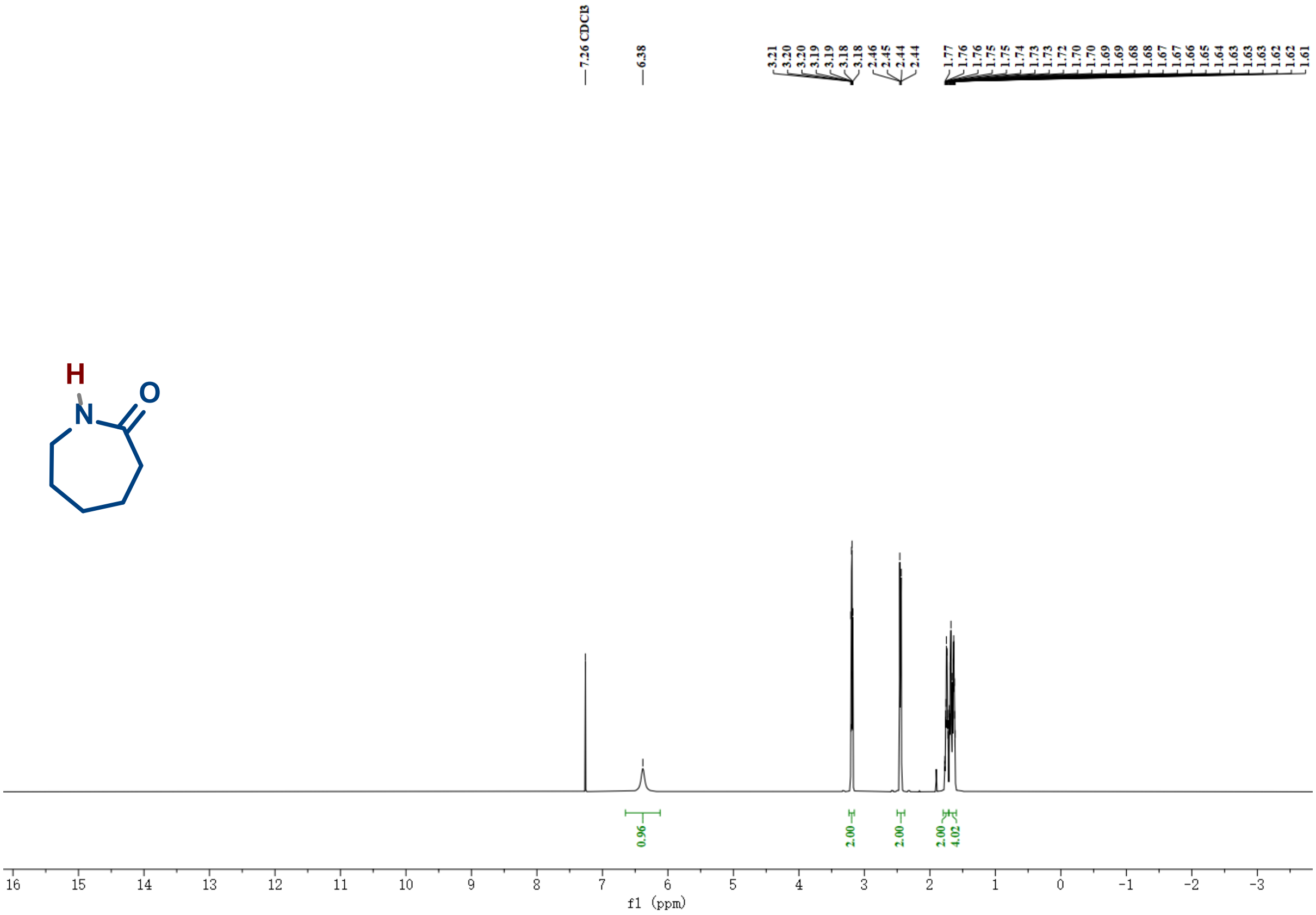

^{1}H NMR (500 MHz, CDCl$_3$)

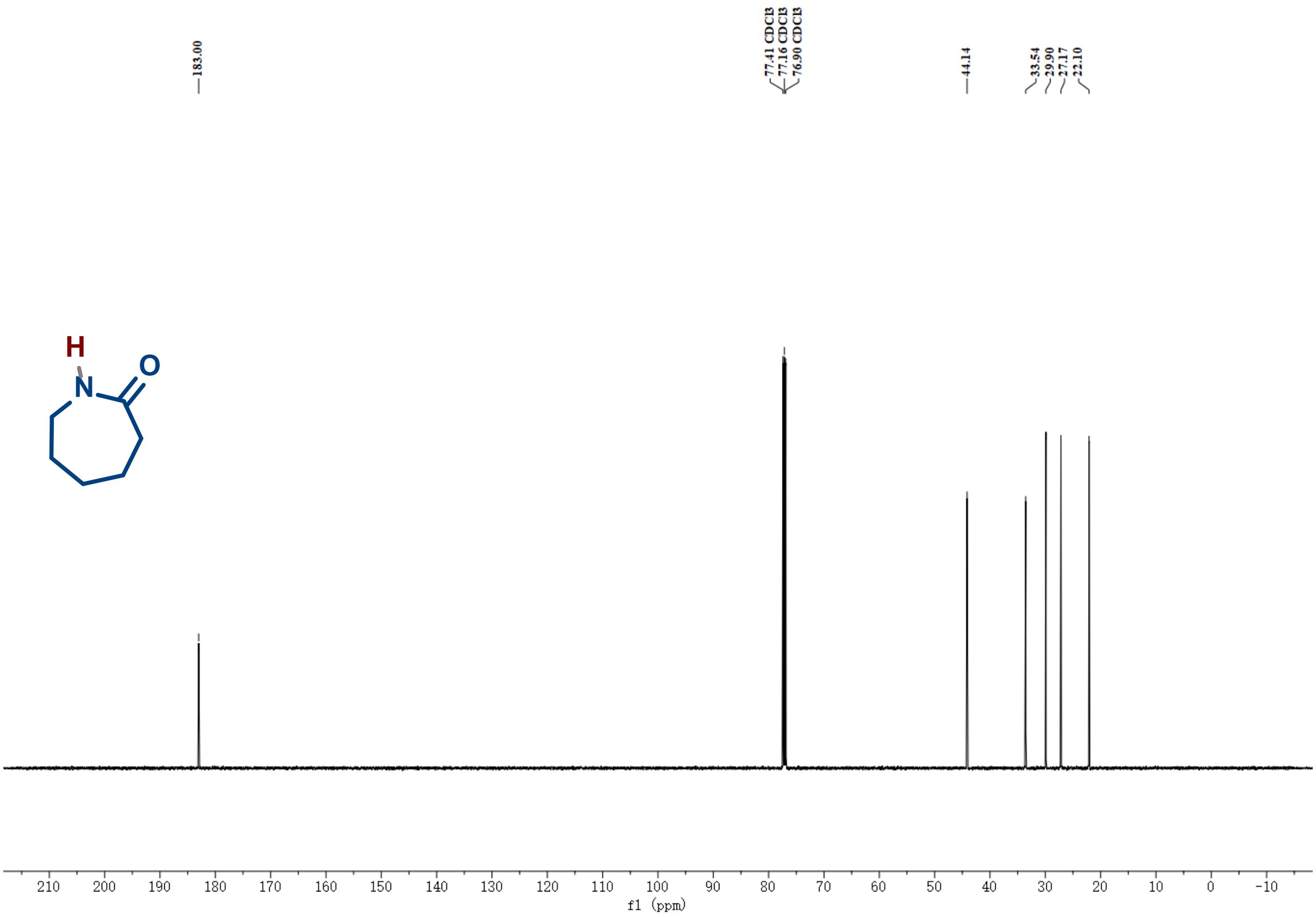

^{13}C NMR (126 MHz, CDCl$_3$)

2,2,6,6-tetramethylpiperidine (10)

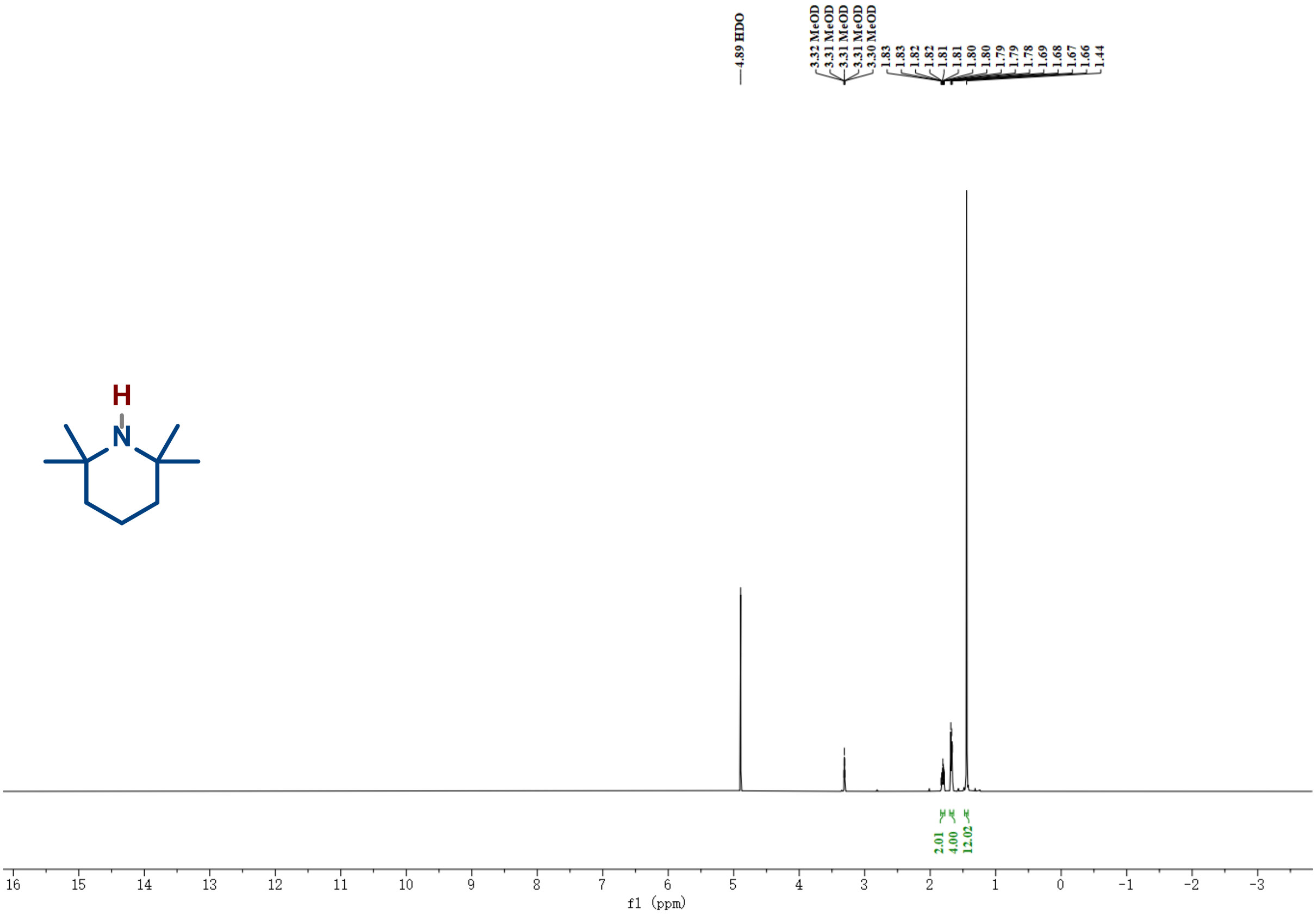

^{1}H NMR (500 MHz, MeOD)

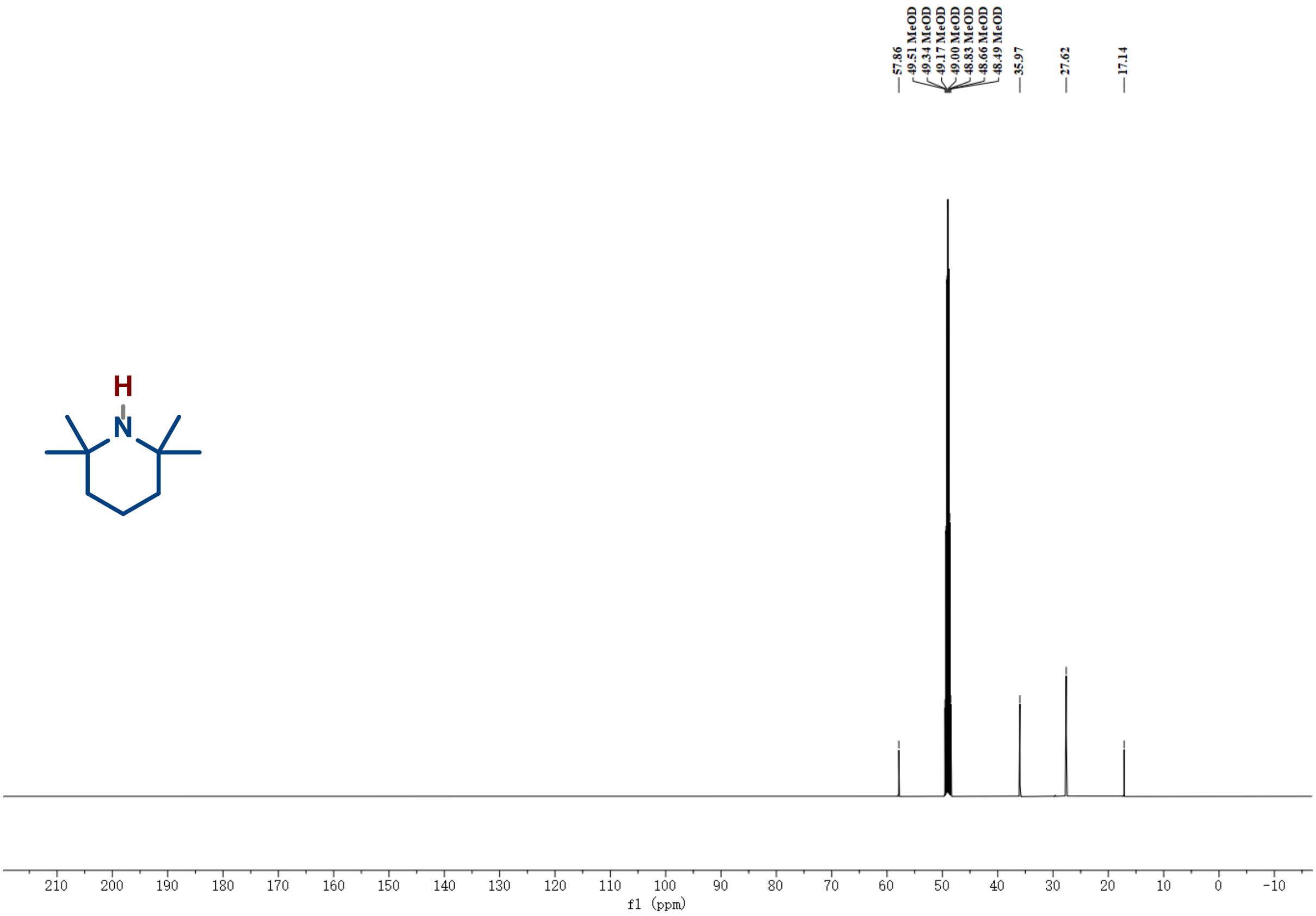

^{13}C NMR (126 MHz, MeOD)

2,2,6,6-tetramethylpiperidin-4-one (11)

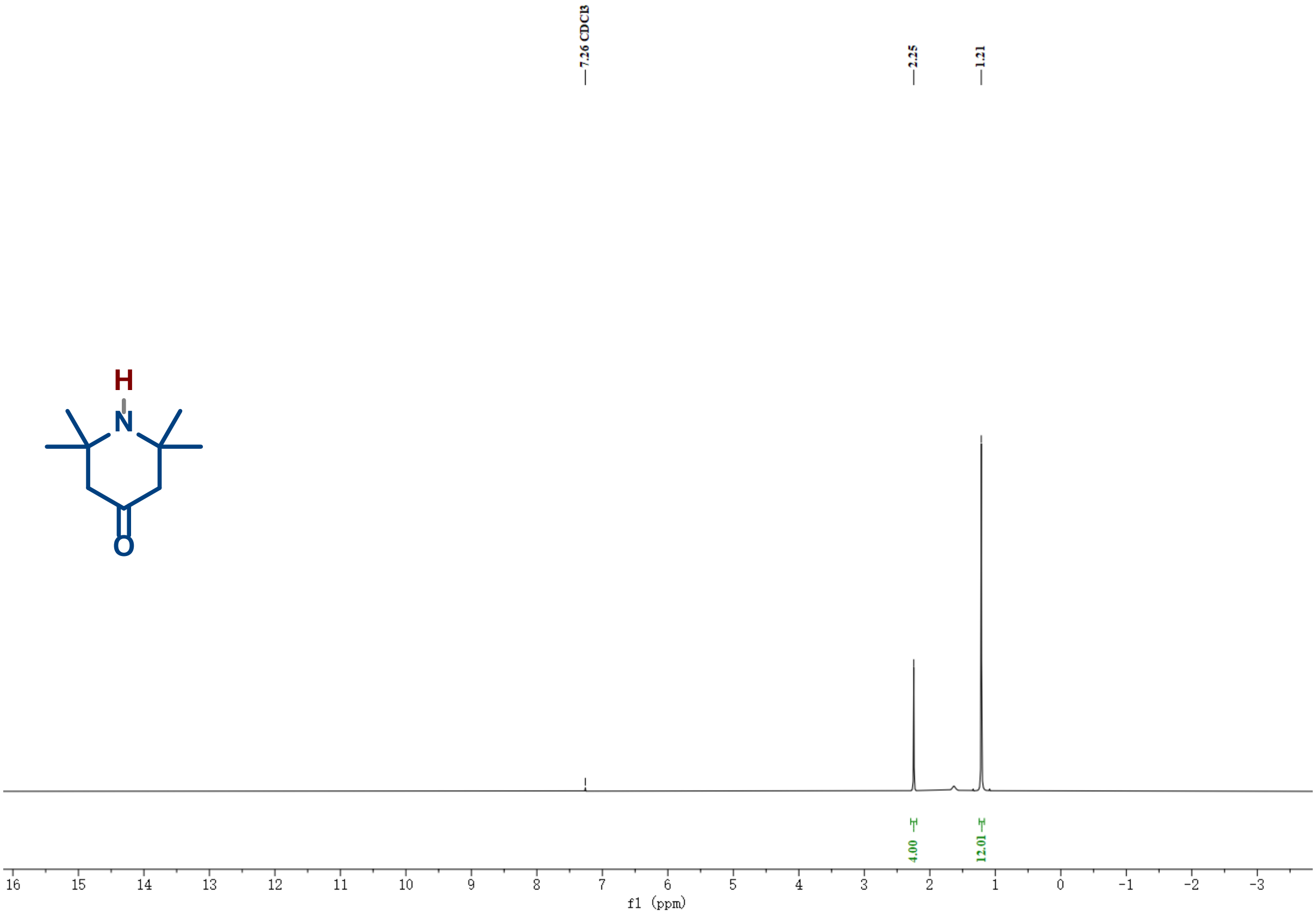

^{1}H NMR (500 MHz, CDCl$_3$)

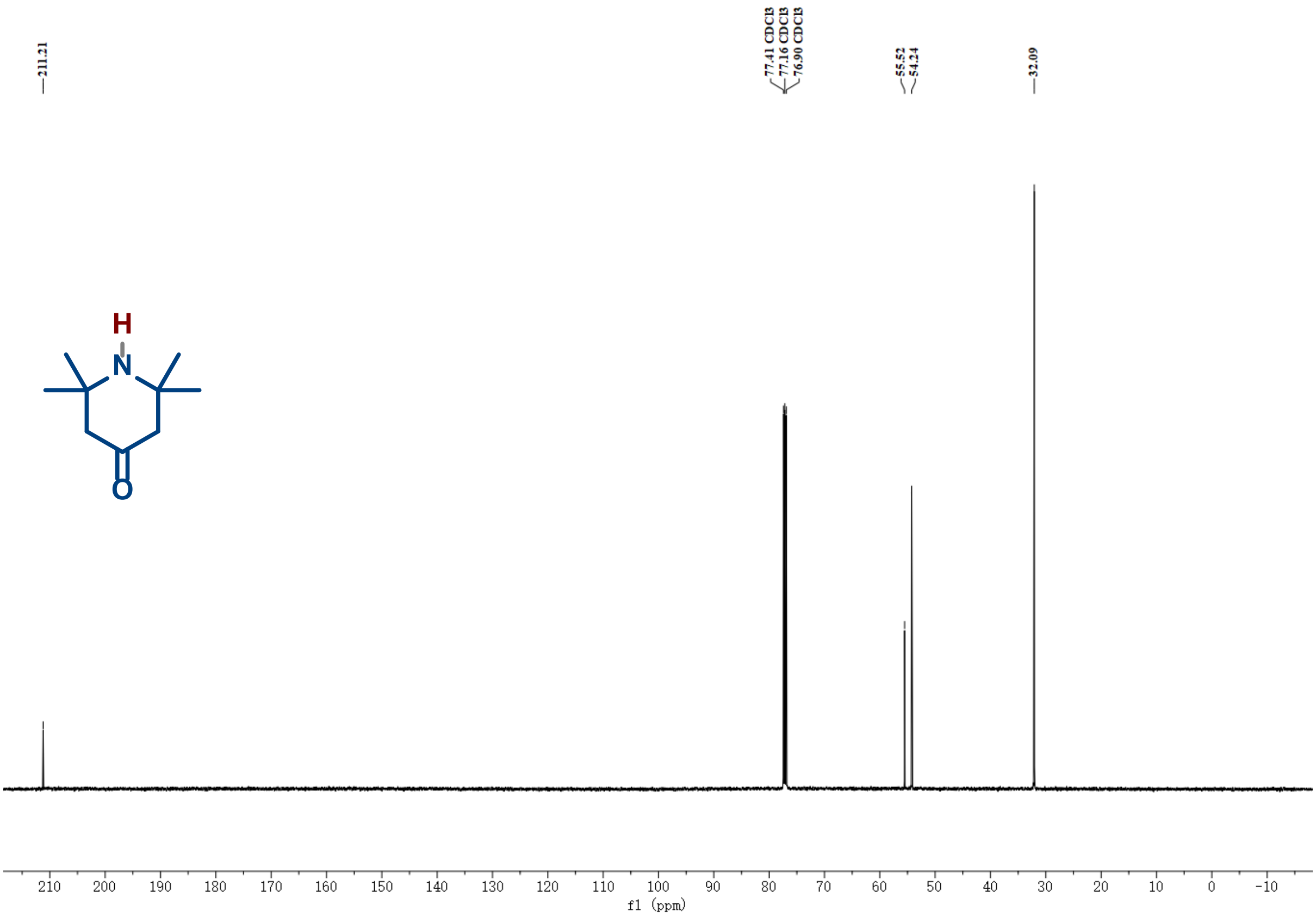

^{13}C NMR (126 MHz, CDCl$_3$)

2,2,6,6-tetramethylpiperidin-4-ol (12)

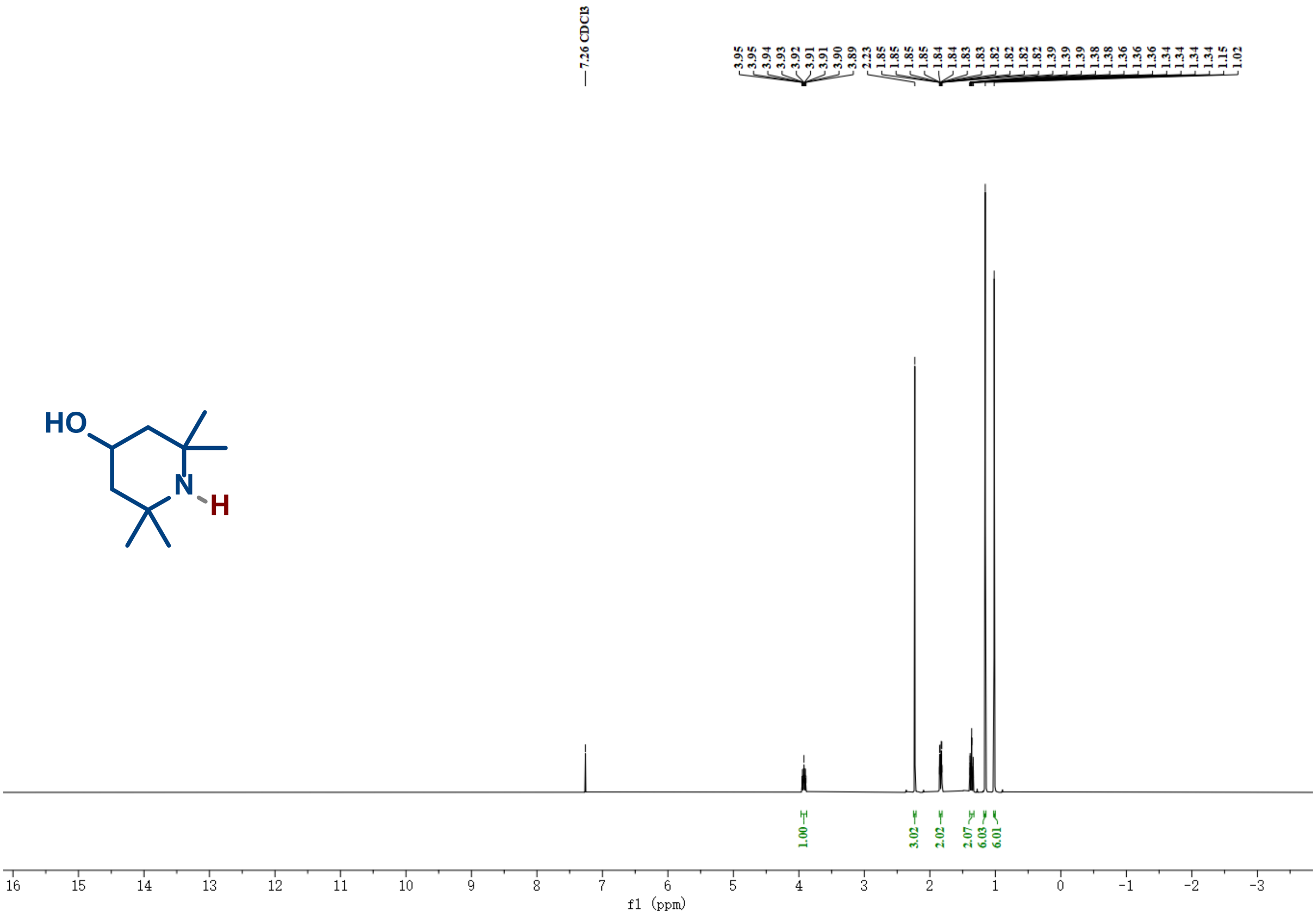

^{1}H NMR (500 MHz, CDCl$_3$)

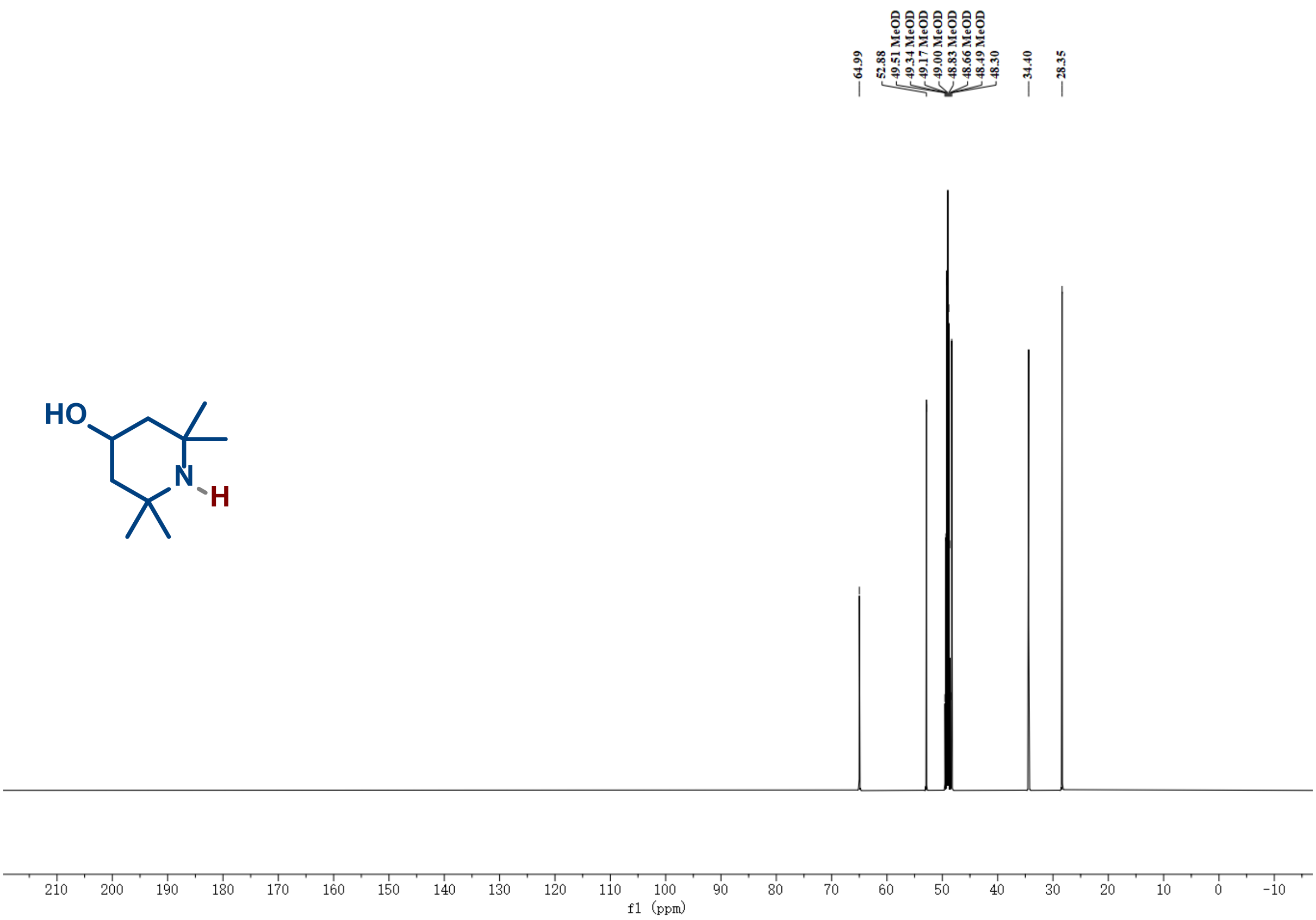

^{13}C NMR (126 MHz, MeOD)

2,2,6,6-tetramethylpiperidin-4-yl methacrylate (13)

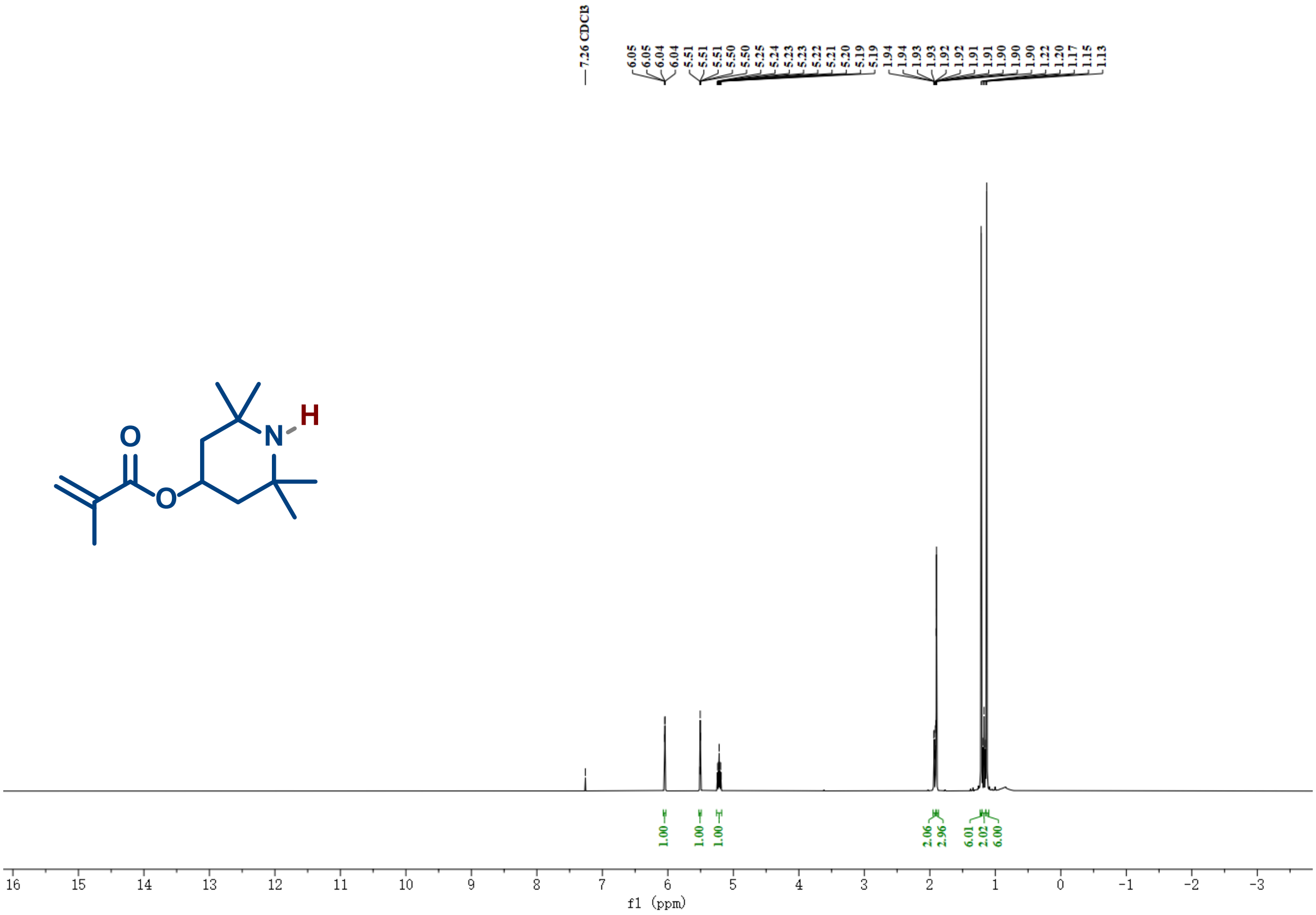

^{1}H NMR (500 MHz, $CDCl_3$)

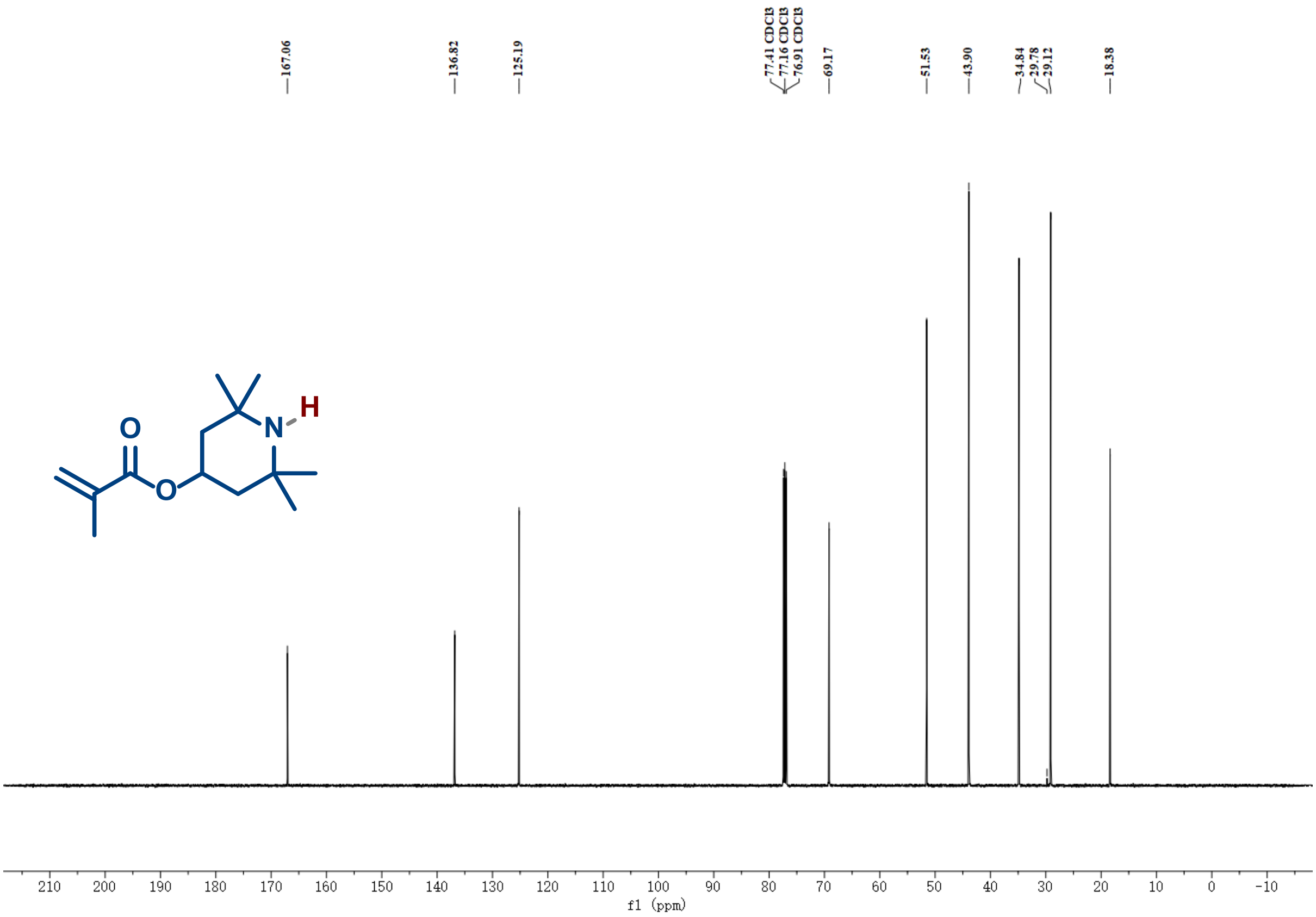

^{13}C NMR (126 MHz, $CDCl_3$)

N,N,2,2,6,6-hexamethylpiperidin-4-amine (14)

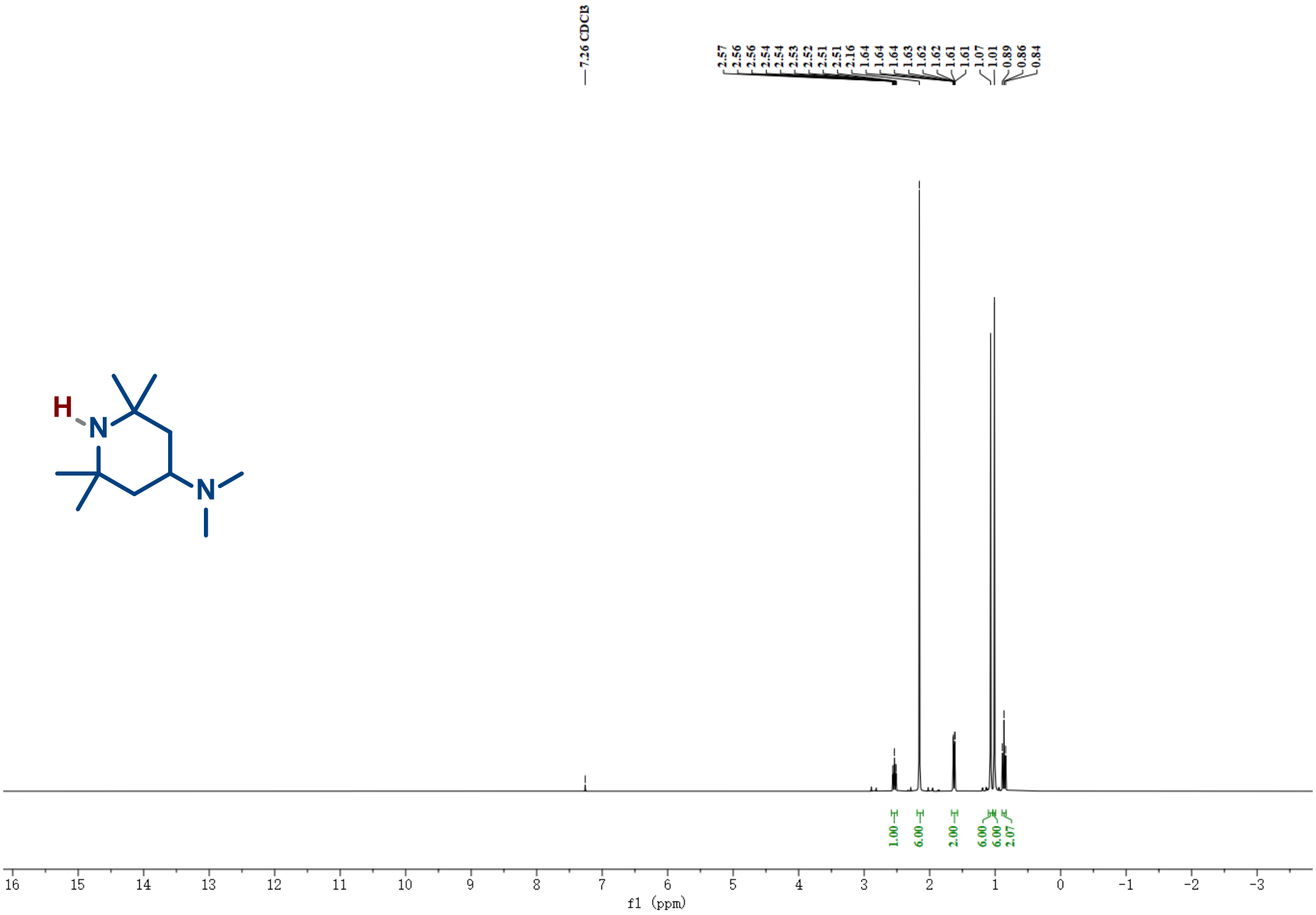

^{1}H NMR (500 MHz, $CDCl_3$)

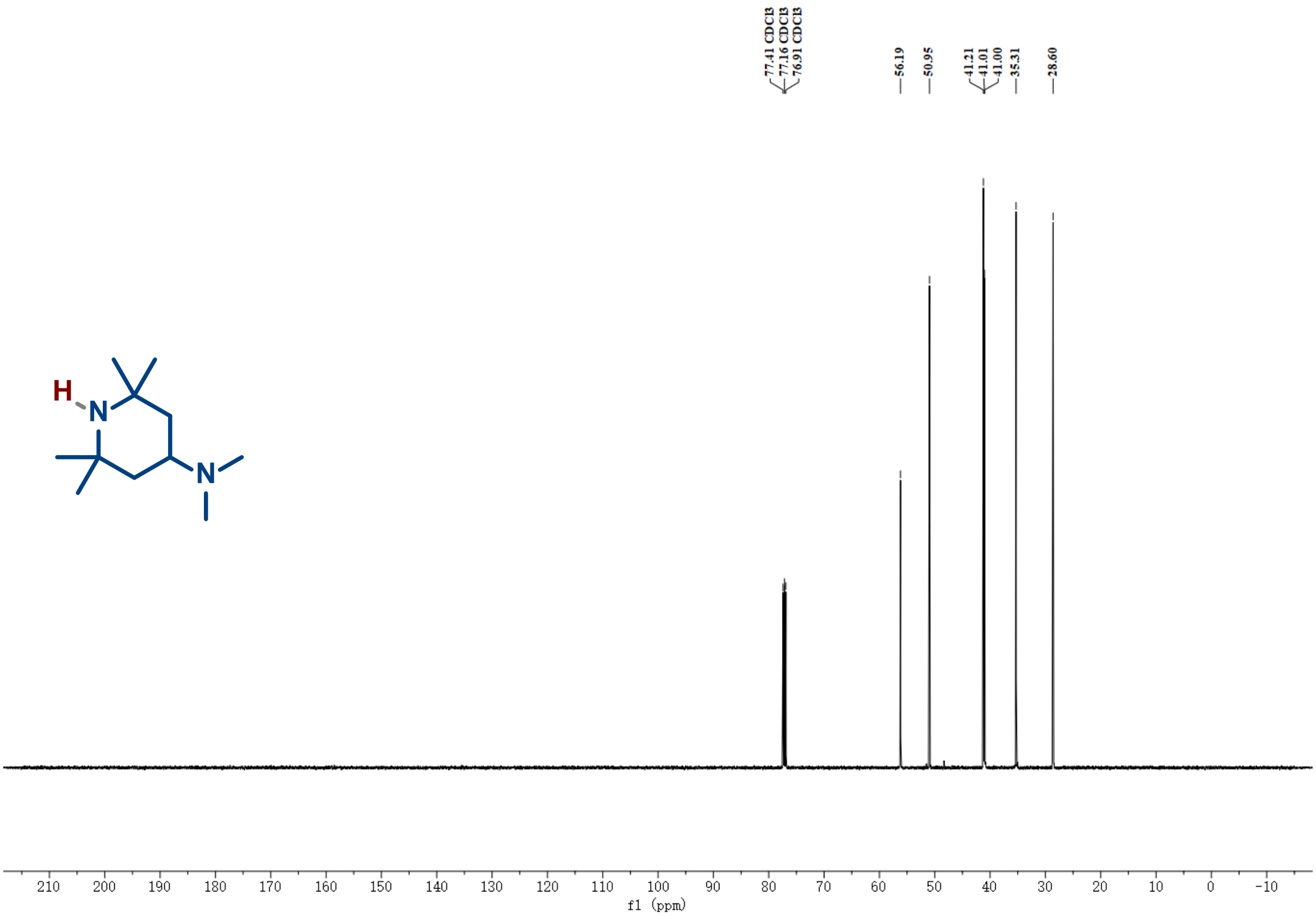

^{13}C NMR (126 MHz, $CDCl_3$)

bis(2,2,6,6-tetramethylpiperidin-4-yl) decanedioate (15)

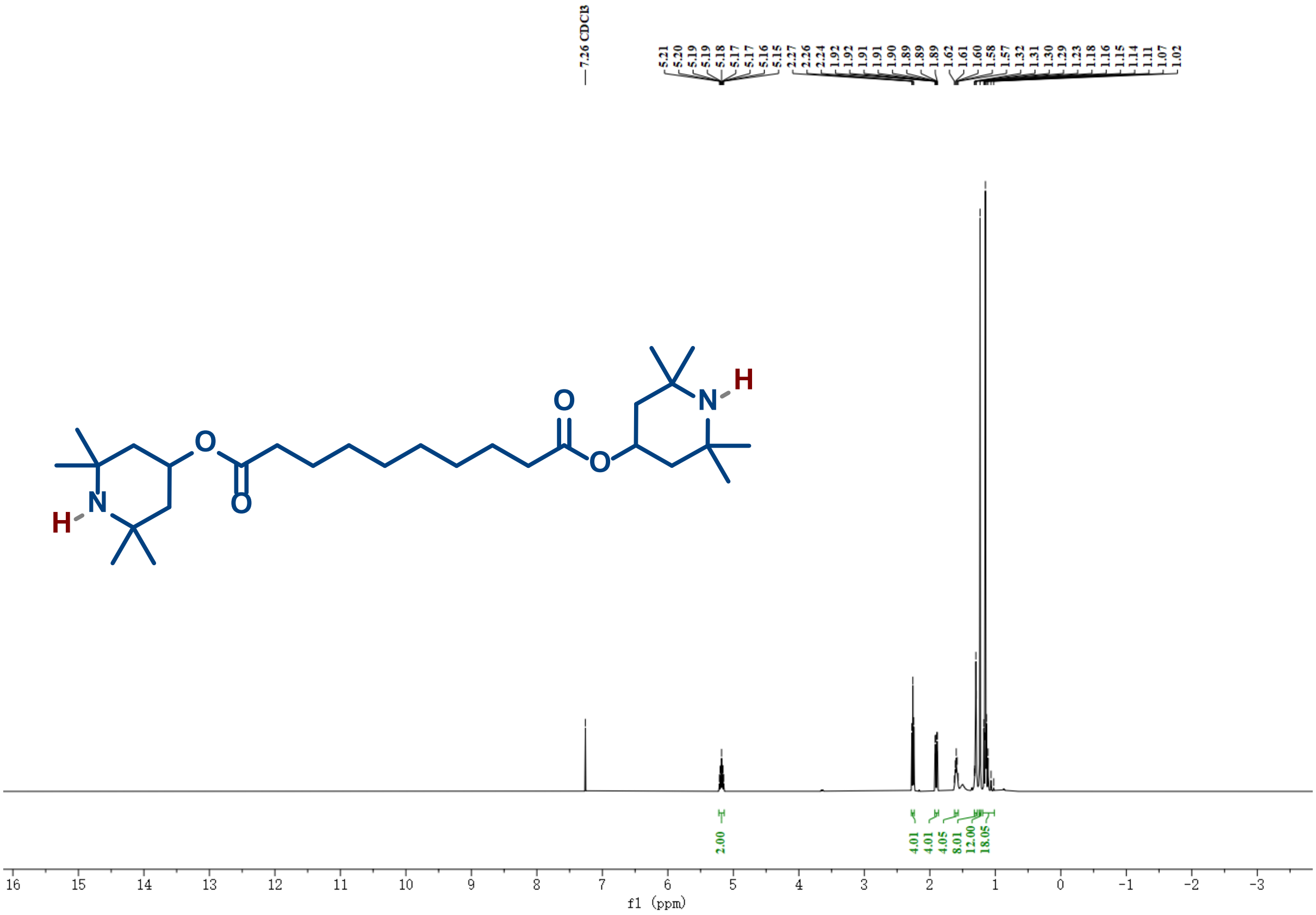

^{1}H NMR (500 MHz, CDCl$_3$)

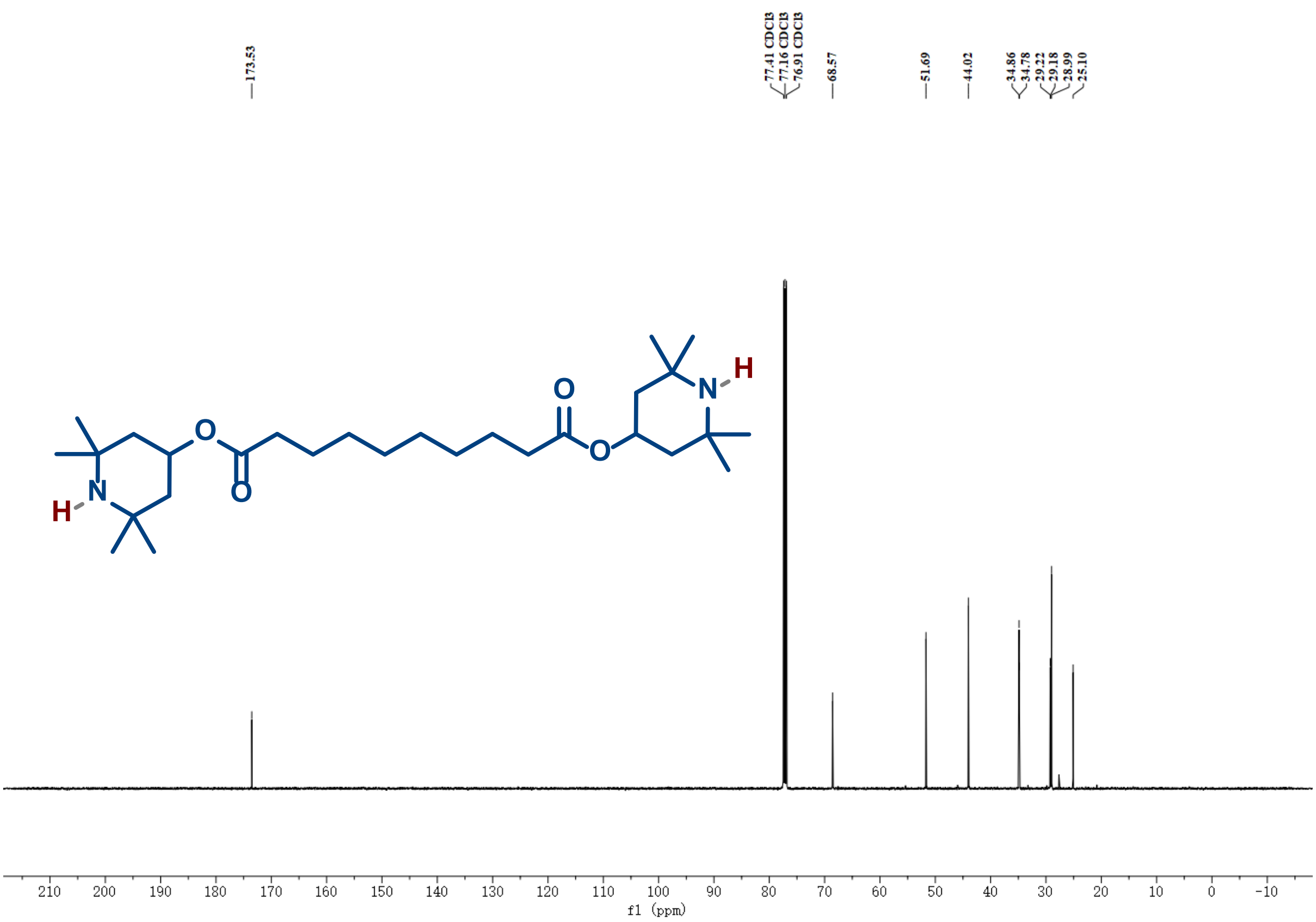

^{13}C NMR (126 MHz, CDCl$_3$)

N-cyclopentylaniline (16)

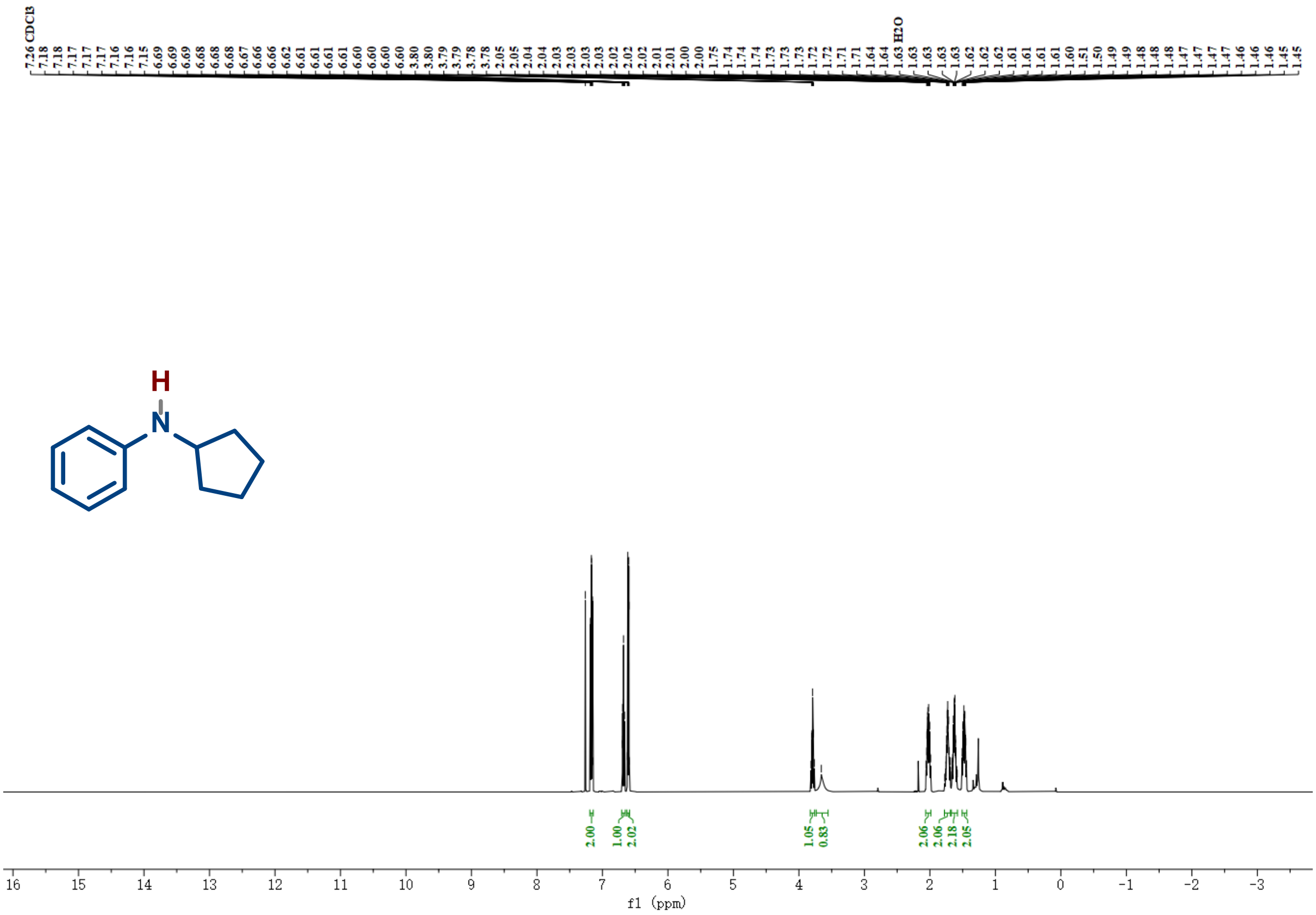

^{1}H NMR (500 MHz, $CDCl_3$)

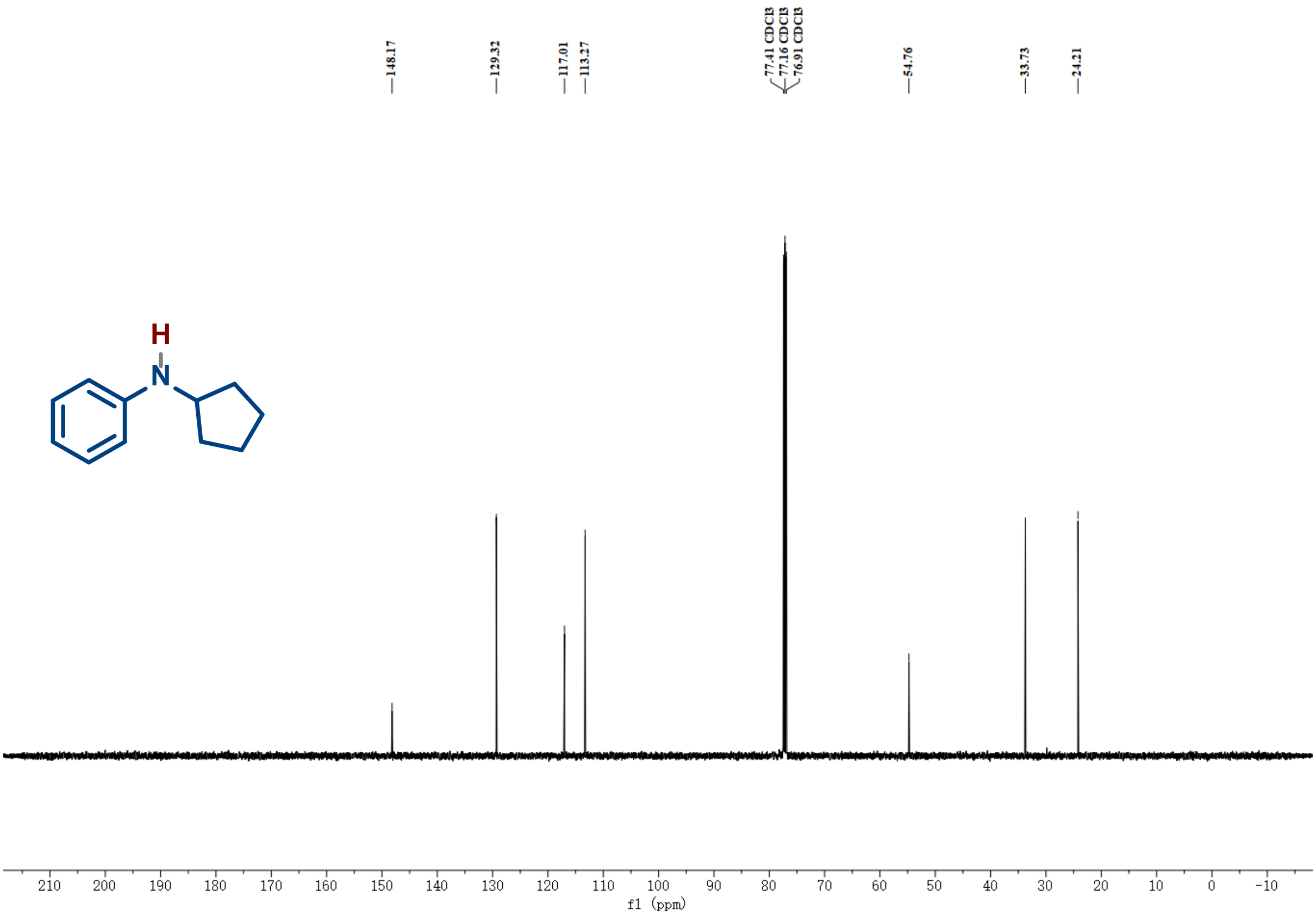

^{13}C NMR (126 MHz, $CDCl_3$)

4-chloro-*N*-cyclopentylaniline (17)

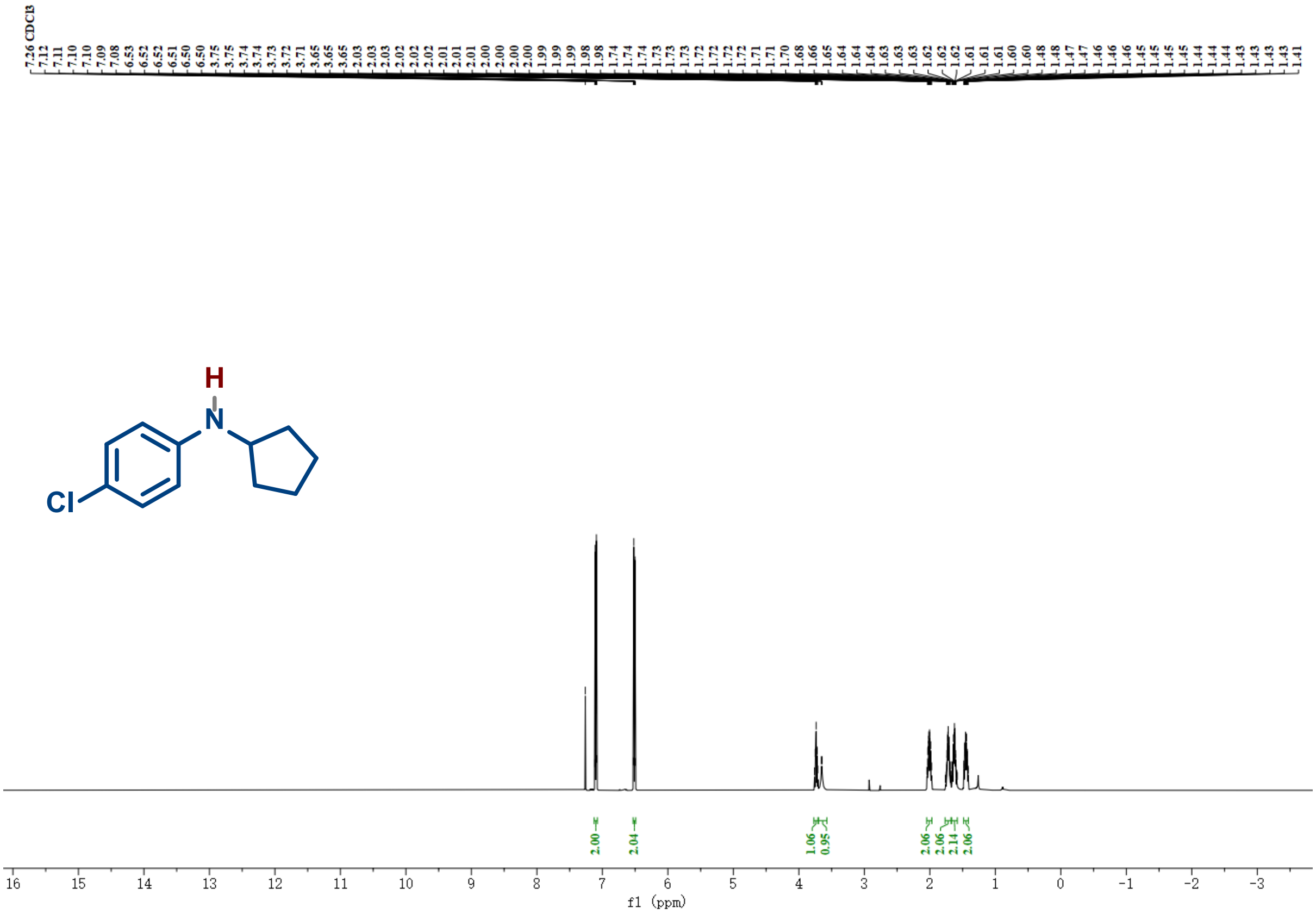

^{1}H NMR (500 MHz, $CDCl_3$)

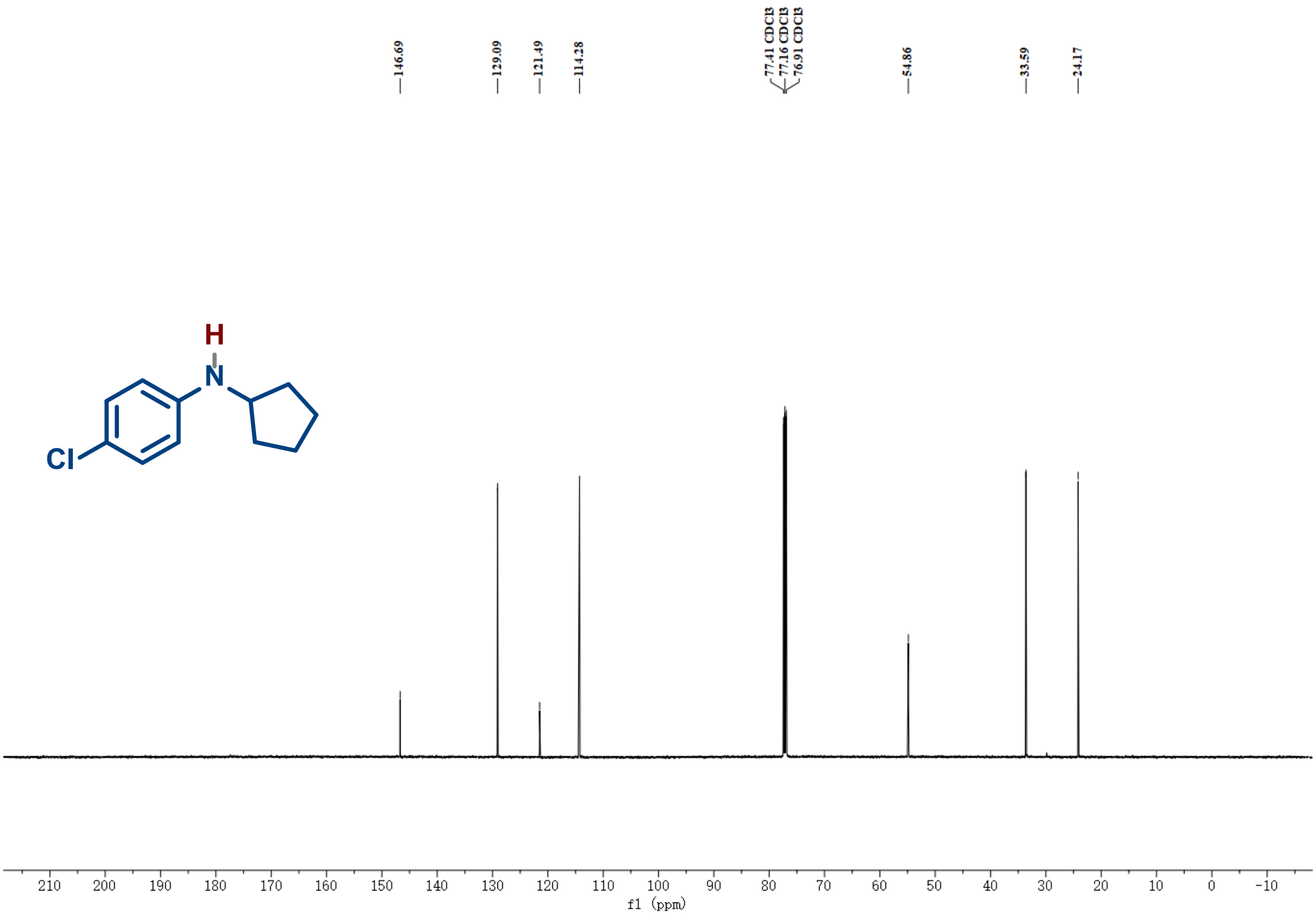

^{13}C NMR (126 MHz, $CDCl_3$)

N-cyclopentyl-4-(trifluoromethyl)aniline (18)

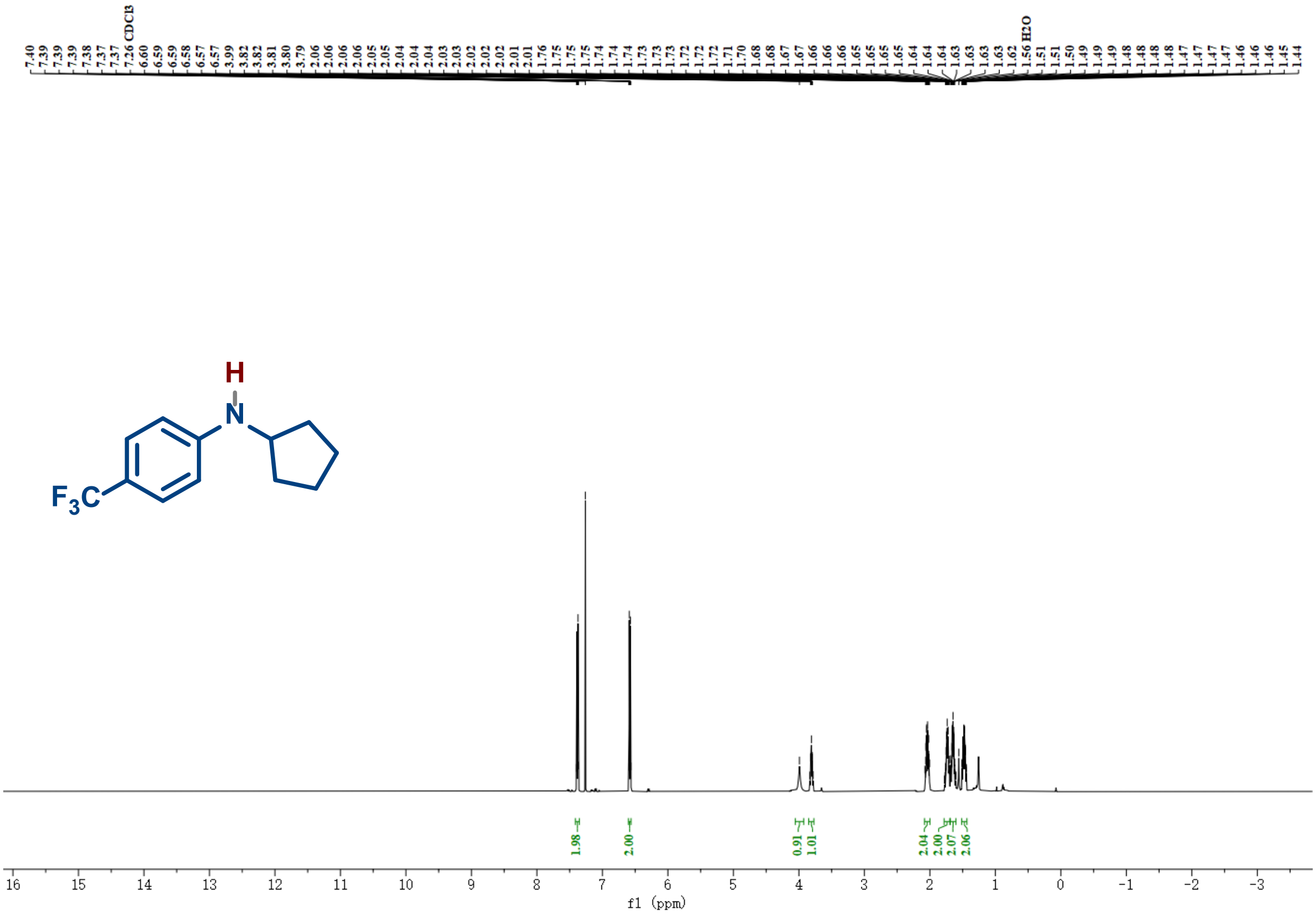

^{1}H NMR (500 MHz, $CDCl_3$)

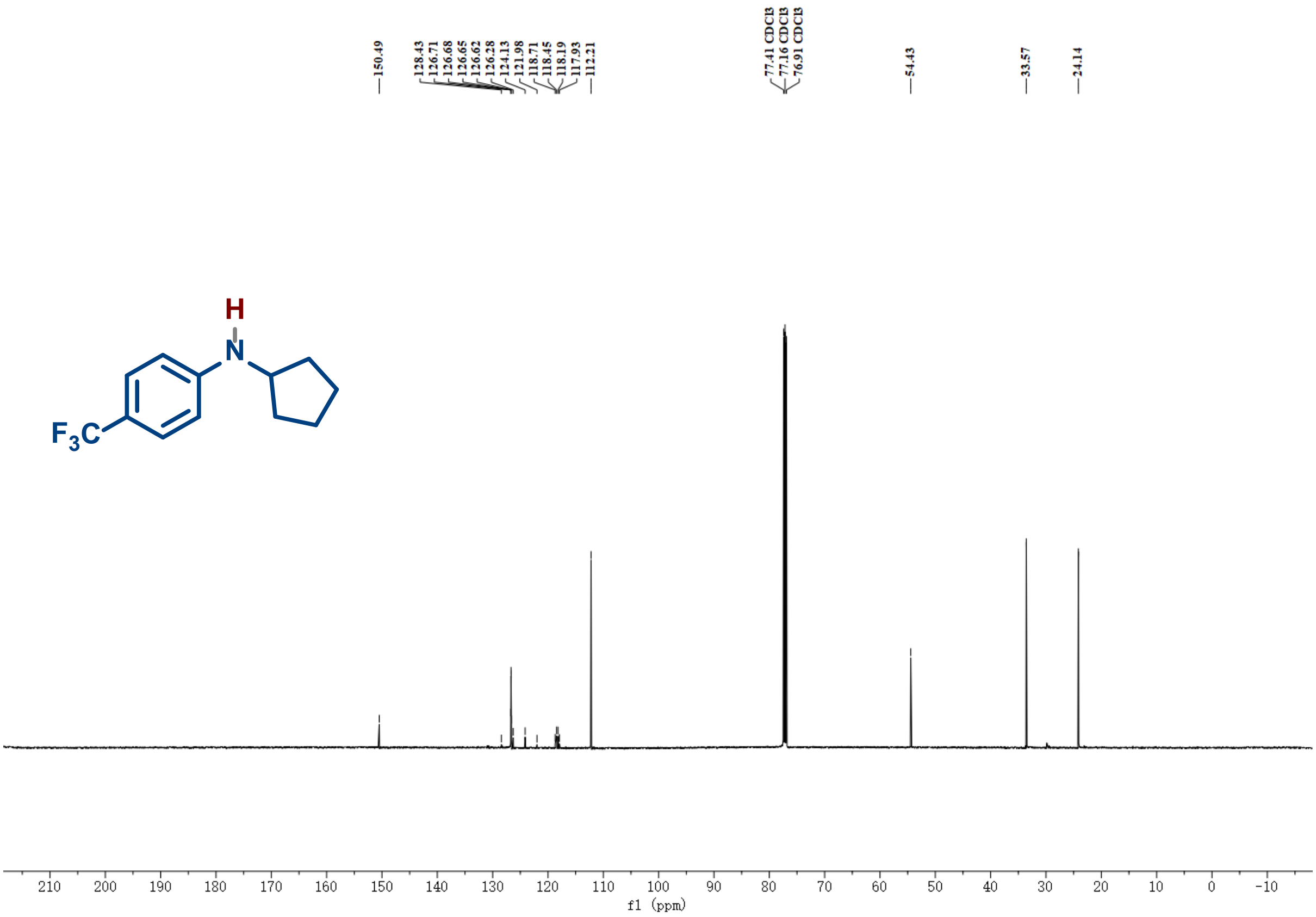

^{13}C NMR (126 MHz, $CDCl_3$)

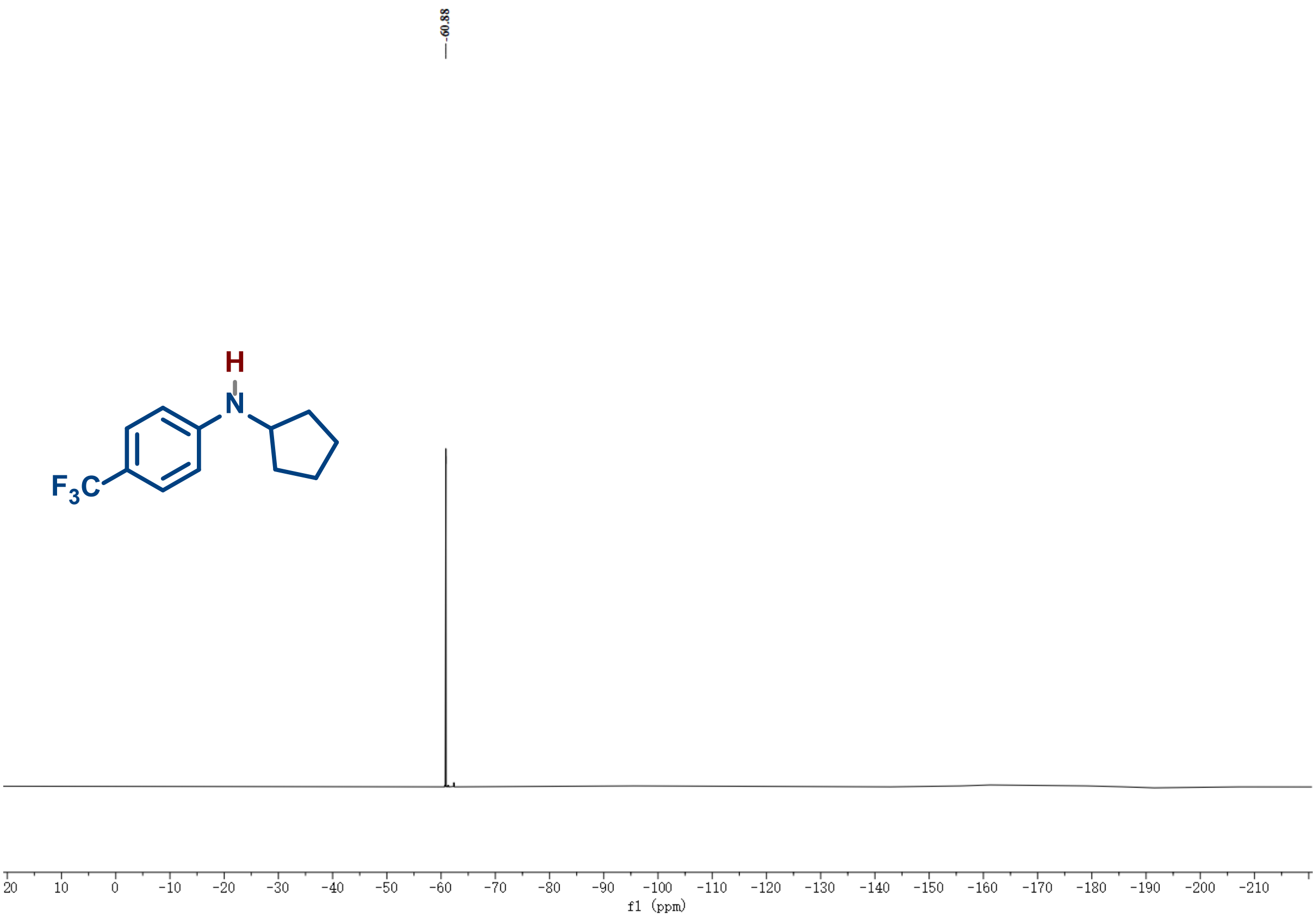

^{19}F NMR (471 MHz, $CDCl_3$)

***N*-cyclopentyl-4-methoxyaniline (19)**

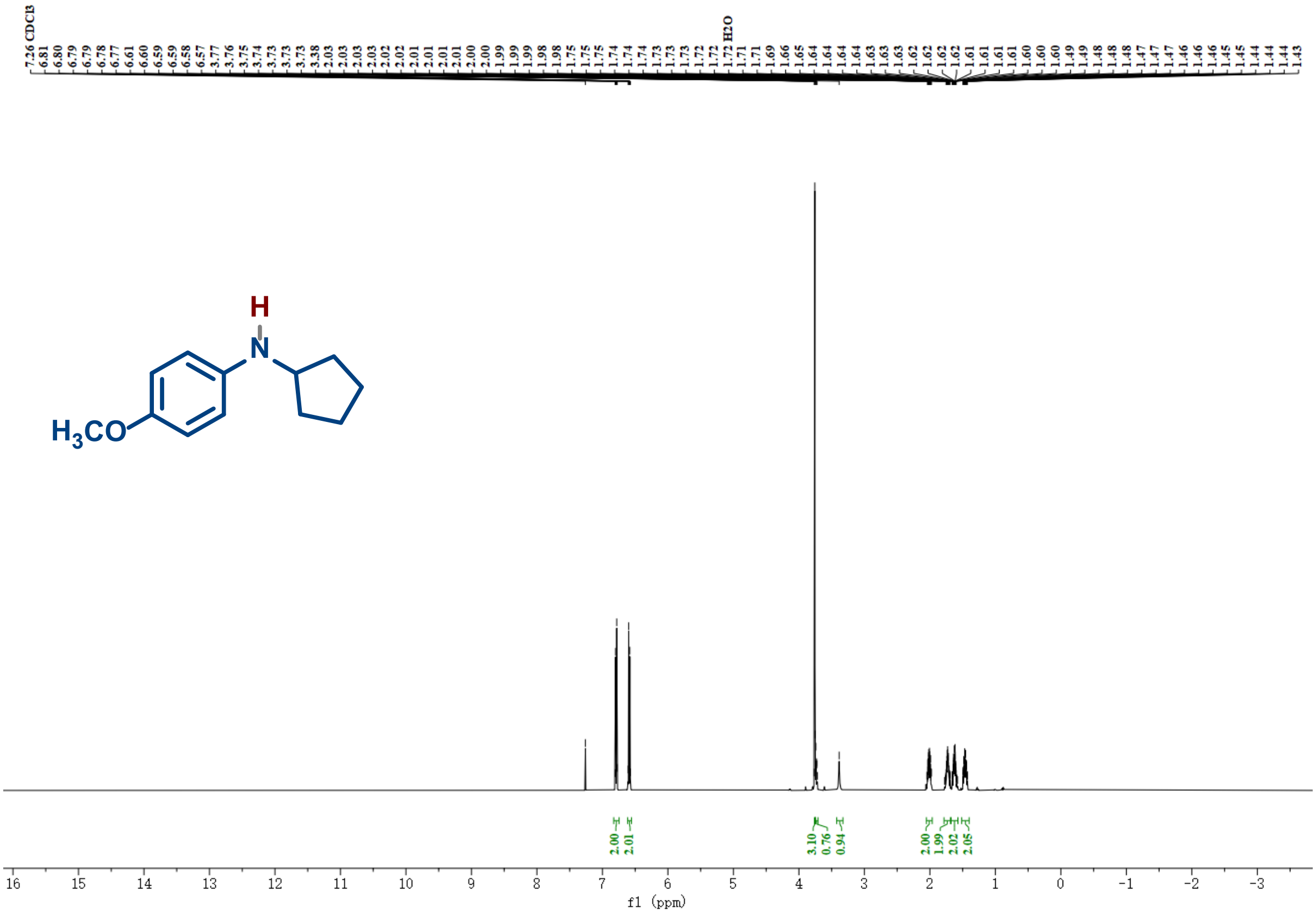

^{1}H NMR (500 MHz, $CDCl_3$)

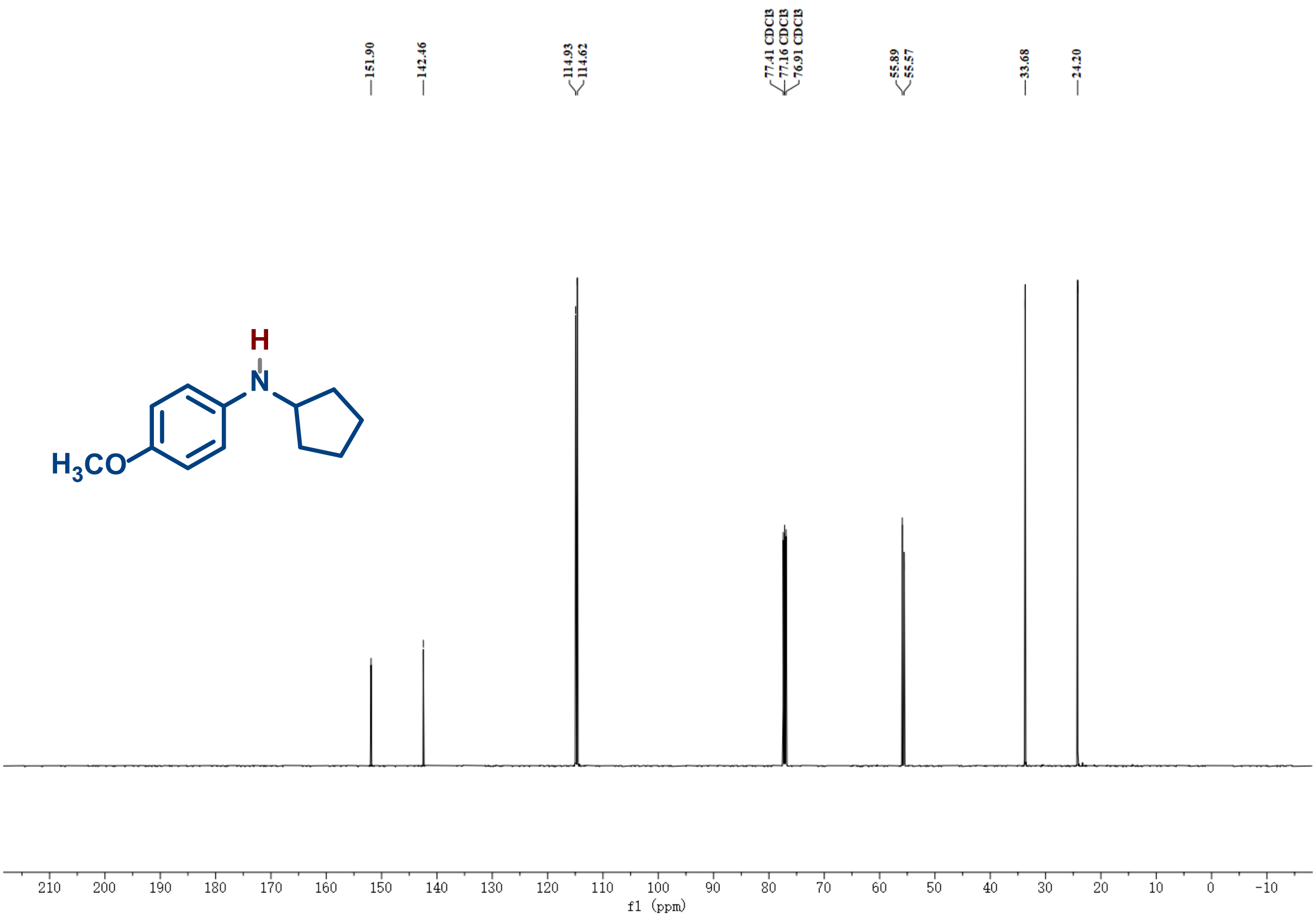

^{13}C NMR (126 MHz, $CDCl_3$)

***N*-cyclohexylaniline (20)**

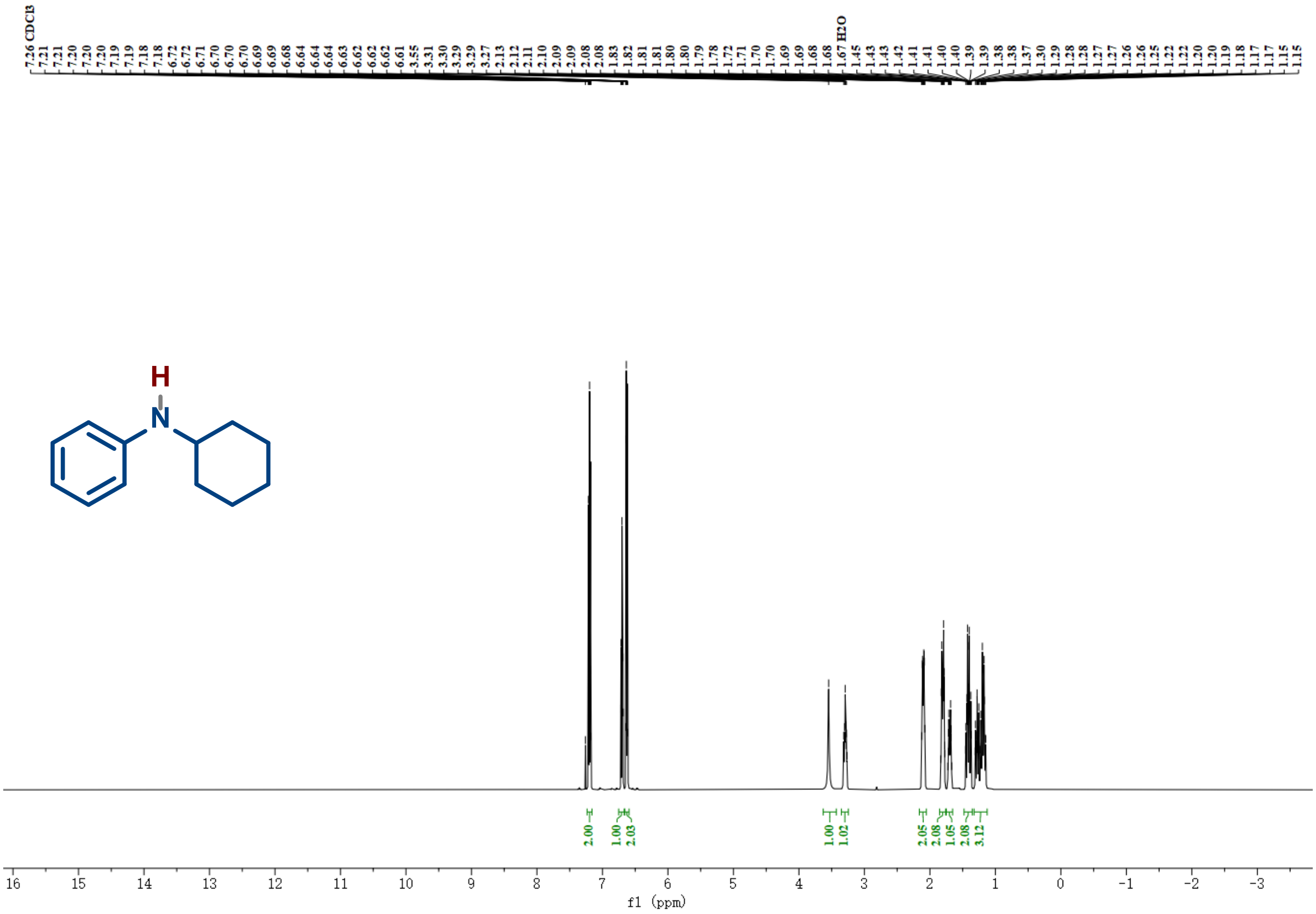

^{1}H NMR (500 MHz, $CDCl_3$)

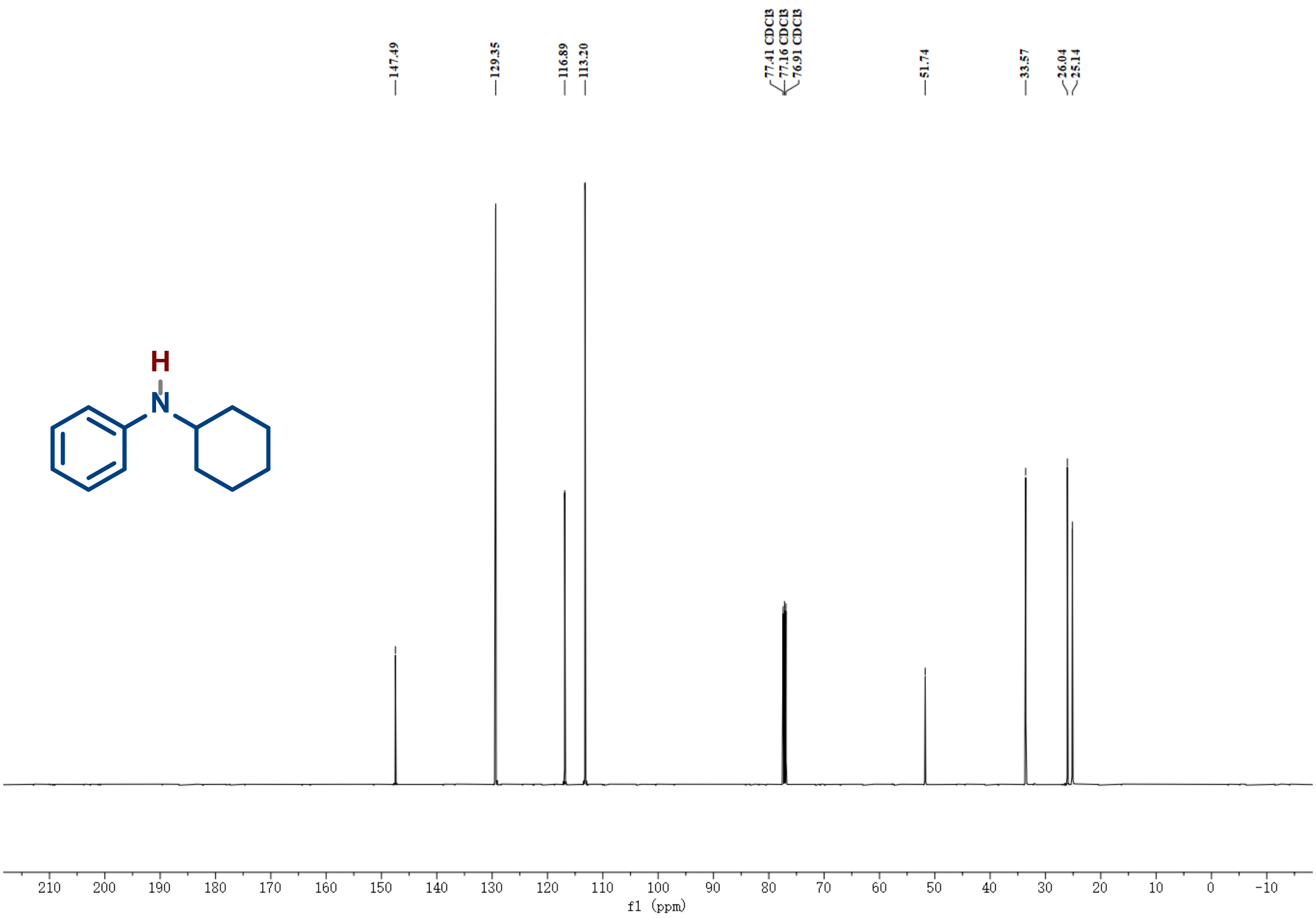

^{13}C NMR (126 MHz, $CDCl_3$)

_N_-cyclohexyl-4-fluoroaniline (21)

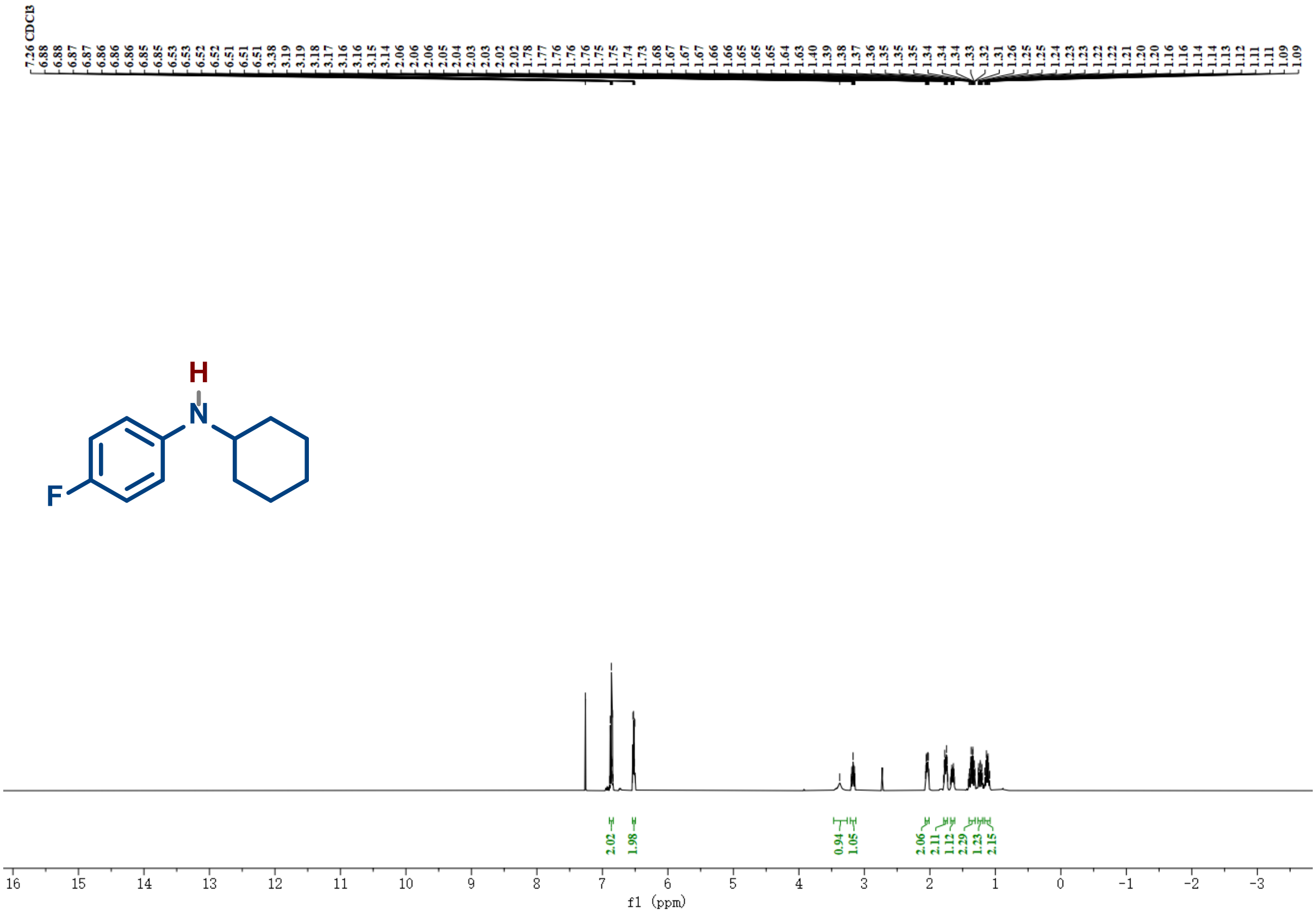

^{1}H NMR (500 MHz, $CDCl_3$)

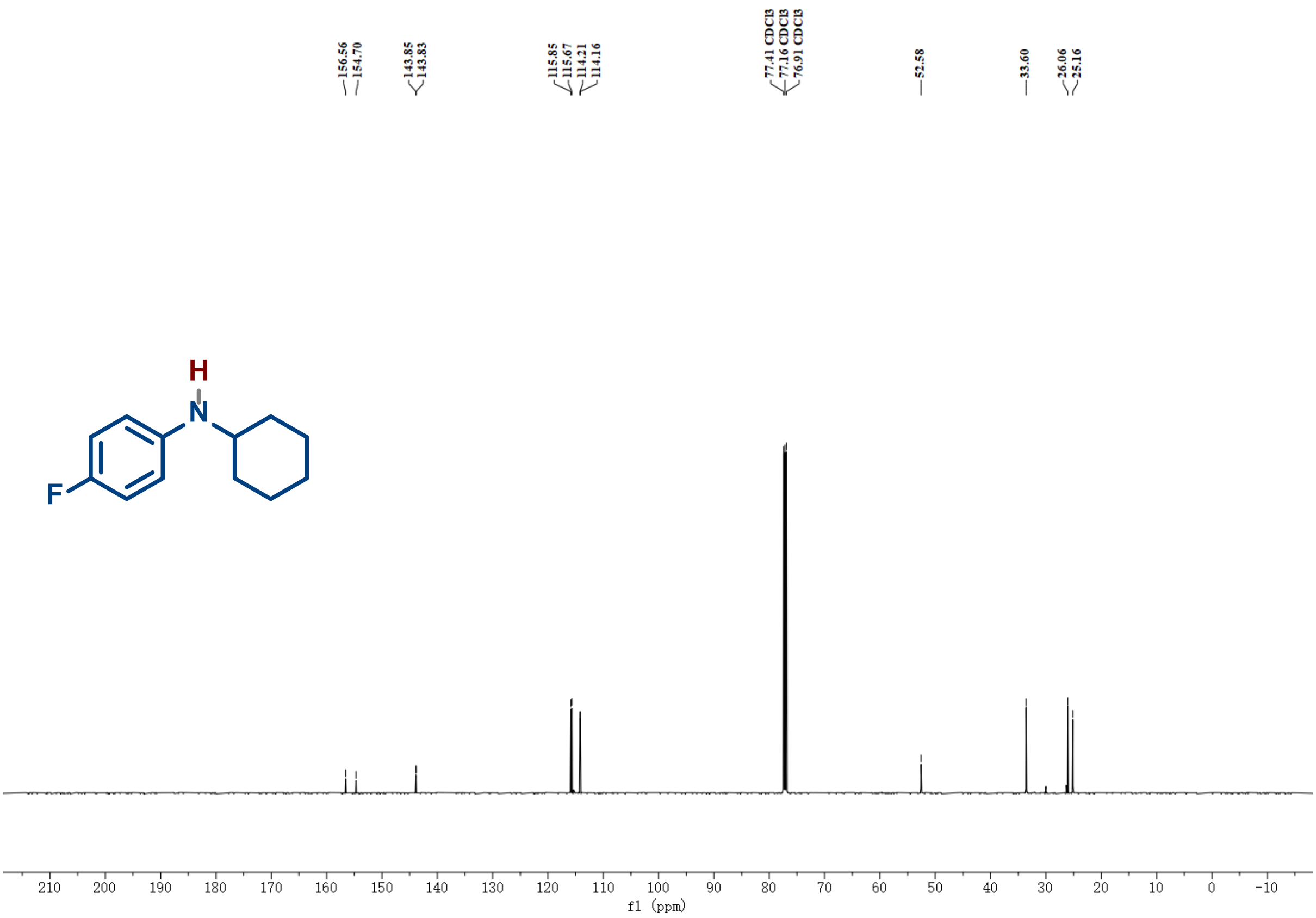

^{13}C NMR (126 MHz, $CDCl_3$)

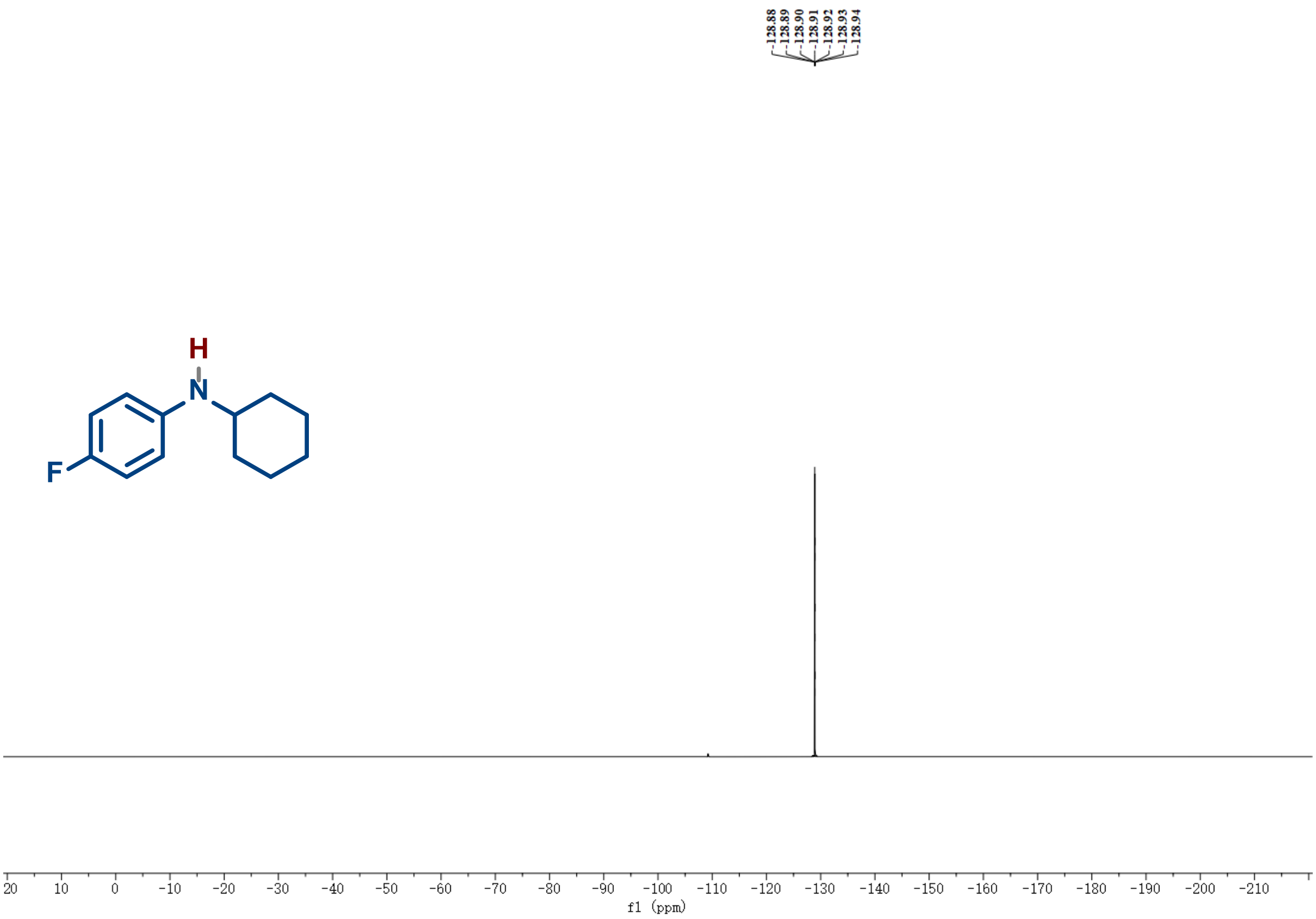

^{19}F NMR (471 MHz, $CDCl_3$)

4-bromo-*N*-cyclohexylaniline (22)

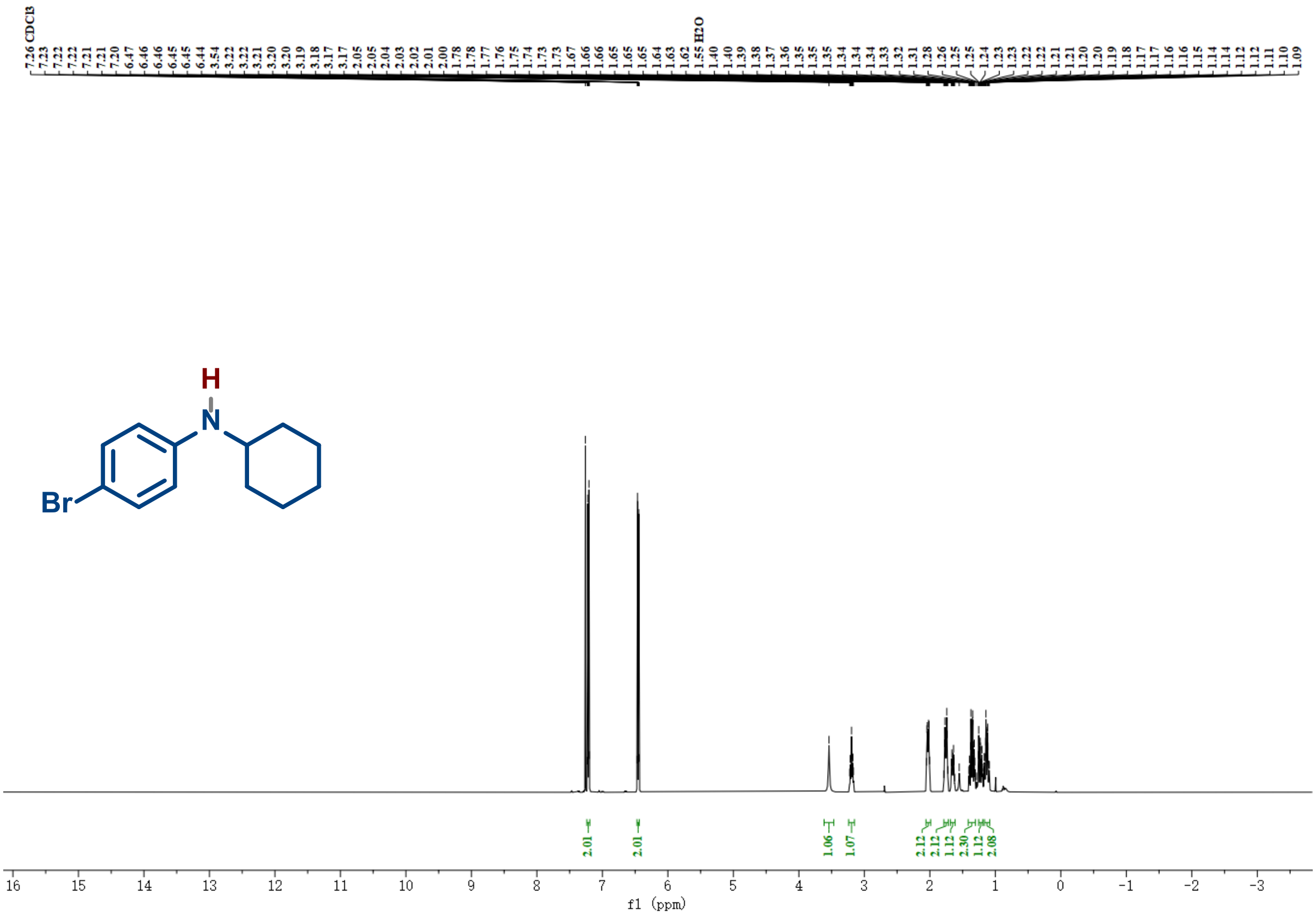

^{1}H NMR (500 MHz, $CDCl_3$)

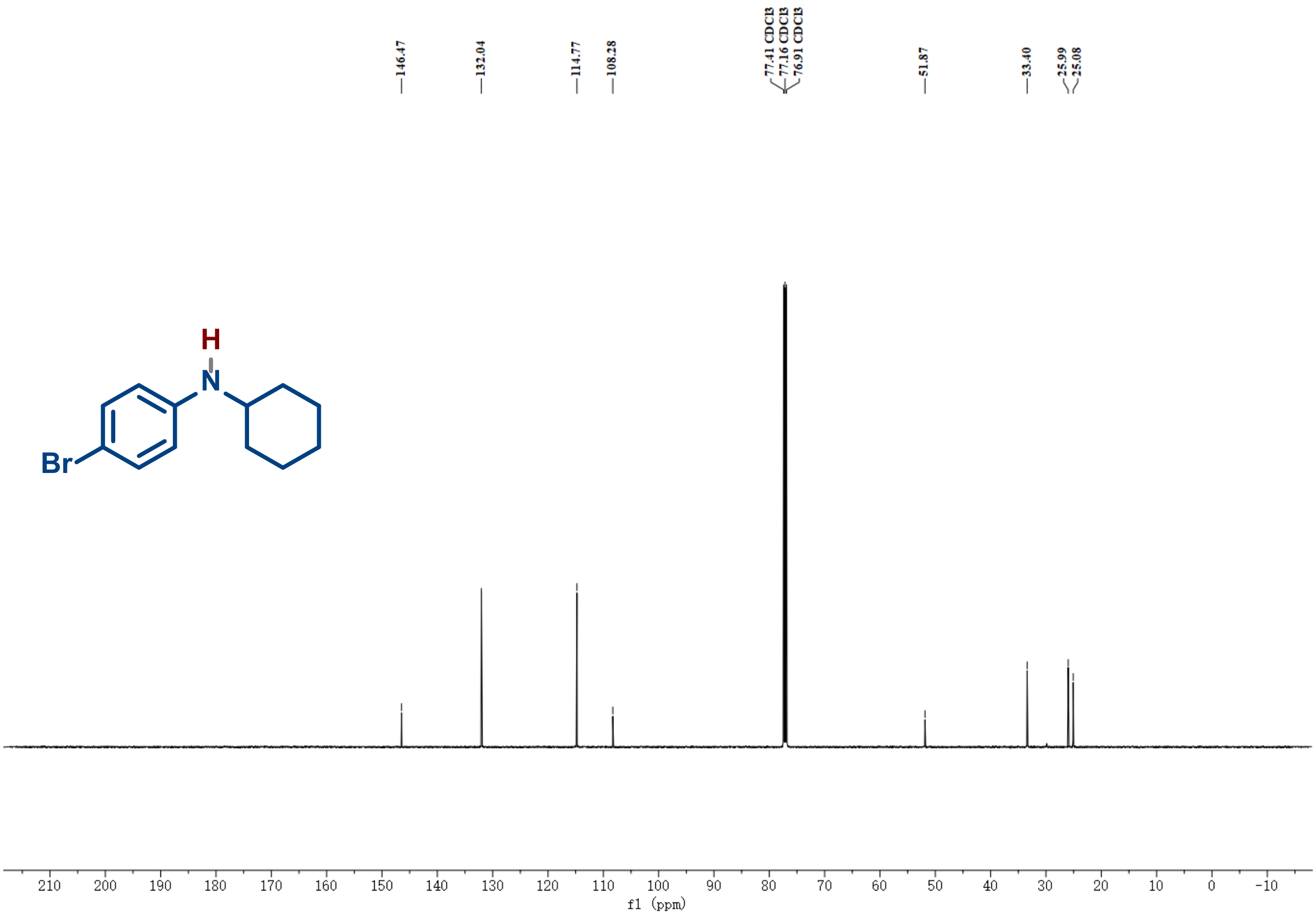

^{13}C NMR (126 MHz, $CDCl_3$)

N-cyclohexyl-4-nitroaniline (23)

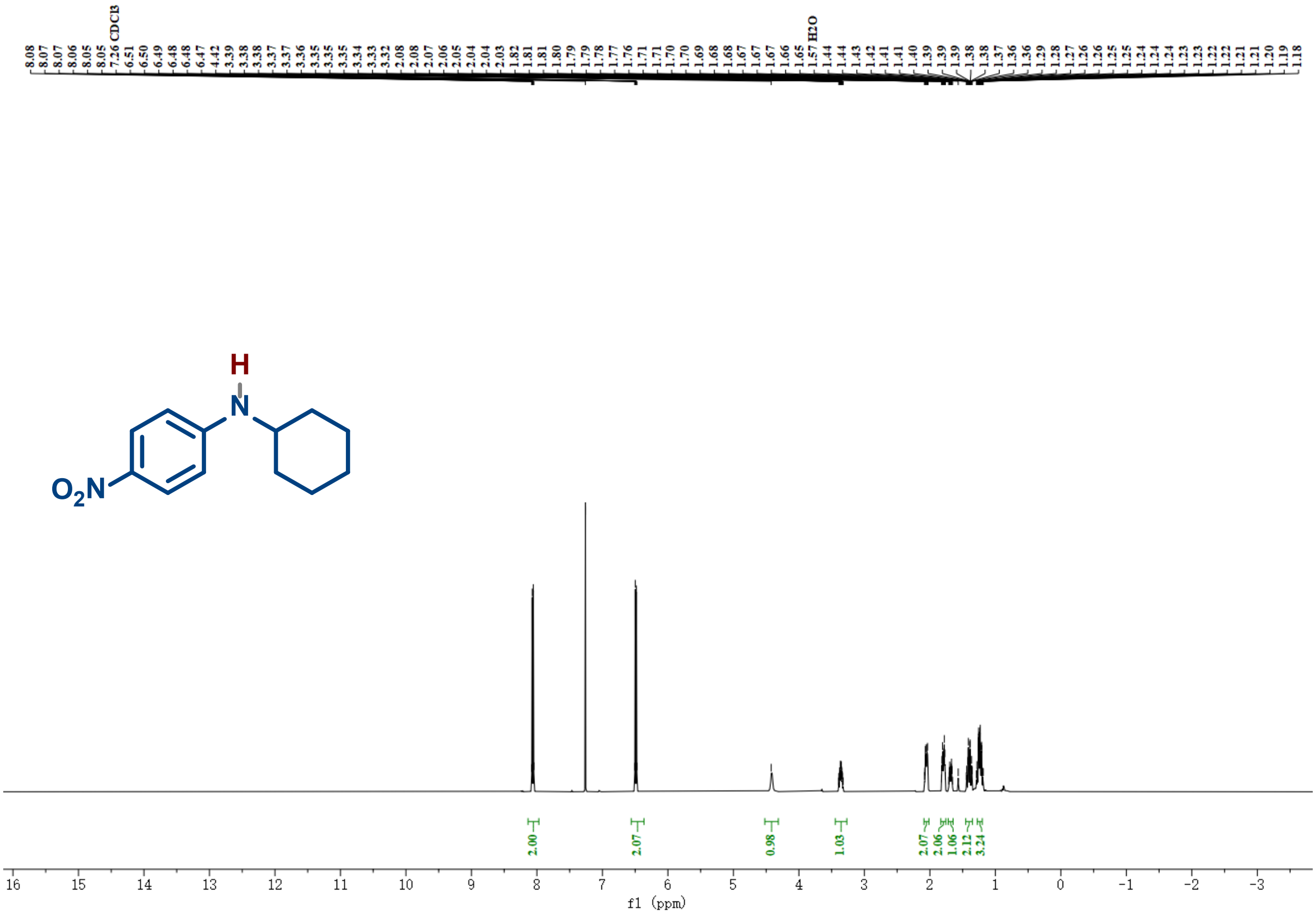

^{1}H NMR (500 MHz, $CDCl_3$)

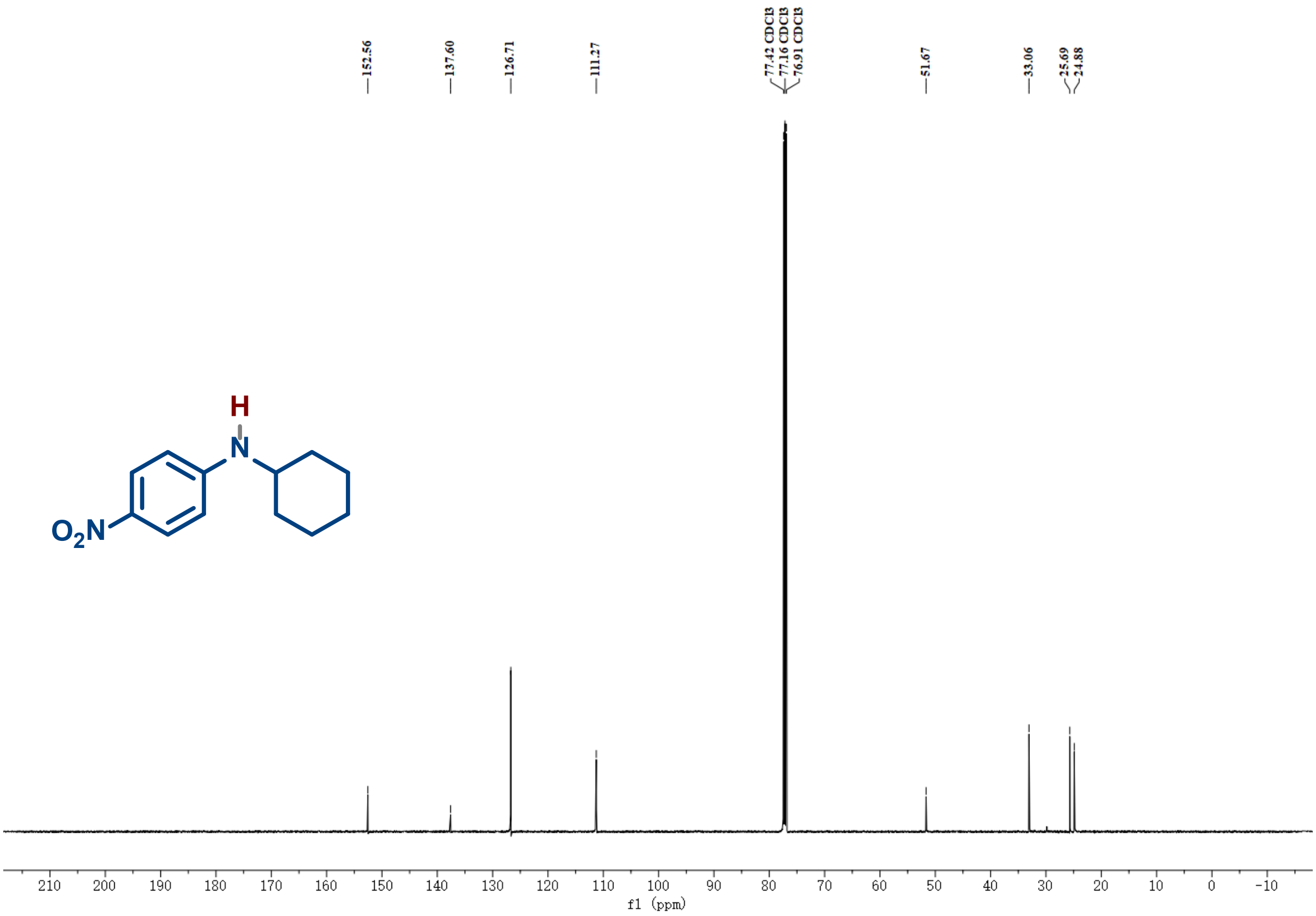

^{13}C NMR (126 MHz, $CDCl_3$)

_N_-cyclohexyl-3-methylaniline (24)

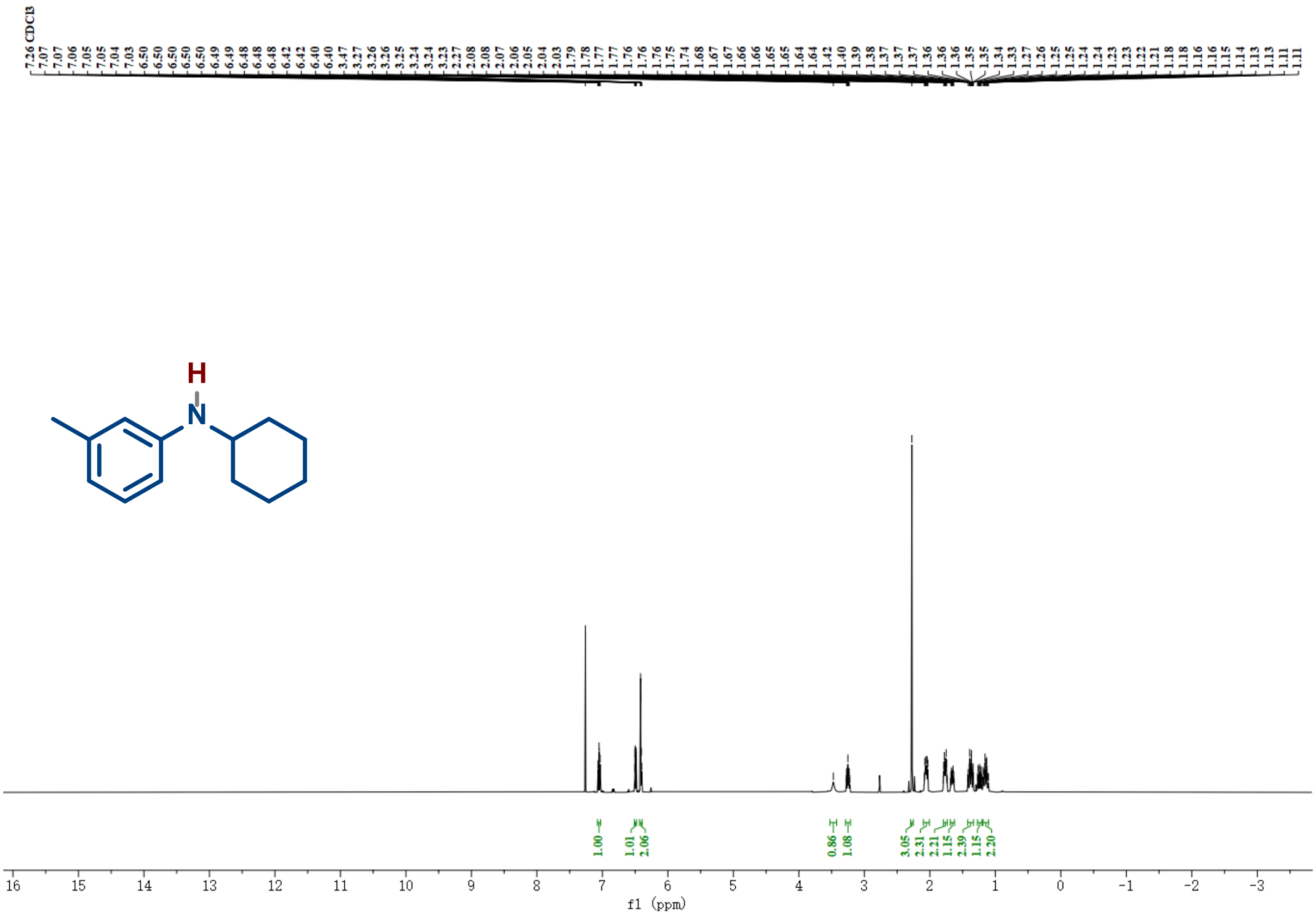

^{1}H NMR (500 MHz, $CDCl_3$)

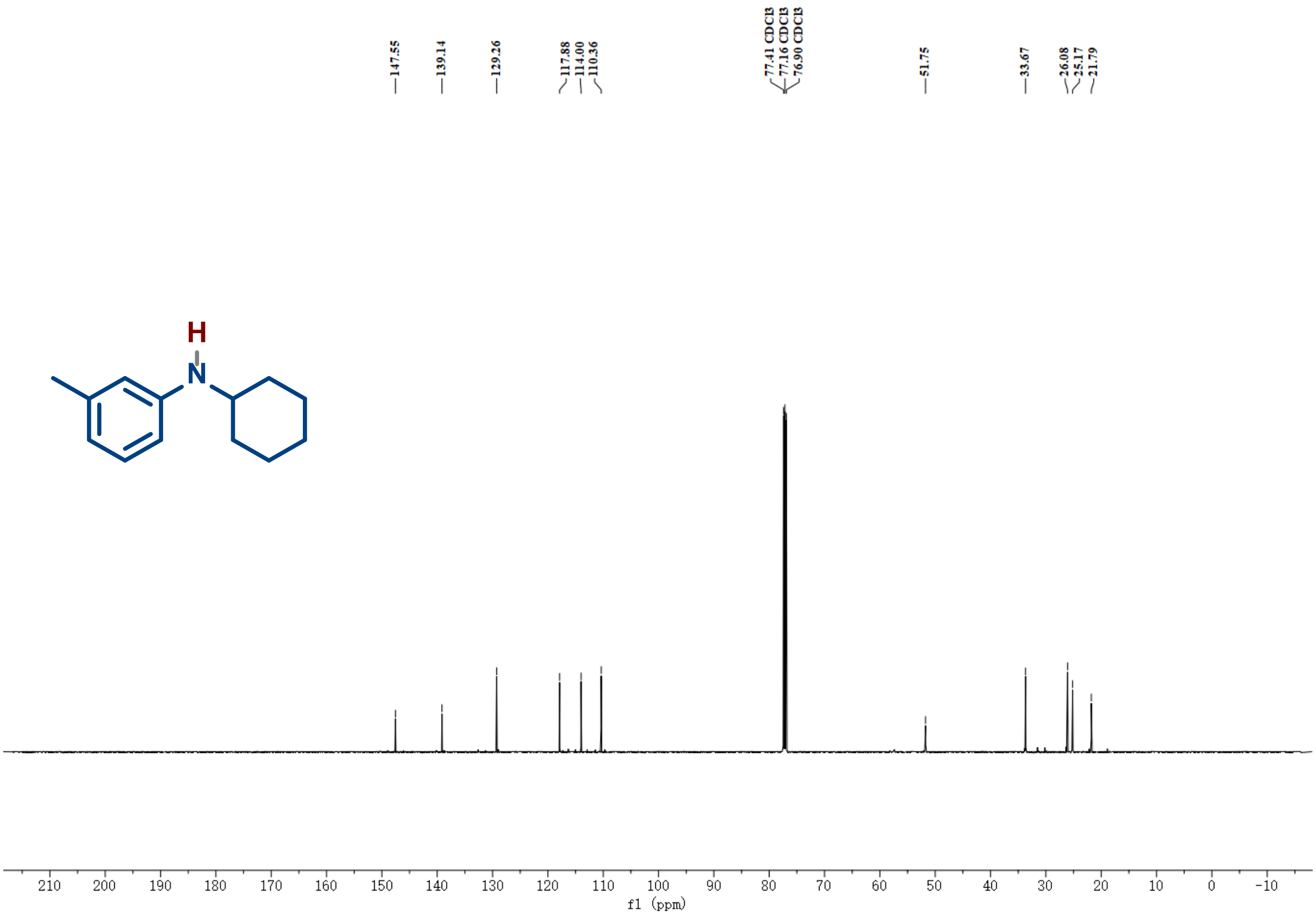

^{13}C NMR (126 MHz, $CDCl_3$)

N-(4,4-dimethoxycyclohexyl)aniline (25)

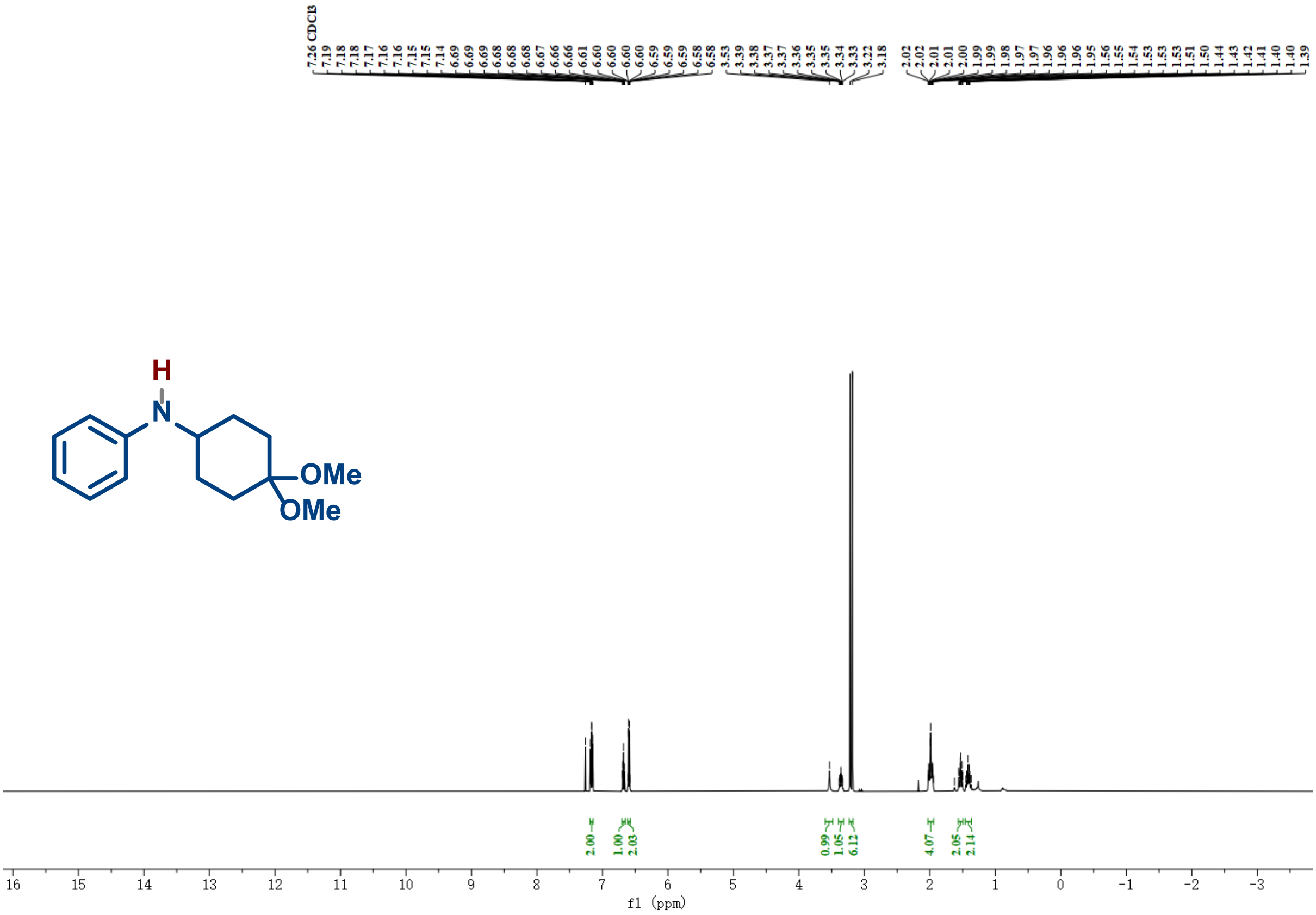

^{1}H NMR (500 MHz, $CDCl_3$)

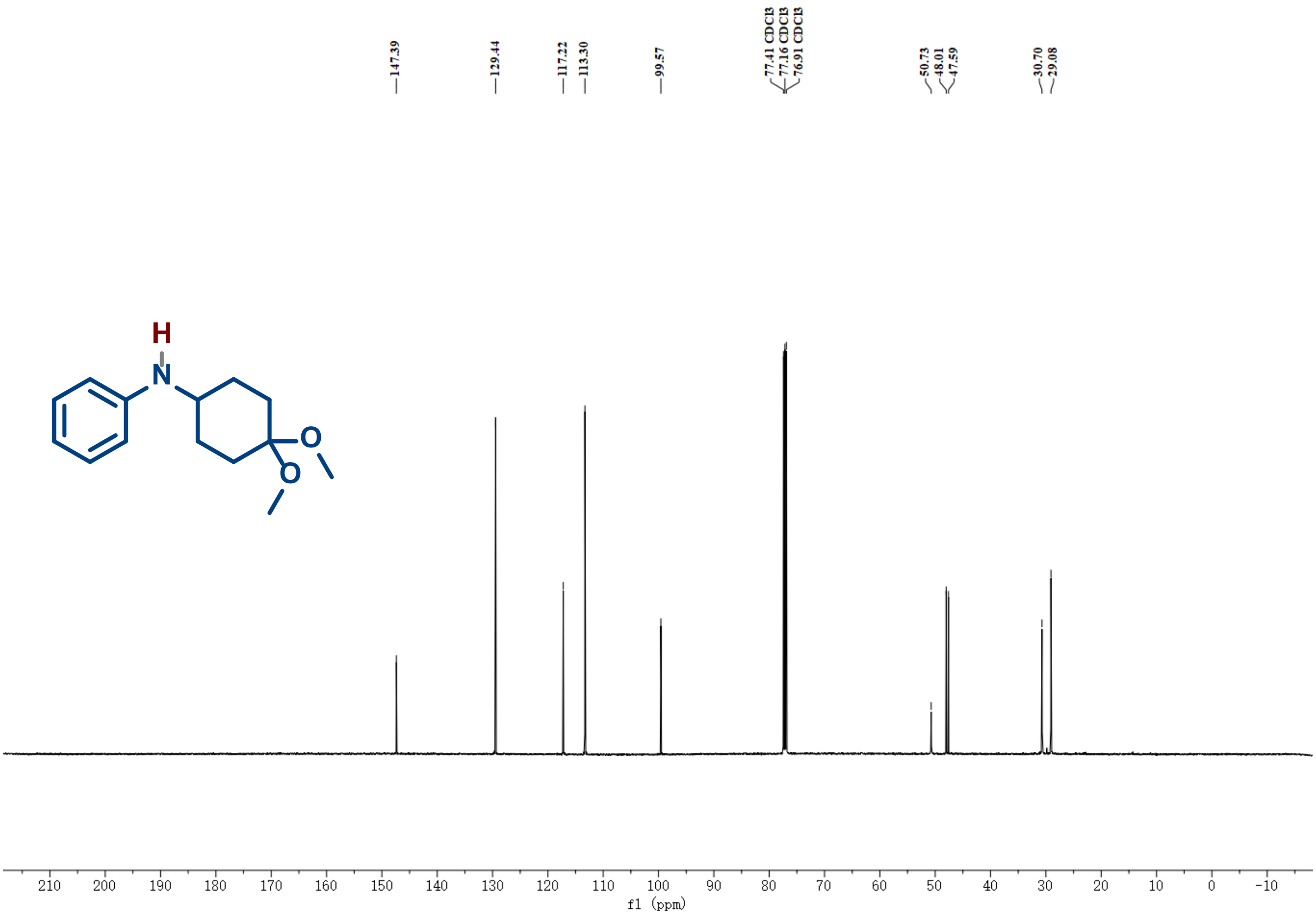

^{13}C NMR (126 MHz, $CDCl_3$)

N-phenyl-1,4-dioxaspiro[4.5]decan-8-amine (26)

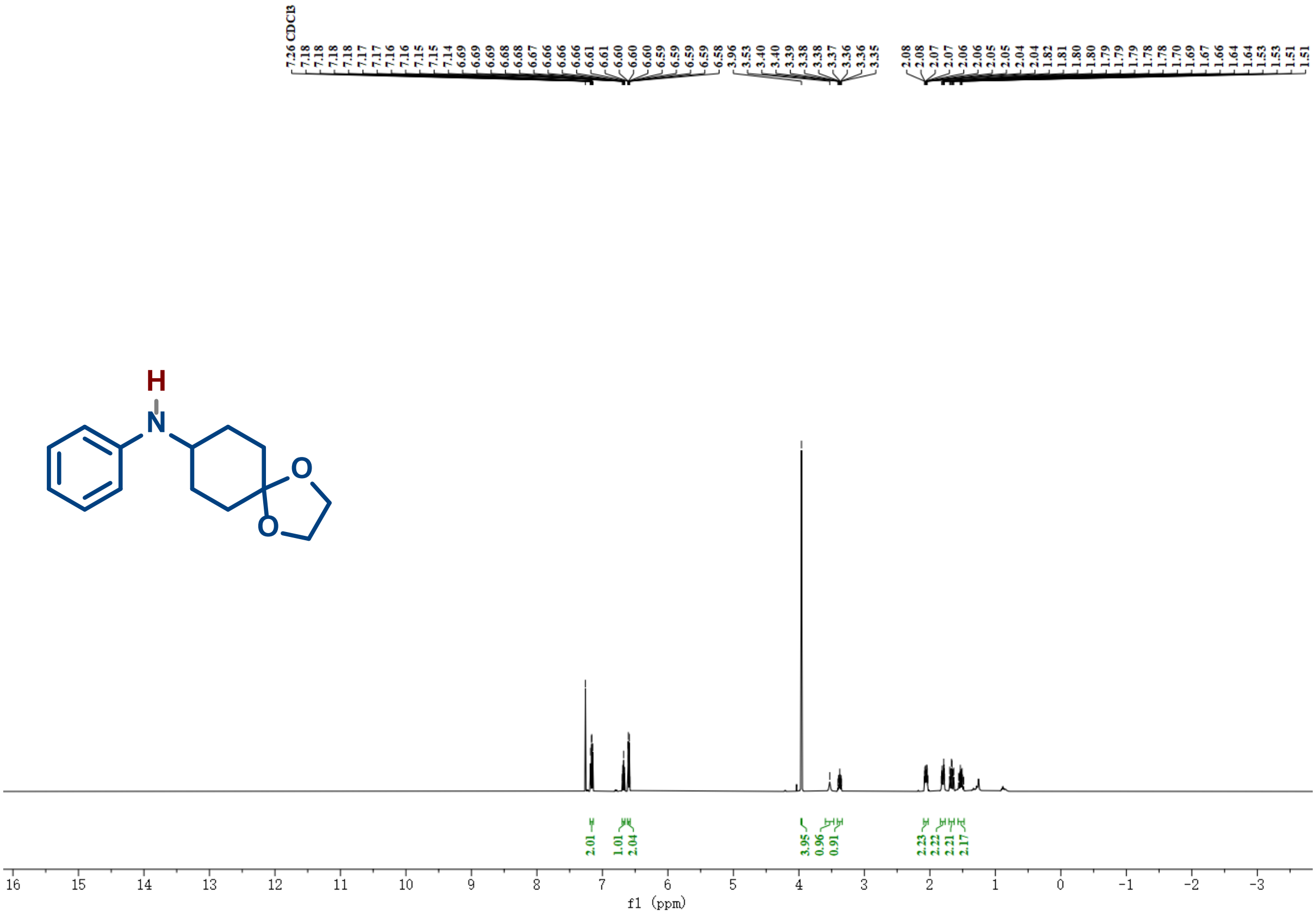

^{1}H NMR (500 MHz, CDCl$_3$)

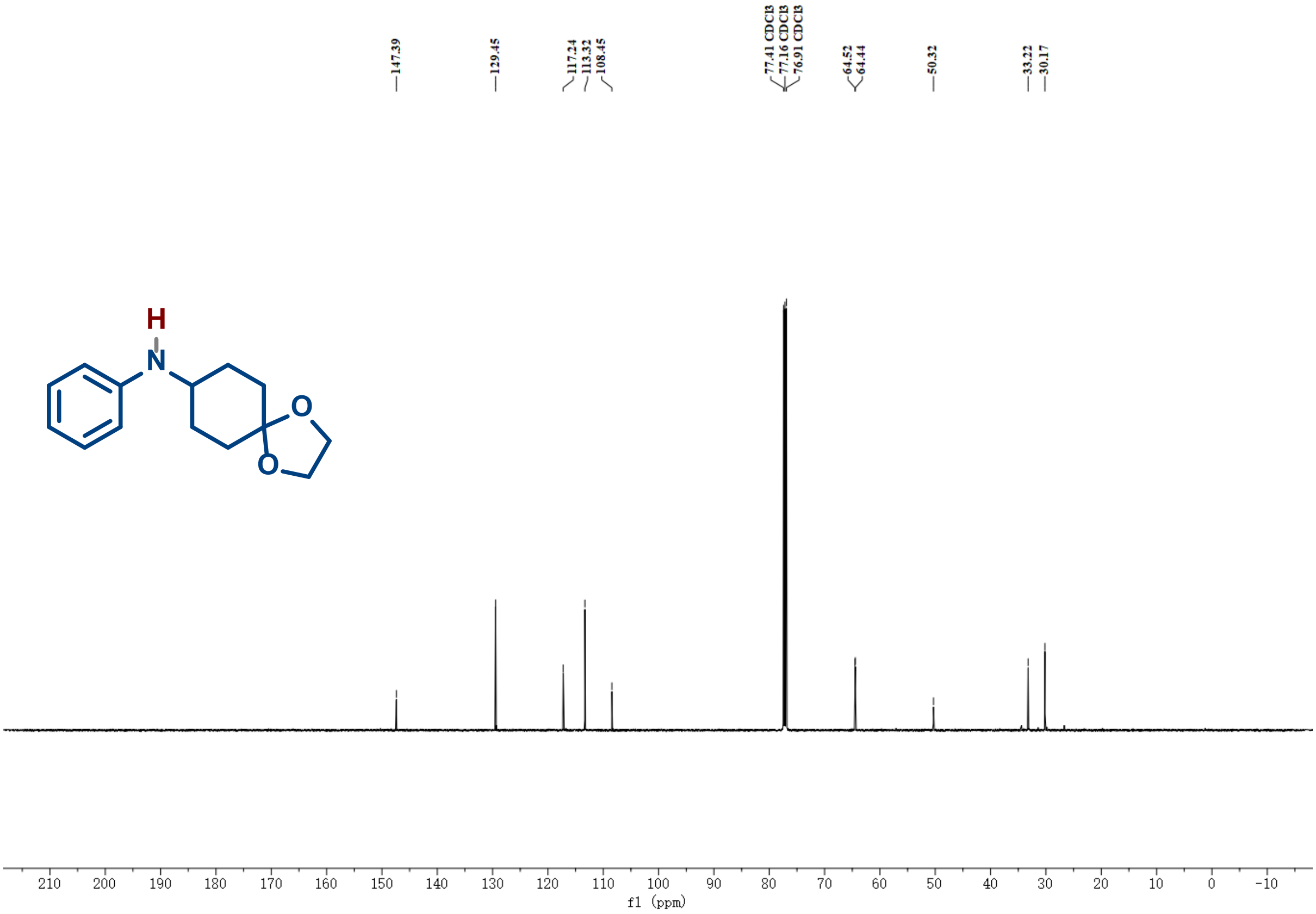

^{13}C NMR (126 MHz, CDCl$_3$)

4-(phenylamino)cyclohexan-1-one (27)

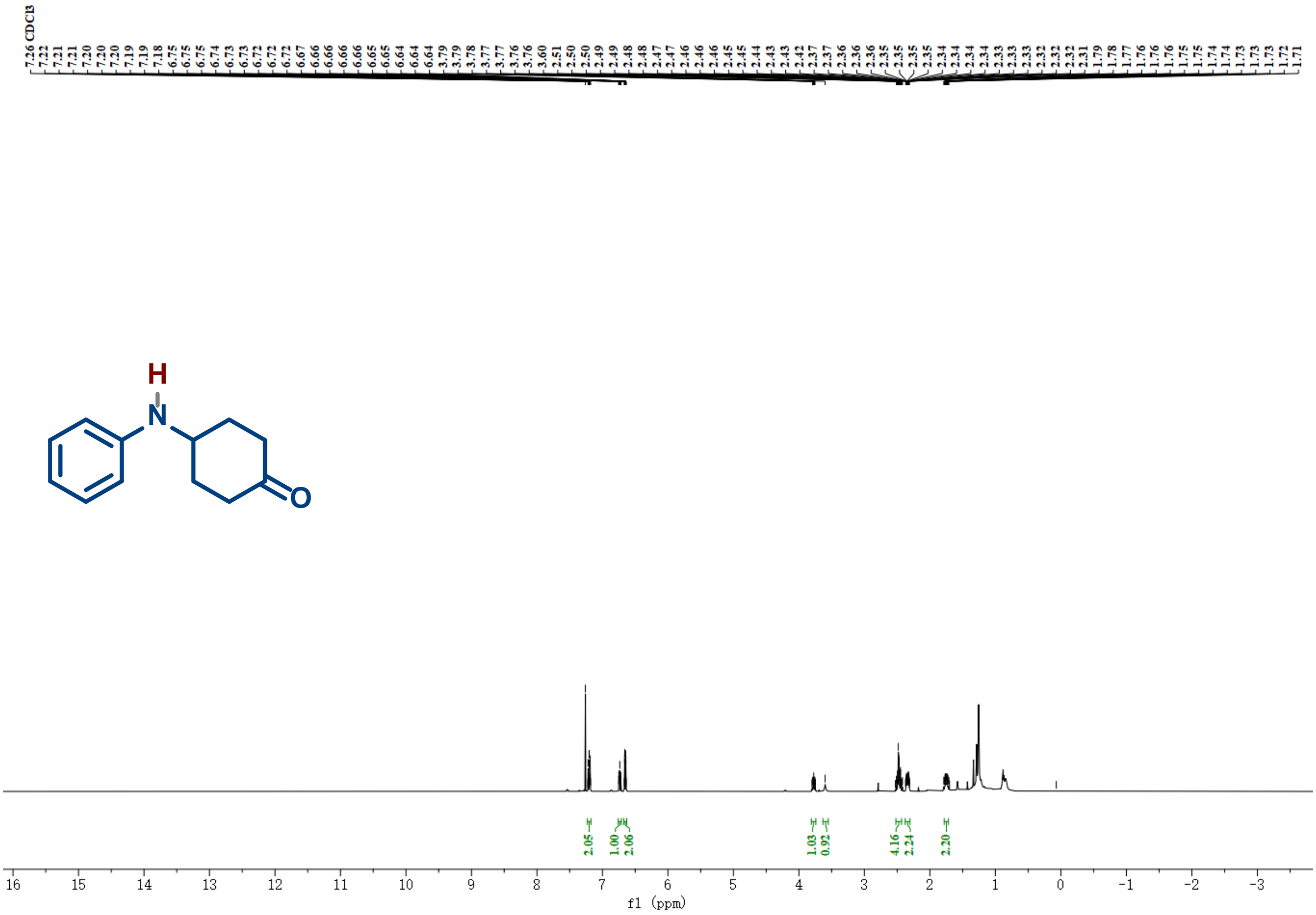

^{1}H NMR (500 MHz, $CDCl_3$)

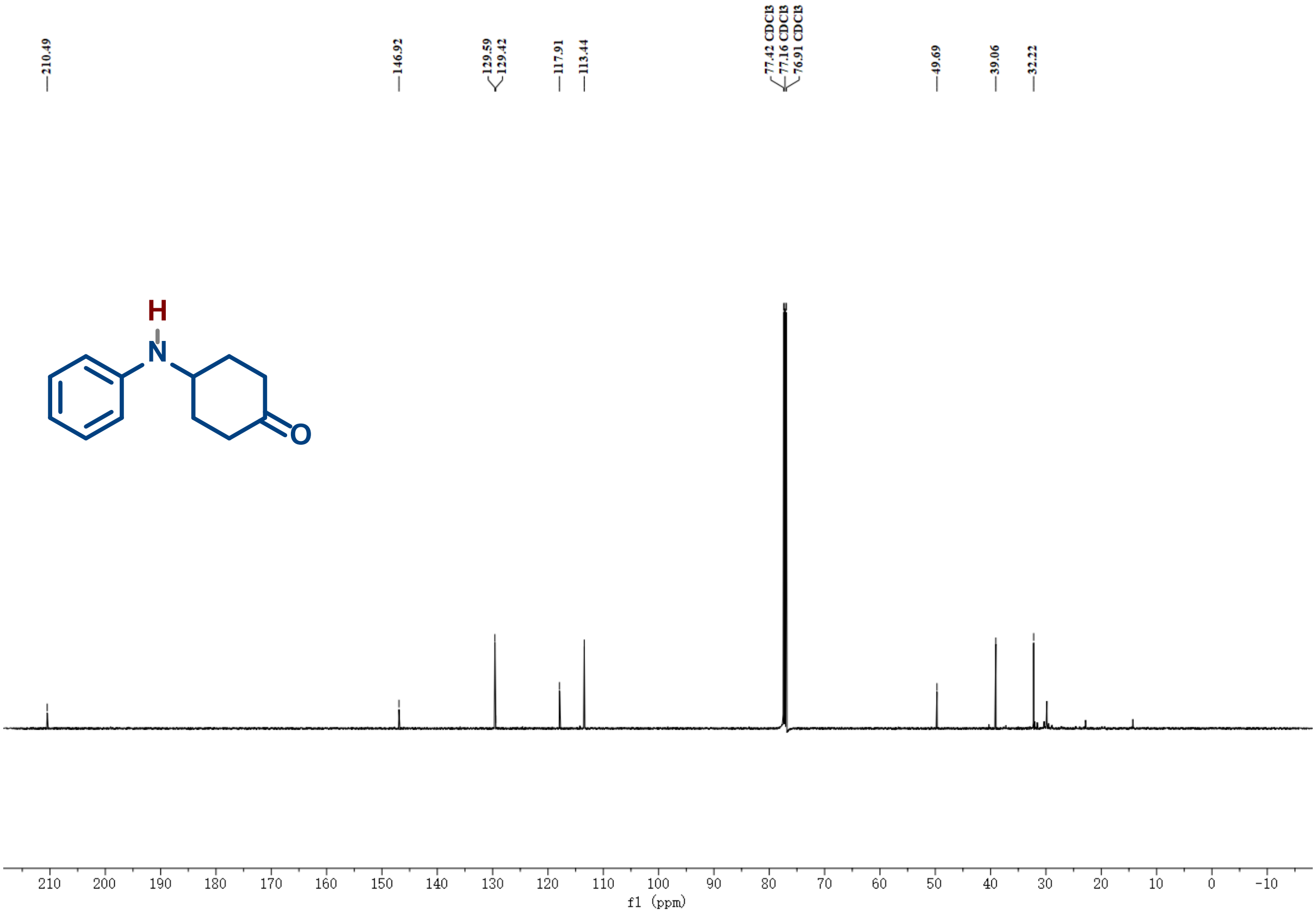

^{13}C NMR (126 MHz, $CDCl_3$)

9*H*-carbazole (28)

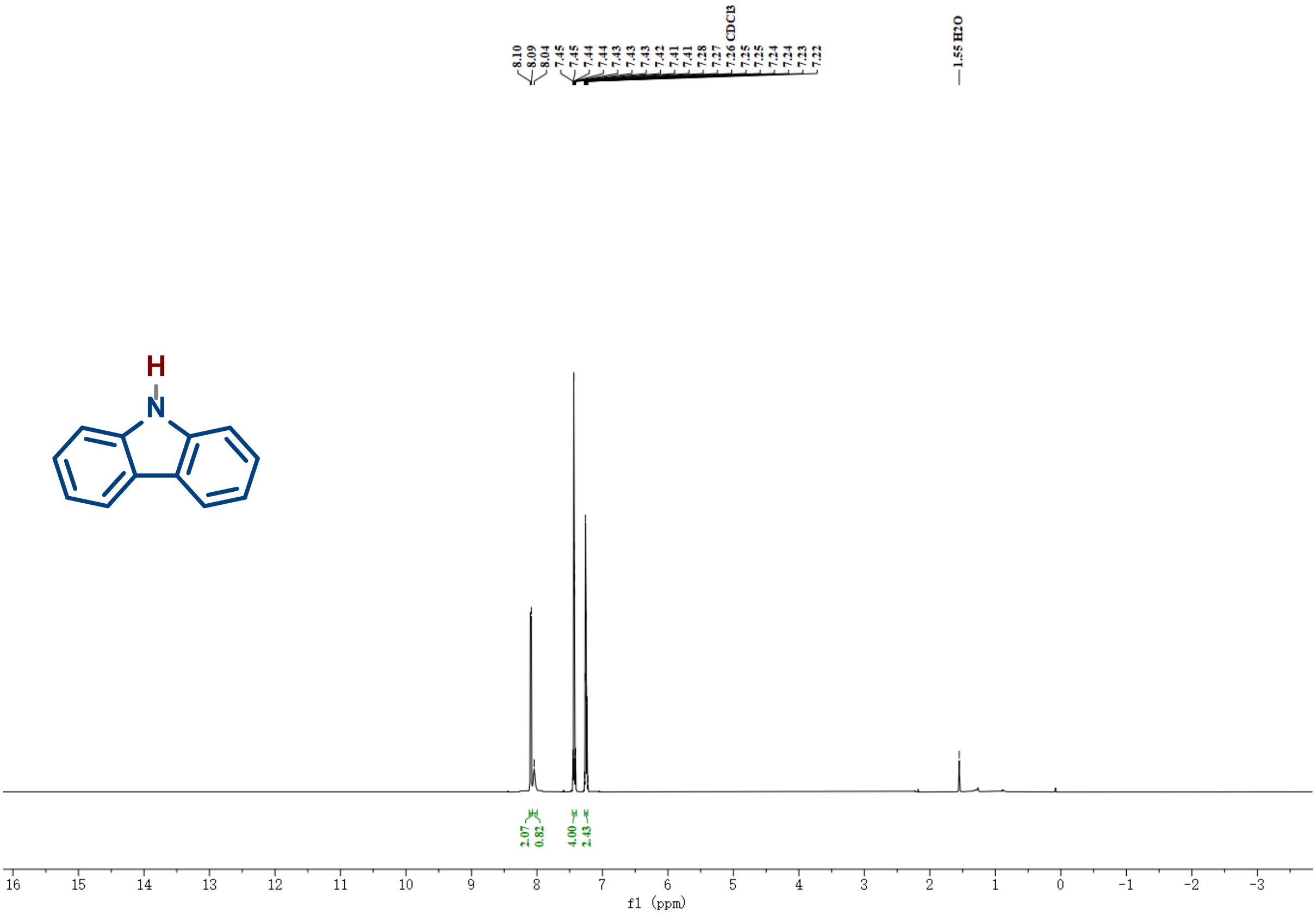

^{1}H NMR (500 MHz, $CDCl_3$)

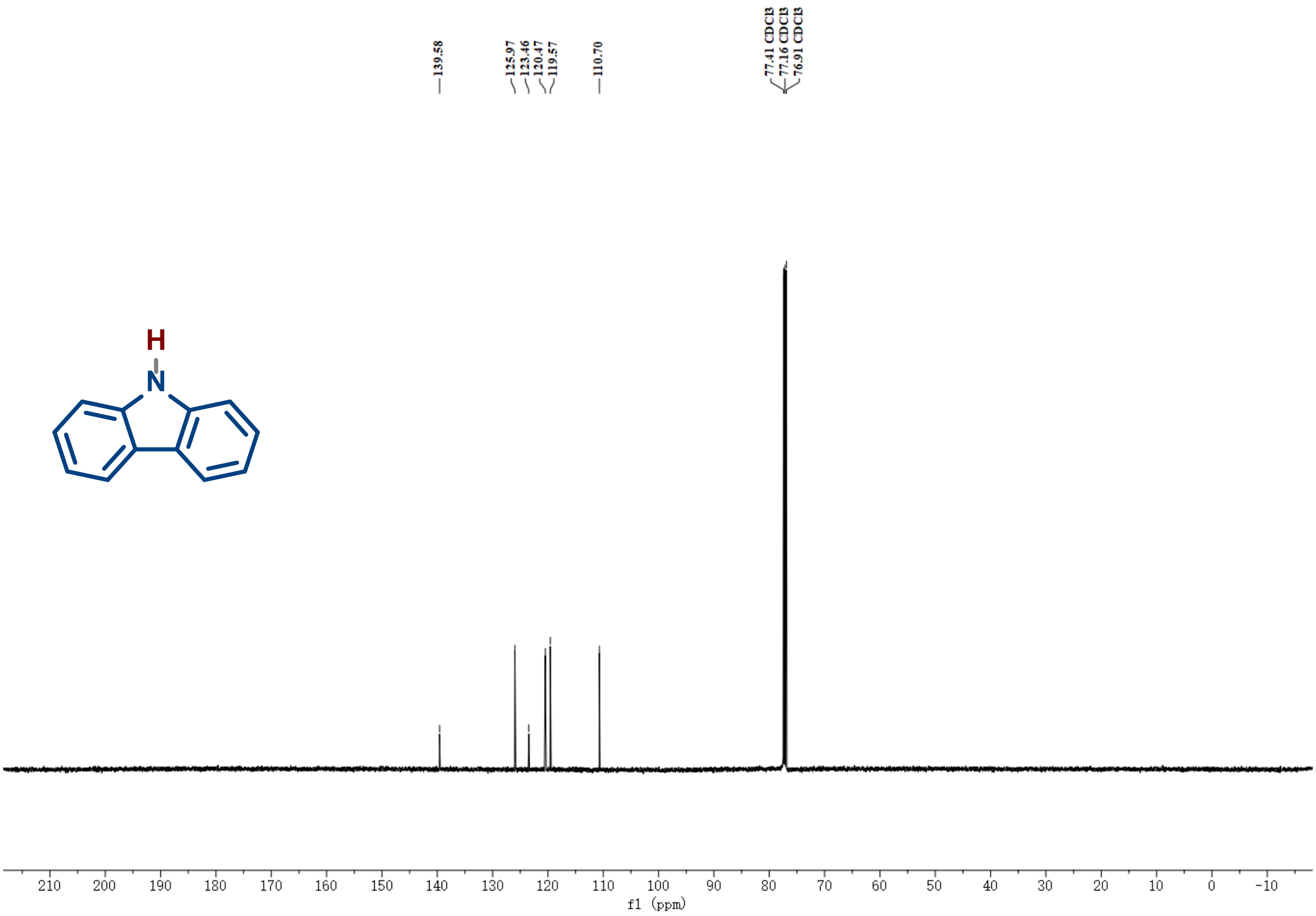

^{13}C NMR (126 MHz, $CDCl_3$)

diphenylamine (29)

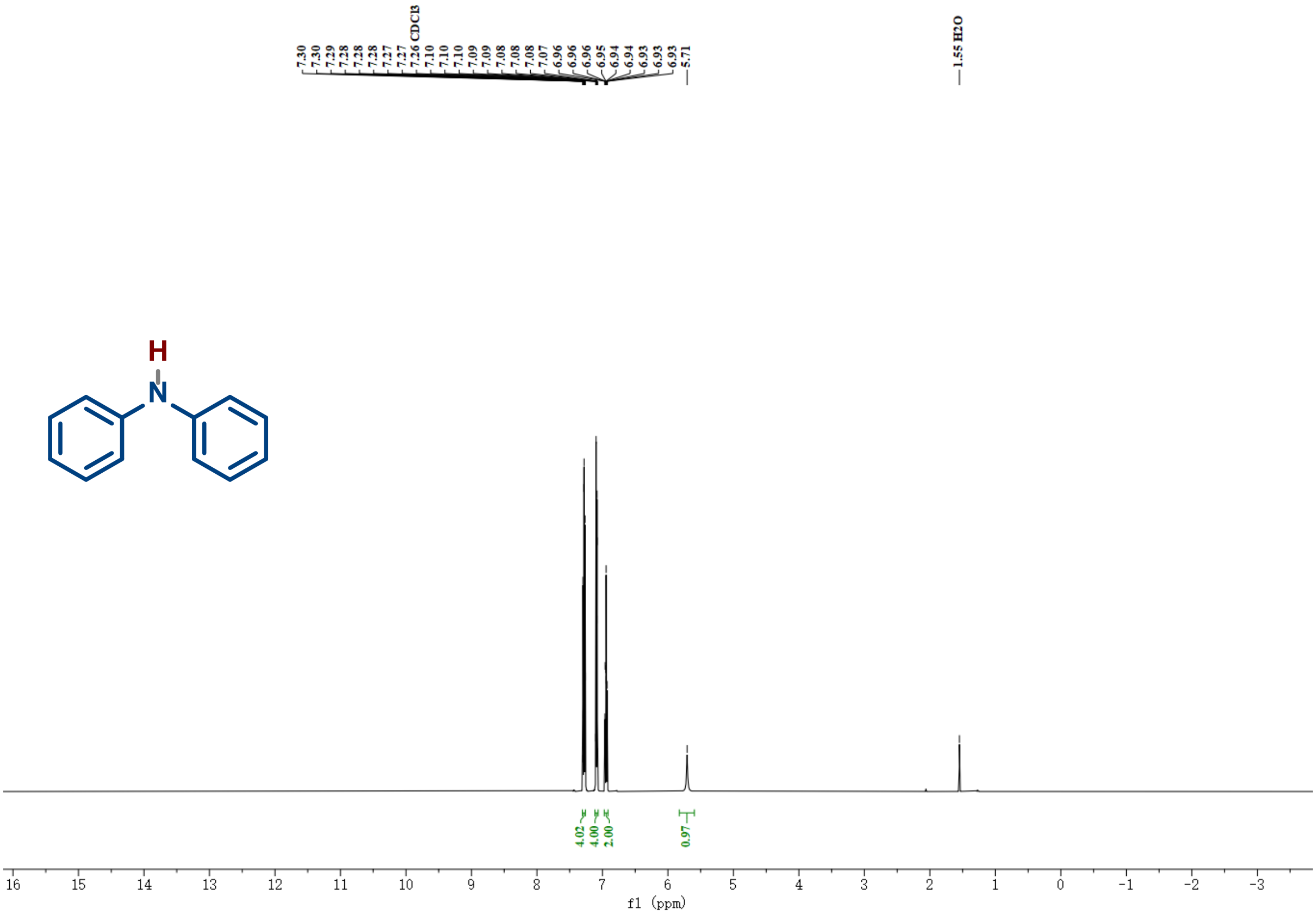

^{1}H NMR (500 MHz, $CDCl_3$)

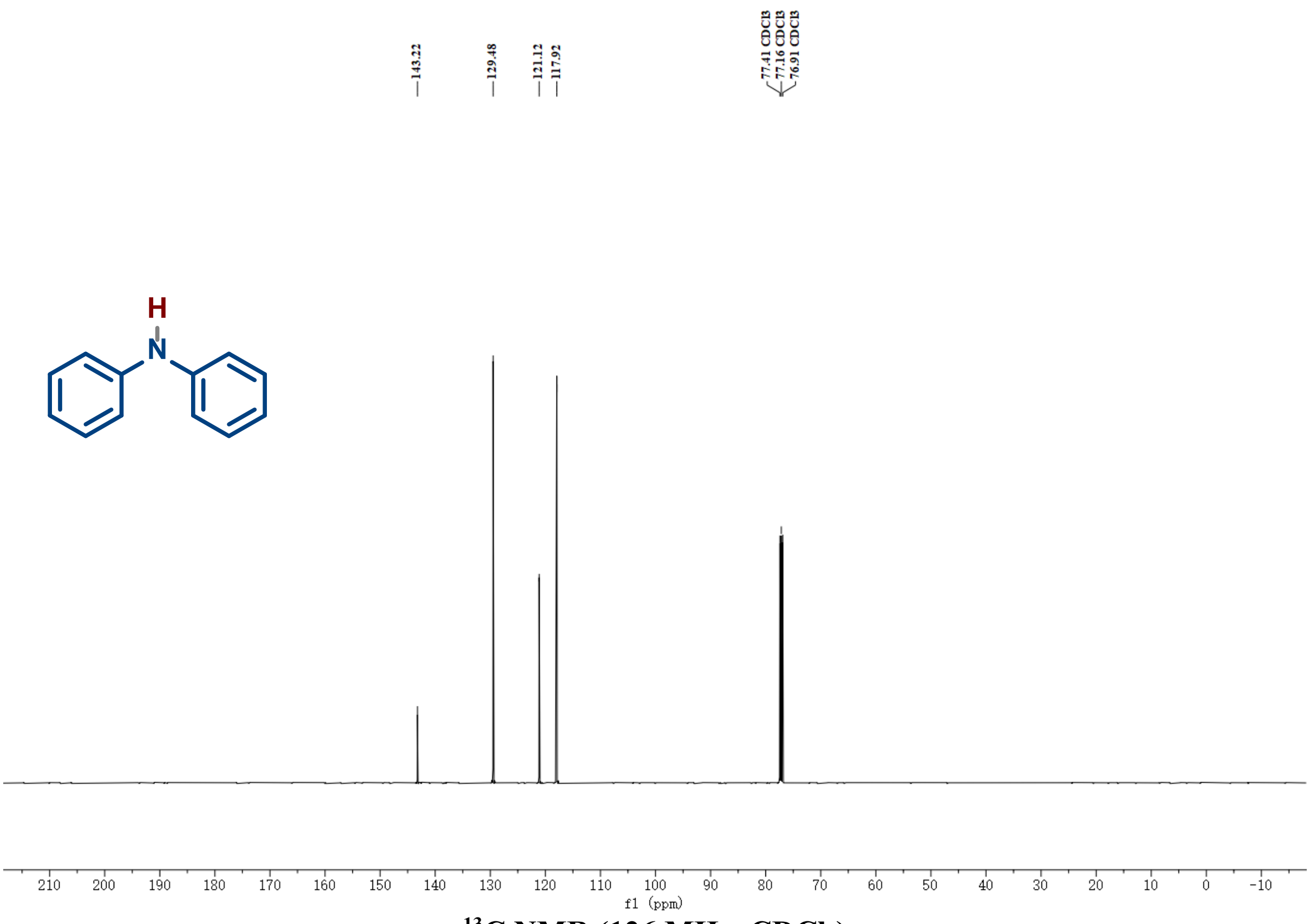

^{13}C NMR (126 MHz, $CDCl_3$)

bis(4-methoxyphenyl)amine (30)

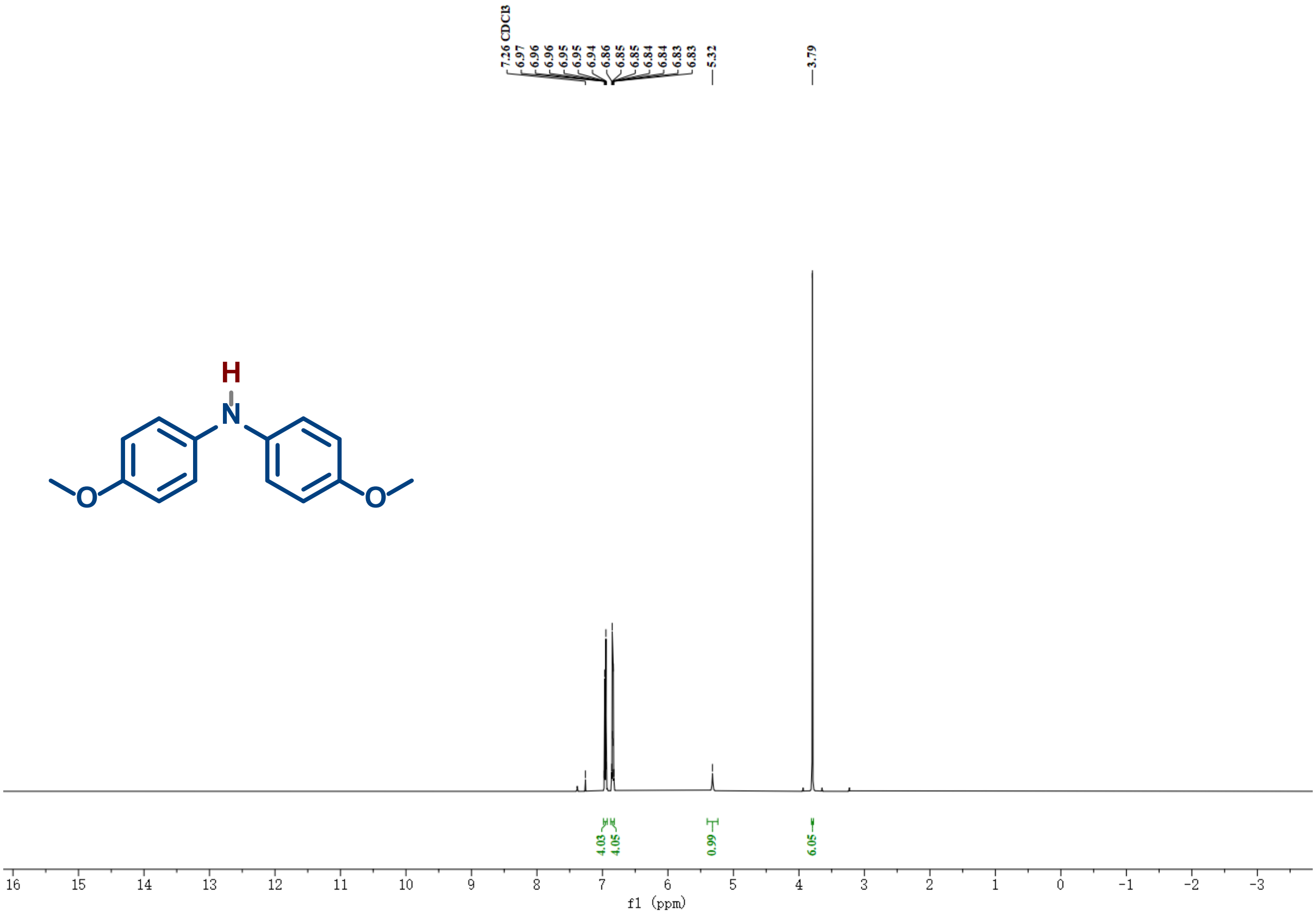

^{1}H NMR (500 MHz, CDCl$_3$)

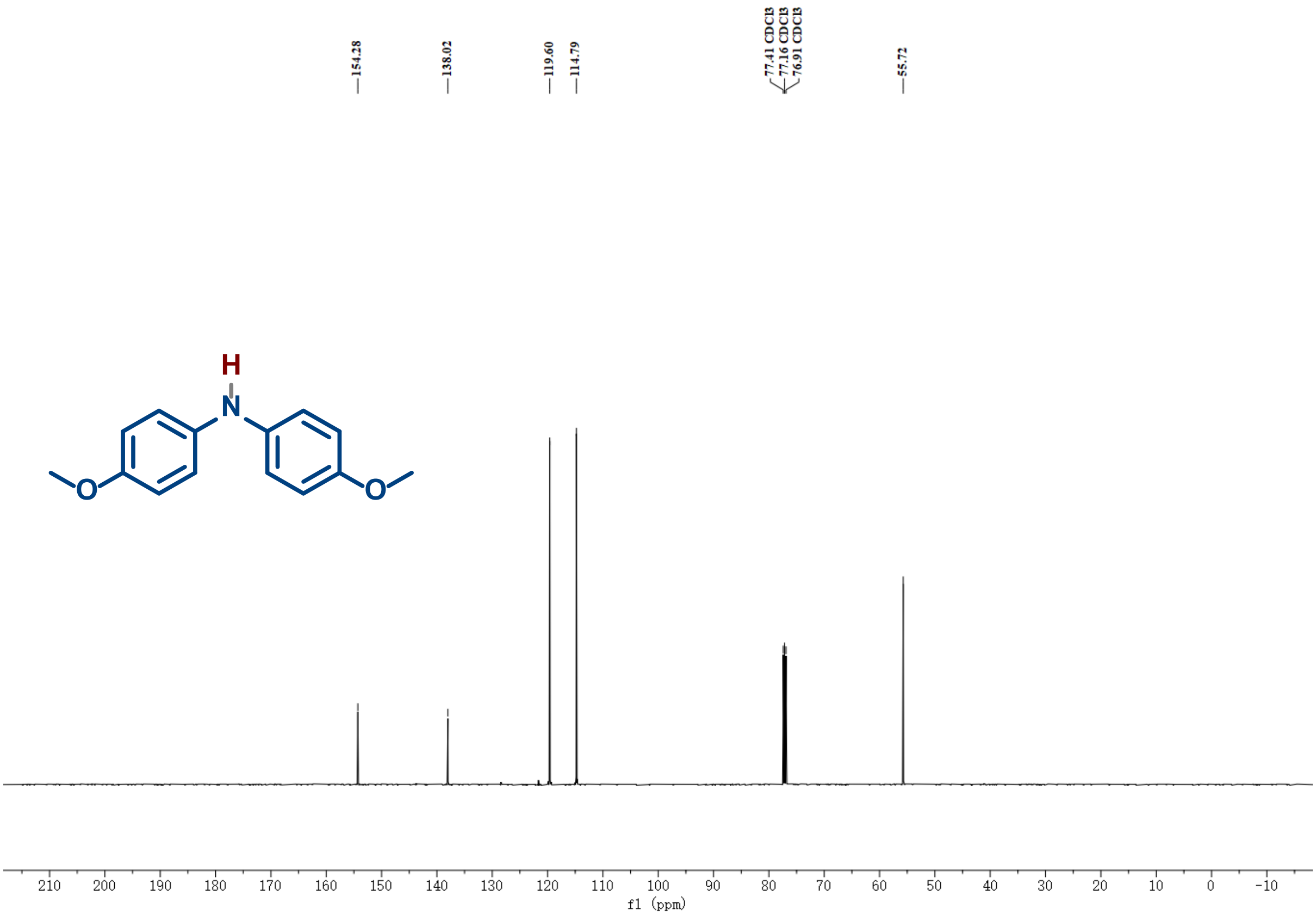

^{13}C NMR (126 MHz, CDCl$_3$)

4-methyl-*N*-phenylaniline (31)

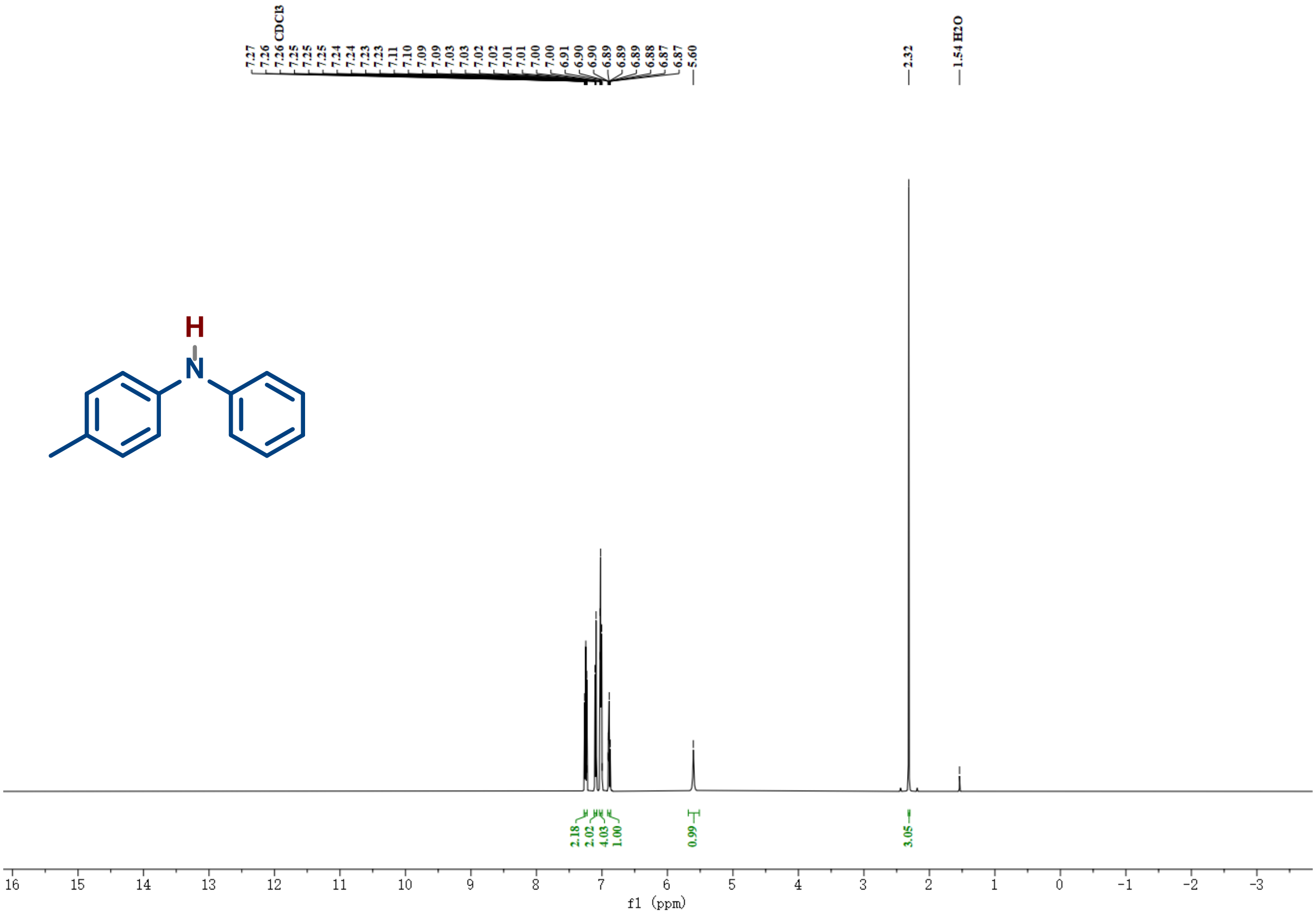

^{1}H NMR (500 MHz, CDCl$_3$)

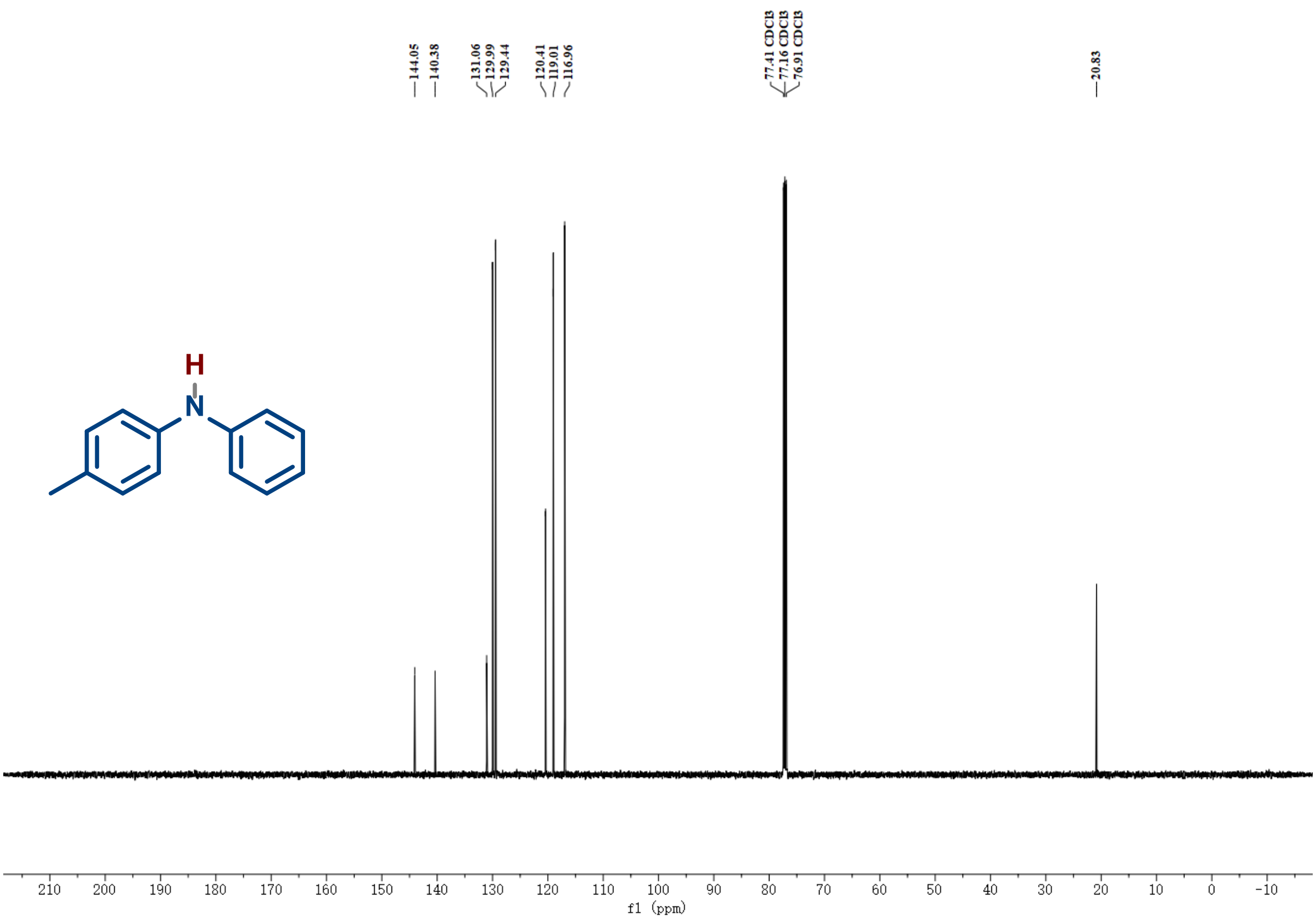

^{13}C NMR (126 MHz, CDCl$_3$)

acridin-9(10*H*)-one (32)

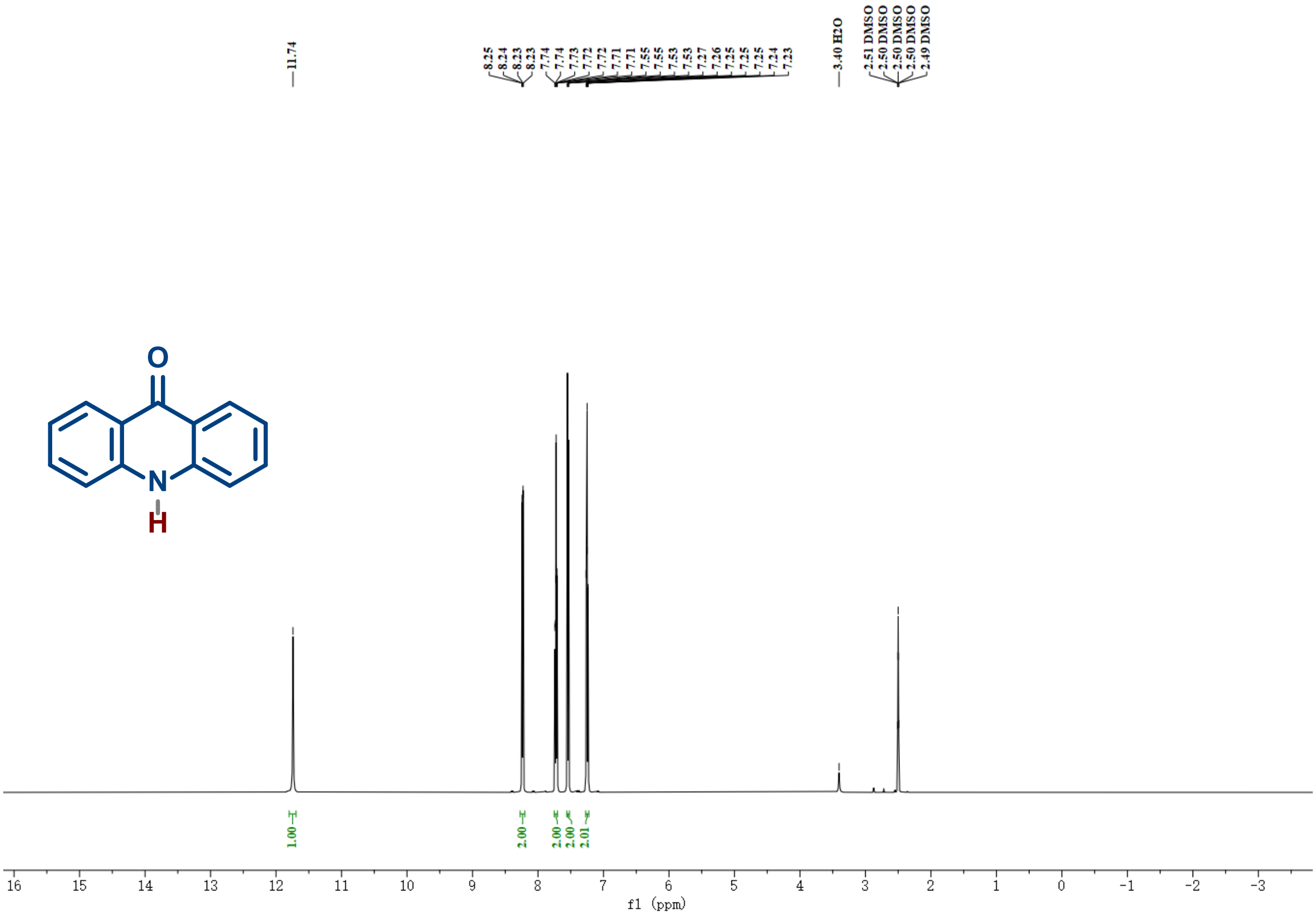

^{1}H NMR (500 MHz, DMSO)

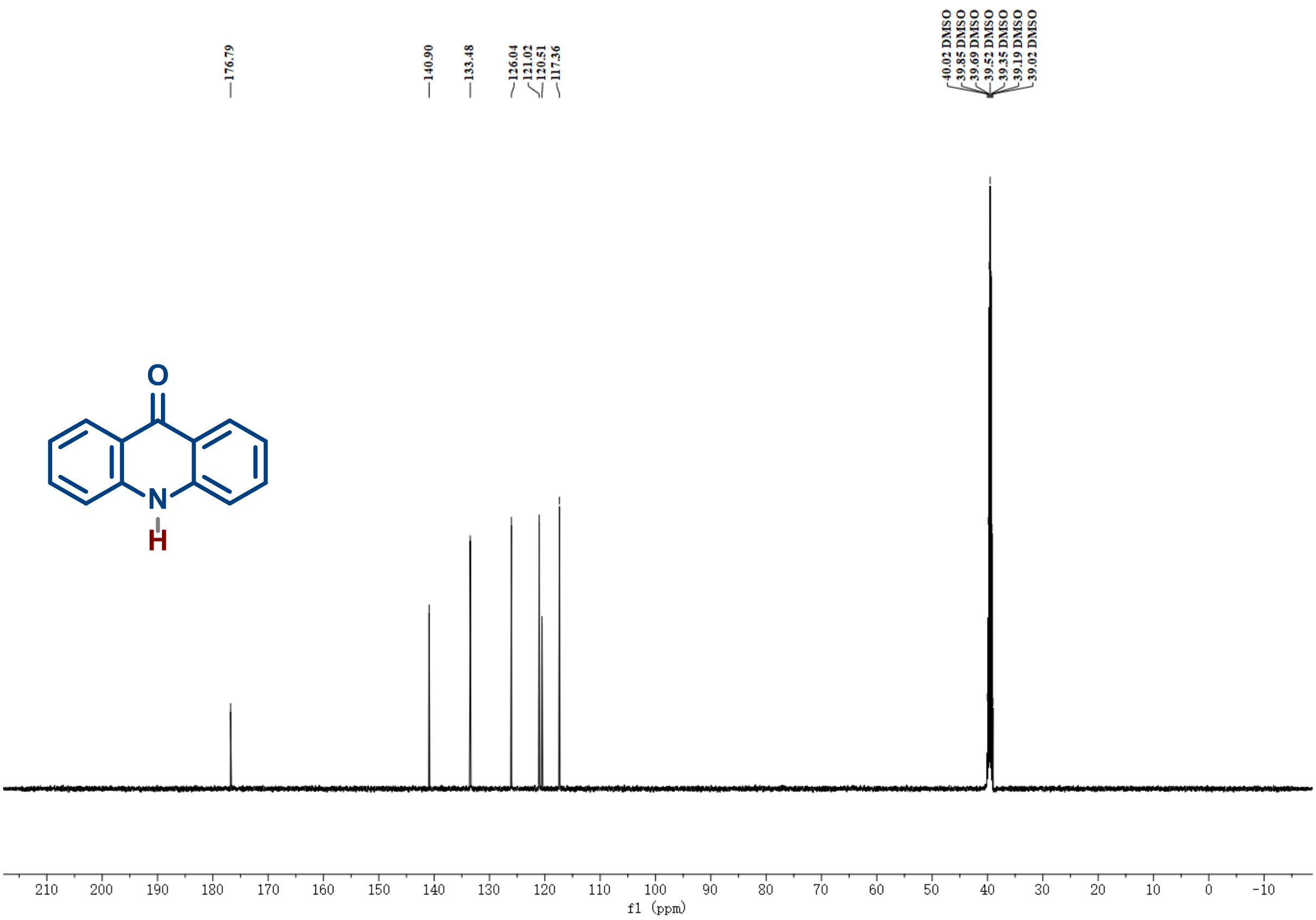

^{13}C NMR (126 MHz, DMSO)

***N*-phenylcycloheptanamine (33)**

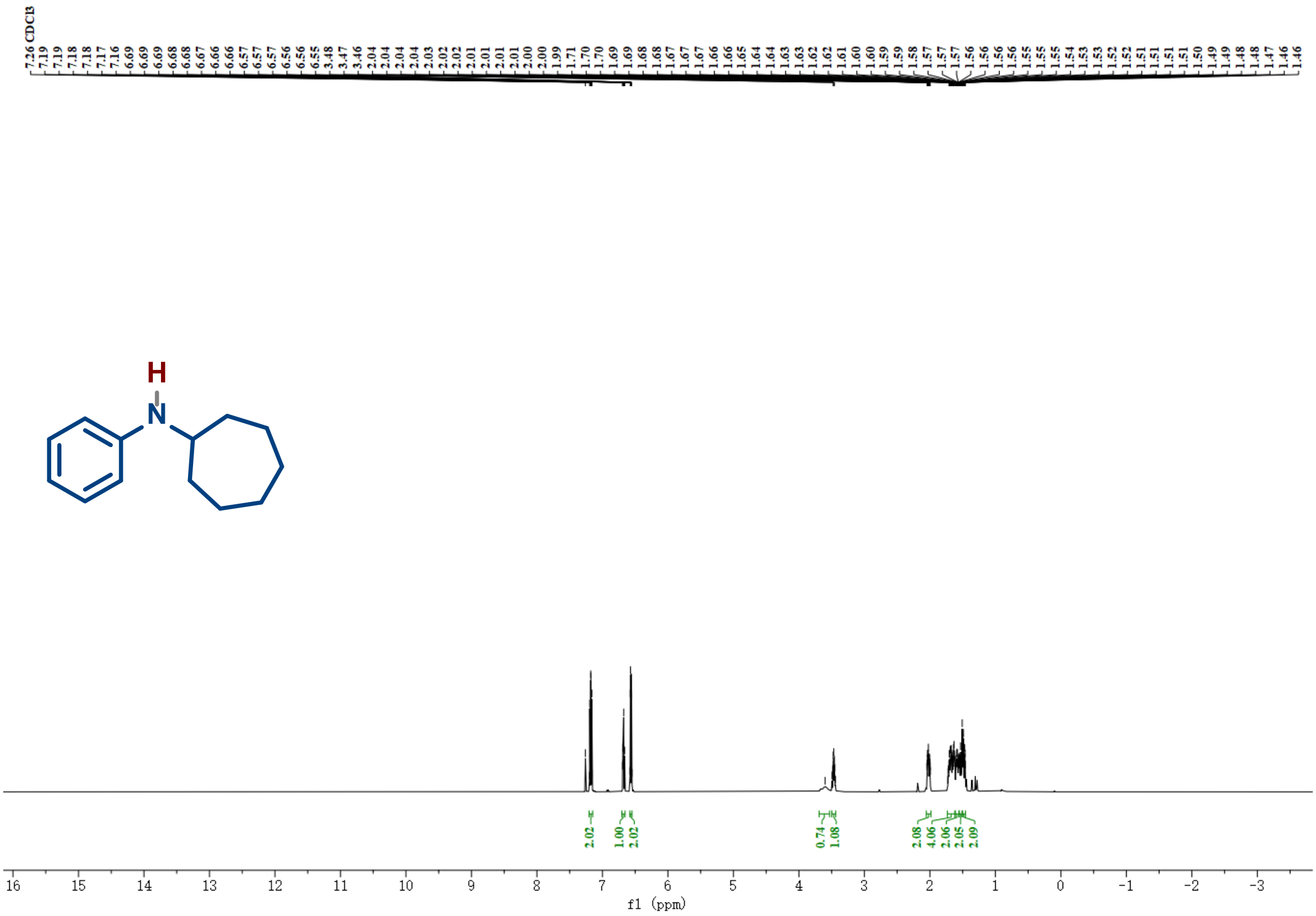

^{1}H NMR (500 MHz, CDCl$_3$)

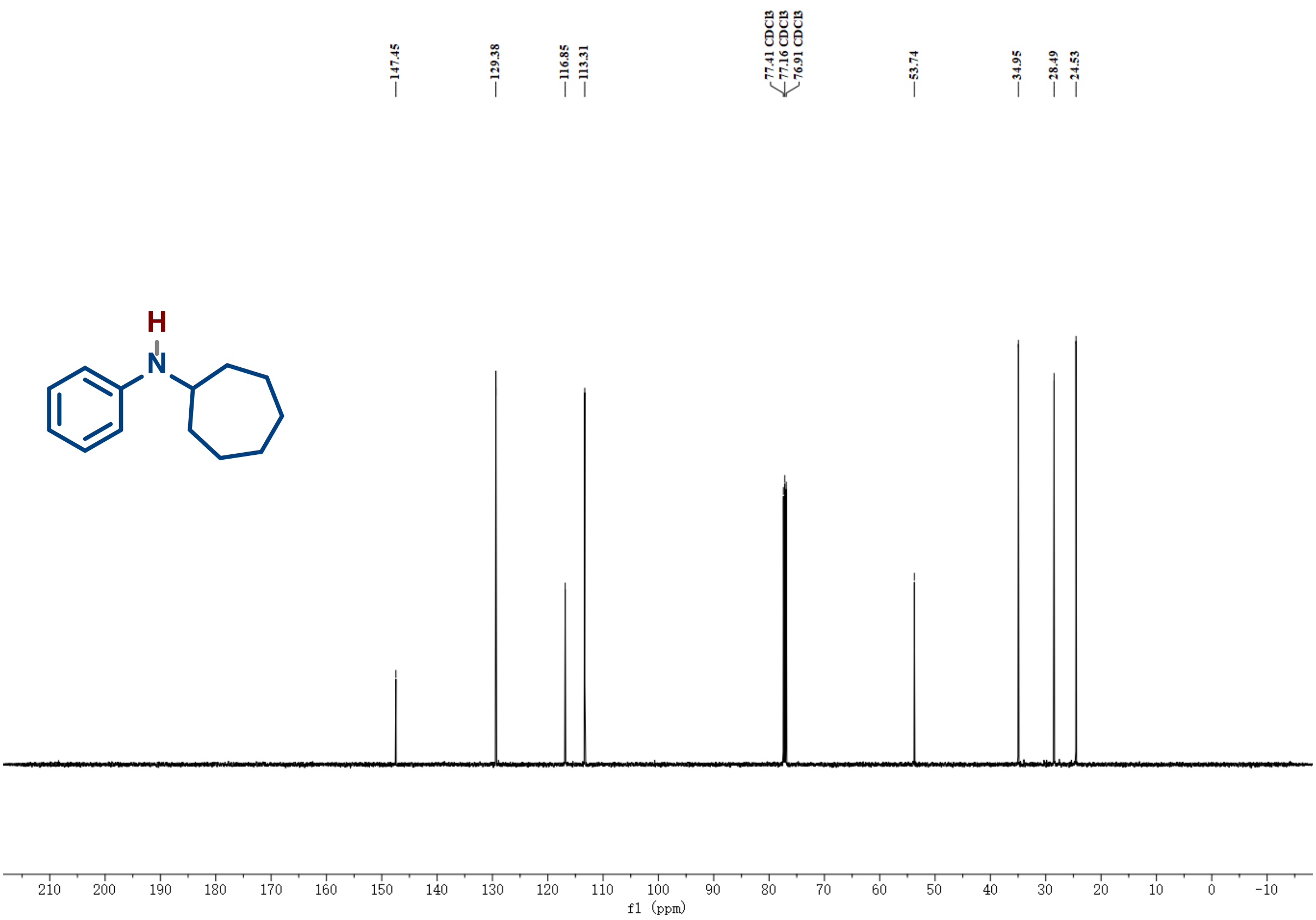

^{13}C NMR (126 MHz, CDCl$_3$)

10,11-dihydro-5*H*-dibenzo[*b*,*f*]azepine (34)

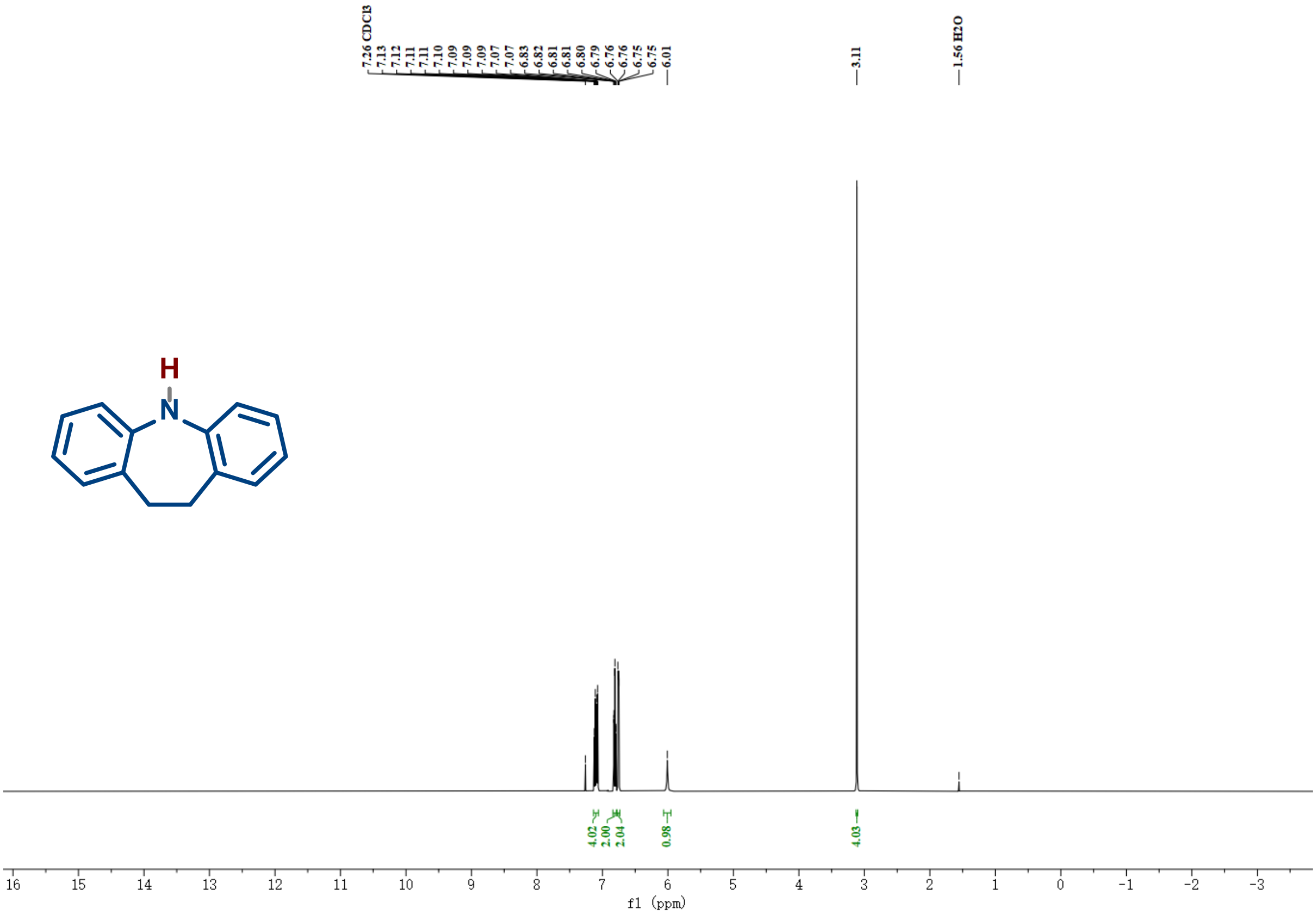

^{1}H NMR (500 MHz, $CDCl_3$)

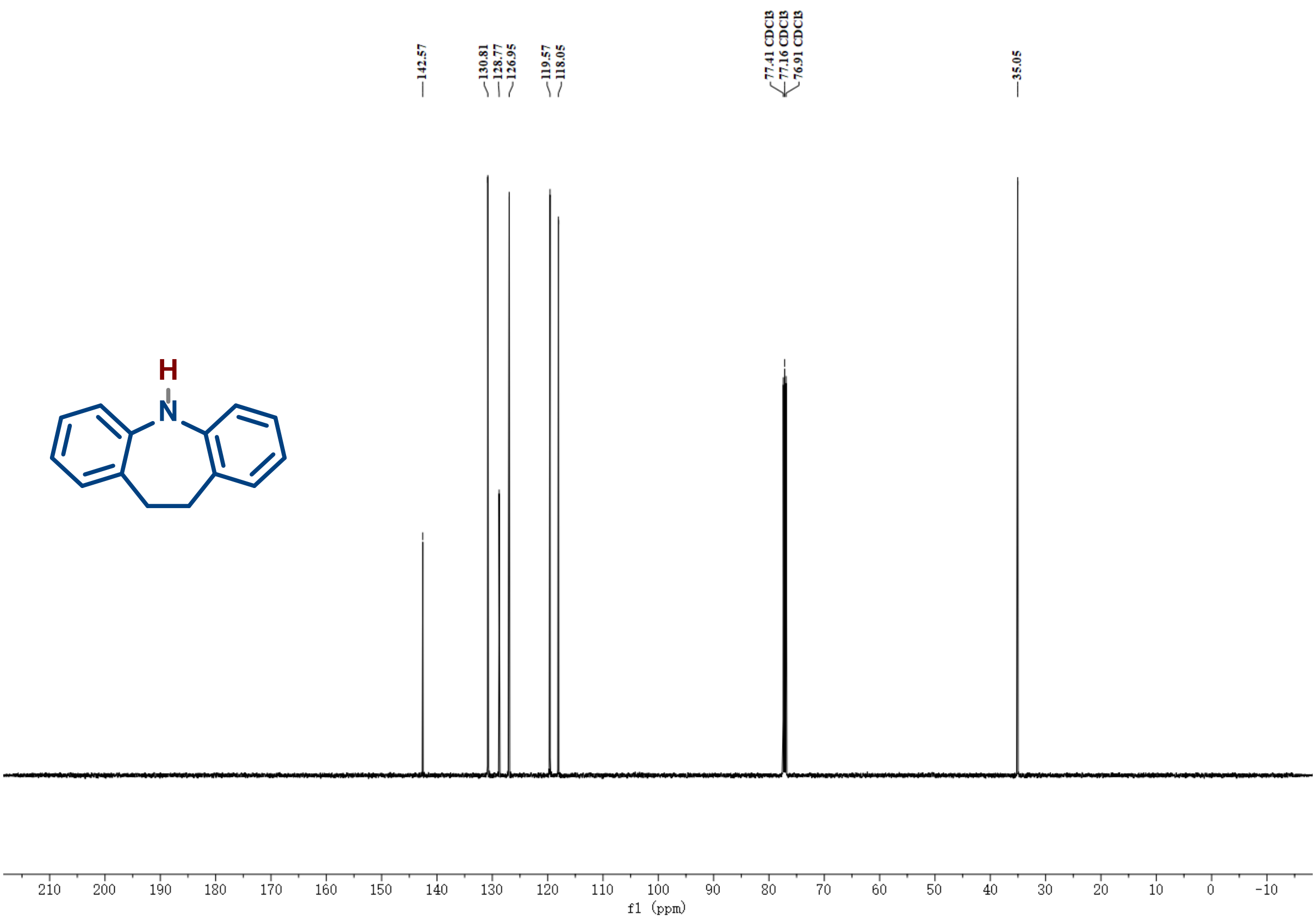

^{13}C NMR (126 MHz, $CDCl_3$)

2,4-dinitro-*N*-phenylaniline (35)

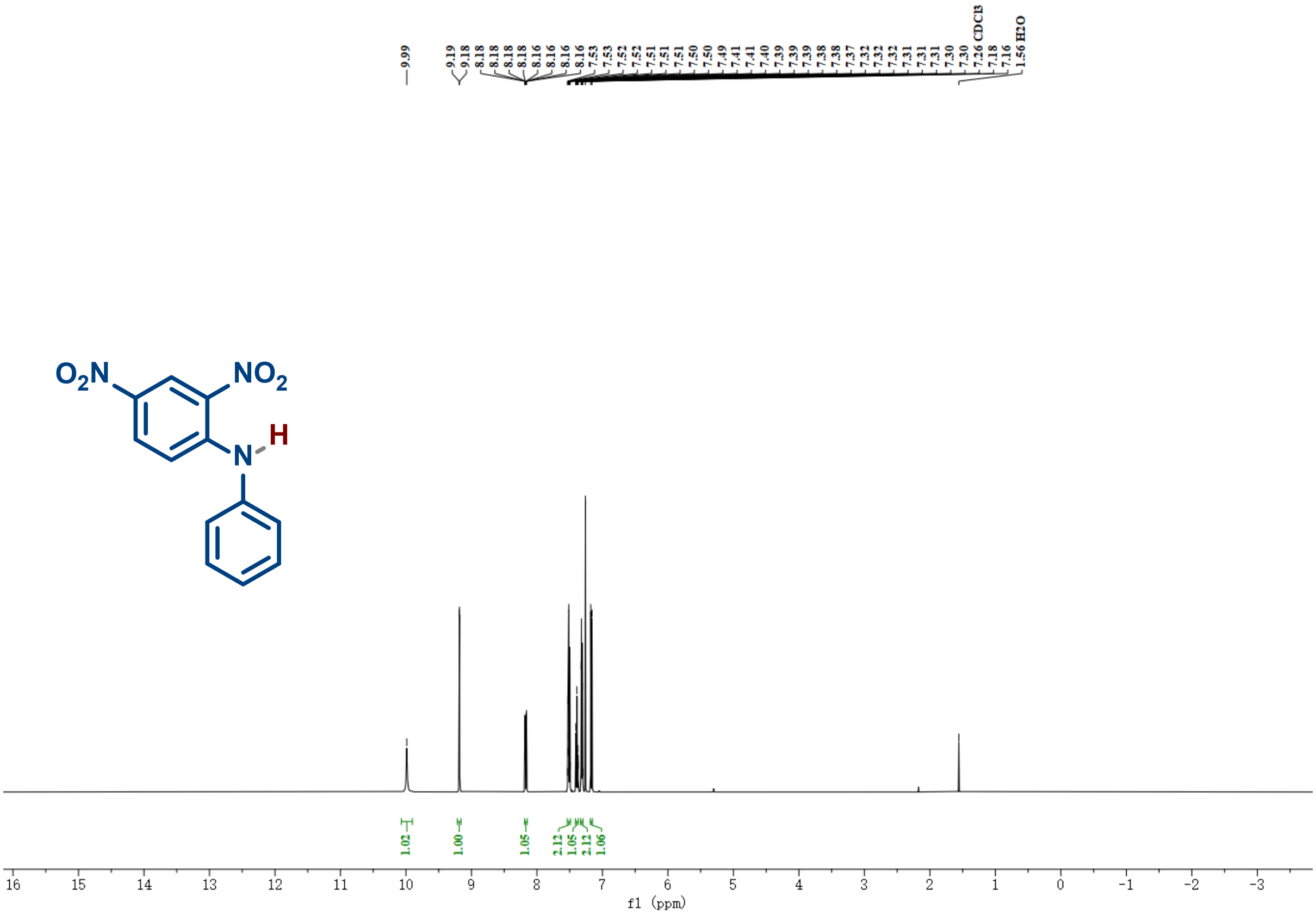

^{1}H NMR (500 MHz, CDCl$_3$)

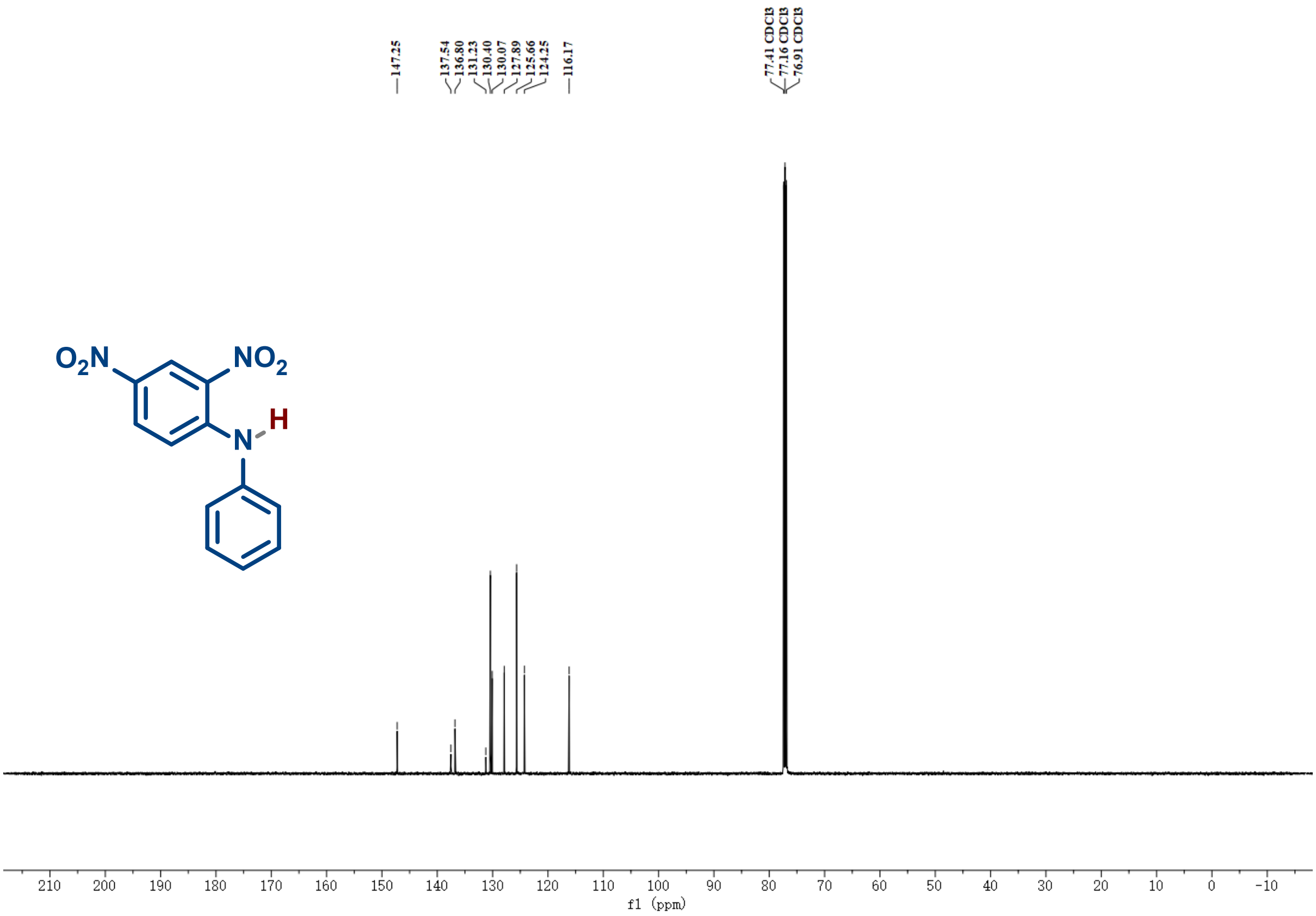

^{13}C NMR (126 MHz, CDCl$_3$)

_N_-phenylnaphthalen-1-amine (36)

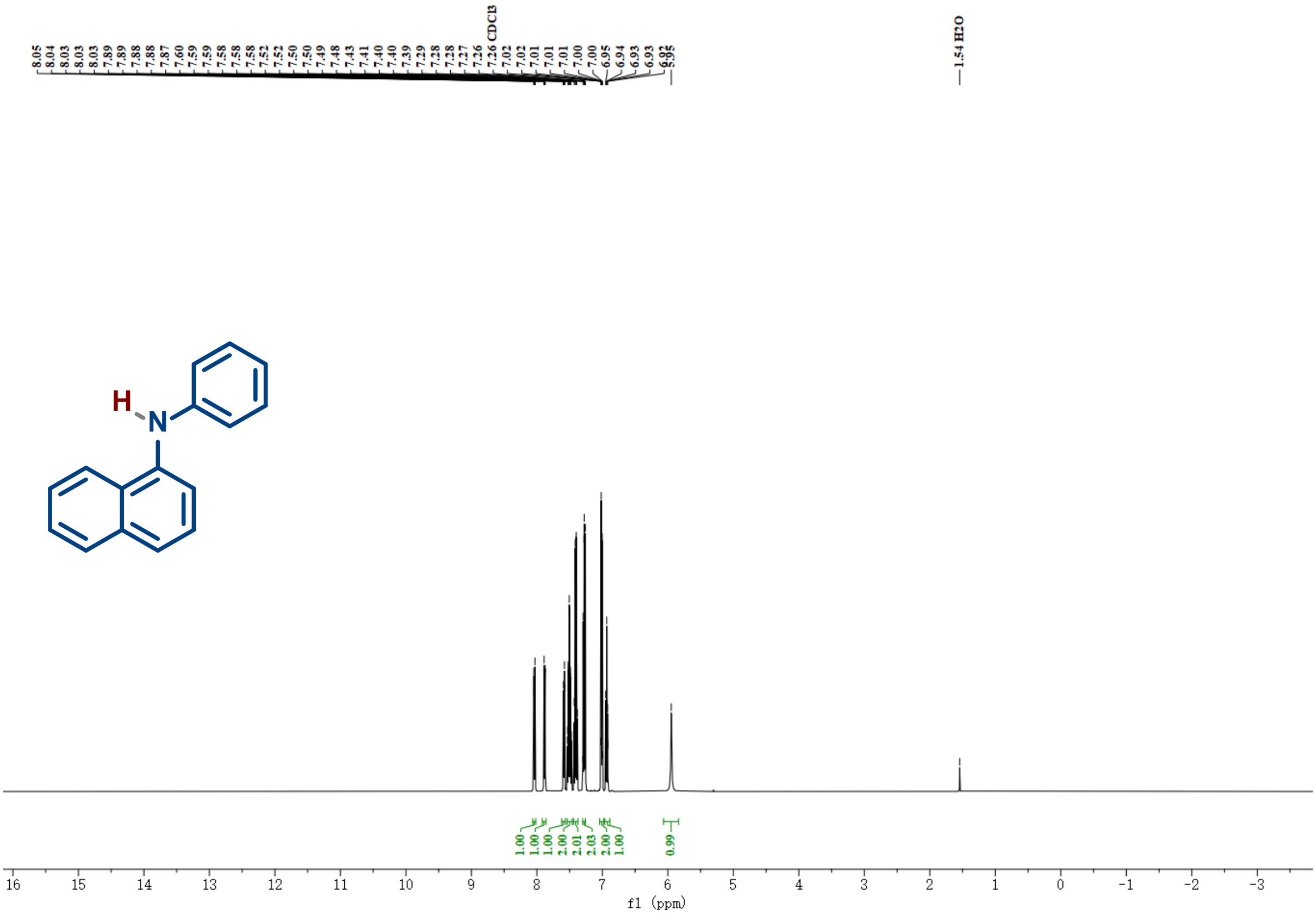

^{1}H NMR (500 MHz, CDCl$_3$)

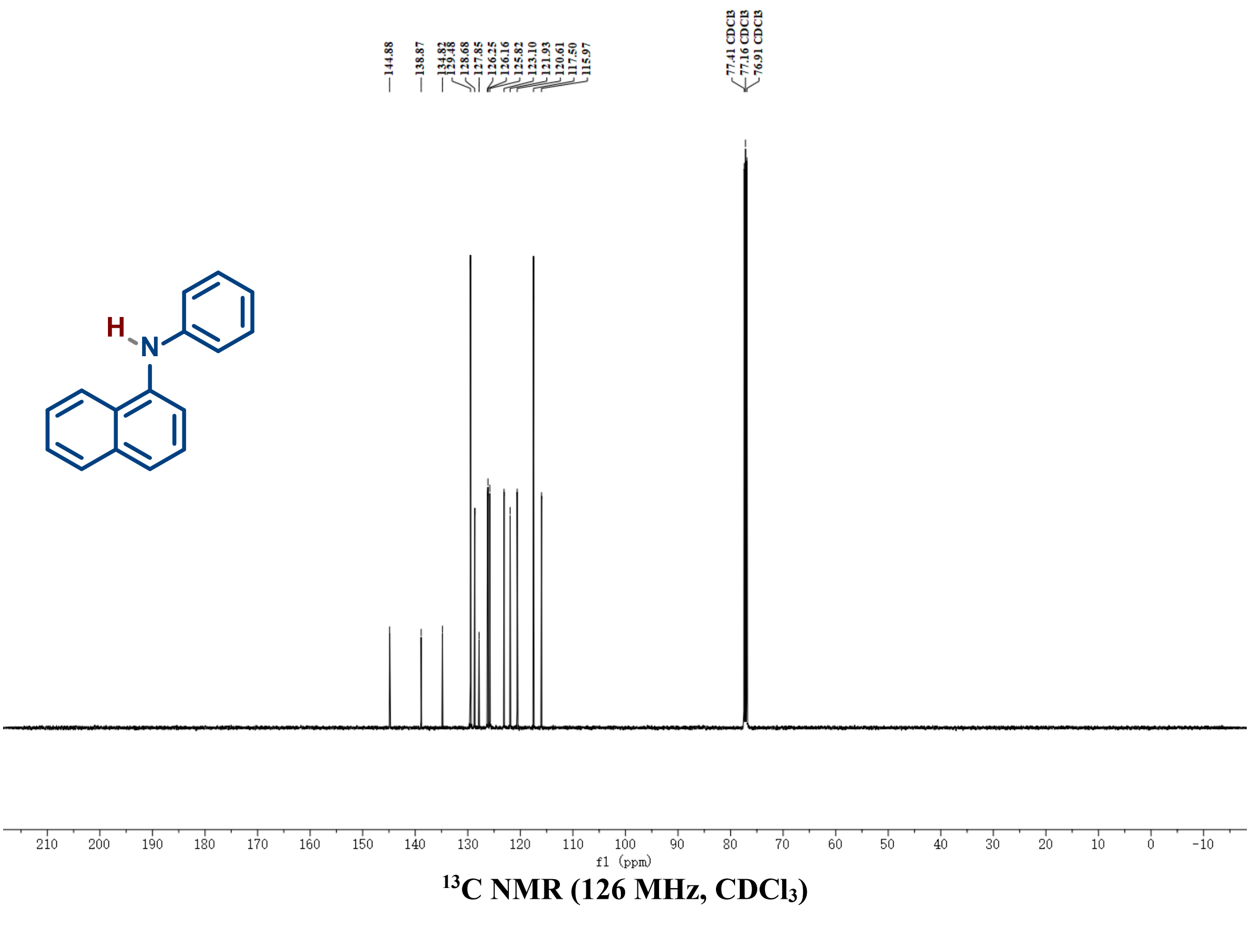

^{13}C NMR (126 MHz, CDCl$_3$)

9*H*-pyrido[3,4-*b*]indole (37)

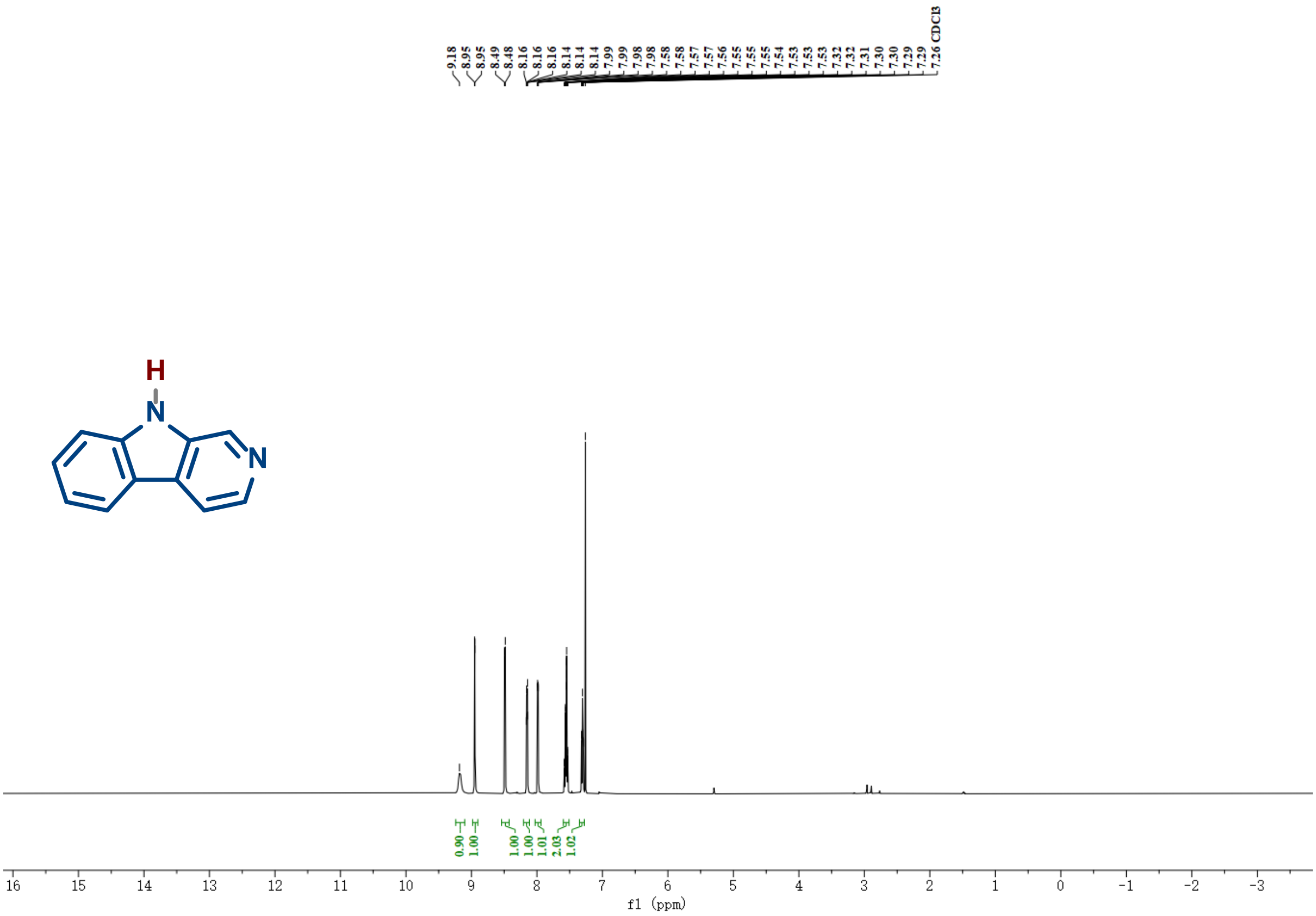

^{1}H NMR (500 MHz, $CDCl_3$)

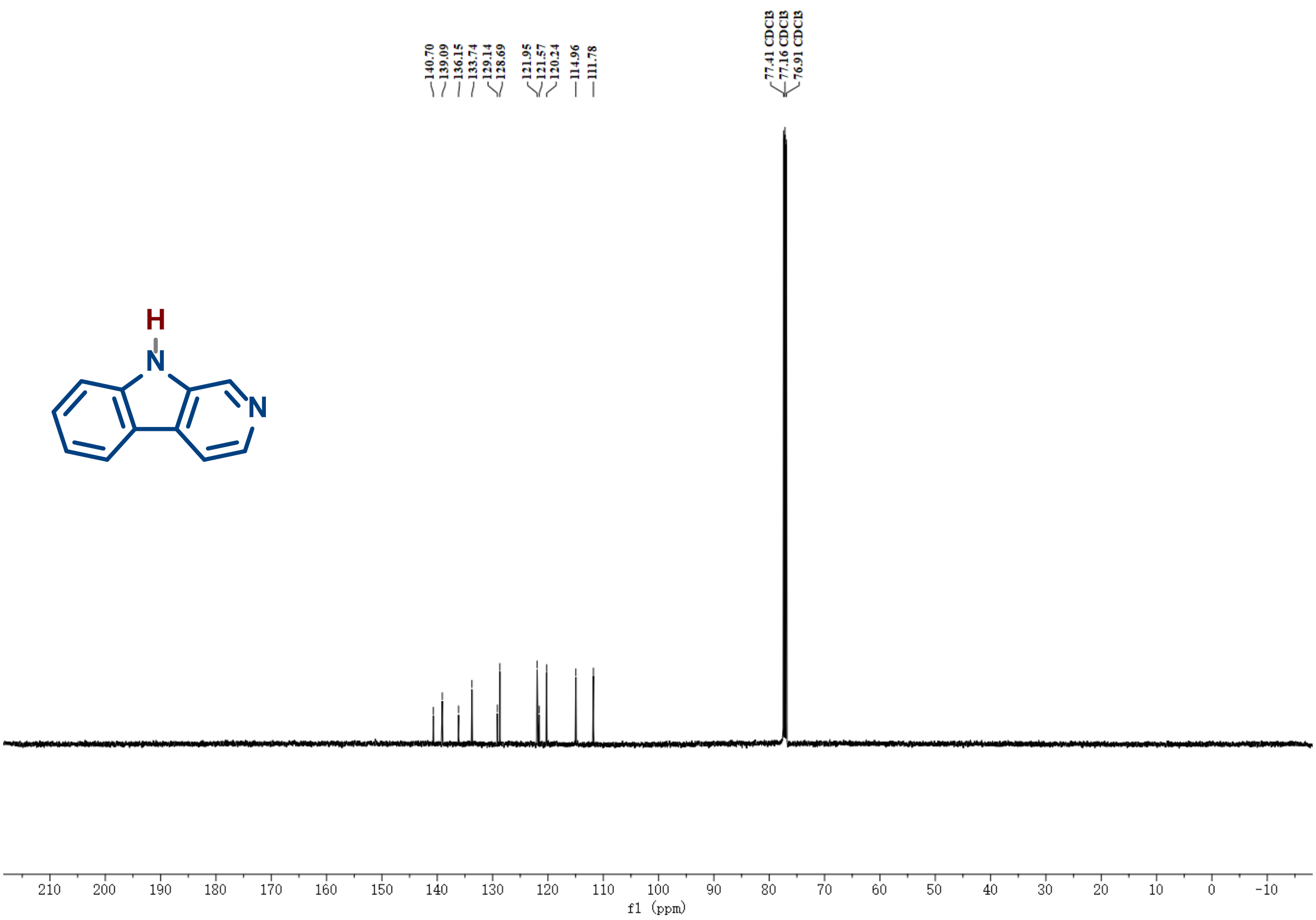

^{13}C NMR (126 MHz, $CDCl_3$)

(5*S*,8*R*,9*S*,10*S*,13*S*,14*S*,17*S*)-10,13-dimethyl-3-(phenylamino)hexadecahydro-1*H*-cyclopenta[a]phenanthren-17-ol (38)

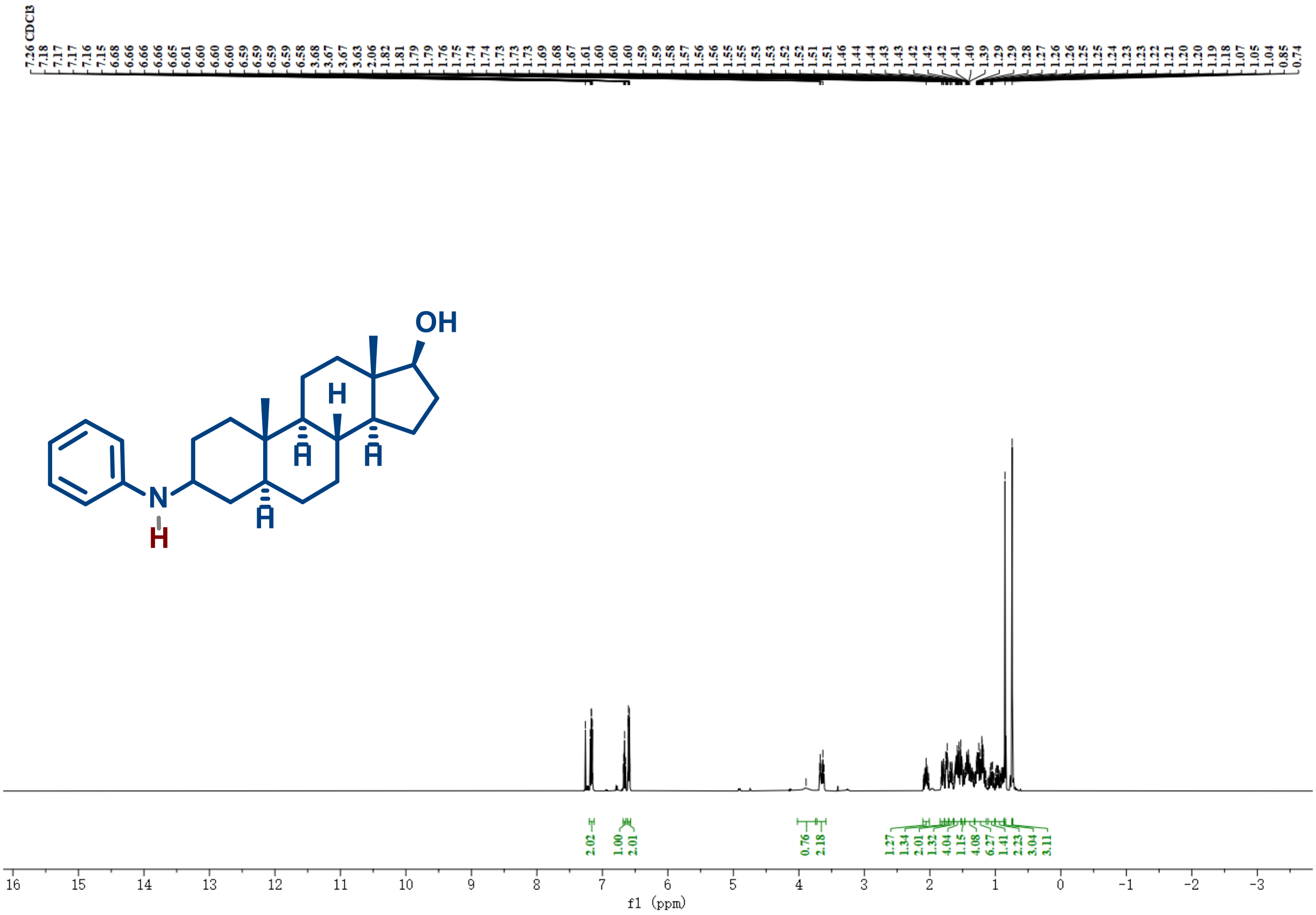

^{1}H NMR (500 MHz, $CDCl_3$)

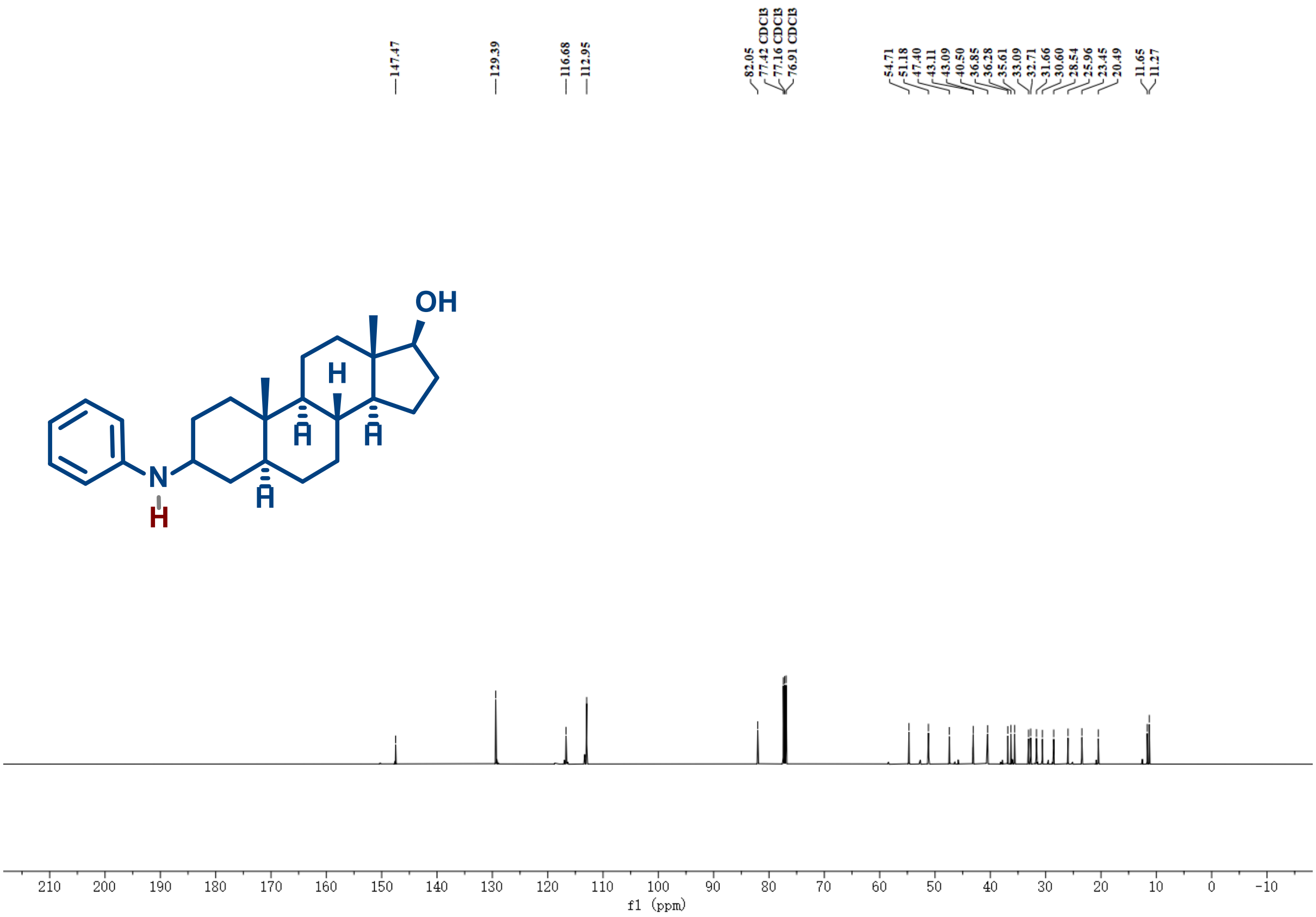

^{13}C NMR (126 MHz, $CDCl_3$)

2-((2*R*,6*S*)-6-((*S*)-2-hydroxy-2-phenylethyl)piperidin-2-yl)-1-phenylethan-1-one (39)

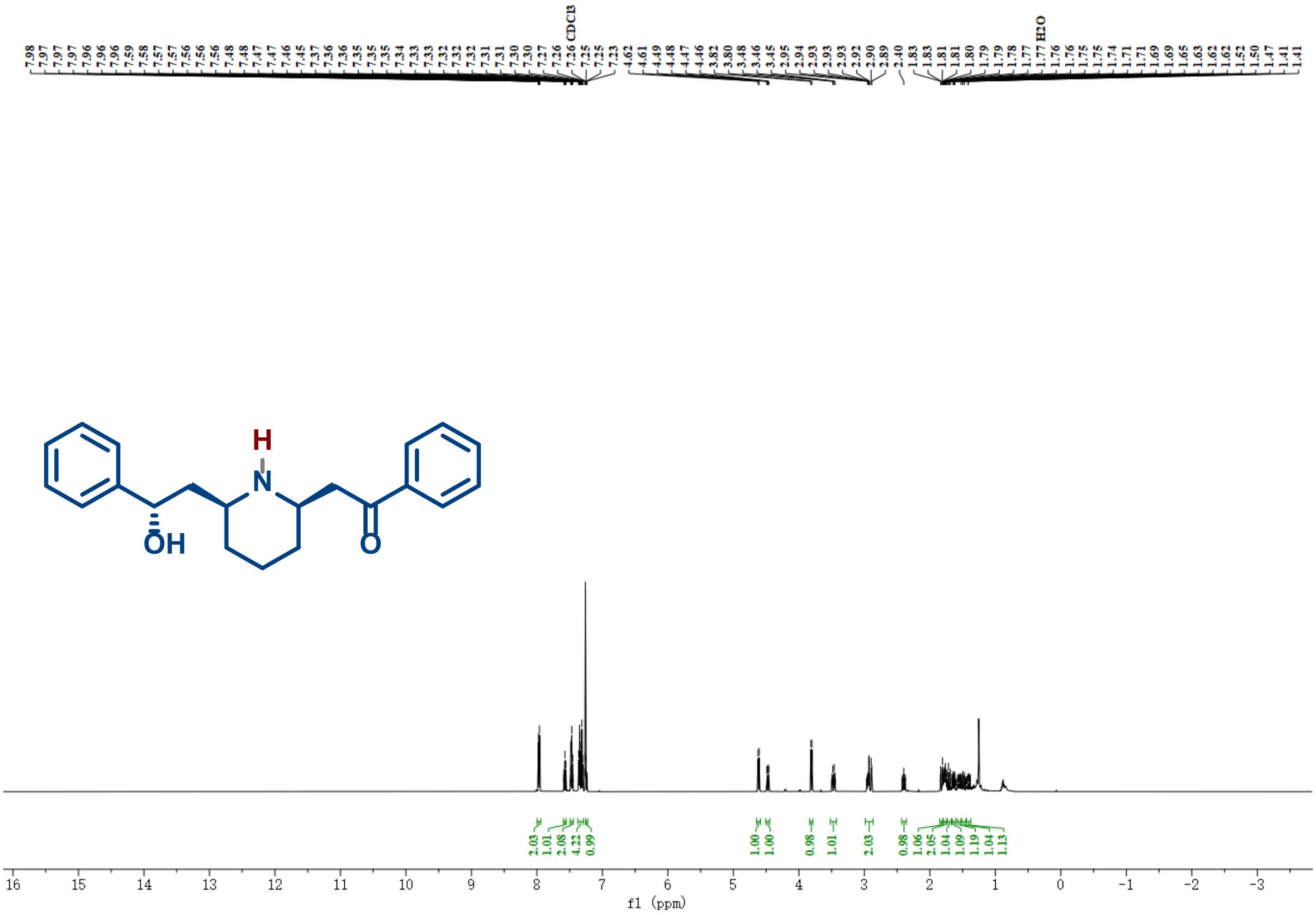

^{1}H NMR (500 MHz, $CDCl_3$)

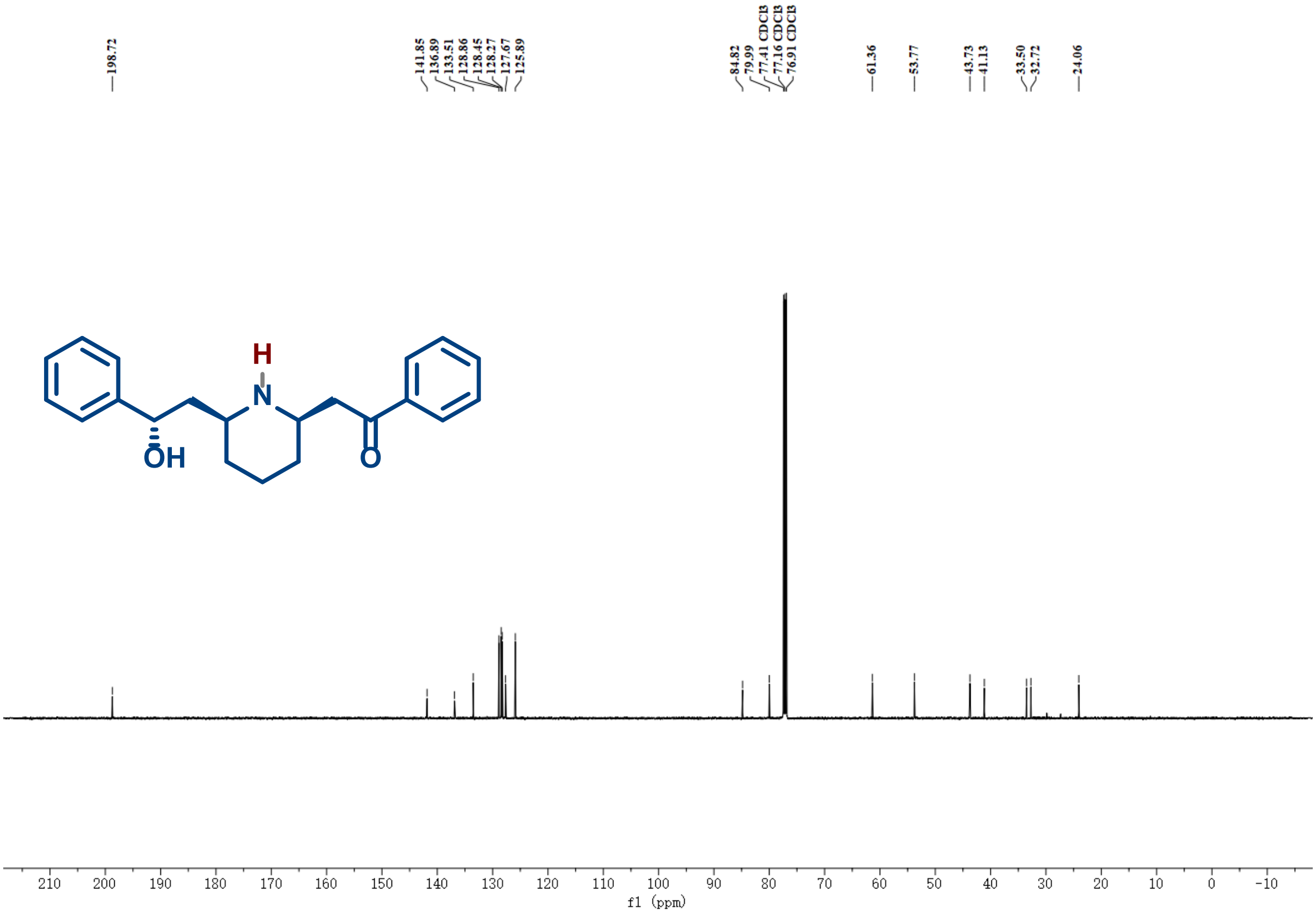

^{13}C NMR (126 MHz, $CDCl_3$)

***N*-benzyl-*N*-cyclohexylcyclohexanamine (45)**

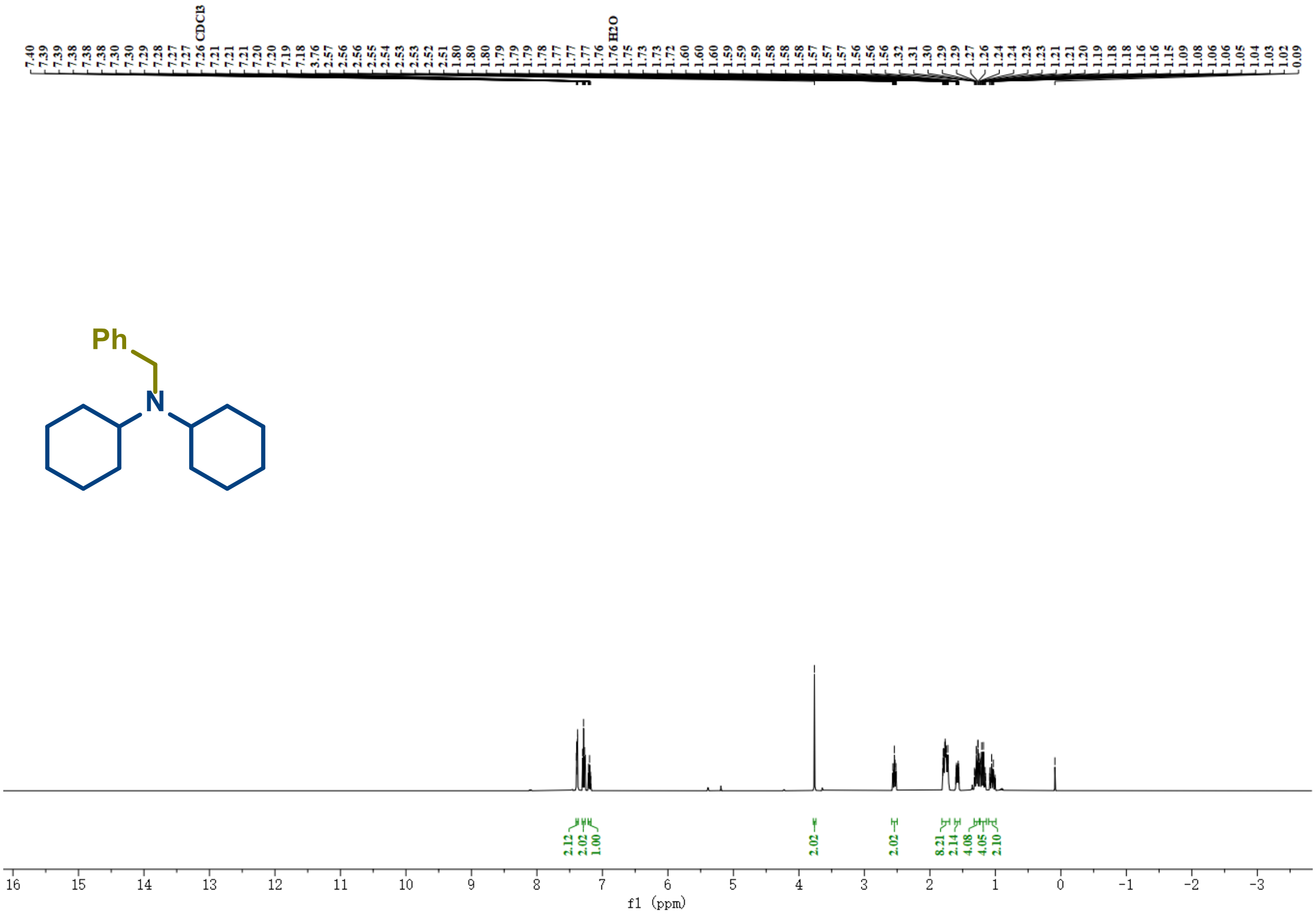

^{1}H NMR (500 MHz, CDCl$_3$)

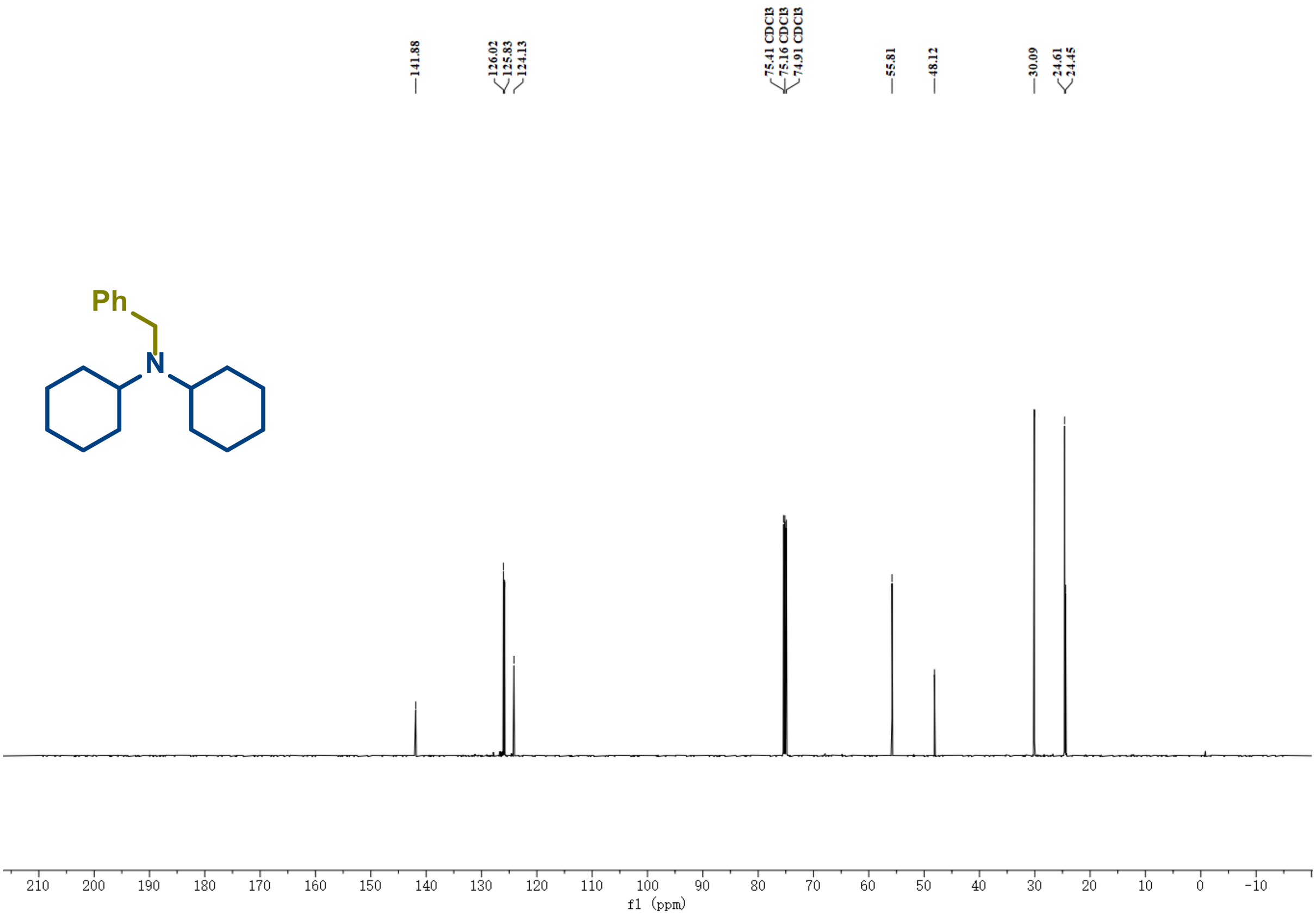

^{13}C NMR (126 MHz, CDCl$_3$)

***N*-allyl-*N*-cyclohexylcyclohexanamine (46)**

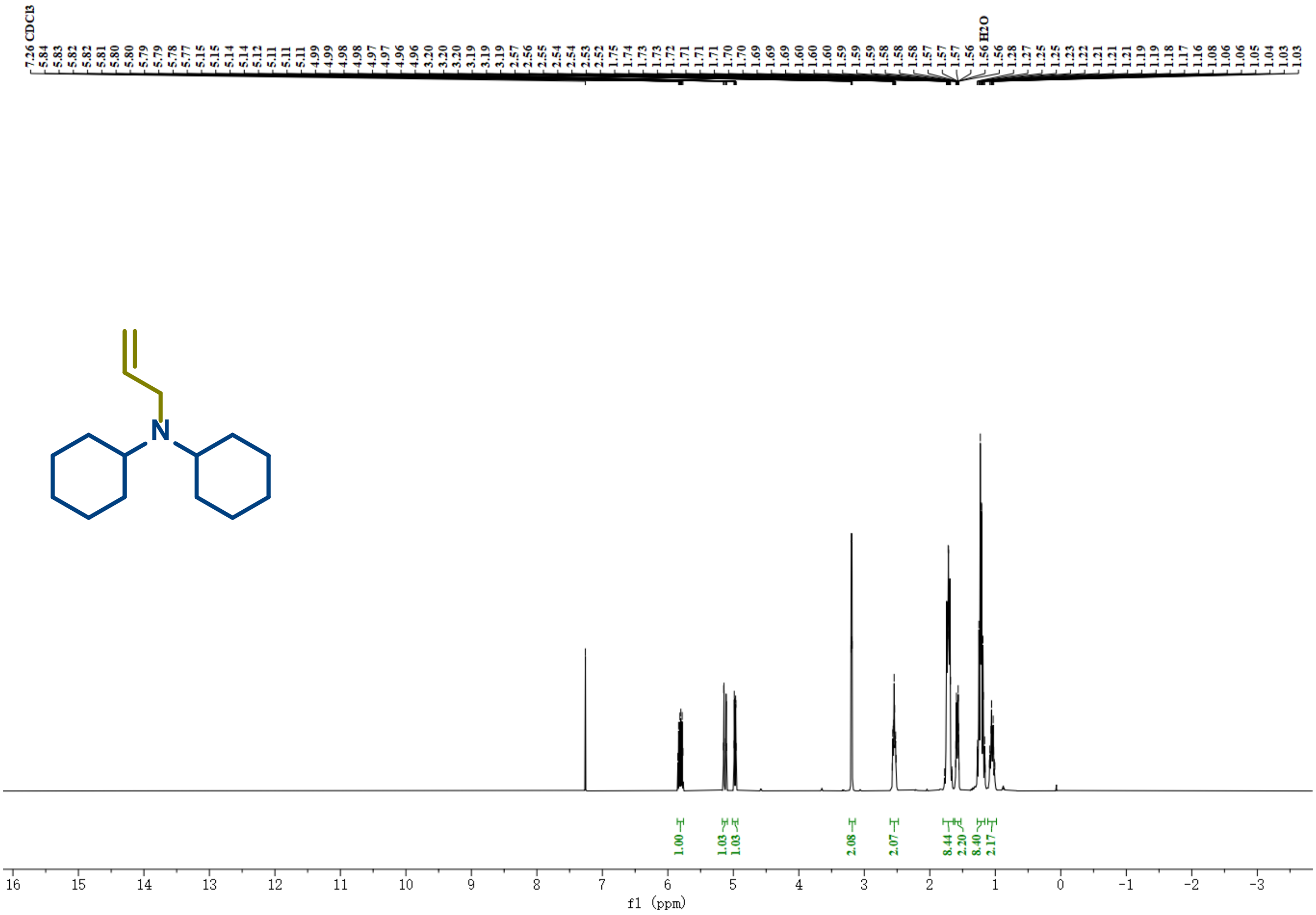

^{1}H NMR (500 MHz, CDCl$_3$)

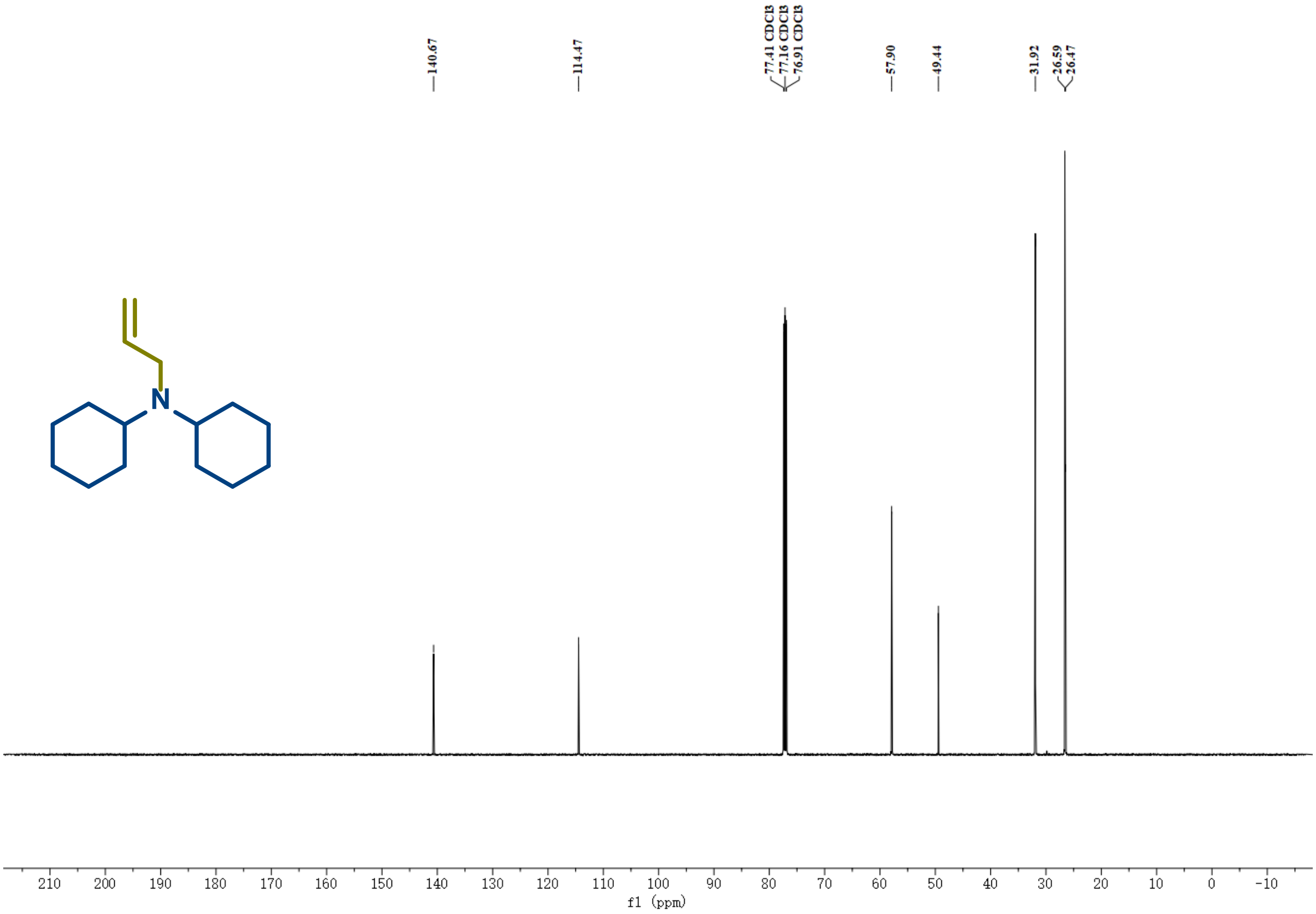

^{13}C NMR (126 MHz, CDCl$_3$)

N-cyclohexyl-*N*-(prop-2-yn-1-yl)cyclohexanamine (47)

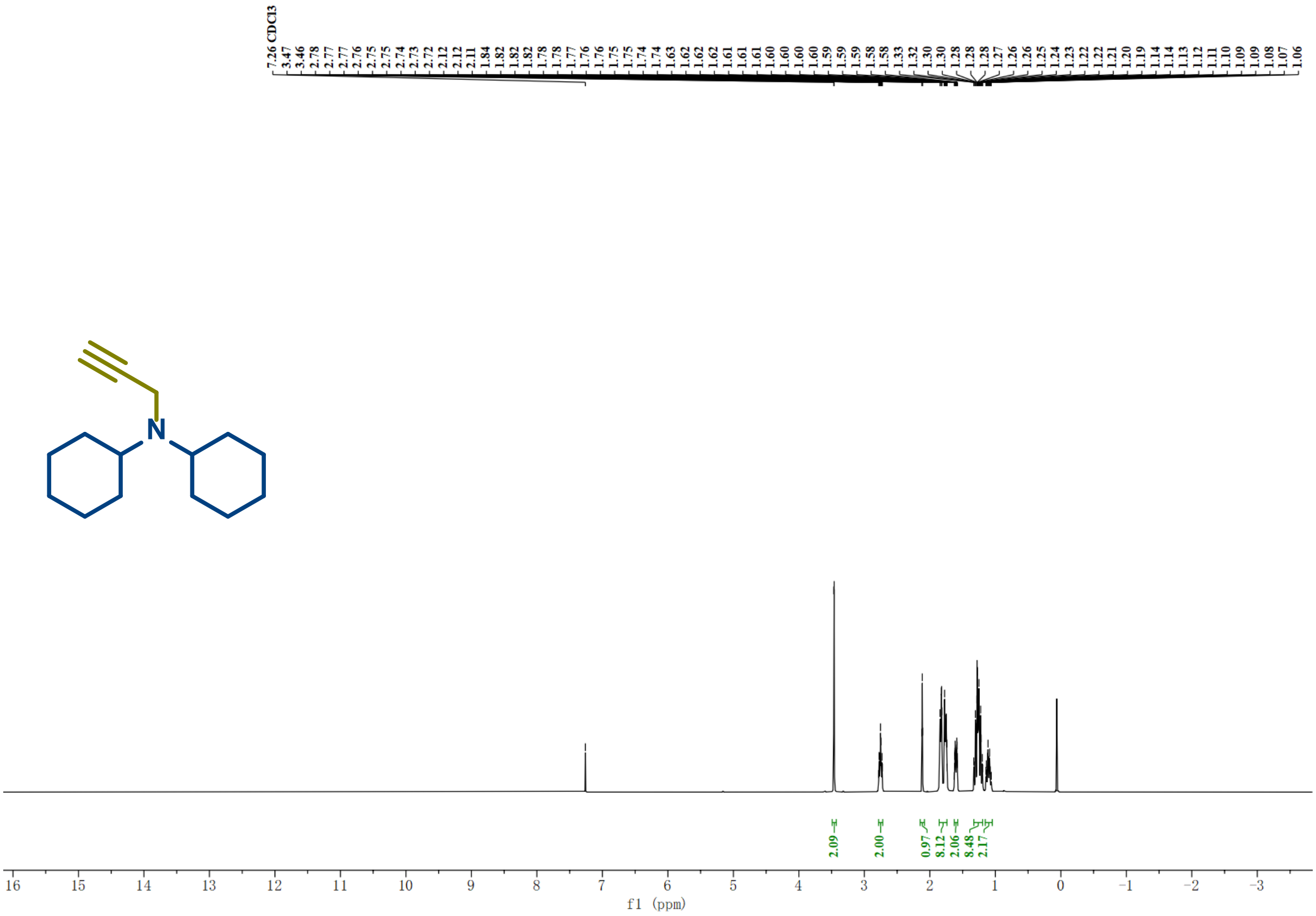

^{1}H NMR (500 MHz, $CDCl_3$)

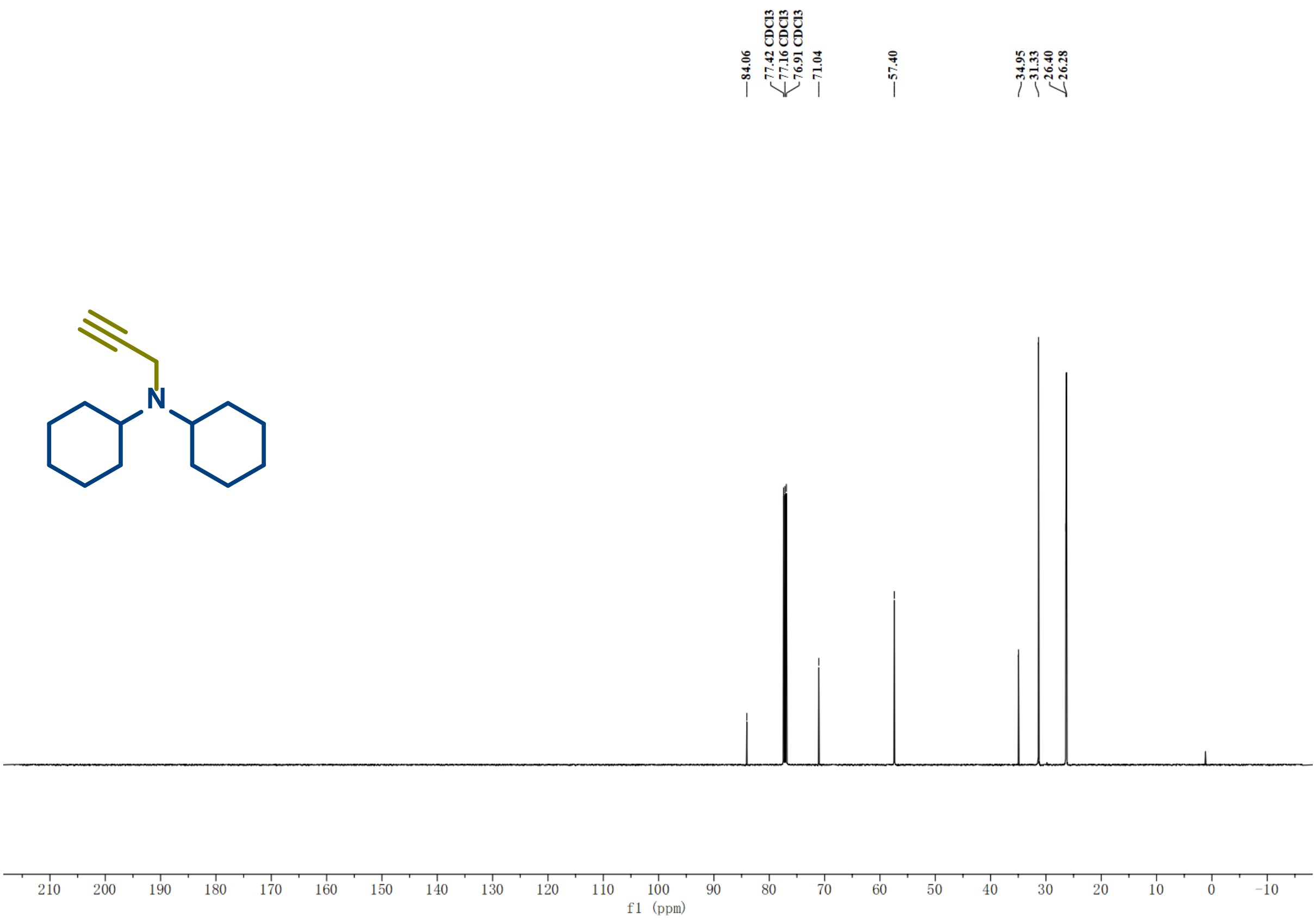

^{13}C NMR (126 MHz, $CDCl_3$)

N-(3,3-diphenylpropyl)-*N*-phenylaniline (48)

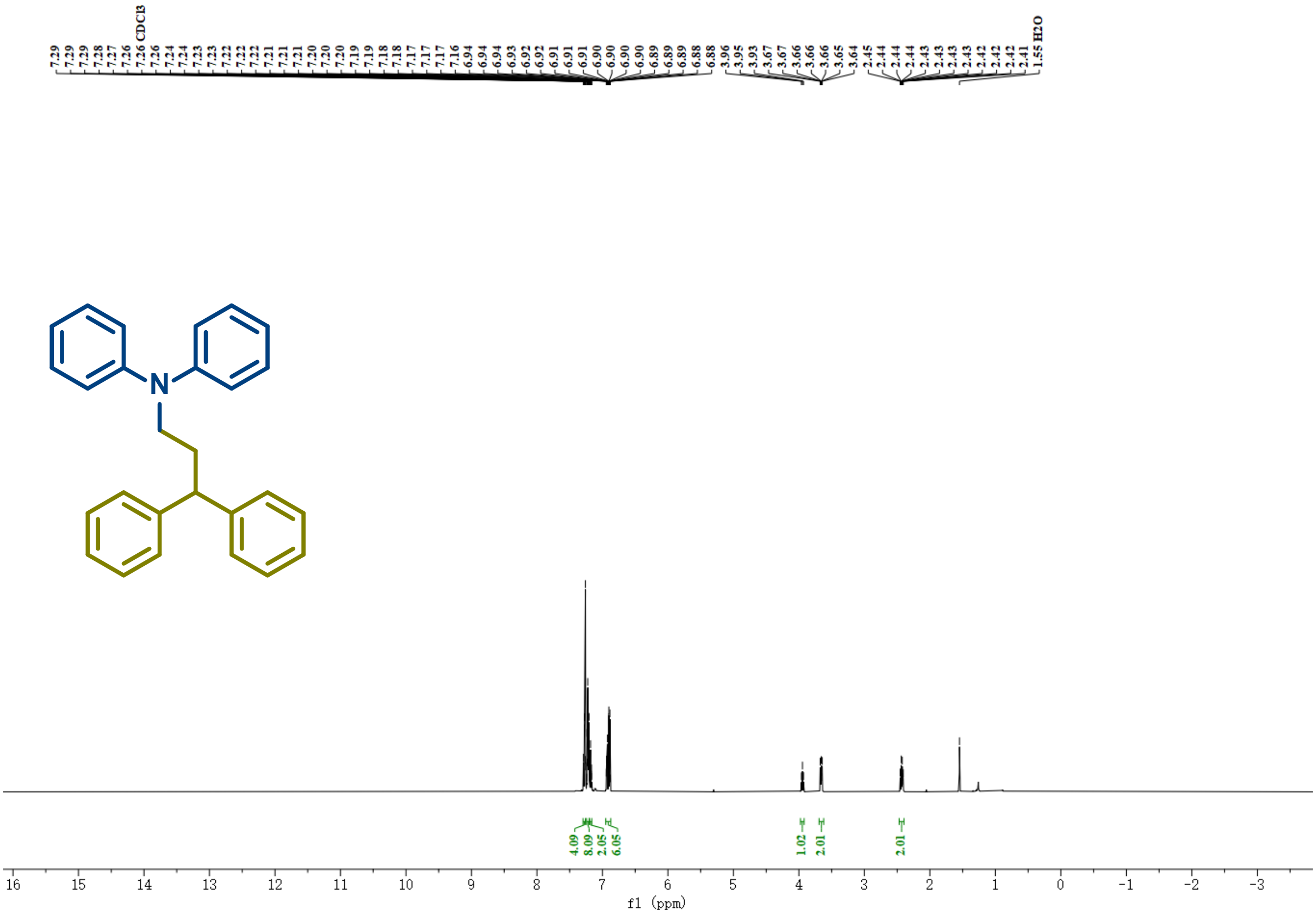

^{1}H NMR (500 MHz, $CDCl_3$)

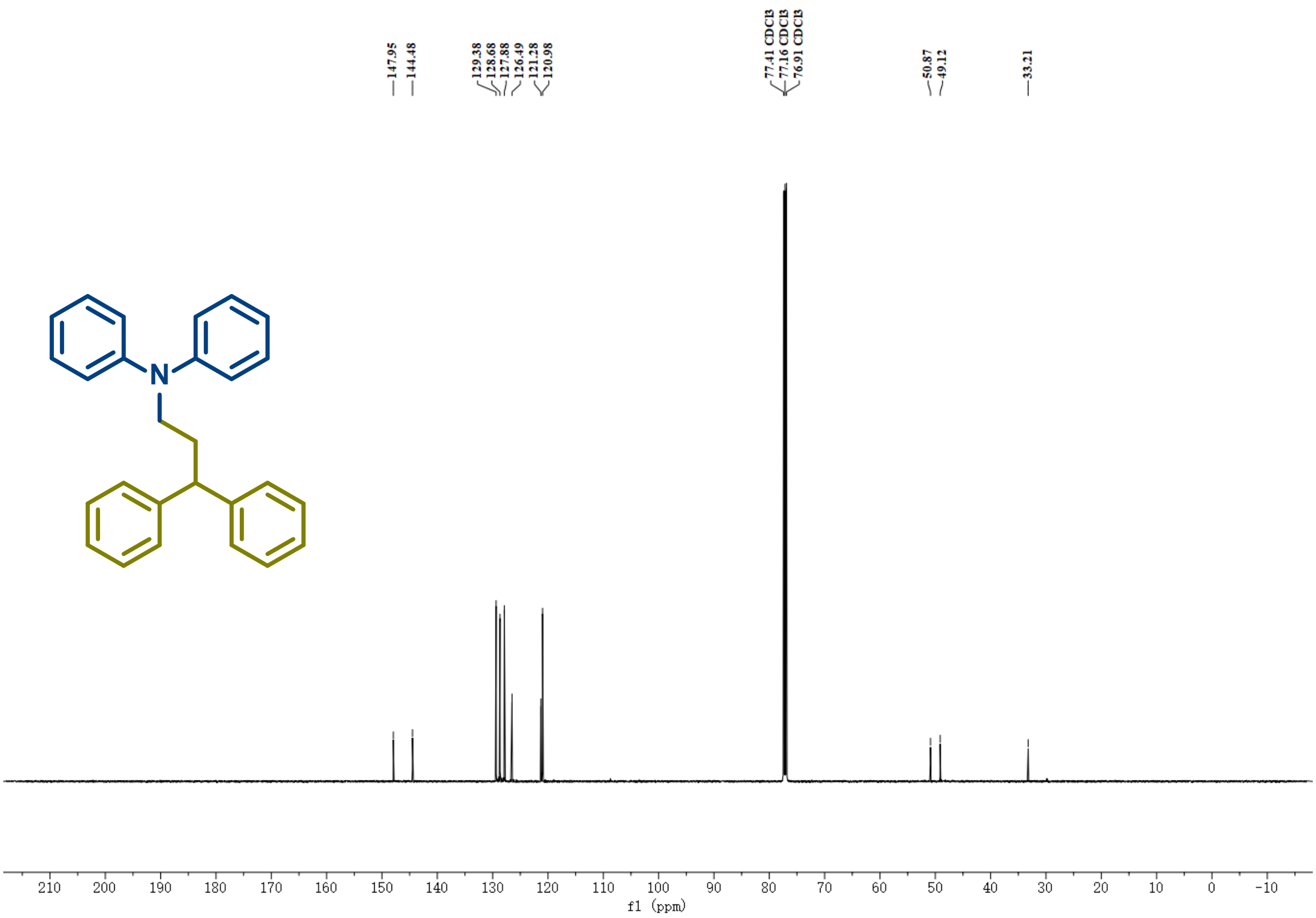

^{13}C NMR (126 MHz, $CDCl_3$)

7 Supplementary references

1 Alvarez-Montoya, A. *et al.* B(C6F5)3-Catalyzed Dehydrogenation of Pyrrolidines to Form Pyrroles. *ACS Catal.* **14**, 4856–4864 (2024).

2 Senaweera, S. M., Singh, A. & Weaver, J. D. Photocatalytic Hydrodefluorination: Facile Access to Partially Fluorinated Aromatics. *J. Am. Chem. Soc.* **136**, 3002–3005 (2014).

3 Lee, O.-Y., Law, K.-L., Ho, C.-Y. & Yang, D. Highly Chemoselective Reductive Amination of Carbonyl Compounds Promoted by InCl3/Et3SiH/MeOH System. *J. Org. Chem.* **73**, 8829–8837 (2008).

4 Odabachian, Y., Wang, Q. & Zhu, J. Tertiary Amines as Synthetic Equivalents of Vinyl Cations: Zinc Bromide Promoted Coupling of Propargylamines with α-Isocyanoacetamides To Give 2,4,5-Trisubstituted Oxazoles Initiated by an Internal Redox Process. *Chem. – Eur. J.* **19**, 12229–12233 (2013).

5 Margrey, K. A., Levens, A. & Nicewicz, D. A. Direct Aryl C−H Amination with Primary Amines Using Organic Photoredox Catalysis. *Angew. Chem.* **129**, 15850–15854 (2017).

6 Simonelli, L. *et al.* CLÆSS: The hard X-ray absorption beamline of the ALBA CELLS synchrotron. *Cogent Phys.* **3**, 1231987 (2016).

7 Monti, S., Li, C. & Carravetta, V. Reactive Dynamics Simulation of Monolayer and Multilayer Adsorption of Glycine on Cu(110). *J. Phys. Chem. C* **117**, 5221–5228 (2013).

8 Monti, S. *et al.* Exploring the conformational and reactive dynamics of biomolecules in solution using an extended version of the glycine reactive force field. *Phys. Chem. Chem. Phys.* **15**, 15062–15077 (2013).

9 Rahaman, O. *et al.* Development of a ReaxFF Reactive Force Field for Aqueous Chloride and Copper Chloride. *J. Phys. Chem. A* **114**, 3556–3568 (2010).

10 Thompson, A. P. *et al.* LAMMPS - a flexible simulation tool for particle-based materials modeling at the atomic, meso, and continuum scales. *Comput. Phys. Commun.* **271**, 108171 (2022).

11 Pradhan, S. *et al.* An Atomically Dispersed Photocatalyst for Undirected para-Selective C─H Bond Functionalizations. *Angew. Chem. Int. Ed.* e202508512 (2025) doi:10.1002/anie.202508512.

12 Sahoo, P. K. *et al.* An Atomically Dispersed Mn Photocatalyst for Vicinal Dichlorination of Nonactivated Alkenes. *J. Am. Chem. Soc.* **147**, 11829–11840 (2025).

13 Ghosh, T. *et al.* A robust Fe-based heterogeneous photocatalyst for the visible-light-mediated selective reduction of an impure CO2 stream. *Chem. Sci.* **15**, 11488–11499 (2024).

14 Giannozzi, P. *et al.* Advanced capabilities for materials modelling with Quantum ESPRESSO. *J. Phys. Condens. Matter* **29**, 465901 (2017).

15 Gaussian 16 Rev. C.01 (Wallingford, CT, 2016).

16 Marenich, A. V., Cramer, C. J. & Truhlar, D. G. Universal Solvation Model Based on Solute Electron Density and on a Continuum Model of the Solvent Defined by the Bulk

Dielectric Constant and Atomic Surface Tensions. *J. Phys. Chem. B* **113**, 6378–6396 (2009).

17 Xu, J. *et al.* Hf4+-exchanged montmorillonite-boosted Pd-catalyzed reductive aminolysis of aryl ethers to efficiently synthesize cyclohexylamines. *Green Chem.* **25**, 2318–2326 (2023).

18 Kawahara, R., Fujita, K. & Yamaguchi, R. Multialkylation of Aqueous Ammonia with Alcohols Catalyzed by Water-Soluble Cp*Ir−Ammine Complexes. *J. Am. Chem. Soc.* **132**, 15108–15111 (2010).

19 Moriyama, K., Nakamura, Y. & Togo, H. Oxidative Debenzylation of N-Benzyl Amides and O-Benzyl Ethers Using Alkali Metal Bromide. *Org. Lett.* **16**, 3812–3815 (2014).

20 Saper, N. I. & Snider, B. B. 2,2,6,6-Tetramethylpiperidine-Catalyzed, Ortho-selective Chlorination of Phenols by Sulfuryl Chloride. *J. Org. Chem.* **79**, 809–813 (2014).

21 Vasseur, A. *et al.* Secondary Phosphine Oxides as Multitalented Preligands En Route to the Chemoselective Palladium-Catalyzed Oxidation of Alcohols. *ChemCatChem* **9**, 728–732 (2017).

22 Haugland, M. M. *et al.* 2′-Alkynylnucleotides: A Sequence- and Spin Label-Flexible Strategy for EPR Spectroscopy in DNA. *J. Am. Chem. Soc.* **138**, 9069–9072 (2016).

23 Anderson, C. D., Shea, K. J. & Rychnovsky, S. D. Strategies for the Generation of Molecularly Imprinted Polymeric Nitroxide Catalysts. *Org. Lett.* **7**, 4879–4882 (2005).

24 Janoschka, T., Martin, N., Hager, M. D. & Schubert, U. S. An Aqueous Redox-Flow Battery with High Capacity and Power: The TEMPTMA/MV System. *Angew. Chem. Int. Ed.* **55**, 14427–14430 (2016).

25 Zeng, H., Cao, D., Qiu, Z. & Li, C.-J. Palladium-Catalyzed Formal Cross-Coupling of Diaryl Ethers with Amines: Slicing the 4-O-5 Linkage in Lignin Models. *Angew. Chem.* **130**, 3814–3819 (2018).

26 Li, G., Kanda, Y., Hong, S. Y. & Radosevich, A. T. Enabling Reductive C–N Cross-Coupling of Nitroalkanes and Boronic Acids by Steric Design of P(III)/P(V)═O Catalysts. *J. Am. Chem. Soc.* **144**, 8242–8248 (2022).

27 Escobar, R. A. & Johannes, J. W. A Unified and Practical Method for Carbon–Heteroatom Cross-Coupling using Nickel/Photo Dual Catalysis. *Chem. – Eur. J.* **26**, 5168–5173 (2020).

28 Senzaki, T., Saito, Y. & Kobayashi, S. Reductive N-Alkylation of Amines with Ketones Using Heterogeneous Polysilane–Palladium Catalysts under Continuous-Flow Conditions. *Org. Lett.* **26**, 3772–3777 (2024).

29 Chen, Z., Zeng, H., Gong, H., Wang, H. & Li, C.-J. Palladium-catalyzed reductive coupling of phenols with anilines and amines: efficient conversion of phenolic lignin model monomers and analogues to cyclohexylamines. *Chem. Sci.* **6**, 4174–4178 (2015).

30 Mao, R., Frey, A., Balon, J. & Hu, X. Decarboxylative C(sp3)–N cross-coupling via synergetic photoredox and copper catalysis. *Nat. Catal.* **1**, 120–126 (2018).

31 Wu, Z. *et al.* Sulfonato–Cu(salen) Complex Catalyzed N-Arylation of Aliphatic Amines with Aryl Halides in Water. *Eur. J. Org. Chem.* **2010**, 4971–4975 (2010).

32 Apodaca, R. & Xiao, W. Direct Reductive Amination of Aldehydes and Ketones Using Phenylsilane: Catalysis by Dibutyltin Dichloride. *Org. Lett.* **3**, 1745–1748 (2001).

33 Forberg, D., Schwob, T. & Kempe, R. Catalytic condensation for the formation of polycyclic heteroaromatic compounds. *Nat. Commun.* **9**, 1751 (2018).

34 Tian, Z.-Y., Ming, X.-X., Teng, H.-B., Hu, Y.-T. & Zhang, C.-P. Transition-Metal-Free N-Arylation of Amines by Triarylsulfonium Triflates. *Chem. – Eur. J.* **24**, 13744–13748 (2018).

35 Liu, C., Zhang, Y. & Shang, R. BINOLates as potent reducing photocatalysts for inert-bond activation and reduction of unsaturated systems. *Chem* **11**, (2025).

36 Tian, M., Li, J., Mou, Q. & Liu, M. Selective Oxyfunctionalization of Benzylic C–H with No Solvent. *J. Org. Chem.* **89**, 16645–16652 (2024).

37 Brown, T. J., Cumbes, M., Diorazio, L. J., Clarkson, G. J. & Wills, M. Use of (Cyclopentadienone)iron Tricarbonyl Complexes for C–N Bond Formation Reactions between Amines and Alcohols. *J. Org. Chem.* **82**, 10489–10503 (2017).

38 Yao, W., Wang, J., Zhong, A., Li, J. & Yang, J. Combined KOH/BEt3 Catalyst for Selective Deaminative Hydroboration of Aromatic Carboxamides for Construction of Luminophores. *Org. Lett.* **22**, 8086–8090 (2020).

39 Muñoz, A., Leo, P., Orcajo, G., Martínez, F. & Calleja, G. URJC-1-MOF as New Heterogeneous Recyclable Catalyst for C-Heteroatom Coupling Reactions. *ChemCatChem* **11**, 3376–3380 (2019).

40 Topchiy, M. A. *et al.* Solvent-Free Buchwald–Hartwig (Hetero)arylation of Anilines, Diarylamines, and Dialkylamines Mediated by Expanded-Ring N-Heterocyclic Carbene Palladium Complexes. *Eur. J. Org. Chem.* **2016**, 1908–1914 (2016).

41 Yan, Q., Gin, E., Banwell, M. G., Willis, A. C. & Carr, P. D. A Unified Approach to the Isomeric α-, β-, γ-, and δ-Carbolines via their 6,7,8,9-Tetrahydro Counterparts. *J. Org. Chem.* **82**, 4328–4335 (2017).
