# Supplementary material for: An Atomically Dispersed Copper-Catalyst Lights the Way to Selective N-Demethylations
Source: Nat Commun. 2026 Aug 19;17:8489. doi: 10.1038/s41467-026-76282-0 (PMC13490395; doi:10.1038/s41467-026-76282-0)
Supplement: Supplementary file 2 [file 41467_2026_76282_MOESM2_ESM.pdf]

nature portfolio

Peer Review File

An Atomically Dispersed Copper-Catalyst Lights the Way to Selective N-Demethylations

Corresponding Author: Professor Shoubhik Das

This file contains all reviewer reports in order by version, followed by all author rebuttals in order by version.

Version 0:

Reviewer comments:

Reviewer #1

(Remarks to the Author)
This article presents a method for N-demethylation (N-dealkylation) developed using photocatalytic techniques, along with substrate applications and in-depth mechanistic studies, utilizing an atomically dispersed Cu catalyst supported on graphitic carbon nitride (Cu@g-C3N4). Selective N-dealkylation reactions remain of great importance in the fields of medicinal chemistry, metabolite synthesis, and late-stage functionalization. Overall, the article is well-prepared, the results are presented in detail, and it is scientifically robust. In particular, the synthesis and characterization of the catalyst, along with the mechanism studies, are very comprehensive and constitute one of the strongest aspects of the work. The authors have employed a wide range of techniques, including HAADF-STEM, XANES/EXAFS, ssNMR, EPR, radical trapping studies, isotope labeling experiments, kinetic studies, and DFT calculations, and have produced significant results. Furthermore, the application of the method to pharmaceutically important substrates enhances the practical value of the study.

On the other hand, the study does not introduce any significant innovations in terms of mechanism or concept, apart from the catalyst developed. For example, photocatalytic oxidative N-dealkylation under aerobic conditions is a well-established area of research in the literature, and mechanisms involving aminium radical cations, iminium intermediates, reactive oxygen species (ROS), and hydrolysis steps have been extensively discussed in previous studies.

Another issue concerns the fact that the nature of the conceptual innovation has not been discussed in relation to the photoredox-catalyzed N-dealkylation studies found in the literature (https://doi.org/10.1021/acs.joc.3c00656). In particular, previous studies have demonstrated the aerobic photoredox-assisted N-dealkylation of tertiary amines with a broad substrate scope, including aliphatic amines, aniline-type substrates, complex pharmaceutical compounds, advanced functionalization applications, and mechanism studies involving radical and iminium intermediates. These previous studies have also shown that the transformation is not limited to N-demethylation but can extend to more general N-dealkylation processes. In this context, the present article appears to primarily present a new heterogeneous/single-atom catalyst platform for an already known photoredox reaction, rather than introducing a new reactivity or filling a gap. While the catalyst characterization and mechanism analysis are comprehensive and valuable, the paper currently does not sufficiently clarify how the true innovation extends beyond the replacement of a homogeneous Ir photocatalyst with a heterogeneous Cu SAC system.

In summary, given the comparative literature review presented in the Supporting Information section, this study still appears to focus primarily on the development of a new heterogeneous/single-atom catalyst for an already known photoredox reaction. Although the catalyst design, characterization, and mechanism studies are valuable and scientifically rigorous, general conceptual progress beyond existing photoredox N-dealkylation methods currently appears limited.

Therefore, the study may be more suitable for consideration in a journal more specifically focused on catalysis or photocatalysis.

Some suggestions

1) Beyond the development of the heterogeneous Cu SAC photocatalyst, the authors should more clearly define the study's advantages over methods described in the literature.
2) The article should include a more comprehensive discussion and comparison with previous photoredox-catalyzed N-

dealkylation studies, particularly regarding substrate scope, mechanism, and synthetic feasibility. The differences between the developed Cu SAC system and previously reported homogeneous photoredox systems should be clearly stated. (https://doi.org/10.1021/acs.joc.3c00656)
3) In summary, the article should better explain how the method presented overcomes the limitations of previous photocatalytic N-dealkylation methods, beyond merely describing catalyst development.
4) Given the detailed catalyst design and characterization presented in the study, the reported quantum yield (AQY = 0.7%) appears relatively low. Furthermore, the method requires an oxygen atmosphere and reaction times similar to those reported in the literature. While these results do not diminish the overall value of the study, the authors should discuss more clearly what these observations imply regarding the overall efficiency and practical advantages of the developed catalytic system. At present, the method's most notable practical advantage appears to be the heterogeneous catalyst's ability to be recycled over multiple reaction cycles.
5) Recyclability tests offer significant advantages. However, determining what forms the recycled catalyst takes after losing its activity following numerous catalytic cycles (If it loses after a certain number of cycles) will further support the catalyst's stability.
6) Rather than simply presenting a comparative summary table in the Supporting Information section, it would be more beneficial to include a more detailed discussion of closely related recent studies on photoredox N-dealkylation within the main article. In particular, a clearer comparison of substrate scope, mechanistic similarities, and actual differences among the reported systems would significantly strengthen the discussion section.

Reviewer #2

(Remarks to the Author)
A "hard-to-carry-on" reaction, N-demethylation, is made easier by a system envisaging atomically dispersed non-noble Cu species on a carbon nitride support, under visible light and in aerobic environment.
The work is sound, and the conclusions look well supported by the data. In particular, the authors were not only able to monitor the reaction progress, but also to trap some of the transient species, which gives remarkable insight on the actual photocatalytic mechanism.
I will focus on the computational part mostly, based on my expertise.
The authors follow an accurate procedure: after relaxing reactants and products at the GGA level, refinement calculations are done with hybrid functionals, as well as TD-DFT runs to evaluate the excitations related to the photocatalytic process. The solvent is described implicitely, unless a case where some molecules are explicitely included. It should be mentioned, though, at what level were the PDOS and orbital plots in Fig. 5 obtained.
The structural model is, in general, adequate. I wonder, though, if a reactive species such as Cu1 is bound strongly enough to the matrix. Did the authors check its stability at room temperature through AIMD or similar?
A peculiar detail emerges in Fig. 5: the HOMO (or "VB" is mostly located on a phenyl substituent, whose presence is, in fact, a computational artifact to saturate the model, if I understand correctly. The real material does not envisage the presence of that type of phenyl substituent. Would the general photocatalytic mechanism that the authors depict hold true, even if the HOMO level was shifted down to the first available C_3N_4 state?
In general, what is the reference adopted for the energy scale in Fig. 5? Is it consistent with the level alignment of the real system?
I find interesting that the Cu1 species is not involved in the photogeneration of charge carriers, and its oxidation state remains quite constant through the catalytic cycle (I am aware of similar evidences on comparable models, it is quite expected). One may thus wonder what is precisely its role... would bare C_3N_4 be equally active, based on the computational evidence? Can the authors perhaps elaborate more on this? A further element to question on the actual photocatalytic role of Cu species is given by this very recent work: https://doi.org/10.1002/ange.2299555

Version 1:

Reviewer comments:

Reviewer #2

(Remarks to the Author)
I think that the authors replied thoroughly to all comments. I recommend to accept the paper.

Open Access This Peer Review File is licensed under a Creative Commons Attribution 4.0 International License, which permits use, sharing, adaptation, distribution and reproduction in any medium or format, as long as you give appropriate credit to the original author(s) and the source, provide a link to the Creative Commons license, and indicate if changes were made.
In cases where reviewers are anonymous, credit should be given to 'Anonymous Referee' and the source.
The images or other third party material in this Peer Review File are included in the article's Creative Commons license, unless indicated otherwise in a credit line to the material. If material is not included in the article's Creative Commons license and your intended use is not permitted by statutory regulation or exceeds the permitted use, you will need to obtain permission directly from the copyright holder.
To view a copy of this license, visit https://creativecommons.org/licenses/by/4.0/

Response Letter for Reviewer Comments

We thank referees for their valuable comments. We have carefully addressed all of their comments in this response letter. Our responses are shown in blue, while the revised text quoted from the manuscript is shown in orange. In addition, all changes have been highlighted in yellow in the revised manuscript and SI file.

REVIEWER COMMENTS

Reviewer #1 (Remarks to the Author):

This article presents a method for *N*-demethylation (*N*-dealkylation) developed using photocatalytic techniques, along with substrate applications and in-depth mechanistic studies, utilizing an atomically dispersed Cu catalyst supported on graphitic carbon nitride (Cu@g-C_3N_4). Selective *N*-dealkylation reactions remain of great importance in the fields of medicinal chemistry, metabolite synthesis, and late-stage functionalization. Overall, the article is well-prepared, the results are presented in detail, and it is scientifically robust. In particular, the synthesis and characterization of the catalyst, along with the mechanism studies, are very comprehensive and constitute one of the strongest aspects of the work. The authors have employed a wide range of techniques, including HAADF-STEM, XANES/EXAFS, ssNMR, EPR, radical trapping studies, isotope labeling experiments, kinetic studies, and DFT calculations, and have produced significant results. Furthermore, the application of the method to pharmaceutically important substrates enhances the practical value of the study.

On the other hand, the study does not introduce any significant innovations in terms of mechanism or concept, apart from the catalyst developed. For example, photocatalytic oxidative *N*-dealkylation under aerobic conditions is a well-established area of research in the literature, and mechanisms involving aminium radical cations, iminium intermediates, reactive oxygen species (ROS), and hydrolysis steps have been extensively discussed in previous studies.

Another issue concerns the fact that the nature of the conceptual innovation has not been discussed in relation to the photoredox-catalyzed *N*-dealkylation studies found in the literature (https://doi.org/10.1021/acs.joc.3c00656). In particular, previous studies have demonstrated the aerobic photoredox-assisted *N*-dealkylation of tertiary amines with a broad substrate scope, including aliphatic amines, aniline-type substrates, complex pharmaceutical compounds, advanced functionalization applications, and mechanism studies involving radical and iminium intermediates. These previous studies have also shown that the transformation is not limited to *N*-demethylation but can extend to more general *N*-dealkylation processes. In this context, the present article appears to primarily present a new heterogeneous/single-atom catalyst platform for an already known photoredox reaction, rather than introducing a new reactivity or filling a gap. While the catalyst characterization and mechanism analysis are comprehensive and valuable, the paper currently does

not sufficiently clarify how the true innovation extends beyond the replacement of a homogeneous Ir photocatalyst with a heterogeneous Cu SAC system.

In summary, given the comparative literature review presented in the Supporting Information section, this study still appears to focus primarily on the development of a new heterogeneous/single-atom catalyst for an already known photoredox reaction. Although the catalyst design, characterization, and mechanism studies are valuable and scientifically rigorous, general conceptual progress beyond existing photoredox *N*-dealkylation methods currently appears limited.

Therefore, the study may be more suitable for consideration in a journal more specifically focused on catalysis or photocatalysis.

Response: We sincerely thank the reviewer for the careful evaluation of our manuscript and for the positive comments on the scientific robustness of the work, the comprehensive catalyst characterization, the mechanistic studies, and the application to pharmaceutically relevant substrates. We also appreciate the reviewer's critical assessment regarding the conceptual innovation of the study.

We agree that aerobic photoredox *N*-dealkylation of tertiary amines has been previously established, and that mechanisms involving aminium radical cations, iminium intermediates, reactive oxygen species, and hydrolysis have been discussed in earlier studies. In particular, we recognize the importance of the reported homogeneous Ir-photoredox *N*-dealkylation system. In the revised manuscript, we have therefore refined the positioning of the study. We now clarify that the central contribution of this work is not the discovery of an entirely new *N*-dealkylation mechanism. Rather, the key advance is the translation of aerobic photoredox *N*-dealkylation into a recyclable, non-noble-metal, heterogeneous single-atom photocatalytic platform. We believe this translation is nontrivial and goes beyond a simple catalyst substitution. Homogeneous noble-metal photocatalysts operate in a molecular solution-phase environment and typically benefit from highly tunable excited-state redox properties. In contrast, a heterogeneous Cu-based photocatalytic system requires a different set of reaction parameters, including solid–liquid interfacial charge transfer, substrate interaction with the catalyst surface, oxygen activation, solvent-assisted hydrogen/proton transfer, iminium stabilization, hydrolysis, and catalyst recovery. We have also revised the manuscript to better explain the mechanistic significance of the Cu single-atom platform. While the overall radical/iminium/hydrolysis sequence is related to known aerobic photoredox *N*-dealkylation pathways, the present work provides a structurally defined heterogeneous system in which the roles of isolated Cu sites and the functionalized carbon nitride matrix can be experimentally and computationally interrogated.

We hope that these revisions now make the conceptual contribution of the work clearer. The present study should not be viewed merely as replacing a homogeneous Ir photocatalyst with a heterogeneous Cu material, but rather as

establishing a recyclable, earth-abundant, single-atom heterogeneous photocatalytic manifold for sustainable *N*-demethylation/*N*-dealkylation and late-stage amine editing.

Some suggestions

1) Beyond the development of the heterogeneous Cu SAC photocatalyst, the authors should more clearly define the study's advantages over methods described in the literature.

Response: We thank the reviewer for this constructive suggestion. We agree that the original manuscript did not sufficiently distinguish the advantages of our system from previously reported homogeneous photoredox *N*-dealkylation methods. In the revised manuscript, we have made this distinction a central theme rather than a minor comparison.

The advantages of the present system are manifold. First, the catalyst is based on earth-abundant Cu rather than a precious-metal Ir photocatalyst. Second, Cu@f-gC_3N_4 is heterogeneous and can be recovered and reused for more than ten cycles without significant loss of activity. Third, the reaction proceeds with O_2 as the terminal oxidant and does not require nitromethane as a sacrificial nucleophilic trapping reagent. Fourth, the demethylated secondary amine products can be directly used in downstream *N*-alkylation reactions without purification, demonstrating the synthetic value of the reaction output. Fifth, the atomically dispersed Cu sites provide a structurally defined platform that allows us to correlate Cu coordination, charge separation, O_2 activation, radical formation, and iminium hydrolysis using spectroscopy, kinetic experiments, and DFT calculations.

➢ Abstract

"Although aerobic photoredox *N*-dealkylation of tertiary amines has been previously established, most reported systems rely on homogeneous photocatalysts or stoichiometric reagents, which limits catalyst recovery, reuse, and direct structure–activity analysis. Herein, we present a noble-metal-free, recyclable, and structurally defined heterogeneous photocatalytic platform for selective *N*-demethylation based on atomically dispersed Cu sites supported on functionalized graphitic carbon nitride (Cu@f-gC_3N_4). ... The reaction can be performed on a 10 mmol scale and the catalyst can be reused for more than ten cycles without significant loss of activity. … This work establishes atomically dispersed Cu@f-gC_3N_4 as a recyclable and mechanistically interrogable heterogeneous single-atom photocatalyst for this important transformation."

➢ Introduction

"Graphitic carbon nitride is particularly appealing as a photocatalytic support because of its visible-light absorption, chemical robustness, tunable band structure, and ability to coordinate isolated metal sites through nitrogen-rich motifs. Incorporating earth-abundant transition-metal single sites into functionalized carbon nitride may therefore provide a sustainable strategy for

developing recyclable photoredox catalysts that can mediate organic transformations while enabling detailed mechanistic interrogation.[23]"

- Conclusion

"Under 390 nm irradiation, Cu@f-gC_3N_4 enables the conversion of diverse aliphatic, aromatic, and pharmaceutically relevant tertiary amines into secondary amines under mild conditions. And the reaction can be performed on a 10 mmol scale, demonstrating the operational robustness of the system. … The significance of this work extends beyond replacing a homogeneous photocatalyst with a heterogeneous material. In contrast to noble-metal photoredox systems, the present platform uses earth-abundant Cu, allows catalyst recovery and reuse, and operates with molecular oxygen as the terminal oxidant. Moreover, the heterogeneous Cu@f-gC_3N_4 environment enables productive *N*-demethylation without sacrificial nucleophilic trapping additives such as nitromethane. This feature is synthetically important because the resulting secondary amine products can be directly engaged in downstream functionalization, as demonstrated by one-pot benzylation, allylation, and propargylation reactions. Thus, the process delivers not only demethylated products but also a practical entry to late-stage amine diversification. Mechanistic studies further show that the atomically dispersed Cu sites and the functionalized carbon nitride matrix jointly contribute to the reaction by facilitating charge separation, O_2 activation, radical formation, iminium generation, and hydrolysis. HAADF-STEM, ssNMR, XANES/EXAFS, XPS, and EPR confirm the isolated Cu environment, while radical trapping, ROS detection, isotope labeling, kinetic analysis, in situ FTIR, and DFT calculations support the proposed radical/iminium pathway. Therefore, this study establishes Cu@f-gC_3N_4 as a structurally defined and mechanistically interrogable heterogeneous single-atom photocatalyst for sustainable *N*-dealkylation chemistry, providing a complementary platform to existing homogeneous photoredox methods."

2) The article should include a more comprehensive discussion and comparison with previous photoredox-catalyzed *N*-dealkylation studies, particularly regarding substrate scope, mechanism, and synthetic feasibility. The differences between the developed Cu SAC system and previously reported homogeneous photoredox systems should be clearly stated. (https://doi.org/10.1021/acs.joc.3c00656)

Response: We agree with the reviewer. In the revised manuscript, we have added a dedicated discussion comparing our Cu@f-gC_3N_4 system with representative homogeneous photoredox *N*-dealkylation methods, especially the Ir-based aerobic system reported by Yilmaz and Emmert.

- Introduction

"Yilmaz and Emmert described a mild aerobic photoredox *N*-dealkylation of tertiary amines using an Ir-based photocatalyst and equimolar nitromethane as a nucleophilic trapping reagent, demonstrating broad substrate tolerance

across aliphatic amines, aniline derivatives, and pharmaceutically relevant molecules.[15] These studies have established that oxidative *N*-dealkylation can proceed through pathways involving aminium radical cations, iminium intermediates, reactive oxygen species, and hydrolysis. However, most homogeneous photoredox systems for *N*-demethylation still face limitations from the perspective of catalyst sustainability and process design. Precious-metal photocatalysts, particularly Ir- or Ru-based complexes, are highly effective but can be expensive, difficult to recover, and less attractive for applications where catalyst recycling, product purification, and metal contamination are important considerations.[16] In addition, homogeneous systems provide limited opportunities to correlate a structurally defined catalytic site with charge separation, oxygen activation, radical generation, and substrate oxidation.[17] Thus, while the elementary reaction manifold of aerobic photoredox *N*-dealkylation is known, translating this chemistry into a recyclable, noble-metal-free, and mechanistically well-defined heterogeneous photocatalytic platform remains an important challenge."

- Conclusion

"The significance of this work extends beyond replacing a homogeneous photocatalyst with a heterogeneous material. In contrast to noble-metal photoredox systems, the present platform uses earth-abundant Cu, allows catalyst recovery and reuse, and operates with molecular oxygen as the terminal oxidant. Moreover, the heterogeneous Cu@f-gC_3N_4 environment enables productive *N*-demethylation without sacrificial nucleophilic trapping additives such as nitromethane. This feature is synthetically important because the resulting secondary amine products can be directly engaged in downstream functionalization, as demonstrated by one-pot benzylation, allylation, and propargylation reactions. Thus, the process delivers not only demethylated products but also a practical entry to late-stage amine diversification. Mechanistic studies further show that the atomically dispersed Cu sites and the functionalized carbon nitride matrix jointly contribute to the reaction by facilitating charge separation, O_2 activation, radical formation, iminium generation, and hydrolysis. HAADF-STEM, ssNMR, XANES/EXAFS, XPS, and EPR confirm the isolated Cu environment, while radical trapping, ROS detection, isotope labeling, kinetic analysis, in situ FTIR, and DFT calculations support the proposed radical/iminium pathway. Therefore, this study establishes Cu@f-gC_3N_4 as a structurally defined and mechanistically interrogable heterogeneous single-atom photocatalyst for sustainable *N*-dealkylation chemistry, providing a complementary platform to existing homogeneous photoredox methods."

3) In summary, the article should better explain how the method presented overcomes the limitations of previous photocatalytic *N*-dealkylation methods, beyond merely describing catalyst development.

Response: We thank the reviewer for this important point. We have revised the manuscript to clarify that the present method overcomes several practical

limitations of previous homogeneous photoredox *N*-dealkylation methods. In particular, our system avoids precious-metal photocatalysts, enables catalyst recovery and reuse, proceeds without nitromethane as a sacrificial nucleophilic trapping reagent, and allows direct downstream functionalization of the demethylated secondary amine products. We also emphasize that the heterogeneous Cu SAC platform provides mechanistic information that is difficult to obtain from homogeneous systems. The isolated Cu sites are characterized by HAADF-STEM, XANES/EXAFS, ssNMR, and EPR, and their function in O_2 activation and charge transfer is supported by reactive oxygen species detection, radical trapping, in situ FTIR, kinetic analysis, and DFT calculations. Thus, the catalyst is not only recoverable but also mechanistically informative.

- Abstract

"Herein, we present a noble-metal-free, recyclable, and structurally defined heterogeneous photocatalytic platform for selective *N*-demethylation based on atomically dispersed Cu sites supported on functionalized graphitic carbon nitride (Cu@f-gC_3N_4). Under 390 nm irradiation and an oxygen atmosphere, a broad range of aliphatic, aromatic, and pharmaceutically relevant tertiary amines undergo selective *N*-demethylation or *N*-dealkylation, affording secondary amines in high yields. The reaction can be performed on a 10 mmol scale and the catalyst can be reused for more than ten cycles without significant loss of activity. The presence of atomically isolated Cu sites was confirmed through extensive characterization techniques including XPS, ssNMR, STEM, and EXAFS. Mechanistic studies, including radical trapping, isotope labeling, reactive oxygen species detection, kinetic analysis, in situ FTIR, and DFT calculations, support a photoredox pathway involving amine oxidation, O_2 reduction to superoxide, hydrogen abstraction, iminium formation, and hydrolysis. This work establishes atomically dispersed Cu@f-gC_3N_4 as a recyclable and mechanistically interrogable heterogeneous single-atom photocatalyst for this important transformation."

- Conclusion

"The significance of this work extends beyond replacing a homogeneous photocatalyst with a heterogeneous material. In contrast to noble-metal photoredox systems, the present platform uses earth-abundant Cu, allows catalyst recovery and reuse, and operates with molecular oxygen as the terminal oxidant. Moreover, the heterogeneous Cu@f-gC_3N_4 environment enables productive *N*-demethylation without sacrificial nucleophilic trapping additives such as nitromethane. This feature is synthetically important because the resulting secondary amine products can be directly engaged in downstream functionalization, as demonstrated by one-pot benzylation, allylation, and propargylation reactions. Thus, the process delivers not only demethylated products but also a practical entry to late-stage amine diversification. Mechanistic studies further show that the atomically dispersed Cu sites and the functionalized carbon nitride matrix jointly contribute to the reaction by

facilitating charge separation, O_2 activation, radical formation, iminium generation, and hydrolysis. HAADF-STEM, ssNMR, XANES/EXAFS, XPS, and EPR confirm the isolated Cu environment, while radical trapping, ROS detection, isotope labeling, kinetic analysis, in situ FTIR, and DFT calculations support the proposed radical/iminium pathway. Therefore, this study establishes Cu@f-gC_3N_4 as a structurally defined and mechanistically interrogable heterogeneous single-atom photocatalyst for sustainable *N*-dealkylation chemistry, providing a complementary platform to existing homogeneous photoredox methods."

4) Given the detailed catalyst design and characterization presented in the study, the reported quantum yield (AQY = 0.7%) appears relatively low. Furthermore, the method requires an oxygen atmosphere and reaction times similar to those reported in the literature. While these results do not diminish the overall value of the study, the authors should discuss more clearly what these observations imply regarding the overall efficiency and practical advantages of the developed catalytic system. At present, the method's most notable practical advantage appears to be the heterogeneous catalyst's ability to be recycled over multiple reaction cycles.

Response: We appreciate the reviewer's careful assessment. We agree that the apparent quantum yield of 0.7% is modest and that the present system is not yet optimized for maximum photon-to-product efficiency. At the same time, we have clarified that the small-scale AQY does not fully reflect the practical productivity of the reaction under scale-up conditions. The AQY was measured for the standard 0.2 mmol-scale reaction. When the reaction was scaled to 10 mmol, the substrate loading increased 50-fold and the product was still obtained in 71% yield, corresponding to approximately 40-fold more product formation, while the irradiation surface area increased only about fourfold. This demonstrates that the system maintains productive reactivity at a preparative scale and that reactor geometry strongly affects practical photochemical productivity.

Moreover, the catalyst can be recycled for more than ten cycles without significant activity loss, which improves the material efficiency of the process. Therefore, we now present the practical advantage of the system more precisely: not as superior AQY compared with all homogeneous systems, but as a recyclable, non-noble-metal, scalable heterogeneous photocatalytic platform.

- Abstract

"The reaction can be performed on a 10 mmol scale and the catalyst can be reused for more than ten cycles without significant loss of activity."

- Conclusion

"Under 390 nm irradiation, Cu@f-gC_3N_4 enables the conversion of diverse aliphatic, aromatic, and pharmaceutically relevant tertiary amines into secondary amines under mild conditions. And the reaction can be performed

on a 10 mmol scale, demonstrating the operational robustness of the system.”

- Apparent quantum yield calculation:

The amount of formed product (mol): M = 10 mmol x 71% = 7.1 mmol = 7.1 x 10^{-3} mol

The light intensity of Kessil LED lamp (λ = 390 nm, 40W) was 0,06 W/cm^2.

$$\eta = \frac{N_e}{N_p} \times 100\% = \frac{2 \times M \times N_A}{\frac{S \times P \times t}{h \times \frac{c}{\lambda}}} \times 100\% = \frac{2 \times M \times N_A \times h \times c}{S \times P \times t \times \lambda} \times 100\%$$

$$= \frac{2 * 7.1 \times 10^{-3} * 6.022 \times 10^{23} \times 6.63 \times 10^{-34} \times 3 \times 10^{8}}{6 \times \pi \times 0.06 \times 16 \times 3600 \times 390 \times 10^{-9}} \times 100\%$$

$$= 6.7\%$$

Where, M represents the amount of formed product (mol), N_A is the Avogadro constant (6.022x10^{23} mol^{-1}), h is the Planck constant (6.63x10^{-34} J·s), c is the light speed (3x10^8 m·s^{-1}), S is the irradiation area (6π cm^2), P is the light intensity (0.06 W·cm^{-2}), t is irradiation time (16 x 3600 s), λ is the wavelength of light (m).

5) Recyclability tests offer significant advantages. However, determining what forms the recycled catalyst takes after losing its activity following numerous catalytic cycles (If it loses after a certain number of cycles) will further support the catalyst's stability.

Response: We thank the reviewer for this helpful suggestion. Under the recycling conditions examined in this work, the catalyst retained its activity for ten consecutive cycles without significant loss of yield. Therefore, a genuinely deactivated catalyst could not be isolated within the tested recycling window. Nevertheless, we have strengthened the catalyst stability discussion by emphasizing the post-reaction characterization already performed. HAADF-STEM analysis of the catalyst after reaction shows that the principal structure is retained, and isolated Cu sites remain observable. In addition, the spectroscopic evidence for isolated Cu coordination supports the robustness of the Cu@f-gC_3N_4 structure under the reaction conditions.

6) Rather than simply presenting a comparative summary table in the Supporting Information section, it would be more beneficial to include a more detailed discussion of closely related recent studies on photoredox *N*-dealkylation within the main article. In particular, a clearer comparison of substrate scope, mechanistic similarities, and actual differences among the reported systems would significantly strengthen the discussion section.

Response: We agree with the reviewer. The revised manuscript now includes a main-text discussion comparing our Cu@f-gC_3N_4 system with closely related photoredox *N*-dealkylation studies. In addition to retaining the Supporting Information table as a concise reference, we have added a dedicated comparison in the main text. This discussion addresses substrate scope,

mechanism, catalyst identity, metal sustainability, trapping additives, scalability, recyclability, and post-synthetic utility. We believe this revision makes the conceptual contribution of the work clearer. The present study does not claim that the global *N*-dealkylation mechanism is unprecedented but rather demonstrates that this important photoredox transformation can be translated into a recyclable, non-noble-metal, heterogeneous Cu single-atom catalytic platform.

- Introduction

"Yilmaz and Emmert described a mild aerobic photoredox *N*-dealkylation of tertiary amines using an Ir-based photocatalyst and equimolar nitromethane as a nucleophilic trapping reagent, demonstrating broad substrate tolerance across aliphatic amines, aniline derivatives, and pharmaceutically relevant molecules.[15] These studies have established that oxidative *N*-dealkylation can proceed through pathways involving aminium radical cations, iminium intermediates, reactive oxygen species, and hydrolysis. However, most homogeneous photoredox systems for *N*-demethylation still face limitations from the perspective of catalyst sustainability and process design. Precious-metal photocatalysts, particularly Ir- or Ru-based complexes, are highly effective but can be expensive, difficult to recover, and less attractive for applications where catalyst recycling, product purification, and metal contamination are important considerations.[16] In addition, homogeneous systems provide limited opportunities to correlate a structurally defined catalytic site with charge separation, oxygen activation, radical generation, and substrate oxidation.[17] Thus, while the elementary reaction manifold of aerobic photoredox *N*-dealkylation is known, translating this chemistry into a recyclable, noble-metal-free, and mechanistically well-defined heterogeneous photocatalytic platform remains an important challenge."

- Conclusion

"The significance of this work extends beyond replacing a homogeneous photocatalyst with a heterogeneous material. In contrast to noble-metal photoredox systems, the present platform uses earth-abundant Cu, allows catalyst recovery and reuse, and operates with molecular oxygen as the terminal oxidant. Moreover, the heterogeneous Cu@f-gC_3N_4 environment enables productive *N*-demethylation without sacrificial nucleophilic trapping additives such as nitromethane. This feature is synthetically important because the resulting secondary amine products can be directly engaged in downstream functionalization, as demonstrated by one-pot benzylation, allylation, and propargylation reactions. Thus, the process delivers not only demethylated products but also a practical entry to late-stage amine diversification. Mechanistic studies further show that the atomically dispersed Cu sites and the functionalized carbon nitride matrix jointly contribute to the reaction by facilitating charge separation, O_2 activation, radical formation, iminium generation, and hydrolysis. HAADF-STEM, ssNMR, XANES/EXAFS, XPS, and EPR confirm the isolated Cu environment, while radical trapping, ROS

detection, isotope labeling, kinetic analysis, in situ FTIR, and DFT calculations support the proposed radical/iminium pathway. Therefore, this study establishes Cu@f-gC_3N_4 as a structurally defined and mechanistically interrogable heterogeneous single-atom photocatalyst for sustainable *N*-dealkylation chemistry, providing a complementary platform to existing homogeneous photoredox methods."

Reviewer #2 (Remarks to the Author):

A "hard-to-carry-on" reaction, *N*-demethylation, is made easier by a system envisaging atomically dispersed non-noble Cu species on a carbon nitride support, under visible light and in aerobic environment.

The work is sound, and the conclusions look well supported by the data. In particular, the authors were not only able to monitor the reaction progress, but also to trap some of the transient species, which gives remarkable insight on the actual photocatalytic mechanism.

I will focus on the computational part mostly, based on my expertise.

1. The authors follow an accurate procedure: after relaxing reactants and products at the GGA level, refinement calculations are done with hybrid functionals, as well as TD-DFT runs to evaluate the excitations related to the photocatalytic process. The solvent is described implicitely, unless a case where some molecules are explicitely included. It should be mentioned, though, at what level were the PDOS and orbital plots in Fig. 5 obtained.

Response: We thank the reviewer for this positive assessment of our computational procedure and for pointing out the missing methodological detail. We agree that the level of theory used to obtain the PDOS and orbital plots in Figure 5 should be explicitly stated. We have included the details related to the PDOS and orbital plots calculations in the SI.

"Solvent (ethanol) was implicitly described by using the SMD method. The PDOS and the MO iso-surfaces shown in Figure 5 of the main article have been derived with such an approach."

2. The structural model is, in general, adequate. I wonder, though, if a reactive species such as Cu1 is bound strongly enough to the matrix. Did the authors check its stability at room temperature through AIMD or similar?

Response: We thank the reviewer for this important and insightful comment. We agree that the stability of isolated Cu species within the carbon nitride matrix is an important point, especially because the proposed model involves atomically dispersed Cu sites that participate in the photocatalytic process. We checked the stability and behavior of the Cu atoms at room temperature by performing classical reactive molecular dynamics simulations based on ad hoc (tested and validated) force fields (ReaxFF flavor [Monti et al. J. Phys. Chem. C, 2013, 117, pp 5221-5228; Monti et al. Phys. Chem. Chem. Phys., 2013,15,

15062-15077; Rahaman et al. J. Phys. Chem. A 114 (2010), 3556-3568) in a more complex environment where all the species were considered at the atomic level, and reactivity was always on. We did not include these calculations/data in the manuscript or SI because the core of the study was based on quantum chemistry calculations. The classical RMDs were used solely to generate candidate reduced models to serve as starting points for the QC calculations. However, in response to the reviewer's comments, we have decided to include in the SI details of the simulations and relevant data showing the behavior of copper ions across different regions of the g-C_3N_4 catalyst.

Here are the added paragraphs with three Figures.

Preliminary model building and reactive molecular dynamics (RMD) simulations

We started the theoretical investigation by considering an extended atomistic model of the 2D catalyst material, including reactants, in an ethanol solution (**Figure S12**).

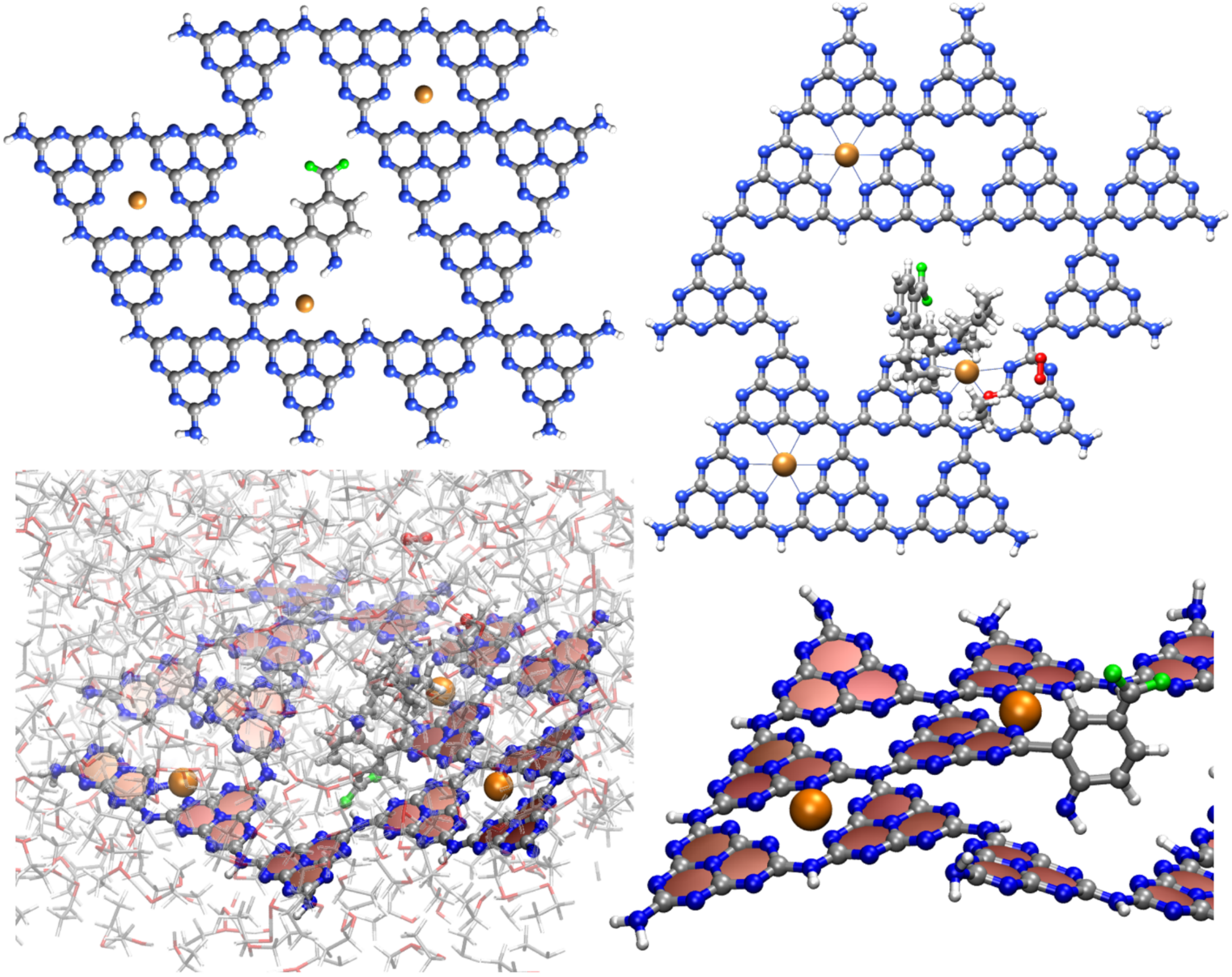

Figure S12. **Top left:** starting structure of the supramolecular model representing a portion of the catalyst with three Cu atoms and the functionalizing ligand. **Top-right:** optimized configuration where a tertiary amine is present (substrate 2 - **Figure 2** of the main article – ethanol molecules have been undisplayed for clarity). **Bottom left:** a snapshot from the RMD simulations at T=300K showing the evolution of catalyst and reactants in solution. **Bottom right:** evolved configuration of the

catalyst during its dynamics (extracted from an RMD snapshot). The color of the ring filling indicates their deformation relative to the flat arrangement (in practice, they preserve planarity). Color coding: C gray, O red, N blue, H white, F green, and Cu orange.

We checked the stability of the whole structure, and the dynamics of the Cu atoms at room temperature and pressure by performing RMD simulations based on *ad hoc* (tested and validated) force fields (ReaxFF flavor [Monti et al. J. Phys. Chem. C, 2013, 117, pp 5221-5228; Monti et al. Phys. Chem. Chem. Phys., 2013,15, 15062-15077; Rahaman et al. J. Phys. Chem. A 114 (2010), 3556-3568]) in a complex environment where all the species were considered at the atomic level, and reactivity was always on.

After the system optimization and equilibration for approximately 100 ps, the production RMD was performed in the NVT ensemble for about 800 ps. The time step was set to 0.2 fs, and the Anderson thermostat controlled the temperature with a relaxation constant of 0.1 ps. The system configurations were collected every 0.05 ps. The analysis of the sampled data was focused on the last 500 ps of the trajectories. We examined atom-atom radial distribution functions (RDFs), distance distributions, and distance evolutions.

All these calculations were carried out by the ReaxFF MD code available in the LAMMPS program [LAMMPS - a flexible simulation tool for particle-based materials modeling at the atomic, meso, and continuum scales, A. P. Thompson, H. M. Aktulga, R. Berger, D. S. Bolintineanu, W. M. Brown, P. S. Crozier, P. J. in 't Veld, A. Kohlmeyer, S. G. Moore, T. D. Nguyen, R. Shan, M. J. Stevens, J. Tranchida, C. Trott, S. J. Plimpton, Comp Phys Comm, 271 (2022) 10817]. Visual inspection of the collected snapshots was fundamental to understanding the reactive scenarios and the reorganization of the catalyst structure.

The snapshots revealed that the 2D polymeric framework underwent significant bending and twisting in the liquid environment, preserving the planarity of the individual heptazine/triazine rings, which were not deformed internally. Cu atoms shifted from their highly ordered, symmetrical locations, visible in the starting structure, to dynamic coordination environments during the system fluctuations. The rigid holes in the initial framework deformed flexibly to encapsulate the bulky functionalizing ligand and the tertiary amine substrate, stabilizing the intermediate states via supramolecular host-guest interactions. The ethanol solvent environment heavily crowds the catalyst, driving the conformational changes through continuous intermolecular collisions and solvation forces.

Having two distinct characteristic positions of the Cu atoms (depicted in **Figure S13**), namely a pristine region of the supramolecular framework (top panel), and a region featuring the attached functionalizing ligand (bottom panel), we

examined the behavior of the coordinated N atoms separately. **Figure S13** shows the distribution of the Cu-N distances, tracking the thermal structural fluctuations of the coordinated nitrogen atoms surrounding the copper centers. In both states, a highly pronounced, narrow pink peak is centered at approximately 1.85 Å. This corresponds to a strongly bound, highly stable chemisorption distance of the N directly coordinated to the copper center. The sharp distribution confirms that these core coordination bonds remain rigid and firmly bound during the dynamics. The broad distribution centered at approximately 3.3 Å in the top plot reflects the standard non-coordinating Ns farther in the unperturbed, open macrocyclic pocket. When the functionalizing ligand is present, the far-range black peak disappears and is replaced by the red distribution in the range 2.8-3.0 Å, suggesting a confinement effect due to the presence of the bulky ligand. Indeed, the distant Ns were forced closer to the active Cu center, modifying the local electronic environment and favoring the reaction kinetics. This picture was complemented by an examination of the Cu-N radial distribution function and the evolution of various Cu-N distances (**Figure S14**).

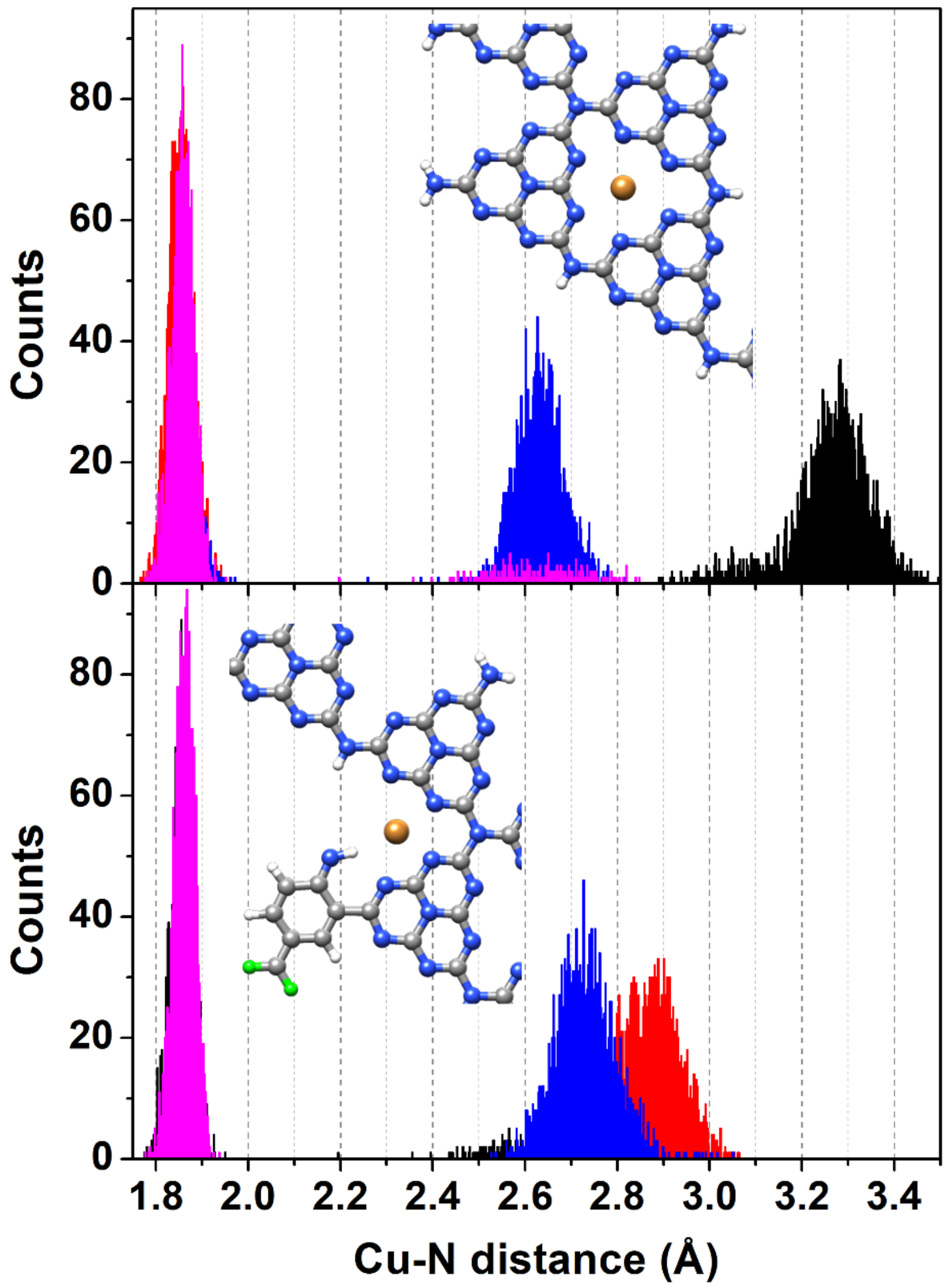

Figure S13. Distribution of Cu–N distances (Å) from RMD simulations, comparing unfunctionalized (**top**) and functionalized (**bottom**) supramolecular catalyst holes.

The very sharp, high-intensity RDF peak at ~1.85 Å (**Figure S14**) confirms a highly ordered and rigid first coordination shell between Cu and the closest framework N, whereas the other low peaks stretching out to 9 Å depict the maintained well-defined periodic organization of the successive nitrogen shells. The examination of the time-resolved Cu-N distances shows that structural rearrangement and requilibration can occur in the regions far from the ligand. Indeed, the lines corresponding to the ligand-functionalized pocket remain constrained between 2.6 Å and 2.9 Å, due to the pocket-locking effect of the bulky ligand.

These four structural views show the continuous, undulating, wave-like rippling of the polymeric sheet across the simulation, and the uniform color filling of the ring shows that individual triazine/heptazine rings remain perfectly flat. The entire sheet accommodates stress by bending, rather than breaking or distorting its chemical bonds. Besides, the ligand remained stably anchored on

the top ridge of the corrugated sheet, keeping its active site accessible to incoming substrates.

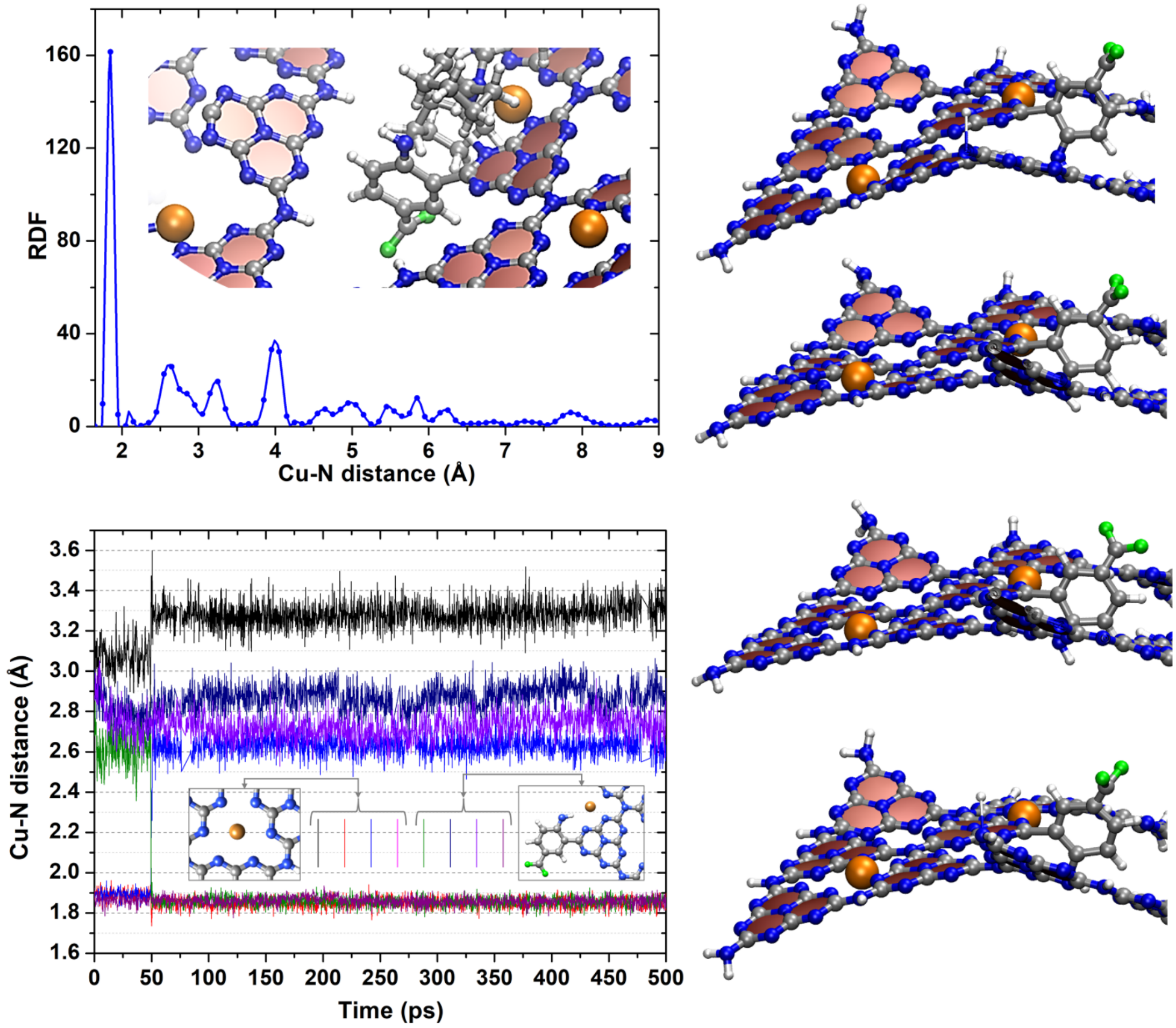

Figure S14. Structural evolution of the supramolecular catalyst showing long-range order through Cu–N distance radial distribution function (**top left**) and marked distance fluctuations in the pristine regions of the framework (**bottom left plots**). The simulation snapshots on the right depict macroscopic wave-like wrinkling of the 2D polymeric sheet in ethanol, while individual triazine/heptazine rings retain the local planarity.

3. A peculiar detail emerges in Fig. 5: the HOMO (or "VB" is mostly located on a phenyl substituent, whose presence is, in fact, a computational artifact to saturate the model, if I understand correctly. The real material does not envisage the presence of that type of phenyl substituent. Would the general photocatalytic mechanism that the authors depict hold true, even if the HOMO level was shifted down to the first available C_3N_4 state?

Response: We thank the reviewer for this careful observation. This phenyl substituent with fluorine atoms is not a computational artifact but a fundamental component of the catalyst, as already commented in the manuscript. It also induces a shift in the light-absorption wavelength of the g-C_3N_4 model from the UV region towards the LED emission wavelengths.

4. In general, what is the reference adopted for the energy scale in Fig. 5? Is it

consistent with the level alignment of the real system?

Response: We thank the reviewer for raising this important point. We agree that the reference adopted for the energy scale in Figure 5 should be explicitly clarified. Our model is not aligned with the "real system" (not properly defined, given the extreme complexity and heterogeneity of the material) because it is just a portion of it: it is only a finite molecular organometallic complex model, representing the local environment of the aryl ligand which is the active site of the photocatalyst. It was built without considering the periodic-boundary conditions of the g-C_3N_4 material. The energy scale used in the PDOS plots of Figure 5 derives from the energy levels of such a complex.

5. I find interesting that the Cu1 species is not involved in the photogeneration of charge carriers, and its oxidation state remains quite constant through the catalytic cycle (I am aware of similar evidences on comparable models, it is quite expected). One may thus wonder what is precisely its role... would bare C_3N_4 be equally active, based on the computational evidence?

Response: We thank the reviewer for this insightful comment. We agree that, according to the computational results, the primary photoexcitation is mainly associated with the functionalized g-C_3N_4 framework rather than a formal redox change at the Cu center. The calculated oxidation state of the Cu single site remains relatively stable throughout the modeled catalytic cycle, which is also consistent with related single-atom photocatalytic systems. However, this does not imply that the Cu site is catalytically inactive.

From a computational perspective, we have found that one of its roles is to strengthen molecular binding to the catalyst framework. As shown in the catalytic cycle of Fig. 5, the copper single atom serves as the direct coordination center across all intermediate states (1-5). It acts as a Lewis acid site that anchors the amine substrate. This localized coordination polarizes the reacting chemical bonds, bringing the reactants into the precise spatial alignment required for the reaction to progress.

The proximity to the metal center stabilizes the migrating hydrogen atoms, the radical species, the leaving amine group, and the resulting aldehyde intermediate, lowering the activation barrier compared to a metal-free pathway.

While the photoexcitation occurs primarily on the g-C_3N_4 framework, the PDOS in Figure 5 shows distinct Cu peaks (orange) near the band edges. The Cu center acts as a localized gateway or "sink" that traps the photogenerated carriers and directly funnels them into the targeted bonds of the coordinated substrate.

Without the single-atom metal site, bare g-C_3N_4 lacks these highly active, localized coordination environments. The chemically inert cavities of pristine carbon nitride cannot effectively bind or polarize the amine and O_2 molecules. Consequently, the activation energies for hydrolysis and C-N cleavage would be higher, making the metal-free support less active, or possibly entirely inactive, for this specific catalytic pathway.

6. Can the authors perhaps elaborate more on this? A further element to question on the actual photocatalytic role of Cu species is given by this very recent work: https://doi.org/10.1002/ange.2299555

Response: We thank the reviewer for bringing this very recent and highly relevant work to our attention. The cited paper provides a spectroscopically confirmed reason to question the active photocatalytic role of single-atom copper on carbon nitride supports, revealing that the smooth, continuous catalytic cycle often depicted for Cu may be an idealized model that fails in real systems due to irreversible structural collapse. The structural collapse appears to be prevented in this system due to the presence of the vicinal aryl ligand, which rigidifies the local nitrogen coordination environment. This rigidity imposes a significant steric and energetic penalty, thereby hindering the transition toward a linear geometry that would otherwise lead to an irreversible reduction of the metal site, as reported by Colonnello et al. [Angew. Chem. Int. Ed. 2026, 65, e2299555]. Furthermore, virtual states within the band gap of oxygen molecules facilitate the transfer of photoexcited electrons, thereby forming superoxide radical species. This mechanism prevents reduction of the copper center, allowing it to retain a +2 oxidation state throughout the catalytic cycle. Such behavior is consistent with the experimentally observed high recyclability of the photocatalyst.

This last paragraph has been added to the main paper in the discussion of the computational modeling.

"A recent paper by Colonnello et al.[39] provided a spectroscopically confirmed reason to question the active photocatalytic role of single-atom copper on carbon nitride supports, revealing that the smooth, continuous catalytic cycle often depicted for Cu may be an idealized model that fails in real systems due to irreversible structural collapse. However, in the present system, the structural collapse appears to be prevented, due to the presence of the vicinal aryl ligand, which rigidifies the local nitrogen coordination environment. This rigidity imposes a significant steric and energetic penalty, thereby hindering the transition toward a linear geometry that would otherwise lead to an irreversible reduction of the metal site, as reported in the cited paper. Furthermore, virtual states within the band gap of oxygen molecules facilitate the transfer of photoexcited electrons, thereby forming superoxide radical species. This mechanism prevents reduction of the copper center, allowing it to retain a +2 oxidation state throughout the catalytic cycle. Such behavior is consistent with the experimentally observed high recyclability of the photocatalyst."
